# Lipidomic analysis reveals sphingomyelin and phosphatidylcholine species associated with renal impairment and all-cause mortality in type 1 diabetes

**DOI:** 10.1038/s41598-019-52916-w

**Published:** 2019-11-08

**Authors:** Nete Tofte, Tommi Suvitaival, Linda Ahonen, Signe A. Winther, Simone Theilade, Marie Frimodt-Møller, Tarunveer S. Ahluwalia, Peter Rossing

**Affiliations:** 1 Steno Diabetes Center Copenhagen, Gentofte, Denmark; 2 University of Copenhagen, Copenhagen, Denmark

**Keywords:** End-stage renal disease, Diabetes complications

## Abstract

There is an urgent need for a better molecular understanding of the pathophysiology underlying development and progression of diabetic nephropathy. The aim of the current study was to identify novel associations between serum lipidomics and diabetic nephropathy. Non-targeted serum lipidomic analyses were performed with mass spectrometry in 669 individuals with type 1 diabetes. Cross-sectional associations of lipid species with estimated glomerular filtration rate (eGFR) and urinary albumin excretion were assessed. Moreover, associations with register-based longitudinal follow-up for progression to a combined renal endpoint including ≥30% decline in eGFR, ESRD and all-cause mortality were evaluated. Median follow-up time was 5.0–6.4 years. Adjustments included traditional risk factors and multiple testing correction. In total, 106 lipid species were identified. Primarily, alkyl-acyl phosphatidylcholines, triglycerides and sphingomyelins demonstrated cross-sectional associations with eGFR and macroalbuminuria. In longitudinal analyses, thirteen lipid species were associated with the slope of eGFR or albuminuria. Of these lipids, phosphatidylcholine and sphingomyelin species, PC(O-34:2), PC(O-34:3), SM(d18:1/24:0), SM(d40:1) and SM(d41:1), were associated with lower risk of the combined renal endpoint. PC(O-34:3), SM(d40:1) and SM(d41:1) were associated with lower risk of all-cause mortality while an SM(d18:1/24:0) was associated with lower risk of albuminuria group progression. We report distinct associations between lipid species and risk of renal outcomes in type 1 diabetes, independent of traditional markers of kidney function.

## Introduction

In current clinical practice eGFR and urinary albumin excretion rate (UAER) are measured as markers of renal status in diabetic nephropathy (DN). These biomarkers are strong predictors of progression in DN^1^, although neither is necessarily altered at the early stages of disease. Hence, there is a need to identify potential metabolites and novel pathways underlying development and progression of DN and/or future therapeutic targets.

In recent years, new omics-based techniques have emerged and allowed for the study of complex metabolic pathways. Lipidomics enables the comprehensive investigation and analysis of the lipid species present in biological systems^2^. Both intracellular and circulating lipids have been hypothesized to play a role in the pathogenesis of both diabetic and non-diabetic kidney disease^3,4^. In a review of basic animal and human studies, the evidence pointed towards intracellular sphingolipid accumulation in the glomeruli as a causal factor for glomerular proliferation and hypertrophy in DN^5^. In line with this, a study applying genome-wide transcriptome analyses on normal and fibrotic human kidney tubule samples demonstrated that fibrotic kidneys had lower expression of regulators of fatty acid oxidation and higher intracellular lipid deposition compared to normal kidneys^6^. In Fabry disease, a proteinuric kidney disease of genetic origin, targeting sphingolipid metabolism by enzyme replacement therapy has previously been demonstrated to ameliorate disease progression^7^, however, less is known about targeting the sphingolipid metabolism in non-genetic kidney diseases, for example DN.

Recent studies on serum/plasma lipidomics and diabetic kidney disease, in individuals with type 1 diabetes^8–10^ or type 2 diabetes^11–16^, have primarily examined cross sectional or longitudinal associations only with albuminuria status.

To our knowledge, the relation between blood lipidomics and development of end stage renal disease (ESRD) or mortality has never been examined in individuals with type 1 diabetes. This study measures serum lipidomic profiles in persons with type 1 diabetes and examines their cross-sectional association with eGFR and albuminuria. Further, this study has the advantage of also investigating the association of lipids identified in cross-sectional analyses, to longitudinal outcomes of renal impairment, ESRD and all-cause mortality.

## Results

The study included 669 Caucasian individuals with type 1 diabetes with a mean age of 55 ± 13 years, a median diabetes duration of 35 [24–44] years and 45% women (Table 1). Overall, 47% had persistent normoalbuminuria at baseline, 25% and 28% had a history of or current microalbuminuria and macroalbuminuria, respectively. At baseline mean eGFR was 81 ± 26 ml min^−1^ 1.73 m^−2^ and median UAER was 18 [8–64] mg/24-h. Number of individuals in each eGFR category (in ml min^−1^ 1.73 m^−2)^ were as follows: <15 (n = 2), 15–29 (n = 23), 30–44 (n = 50), 45–59 (n = 72), 60–74 (n = 83), 75–89 (n = 161) and >90 (n = 275), for three individuals eGFR at baseline was missing. During follow-up, 37 individuals progressed in albuminuria status, 93 participants experienced a decline in eGFR ≥ 30%, 21 individuals developed ESRD, 58 died and 125 had at least one event in the combined renal endpoint. Median follow-up time was 5.8 [2.5–6.4] years for assessment of albuminuria progression, 5.3 [2.7–6.2] years for ≥30% decline in eGFR, 5.3 [4.8–5.7] years for development of ESRD and 6.2 [5.8–6.7] years for all-cause mortality. The eGFR and UACR slopes were based on medians of 6 measurements in 516 participants and 17 measurements in 517 participants, respectively. The mean yearly change in eGFR was -0.9 ± 2.5 ml/min/year and the median yearly change in UACR was 3.5 [-13.0–8.7] %.

**Table 1:** Baseline characteristics according to albuminuria group

	All participants (*n* = 669)	Normoalbuminuria *(n* = *312)*	Microalbuminuria *(n* = *168)*	Macroalbuminuria *(n* = *189)*	Normo- vs. micro- vs. macro-albuminuria *p*
Female, n (%)	299 (45)	155 (50)	62 (37)	81 (43)	0.031
Age, years	55 ± 13	53 ± 14	58 ± 13	55 ± 10	<0.001
Diabetes duration, years	35 [24–44]	30 [9–41]	36 [26–48]	39 [31–45]	<0.001
Body mass index, kg/m^2^	26 ± 6	25 ± 4	26 ± 4	26 ± 9	0.036
Systolic blood pressure (mmHg)	132 ± 18	129 ± 16	134 ± 18	134 ± 19	0.001
Diastolic blood pressure (mmHg)	74 ± 9	75 ± 9	73 ± 9	74 ± 10	0.030
HbA_1c_, % (mmol/mol)	8.0 ± 1.2 (64 ± 13)	7.8 ± 1.1 (62 ± 12)	8.1 ± 1.2 (65 ± 13)	8.4 ± 1.2 (68 ± 13)	<0.001
Total cholesterol, mmol/l	4.7 ± 0.9	4.7 ± 0.8	4.7 ± 0.9	4.6 ± 1.0	0.360
LDL cholesterol, mmol/l	2.5 ± 0.8	2.5 ± 0.7	2.4 ± 0.8	2.5 ± 0.8	0.639
HDL cholesterol, mmol/l	1.7 ± 0.5	1.8 ± 0.6	1.7 ± 0.5	1.6 ± 0.5	0.001
Triglycerides, mmol/l	1.0 [0.7–1.3]	0.9 [0.7–1.2]	0.9 [0.7–1.4]	1.1 [0.8–1.5]	0.001
eGFR, ml/min/1.73 m^2^	81 ± 26	92 ± 18	82 ± 23	63 ± 28	<0.001
^a^UAER, mg/24-h	18 [8–64]	8 [6–12]	33 [17–61]	137 [32–479]	<0.001
Retinopathy grade, n (%) Nil Simplex Proliferative Blind	142 (24) 277 (42) 228 (34) 19 (3)	108 (35) 149 (48) 49 (16) 3 (1)	26 (15) 79 (47) 59 (35) 4 (2)	8 (4) 49 (26) 120 (63) 12 (6)	<0.001
Smokers, n (%)	139 (21)	59 (19)	32 (19)	48 (25)	0.181
RAAS inhibition treatment, n (%)	450 (67)	134 (43)	138 (82)	178 (94)	<0.001
Statin treatment (n, %)	401 (60)	129 (41)	113 (68)	158 (84)	<0.001
**Follow-up**					
Progression in albuminuria group, n (%)	37 (6)	19 (6)	18 (11)	NA	NA
Decline in eGFR ≥ 30%, n (%)	93 (14)	10 (3)	19 (11)	64 (34)	<0.001
End stage renal disease, n (%)	21 (3)	0	3 (2)	20 (11)	<0.001
All-cause mortality, n (%)	58 (9)	12 (4)	21 (13)	25 (13)	<0.001
Combined renal endpoint, n (%)	123 (18)	16 (5)	29 (17)	80 (42)	<0.001

Data are n (%, rounded), mean ± SD or median [IQR]. NA not applicable, UAER urinary albumin excretion rate, RAAS renin-angiotensin-aldosterone system. The combined renal endpoint consisted of ≥30% decrease in eGFR from baseline, ESRD and all-cause mortality ^a^Some individuals with previous persistent micro- or macroalbuminuria had lower values at baseline due to medication.

### Cross-sectional associations of lipid classes with eGFR or albuminuria

In total, 106 known lipids from 5 classes (diacyl-phosphatidylcholines (PCs), alkyl-acyl-phosphatidylcholines (PC-Os), lyso-phosphatidylcholines (LPCs), triacylglycerols (TGs), and sphingomyelins (SMs)) were identified and included in the analyses. The dominance of individual lipid species within their respective lipid classes are listed in the Supplementary Report, p. 173–175.

**Figure 1:**
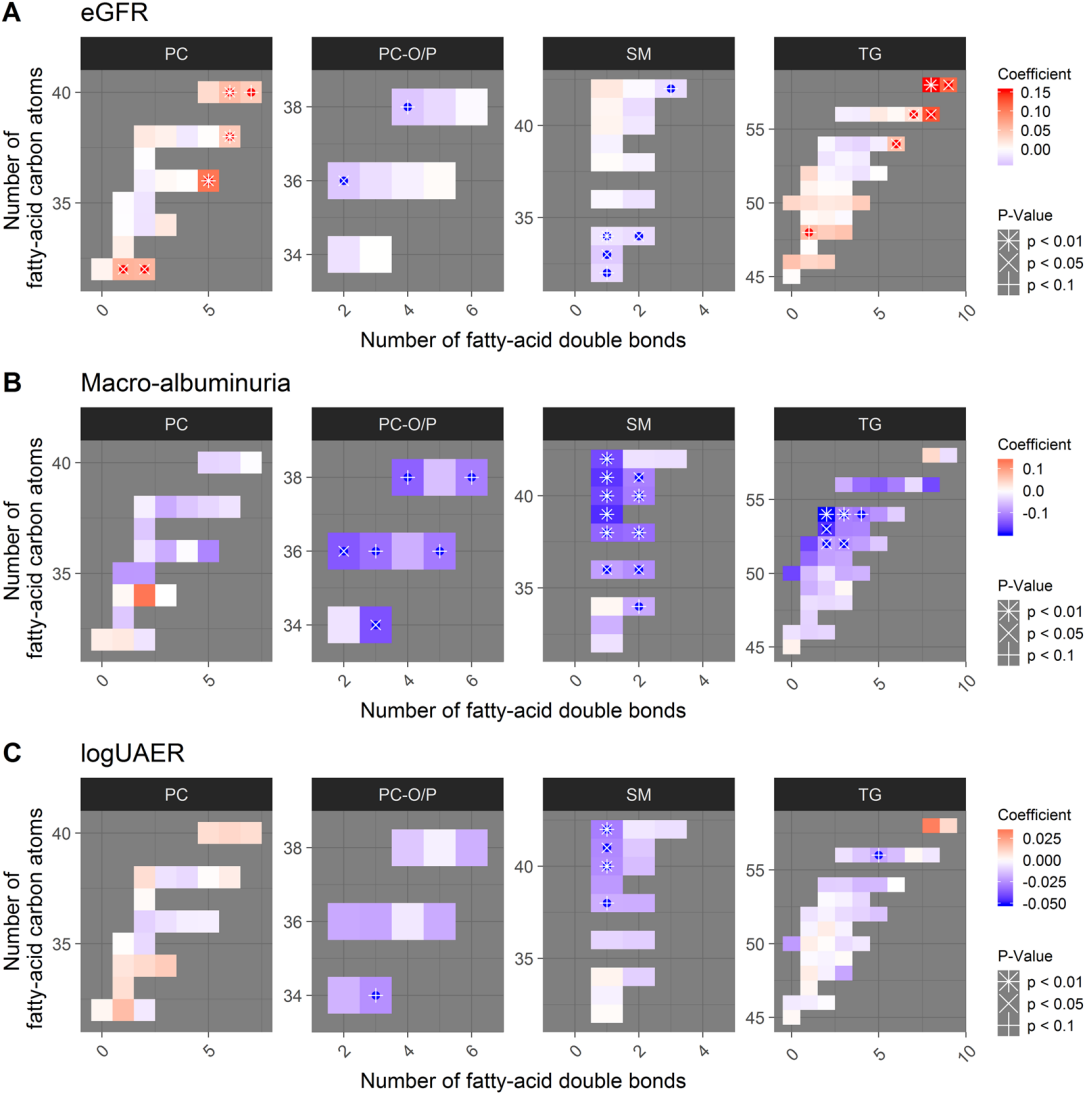
Cross-sectional associations of lipid species and (**A**) eGFR intervals, (**B**) macroalbuminuria (n = 189) vs. normoalbuminuria (n = 312), and, (**C**) logUAER. Lipid species are grouped into panels according to the lipid classes shown in the title of each panel. Each cell in the heatmap represents one lipid species. On the x-axis is number of double-bonds for the specific species (indicating level of unsaturation) and on the y-axis is the number of carbon atoms (indicating total fatty acid chain length). The coefficients arise from adjusted logistic regression models, individual coefficients are shown in the Supplementary Report, p. 19, 37 and 48. Red colours represent positive associations and blue colour negative associations. P-value: ^#^<0.01, x < 0.05 and ≥0.01, *<0.1 and ≥0.05, respectively after correction for multiple testing. PC: Phosphatidylcholine, PC-O: alkyl-acyl-phosphatidylcholine, TG: triglyceride, SM: Sphingomyelin, UAER: urinary albumin excretion rate.

In the cross-sectional analyses, eGFR was inversely associated with SMs and PC-Os, and positively associated with PCs and TGs (Fig. 1A). Also, eGFR positively associated with two short-chain LPCs (Supplementary Report, p. 37).

Participants in the macroalbuminuria group had lower levels of SMs, PC-Os and medium- length TGs compared to the normo-albuminuria group (Fig. 1B). There were no significant differences between the microalbuminuria and normoalbuminuria groups (Supplementary Report, p.17). SM(d40:1), also known as C22:0 SM, and SM(d42:1), also known as C24:0 SM, were inversely associated with UAER (Fig. 1C).

### Lipid species associated with eGFR or albuminuria slope

PC(36:4) was inversely associated with eGFR slope, both, in a crude model (β = -0.013 per 1 SD increase in eGFR; p_BH_ < 0.1; Supplementary Report, p.55) and in an adjusted model (β = -0.014; Supplementary Report, p.80). LPC(18:2), seven PC-Os and SM(d18:1/24:0) were all inversely associated with albuminuria slope in a crude model (p_BH_ < 0.1; Supplementary Report, p.63 and 73). Only LPC(18:2) among these did not remain significant in an adjusted model (Supplementary Report, p.84).

None of the lipid species selected ((p < 0.1) in the adjusted cross-sectional analyses of eGFR) were significantly associated to eGFR slope (Supplementary Report, p.76). In the similar analysis involving UACR slope, four SMs, PC(O-34:3) and TG(18:0/18:1/20:4) all had significant inverse association to UACR slope (p_BH_ < 0.1; Supplementary Report, p.73).

### Longitudinal outcomes of renal impairment, ESRD and all-cause mortality

**Figure 2:**
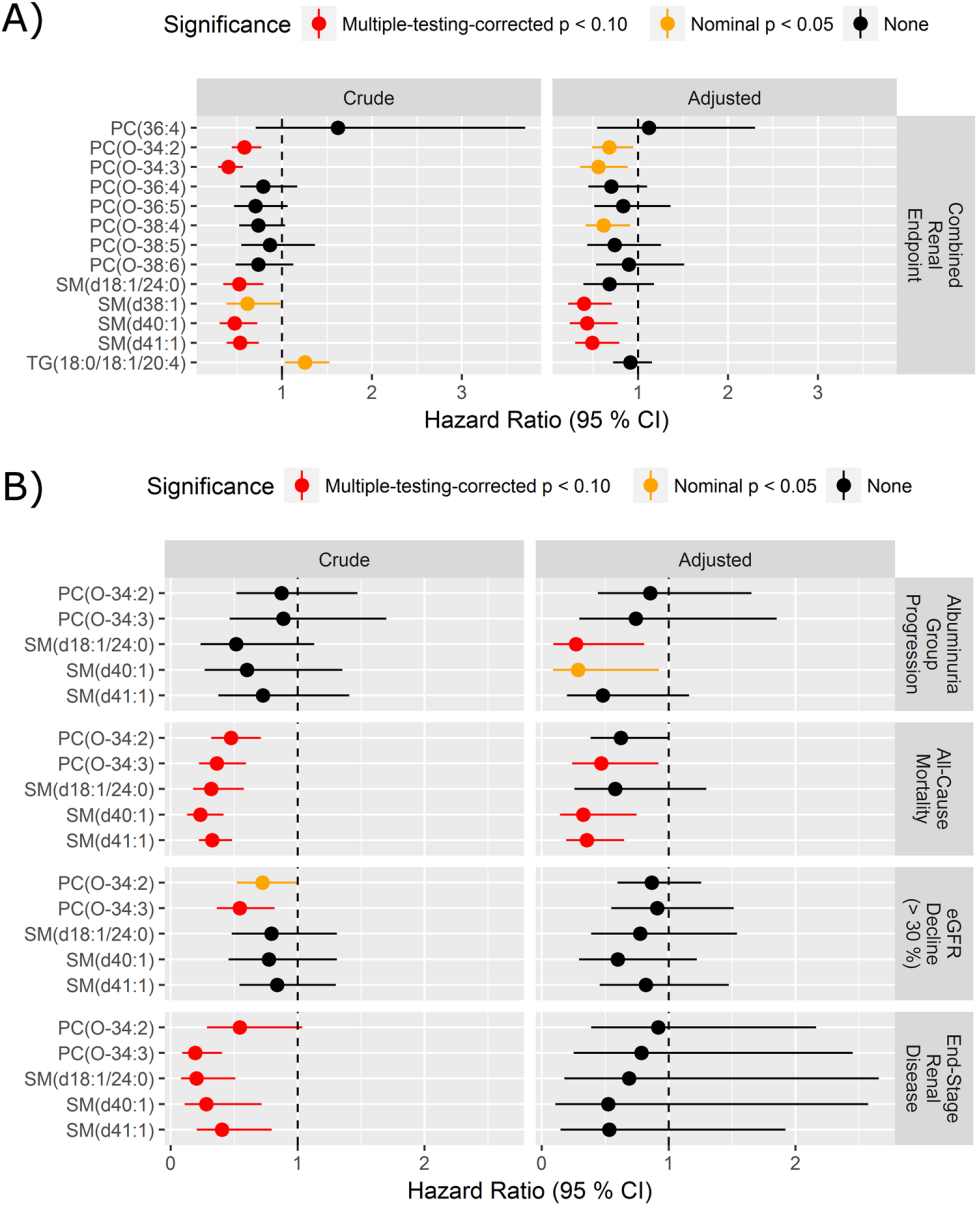
Survival analysis of prioritized lipid species. (**A**) Lipid species with adjusted p-values < 0.1 in analyses of association with eGFR or logUACR slope entered the Cox proportional hazard model for progression to the combined renal endpoint consisting of ≥30% decrease in eGFR, ESRD and all-cause mortality with time to first event. (**B**) Significant lipids from the crude model of the combined endpoint are tested for each separate endpoint with time to each type of event. HRs are presented per 1 SD increase of the log2 lipid. Adjusted HRs (right) controlled for age, sex, HbA_1c_, systolic blood pressure, smoking, BMI, statin treatment, p-triglycerides, total p-cholesterol, eGFR and logUAER. PC phosphatidylcholine, TG triacylglycerol, SM sphingomyelin.

Thirteen lipid species significantly associated with either eGFR or albuminuria slope were further analysed using the Cox proportional hazards regression models to assess the risk of having at least one renal event (combined renal endpoint). Higher levels of three SMs and two PC-Os were associated with lower risk and one TG with higher risk (p_BH_ < 0.1) in crude models (Fig. 2A, left). After multivariate adjustment and correction for multiple testing, three sphingomyelins were significant as follows (Fig. 2A, right): SM(d38:1), also known as C20:0 SM, (HR (95% CI) 0.39 (0.23–0.71), p_BH_ = 0.023), SM(d41:1), also known as C23:0 SM (HR 0.49 (0.30–0.79), p_BH_ = 0.042) and SM(d40:1), also known as C22:0 SM, (HR 0.43 (0.24–0.77), p_BH_ = 0.053).

When computing HRs for progression in albuminuria group and each of the outcomes included in the combined renal endpoint (Fig. 2B), higher PC-Os and SMs were associated with lower risk of ESRD and all-cause mortality in the crude models (p_BH_ < 0.1). Two sphingomyelins (SM(d41:1): HR 0.35 (0.19–0.65), p_BH_ = 0.0040; and SM(d40:1): HR 0.33 (0.14–0.75), p_BH_ = 0.032) and PC(O-34:3) (HR 0.47 (0.24–0.92), p_BH_ = 0.083) remained significant for all-cause mortality. However, significance for ESRD was lost in the adjusted models.

**Figure 3:**
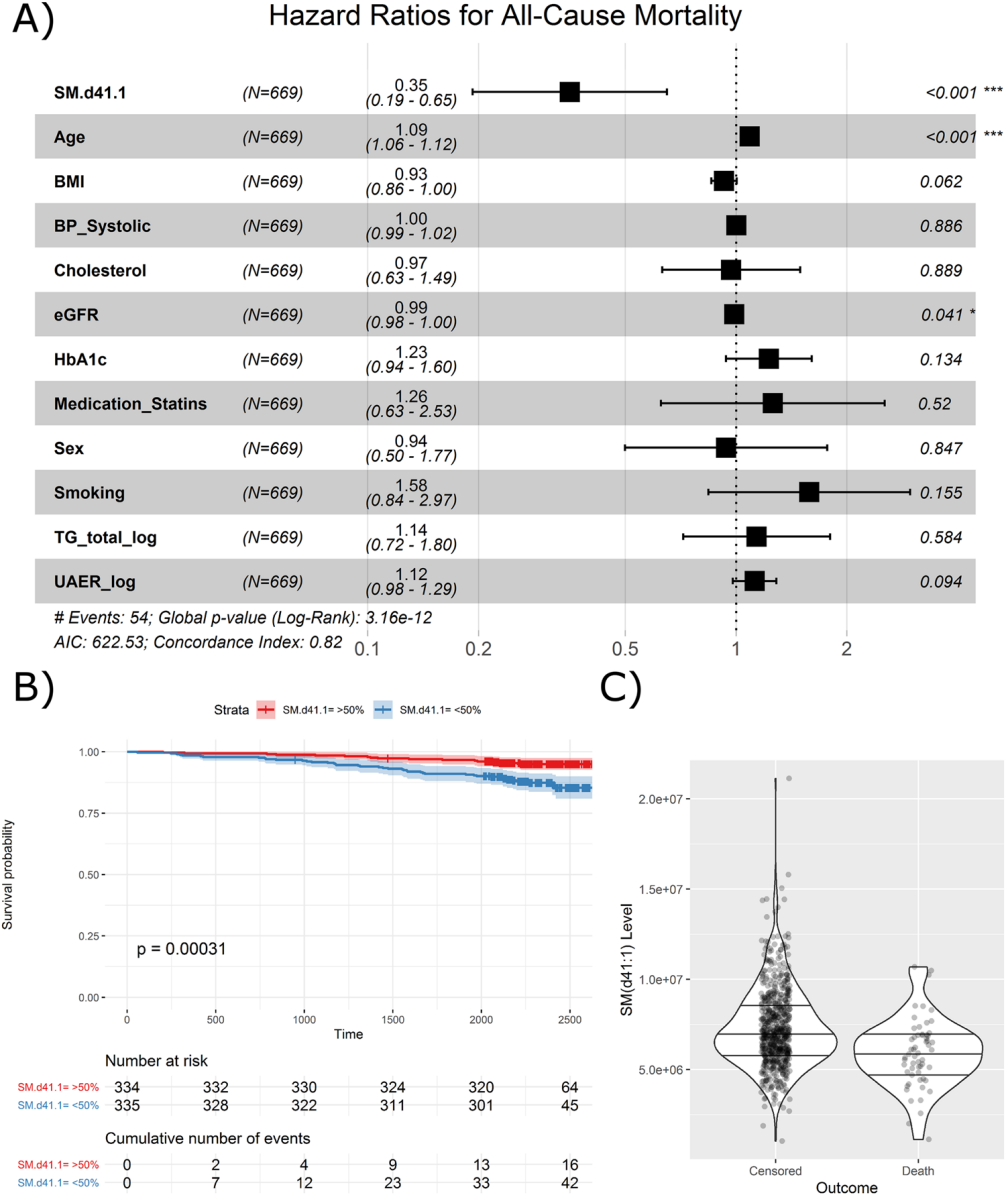
Survival analysis of SM(d41:1) and clinical covariates for all-cause mortality. (**A**) Forest plot of SM(d41:1) and clinical covariates from multivariate analyses. HRs are presented per 1 SD increase. (**B**) Kaplan-Meier curve demonstrating survival probability for levels of SM(d41:1) above (red) and below (blue) the median. (**C**) Distribution of individual SM(d41:1) measurements in a violin plot. Horizontal lines represent the median and interquartile ranges. SM Sphingomyelin.

In addition to the top-ranking lipid species, SM(d41:1), age was also significantly associated with the risk of all-cause mortality (Fig. 3A). Association to all-cause mortality was also visually demonstrated in the Kaplan-Meier curves of the top and bottom 50% quantiles of SM(d41:1), where individuals with values below the median had a higher risk (Fig. 3B). At baseline, the median level of SM(d41:1) in the deceased individuals was equal to the 25% quartile in the subjects alive at study end (Fig. 3C).

Similar trends as observed with ESRD and all-cause mortality were present between higher levels of SMs and PC-Os and lower risk of ≥30% decline in eGFR or albuminuria group progression. Particularly, SM(d18:1/24:0) was associated with albuminuria group progression in the adjusted model (HR: 0.27 (0.09–0.81), p_BH_ = 0.096) although this association was not present in the crude model.

### Prediction models for all-cause mortality

After performing c-statistics for predictions of all-cause mortality, the area under the curve (AUC) for the model with clinical variables only was 0.77 (0.72–0.80). By adding the significant lipid species to the model, the AUC did not improve significantly, AUC 0.79 (0.74–0.81), p = 0.23. The relative integrated discrimination improvement (rIDI), between a model including traditional risk factors and a model including clinical variables and significant lipid species, however, was 16%, (p = 0.045).

### Correlation of lipid species and clinical covariates

In a Spearman correlation matrix including significant lipid species, clinical covariates and endpoints (Supplementary Report, p.161), strong positive correlations were demonstrated between total cholesterol and SMs. Individual SMs were positively correlated as were individual PC-Os.

### Sensitivity analyses

More extensive models with further adjustments were also computed, including antihypertensive medication, diabetes duration, HDL-C, LDL-C, VLDL-C, as well as technical adjustments for sampling date, analysis batch, and analysis order. These additional adjustments did not significantly influence the results, and thus are not shown.

## Discussion

We examined serum lipidomic profiles in persons with type 1 diabetes to evaluate potential cross-sectional and longitudinal associations with renal function (eGFR) and albuminuria as the clinical features of diabetic nephropathy. Additionally, with the follow up information of the cohort we analysed longitudinal associations to development of renal impairment, ESRD and mortality. The key findings can be summarized as follows: (1) cross-sectional inverse associations of PC-Os and SMs with eGFR, (2) inverse cross-sectional associations of PC-Os and SMs with macroalbuminuria, and, (3) in longitudinal analyses, higher levels of specific PC-Os and SMs being associated with lower risk of the combined renal endpoint, ESRD and all-cause mortality.

We recognize some limitations in our study. The main limitation is the lack of a replication cohort. Replication in an independent cohort would be a requirement for assessing the lipid species as potential biomarkers. Since the follow up part of the study was based on available measurements performed at outpatient visits and therefore not performed according to a specified protocol, the definition of persistent albuminuria progression may have been too conservative. As only one annual assessment of albuminuria was available for many participants, it would take longer to develop confirmed progression in albuminuria than if we had three consecutive measurements per visit. Since albuminuria has significant day-to-day variation, using a single elevated albuminuria measurement for definition of progression would also not be valid. Data on diet, stress and exercise, which may affect the specific lipids^17^, were not available. Data concerning potential changes in concomitant medication during follow-up were not available, and particularly changes in antihypertensive and lipid lowering medication could have been relevant. The major strengths of this study are the large population of individuals with type 1 diabetes, the well-described comprehensive analyses of lipid species, and, the availability of longitudinal register data for up to 7 years.

Overall, there are few comprehensive investigations of global lipidomics in longitudinal studies of DN. Many of these studies have focused on both polar and non-polar metabolites (lipids) and most were cross-sectional^11–13,15,16^. The prior longitudinal studies have primarily focused on albuminuria progression as the primary endpoint^8,14^.

The cross-sectional results demonstrated that PC-Os and SMs were inversely associated with eGFR levels. These results seem contradictory to their associations observed with albuminuria, which are also inverse, cross sectionally (Fig. 1A,B). The effect sizes, though, were numerically small. However, longitudinally the PC-Os and SMs associate negatively with lower eGFR and albuminuria, as expected (Fig. 2B). In the longitudinal analyses, one PC specie (PC(36:4)) also demonstrated a significantly positive association with eGFR slope. Part of the explanation for these weak and contradictory seeming associations with eGFR (cross-sectionally) may be attributed to glomerular hyperfiltration, a phenomenon that is often present at the very early stages of DN.

PC-Os and SMs were inversely associated with macroalbuminuria compared to normoalbuminuria in our cross sectional analyses whereas a study from Finland by Mäkinen *et al*., including 326 individuals with type 1 diabetes, reported higher serum levels of SMs to be associated with presence of macroalbuminuria^9^. Another Finnish study comprising 325 individuals with type 1 diabetes observed higher SMs and lower PC levels associated with progression of albuminuria over 8.3 years^10^. In these Finnish studies, a different platform (proton nuclear magnetic resonance (NMR)) was used, and therefore it may be difficult to compare them to the present study. In our study the SMs were positively correlated with total plasma cholesterol levels, as demonstrated in the correlation matrix (Supplementary Report, p.161). NMR only quantifies the total SM in contrast to the multiple SM species measured with the MS. Further, NMR in general measures larger lipid particles, which may have higher correlation to total plasma cholesterol levels. Moreover, the participants in the Finnish studies were younger and the age difference between the albuminuria groups was larger than in the present study, which could contribute to the differing results.

A recent two stage cross-sectional study from Singapore performed in 409 subjects in the discovery and 298 subjects in the validation cohort analysed both polar and non-polar metabolite levels among individuals with type 2 diabetes and varying degrees of diabetic kidney disease^16^. They observed lower PC and higher long-chain SM and ceramide (precursors of sphingolipids) levels associated with a higher degree of albuminuria. Differing disease aetiology (type 1 and type 2 diabetes), ethnicity and presumably large dietary (or environmental) differences could potentially explain the differing results.

In the longitudinal analyses in the present study, higher levels of PC-Os and SMs were associated with lower risk of a combined renal endpoint, ESRD and all-cause mortality, although an accurate estimation of adjusted HRs would require more events. Moreover, a trend towards higher levels of SMs being associated with progression in albuminuria status was observed. In a previous study of a subset of 497 individuals with type 1 diabetes from the Diabetes Control and Complications Trial, with a follow-up of 14–20 years, lower plasma levels of very long chain ceramide species (precursors of sphingolipids)) were also associated with albuminuria progression^8^. Also in line with our findings, a previous case-control study demonstrated lower levels of PCs and other lipid species, in 79 subjects who progressed to ESRD, compared to 121 non-progressors, (including 50% with diabetes) over 6 years of follow-up^18^.

The prioritized lipid species that we investigated in the longitudinal models of ESRD and all-cause mortality were mostly associated with albuminuria slope. However, these lipids were not associated with eGFR. Albeit albuminuria and decline in eGFR are considered markers of glomerular damage, there is growing evidence that albuminuria is predominantly of tubular origin (proximal tubule) and somewhat independent of glomerular barrier^19^.

Although untargeted lipidomics detects a broader panel of lipid species, and we here identified 5 out of 17 classes (that were seemingly targeted lipidomics), this shows that the current approach may be conservative but has undergone robust QC methods (SRM based) reflecting high quality which is next to validation.

In conclusion, altered lipidomic profiles were associated with renal impairment independent of traditional markers of kidney function. Higher levels of specific alkyl-acyl-phosphatidylcholines and sphingomyelins species were associated with lower risk of ESRD and all-cause mortality, in type 1 diabetes. These results support the hypothesis of altered lipid metabolism in renal complications, however, need external validation to assess their potential as biomarkers as well as exploration of potential future therapeutic options targeting lipid metabolism.

## Methods

### Participants

A cohort of 676 individuals with type 1 diabetes followed at Steno Diabetes Center Copenhagen between 2009 and 2011 was included in a cross-sectional study and a biobank was created. The selection process has previously been described in detail^20^. The cohort was selected to include individuals within a wide range of albuminuria (normo- to macroalbuminuria). Participants were stratified as normoalbuminuric if UAER was <30 mg/24 h or mg/g, as microalbuminuric if UAER was or previously had been recorded between 30 and 299 mg/24 h or mg/g, and as macroalbuminuric if UAER was or previously had been recorded ≥300 mg/24 h or mg/g in two out of three consecutive measurements. All subjects classified as normoalbuminuric did not have any history of micro- or macroalbuminuria prior to enrolment in the study. Individuals with ESRD defined as either receiving dialysis, renal transplantation or having a GFR/ eGFR < 15 mL/min/1.73 m^2^ were excluded from the study.

In the current study, plasma samples from 669 (99%) individuals from the biobank were available for serum lipidomics analysis which was performed in 2017.

The study was conducted in accordance with the Declaration of Helsinki. The ethics committee E, Region Hovedstaden, Denmark, approved the original as well as the follow-up research study protocol, and all participants gave a written informed consent.

### Study baseline (biochemical and other) measures

UAER was measured at baseline in three consecutive 24 h urine collections by enzyme immunoassay (mg/24 h). The eGFR was calculated from serum creatinine (determined by an enzymatic method; Hitachi 912; Roche Diagnostics, Mannheim, Germany) using the Chronic Kidney Disease Epidemiology Collaboration (CKD-EPI) equation^21^. Information on medication was collected from electronic medical records at study baseline.

### Lipidomic analyses, data pre-processing and quality control

The serum samples were stored at −80 °C until analysis. Samples were prepared and analysed using previously published methods at Steno Diabetes Center Copenhagen^22,23^. The Folch procedure^24^ was used for sample preparation with minor modifications. The samples were analysed using an in-house ultra-high-performance liquid chromatography quadrupole time-of-flight mass spectrometry method (UHPLC-QTOF/MS)^22,23^. The UHPLC system was a 1290 Infinity system from Agilent Technologies (Santa Clara, CA, USA). A detailed description is available in the Supplementary Methods and a complete list of all identified and included lipids in the Supplementary Report, p.5–7. The lipidomics method has been cross-validated in an interlaboratory study including 31 European laboratories that used the National Institute of Standards and Technology (NIST) Standard Reference Material (SRM) 1950-Metabolites in Frozen Human Plasma^25^. The lipidomics data were pre-processed with MZmine2^26^. Subsequent quality control, post-processing and data analysis were done in R. In brief: (1) lipids were semi-quantified by normalizing the peak areas to internal standards^27^; (2) systematic day-to-day variation in the measurements was removed by median batch correction^28^, (3) Lipids with more than 20% missing/undetected values were omitted from subsequent analysis; (4) remaining missing values were imputed with the k-nearest neighbour algorithm^29^; and (5) all values were log-2-transformed to achieve normal-distributed data.

### Follow-up

All participants were traced through the Danish National Death Register and the Danish National Health Register until 31st of December 2016^30,31^. Information was obtained regarding the date and cause of death for deceased participants; data on the cause of death was available only until 31^st^, December 2015. All information concerning hospital admission and related international classification of diseases, tenth revision (ICD-10) diagnoses (www.who.int/classifications/icd/en/) and procedural codes (according to the Nordic Classification of Surgical Procedures; www.sst.dk) were obtained from the Danish National Health Register. Biochemical measurements (p-creatinine (determined by an enzymatic reaction and urinary albumin to creatinine ratio (UACR)) were obtained from the local electronic laboratory records. The follow up part of the study was not designed as a clinical trial with measurements of albuminuria on consecutive days, single assessments of albuminuria with differing time intervals were available. Data regarding potential changes in medication during follow-up were not available.

Progression in albuminuria status was defined as progression from normo- to microalbuminuria, or micro- to macroalbuminuria in at least two out of three consecutive measurements during follow-up. The time of progression was defined as the time of the first elevated measurement. Participants with history of macroalbuminuria were excluded from analyses of albuminuria progression. Albuminuria slope was calculated based on all the available measurements from outpatient visits during follow-up, in participants with at least 2 measurements and a minimum follow-up duration of 3 years (n = 517). Decline in eGFR was assessed as time to the first occurrence of ≥30% decrease from baseline, as proposed by Coresh *et al*.^32^ and as eGFR slope. ESRD was defined as chronic kidney disease stage 5 (ICD-10 code N18.5), chronic dialysis (procedural code BJFD2), kidney transplantation (procedural code KKAS 00, 10 and 20) or eGFR < 15 ml/min/1.73 m^2^. A combined renal endpoint included ≥30% decline in eGFR, ESRD and all-cause mortality. The combined endpoint is in line with the hard renal endpoints used in clinical outcome trials, although modified in terms of eGFR decline since this is an observational study with no initial decline in eGFR due to intervention. Participants who experienced multiple events were in the analyses followed until occurrence of the first event within each outcome.

### Statistical analyses

Continuous variables were reported as mean ± SD for normal-distributed data. Skewed data were reported as median [interquartile range (IQR)] and were log2-transformed for analyses. Categorical variables were presented as total numbers with corresponding percentages. Comparisons of continuous variables between groups were performed using the ANOVA. The chi-squared test was used to compare categorical variables.

Lipid species were tested for association with kidney-related clinical variables and outcomes in a narrowing-down approach (Supplementary Fig. s1) as follows: Cross-sectional associations between single lipid species and eGFR (15 units and a single category for values above 90 ml/min/1.73 m^2^), logUAER or albuminuria groups (normo- vs. microalbuminuria and normo- vs. macroalbuminuria) were assessed with linear regression models adjusted to clinical variables. The adjustment variables were the baseline values of age, BMI, HbA_1c_, p-triglycerides, sex, smoking, statin treatment, systolic blood pressure, total p-cholesterol. Baseline eGFR and logUAER, respectively, were also included as adjustments in models, where the investigated association itself was not baseline eGFR or logUAER. P-values for each analysis were corrected for multiple testing using the Benjamini-Hochberg method (p_BH_)^33,34^. Lipid species with p_BH_ < 0.1 in an adjusted cross-sectional model were included in longitudinal analyses comprising eGFR and albuminuria (logUACR) slopes. Additionally, longitudinal associations between all lipid species and eGFR and albuminuria slopes were examined under a crude model adjusting only for the baseline measure of the outcome variable.

Lipid species associating either in the cross-sectional adjusted model or longitudinal crude model (p_BH_ < 0.1) were further tested longitudinally, adjusting for the baseline values of the eleven clinical variables listed above.

Lipids with an association in the longitudinal adjusted model (p_BH_ < 0.1) were further examined using survival analysis with the Cox proportional hazards model. First, HRs with 95% CI on the log2-scale, were computed for the combined renal endpoint, both, in crude and adjusted models. Lipids associating with the combined renal endpoint in the crude model (p_BH_ < 0.1) were included for testing of four specific endpoints: albuminuria group progression, ≥30% decline in eGFR, ESRD and all-cause mortality.

Finally, the lipid species most significantly associated with all-cause mortality in the adjusted model was investigated in detail. The survival for participants with lipid levels above or below the median were visualized as Kaplan-Meier curves.

Visualizations were done in R (https://www.r-project.org/): Coefficients from lipid-specific linear models were visualized as heatmaps. Coefficients of the lipids from survival models were visualized in forest plots grouped by the dependent variable. The full forest plot of the model with the top-ranking lipid from the adjusted analysis of all-cause mortality was visualized along with the respective Kaplan-Meier curve and outcome-specific violin plots. A Spearman correlation matrix including significant lipid species, clinical covariates and endpoints was computed. Data analysis was performed with SAS Enterprise Guide version 7.11 and R-3.4.2.

## Supplementary information

Supplementary file

## Data Availability

Data available on request for researchers who have the relevant legal permissions to access the data.
