# Supplementary material for: Lipidomic analysis reveals sphingomyelin and phosphatidylcholine species associated with renal impairment and all-cause mortality in type 1 diabetes
Source: Sci Rep. 2019 Nov 8;9:16398. doi: 10.1038/s41598-019-52916-w (PMC6841673; doi:10.1038/s41598-019-52916-w)
Supplement: Supplementary file 1 — Supplementary file [file 41598_2019_52916_MOESM1_ESM.pdf]

Supplementary

Lipidomic analysis reveals sphingomyelin and phosphatidylcholine species associated with renal impairment and all-cause mortality in type 1 diabetes

Nete Tofte[1*], Tommi Suvitaival[1*], Linda Ahonen[1], Signe A. Winther[1], Simone Theilade[1], Marie Frimodt-Møller[1], Tarunveer S. Ahluwalia[1*], Peter Rossing[1,2*]

[1]Steno Diabetes Center Copenhagen, Gentofte, Denmark, [2]University of Copenhagen, Copenhagen, Denmark. * *Equal author contributions*

Supplementary file including:

- Supplementary methods
- Supplementary Figure s1
- Supplementary report

Lipidomic analyses, PROFIL:

The serum samples were prepared according to the previously published Folch procedure[1] with minor modifications. Briefly, 10 µL of 0.9% NaCl, 92 µL of $CHCl_3$:MeOH (2:1, v/v) and 28 µL of a 10 µg mL^{-1} working standard solution of chosen lipid standards (for quality control and data normalization purposes) were added to 10 µL of each serum sample. The standard solution contained nine different lipid standards representing different lipid classes. The following compounds were purchased from Avanti Polar Lipids, Inc. (Alabaster, Alabama, USA): 1,2-diheptadecanoyl-*sn*-glycero-3-phosphoethanolamine (PE(17:0/17:0)), N-heptadecanoyl-D-*erythro*-sphingosylphosphorylcholine (SM(d18:1/17:0)), N-heptadecanoyl-D-*erythro*-sphingosine (Cer(d18:1/17:0)), 1,2-diheptadecanoyl-*sn*-glycero-3-phosphocholine (PC(17:0/17:0)), 1-heptadecanoyl-2-hydroxy-*sn*-glycero-3-phosphocholine (LPC(17:0)) and 1-palmitoyl-d31-2-oleoyl-*sn*-glycero-3-phosphocholine (PC(16:0/d31/18:1)). Additionally, 1,2-Dimyristoyl-*sn*-glycero-3-phospho(choline-d_{13}) (PC(14:0/d13)) was purchased from Sigma-Aldrich Denmark A/S (Copenhagen, Denmark) and Tripalmitin-1,1,1-13C3 (TG(16:0/16:0/16:0)-13C3) and Trioctanoin-1,1,1-13C3 (TG(8:0/8:0/8:0)-13C3) from Larodan (Solna, Sweden) for the same purpose. After the addition of the working standard solution of the chosen lipid standards, the samples were vortex mixed and allowed to stand on ice for 30 min. The samples were then centrifuged (9400 × *g*, 3 min, 4 °C), 60 µL from the lower layer of each sample was transferred to a glass vial with an insert and 60 µL of $CHCl_3$:MeOH (2:1, v/v) was added to each sample. The samples were then stored at -80 °C until analysis.

Calibration curves (at concentration levels of 100, 500, 1000, 1500, 2000 and 2500 ng mL^{-1}) for quantification of lipids were prepared using 1-hexadecyl-2-(9Z-octadecenoyl)-sn-glycero-3-phosphocholine (PC(16:0e/18:1(9Z))), 1-(1Z-octadecenyl)-2-(9Z-octadecenoyl)-sn-glycero-3-phosphocholine (PC(18:0p/18:1(9Z))), 1-octadecanoyl-sn-glycero-3-phosphocholine (LPC(18:0)), 1-(1Z-octadecenyl)-2-docosahexaenoyl-*sn*-glycero-3-phosphocholine (PC(18:0p/22:6)), 1-stearoyl-2-arachidonoyl-*sn*-glycero-3-phosphoinositol (PI(18:0/20:4)) and 1-stearoyl-2-linoleoyl-*sn*-glycerol (DG(18:0/18:2)) from Avanti Polar Lipids, Inc., 1-Palmitoyl-2-Hydroxy-sn-Glycero-3-Phosphatidylcholine (LPC(16:0)) from Larodan, and 1,2,3-Triheptadecanoylglycerol (TG(17:0/17:0/17:0)) and 3β-Hydroxy-5-cholestene 3-linoleate (ChoE(18:2)), 3β-Hydroxy-5-cholestene 3-oleate (ChoE(18:1(9Z))), 5-Cholesten-3β-yl octadecenoate (ChoE(18:0)) and 5-Cholestene 3-palmitate (ChoE(16:0)) from Sigma-Aldrich.

The samples were prepared and analysed using previously published methods.[1-3] and quality control was performed throughout the data set by including blanks, pure standard samples, extracted standard samples and control plasma samples. Relative standard deviations (%RSDs) for retention times and peak areas for lipid standards representing each lipid class in the serum samples and in the control plasma samples were calculated. The %RSDs for the retention times were on below 3.8% for both the serum samples and for the control plasma samples. The %RSDs for the normalized (to PC(16:0/d30/18:1)) peak areas were within accepted analytical limits below 15.4% and 16.8% for the serum samples and for the control plasma samples, respectively. This shows that the method is reliable and repeatable throughout the sample set.

References:

1. Folch, J., M. Lees, and G.H.S. Stanley, *A Simple Method for the Isolation and Purification of Total Lipides from Animal Tissues.* Journal of Biological Chemistry, 1957. **226**(1): p. 497-509.

2. O'Gorman, A., et al., *Identification of a plasma signature of psychotic disorder in children and adolescents from the Avon Longitudinal Study of Parents and Children (ALSPAC) cohort.* Translational Psychiatry, 2017. **7**: p. e1240.

3. Luukkonen, P.K., et al., *Impaired hepatic lipid synthesis from polyunsaturated fatty acids in TM6SF2 E167K variant carriers with NAFLD.* Journal of Hepatology. **67**(1): p. 128-136.

Supplementary Figure S1 Statistical analyses - flow chart

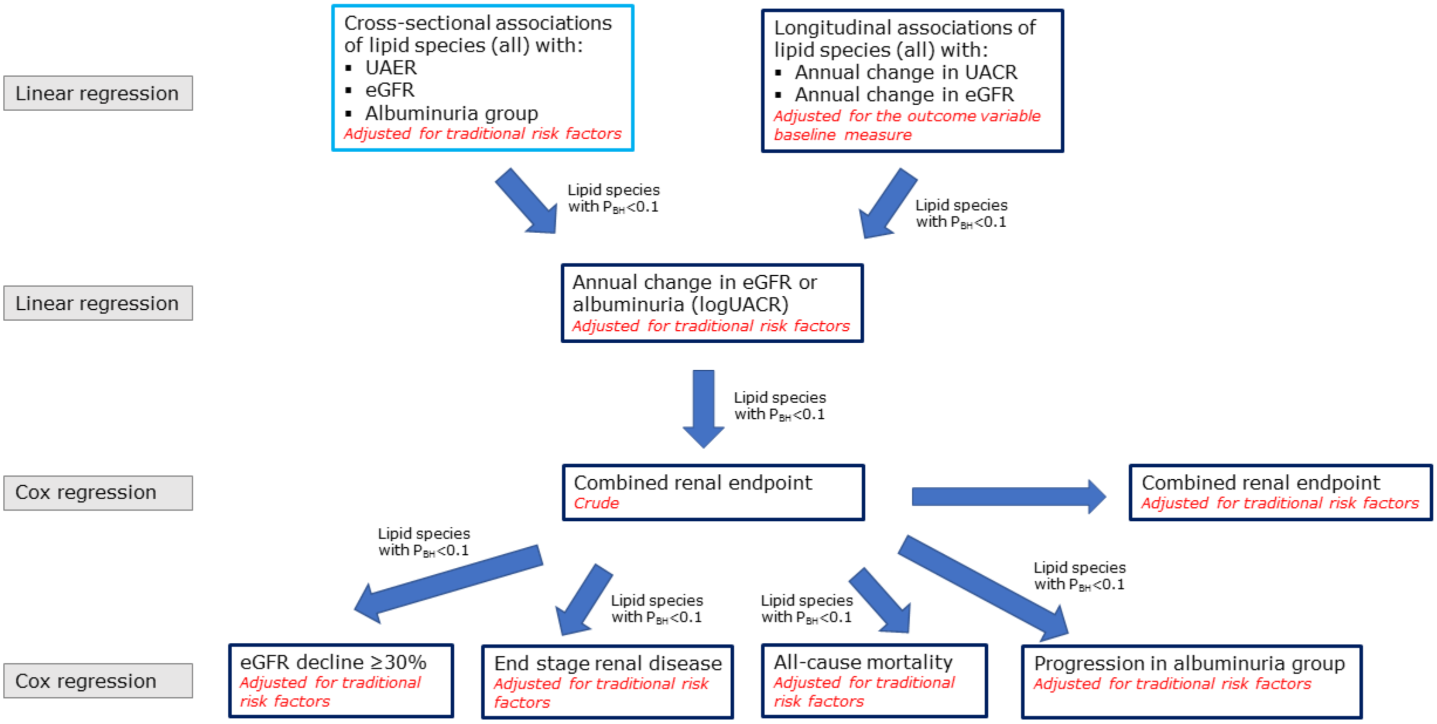

Flow chart illustrating how lipid species were tested for associations with kidney-related clinical variables and outcomes in a narrowing-down approach. P_{BH} corrected for multiple testing using the Benjamini-Hochberg method. UAER urinary albumin excretion rate, UACR urinary albumin to creatinine ratio, eGFR estimated glomerular filtration rate. Traditional risk factors included age, sex, HbA_{1c}, systolic blood pressure, smoking, body mass index, statin treatment, p-triglycerides, total p-cholesterol and, further, eGFR or UAER, where appropriate.

0033_PROFIL_2017: Statistical Analysis of the Lipidome in Relation to Renal Complications

Tommi Suvitaival,
tommi.raimo.leo.suvitaival@regionh.dk,
Steno Diabetes Center Copenhagen

August 12, 2019

Abstract

This document is Supplementary Material to the paper *Lower Serum Sphingomyelin and Phosphatidylcholine Levels Are Associated with Higher Risk of Progression to Diabetic Kidney Disease and All-Cause Mortality in Type 1 Diabetes* by Nete Tofte, Tommi Suvitaival, Linda Ahonen, Signe A. Winther, Simone Theilade, Tarun S. Ahluwalia, Marie Frimodt-M{o}ller and Peter Rossing.

Contents

1 Step 1: Lipidome-Wide Screening 4
1.1 Step 1A: Cross-Sectional Analysis of All Lipids . . . 4
1.1.1 Albuminuria Groups . . . 4
1.1.1.1 Crude Model . . . 4
1.1.1.1.1 Table of Model Coefficients . . . 5
1.1.1.1.2 Heatmaps of Model Coefficients . . . 12
1.1.1.2 Adjusted Model . . . 16
1.1.1.2.1 Table . . . 17
1.1.1.2.2 Heatmaps . . . 24
1.1.2 eGFR . . . 28
1.1.2.1 Crude Model . . . 28
1.1.2.1.1 Table . . . 29
1.1.2.1.2 Heatmaps . . . 34
1.1.2.2 Adjusted Model . . . 36
1.1.2.2.1 Table . . . 37
1.1.2.2.2 Heatmaps . . . 40
1.1.3 logUAER – Continuous Albuminuria . . . 41
1.1.3.1 Crude Model . . . 41
1.1.3.1.1 Table . . . 42
1.1.3.1.2 Heatmaps . . . 45
1.1.3.2 Adjusted Model . . . 47
1.1.3.2.1 Table . . . 48
1.1.3.2.2 Heatmaps . . . 51
1.1.4 Combined Heatmaps from Adjusted Cross-sectional Analyses . . . 52
1.2 Step 1B: Crude Associations of All Lipids to Clinical Changes Over Time . . . 54
1.2.1 eGFR Slope . . . 54
1.2.1.1 Table . . . 55
1.2.1.2 Heatmaps . . . 60
1.2.2 Albuminuria Slope . . . 62
1.2.2.1 Table . . . 63
1.2.2.2 Heatmaps . . . 68
1.3 Combined Heatmaps from Crude Models for Slopes . . . 70

2 Step 2: Adjusted Associations to Clinical Changes Over Time with Prioritized Lipids from Steps 1A-B 72
2.1 Step 2A: Adjusted Model for Prioritized Lipids from the Adjusted Cross-Sectional Models . . 72
2.1.1 Albuminuria Slope . . . 72
2.1.1.1 Table . . . 73
2.1.1.2 Heatmaps . . . 74
2.1.2 eGFR Slope . . . 75
2.1.2.1 Table . . . 76
2.1.2.2 Heatmaps . . . 77
2.2 Step 2B: Adjusted Model for Prioritized Lipids from the Crude Models for Slopes . . . 79
2.2.1 eGFR Slope . . . 79
2.2.1.1 Table . . . 80
2.2.1.2 Heatmaps . . . 81
2.2.2 Albuminuria Slope . . . 83
2.2.2.1 Table . . . 84
2.2.2.2 Heatmaps . . . 85

3 Step 3: Survival Analysis of Combined Renal Endpoint in Relation to Prioritized Lipids from Step 2A-B 87
3.1 Step 3A: Crude Model . . . 87
3.1.1 Table . . . 88
3.1.2 Heatmaps . . . 89
3.1.3 Forest Plot . . . 91
3.2 Step 3B: Adjusted Model . . . 92
3.2.1 Table . . . 93
3.2.2 Heatmaps . . . 94
3.2.3 Forest Plot . . . 96
3.3 Combined Forest Plot from Crude and Adjusted Models . . . 97

4 Step 4: Survival Analysis of Specific Renal Endpoints in Relation to Prioritized Lipids from Step 3A 98
4.1 Step 4A: Albuminuria Group Progression . . . 98
4.1.1 Crude Model . . . 98
4.1.1.1 Table . . . 99
4.1.1.2 Heatmaps . . . 100
4.1.1.3 Forest Plot . . . 102
4.1.2 Adjusted Model . . . 103
4.1.2.1 Table . . . 104
4.1.2.2 Heatmaps . . . 105
4.1.2.3 Forest Plot . . . 107
4.1.3 Combined Forest Plot from Crude and Adjusted Models . . . 108
4.2 Step 4B: All-Cause Mortality . . . 109
4.2.1 Crude Model . . . 109
4.2.1.1 Table . . . 110
4.2.1.2 Heatmaps . . . 111
4.2.1.3 Forest Plot . . . 113
4.2.2 Adjusted Model . . . 114
4.2.2.1 Table . . . 115
4.2.2.2 Heatmaps . . . 116
4.2.2.3 Forest Plot . . . 118
4.2.3 Combined Forest Plot . . . 119
4.3 Step 4C: eGFR Decline > 30 % . . . 120
4.3.1 Crude Model . . . 120
4.3.1.1 Table . . . 121

4.3.1.2 Heatmaps . . . 122
4.3.1.3 Forest Plot . . . 124
4.3.2 Adjusted Model . . . 125
4.3.2.1 Table . . . 126
4.3.2.2 Heatmaps . . . 126
4.3.2.3 Forest Plot . . . 128
4.3.3 Combined Forest Plot from Crude and Adjusted Models . . . 129
4.4 Step 4D: End-Stage Renal Disease . . . 130
4.4.1 Crude Model . . . 130
4.4.1.1 Table . . . 131
4.4.1.2 Heatmaps . . . 132
4.4.1.3 Forest Plot . . . 134
4.4.2 Adjusted Model . . . 135
4.4.2.1 Table . . . 136
4.4.2.2 Heatmaps . . . 136
4.4.2.3 Forest Plot . . . 138
4.4.3 Combined Forest Plot from Crude and Adjusted Models . . . 139
4.5 Compilation Forest Plot from Steps 4A-D . . . 140

5 Step 5: Detailed Assessment of the Top-Lipid in Relation to All-Cause Mortality 141
5.1 Step 5A: Analysis of Full Cohort . . . 141
5.1.1 Survival Model with Details . . . 141
5.1.1.1 Forest Plot with Clinical Variables . . . 142
5.1.1.2 Diagnostics of the Survival Model . . . 143
5.1.1.3 Kaplan-Maier Curve with Median Cutpoint . . . 145
5.1.2 Other Model Fits . . . 147
5.2 Step 5B: Analysis of an Age-and-Sex-Matched Subcohort . . . 149
5.2.1 Survival Model with Details . . . 152
5.2.1.1 Forest Plot with Clinical Variables . . . 153
5.2.1.2 Diagnostics of the Survival Model . . . 154
5.2.1.3 Kaplan-Maier Curve with Median Cutpoint . . . 156
5.2.2 Other Model Fits . . . 158

6 Correlation Matrix 160

7 Boxplots 162
7.1 Entire Cohort . . . 162
7.2 Age-and-Sex-Matched Subcohort . . . 167

8 Lipids' Proportion of the Total Amount of Lipid Class 173

9 Quality Control Table of Lipid Measurements 176

10 Appendix 179

1 Step 1: Lipidome-Wide Screening

1.1 Step 1A: Cross-Sectional Analysis of All Lipids

1.1.1 Albuminuria Groups

1.1.1.1 Crude Model

1.1.1.1.1 Table of Model Coefficients

```
##
##
## Table: Coefficients for  GroupT1D Micro
## Results of the crude model for albuminuria group variable.
## Shown in the table are name of the lipid (Name),
## model coefficient (Coefficient),
## its lower and upper confidence intervals (CI.L, CL.R),
## average value of the variable (AveExpr),
## p-value of the coefficient (P.Value) and
## the p-value after correction for multiple testing (adj.P.Val).
## The rows of the table are shown in an increasing order by the respective p-value.
##
## Name                   Coefficient      CI.L       CI.R   AveExpr    P.Value   adj.P.Val
## -------------------  ------------  --------  ---------  --------  ---------  ----------
## LPC(18:2)                -0.21600   -0.3290   -0.10300      24.0   0.000193      0.0205
## SM(d18:1/24:0)           -0.11100   -0.1900   -0.03330      23.6   0.005250      0.2690
## SM(d40:1)                -0.10100   -0.1750   -0.02680      24.0   0.007620      0.2690
## SM(d41:1)                -0.11300   -0.2000   -0.02590      22.7   0.011100      0.2930
## PC(O-36:3)               -0.12200   -0.2220   -0.02250      20.4   0.016400      0.3240
## PC(O-34:3)               -0.11100   -0.2040   -0.01680      21.9   0.020900      0.3240
## PC(O-36:2)               -0.10600   -0.1960   -0.01570      20.5   0.021400      0.3240
## LPC(18:0)                -0.07150   -0.1390   -0.00353      24.0   0.039200      0.5200
## PC(O-34:2)               -0.11200   -0.2280    0.00426      21.5   0.059000      0.6880
## PC(16:0e/18:1(9Z)        -0.07300   -0.1520    0.00622      21.8   0.070900      0.6880
## LPC(18:1)                -0.07860   -0.1650    0.00734      23.7   0.073000      0.6880
## PC(34:2)                  0.12400   -0.0139    0.26300      26.0   0.077900      0.6880
## LPC(16:0)                -0.03320   -0.0714    0.00498      25.3   0.088200      0.7190
## PC(O-38:5)               -0.06050   -0.1330    0.01200      22.7   0.102000      0.7530
## PC(37:2)                 -0.09810   -0.2170    0.02110      19.3   0.107000      0.7530
## SM(d38:1)                -0.05430   -0.1240    0.01540      23.1   0.127000      0.7740
## PC(O-38:4)               -0.07400   -0.1700    0.02170      21.7   0.129000      0.7740
## TG(18:1/18:1/18:1)       -0.09750   -0.2250    0.02980      24.8   0.133000      0.7740
## SM(d39:1)                -0.07570   -0.1770    0.02570      21.1   0.143000      0.7740
## TG(48:3)                  0.17700   -0.0646    0.41800      20.6   0.151000      0.7740
## PC(O-38:6)               -0.05660   -0.1350    0.02200      20.4   0.158000      0.7740
## TG(54:4)                 -0.08030   -0.1950    0.03450      21.4   0.170000      0.7740
## PC(O-36:4)               -0.05940   -0.1450    0.02580      22.9   0.171000      0.7740
## TG(45:0)                 -0.04070   -0.0996    0.01820      23.1   0.175000      0.7740
## PC(36:2)                 -0.03870   -0.0971    0.01970      26.1   0.194000      0.8230
## TG(54:5)                 -0.08890   -0.2300    0.05180      20.6   0.215000      0.8500
## TG(18:1/18:2/18:2)       -0.09800   -0.2630    0.06700      23.6   0.244000      0.8500
## TG(18:2/18:1/16:0)       -0.05250   -0.1430    0.03760      25.3   0.253000      0.8500
## SM(d38:2)                -0.04320   -0.1180    0.03160      21.3   0.257000      0.8500
## PC(O-36:5)               -0.04430   -0.1250    0.03620      22.3   0.280000      0.8500
## TG(18:2/18:1/18:1)       -0.07270   -0.2070    0.06130      24.4   0.287000      0.8500
## TG(18:0/18:1/20:4)        0.10400   -0.0883    0.29600      21.3   0.289000      0.8500
## TG(50:2)                  0.06080   -0.0517    0.17300      21.2   0.289000      0.8500
## TG(47:1)                 -0.07790   -0.2230    0.06750      19.6   0.293000      0.8500
## TG(54:6)                  0.10000   -0.0874    0.28700      19.5   0.295000      0.8500
## TG(18:1/12:0/18:1)        0.09550   -0.0836    0.27500      22.5   0.296000      0.8500
## TG(46:1)                  0.09600   -0.0845    0.27600      21.3   0.297000      0.8500
## SM(d36:1)                -0.03080   -0.0921    0.03040      23.4   0.323000      0.8760
```

```
## TG(14:0/18:1/18:1)         0.06810   -0.0683   0.20500      24.4  0.327000       0.8760
## TG(18:1/18:1/16:0)        -0.04900   -0.1510   0.05280      25.6  0.345000       0.8760
## PC(38:6)                  -0.03460   -0.1080   0.03900      25.3  0.357000       0.8760
## TG(46:2)                   0.08830   -0.1060   0.28200      20.1  0.372000       0.8760
## TG(18:2/18:2/18:2)         0.08330   -0.1000   0.26600      22.1  0.373000       0.8760
## LPC(20:4)                 -0.04990   -0.1610   0.06120      21.4  0.379000       0.8760
## PC(38:5)                  -0.02340   -0.0755   0.02880      24.5  0.379000       0.8760
## TG(54:3)                  -0.04420   -0.1440   0.05510      21.7  0.383000       0.8760
## SM(d41:2)                 -0.03550   -0.1170   0.04630      22.5  0.394000       0.8760
## SM(d32:1)                  0.03230   -0.0432   0.10800      22.3  0.401000       0.8760
## PC(36:4)                  -0.01950   -0.0653   0.02640      26.0  0.405000       0.8760
## PC(35:2)                  -0.03880   -0.1350   0.05710      21.8  0.427000       0.9050
## PC(34:1)                   0.02720   -0.0412   0.09560      25.9  0.436000       0.9050
## PC(40:7)                  -0.04130   -0.1490   0.06640      21.4  0.451000       0.9200
## SM(d33:1)                 -0.02810   -0.1060   0.04970      21.3  0.479000       0.9340
## PC(34:3)                  -0.05330   -0.2030   0.09630      22.5  0.484000       0.9340
## TG(18:2/22:5/16:0)         0.08070   -0.1490   0.31000      22.1  0.490000       0.9340
## PC(38:2)                  -0.02810   -0.1090   0.05250      21.3  0.493000       0.9340
## TG(14:0/16:0/18:1)         0.06120   -0.1210   0.24300      22.8  0.509000       0.9350
## SM(d40:2)                 -0.02120   -0.0868   0.04440      23.7  0.527000       0.9350
## TG(56:6)                   0.03590   -0.0785   0.15000      19.9  0.538000       0.9350
## TG(16:0/18:2/18:3)        -0.07270   -0.3060   0.16000      22.0  0.541000       0.9350
## PC(35:1)                  -0.02740   -0.1170   0.06240      21.8  0.549000       0.9350
## PC(36:5)                   0.04910   -0.1200   0.21800      24.3  0.569000       0.9350
## TG(56:4)                  -0.05560   -0.2470   0.13600      20.0  0.569000       0.9350
## TG(52:3)                  -0.02200   -0.1010   0.05680      22.3  0.584000       0.9350
## TG(16:0/18:2/18:2)        -0.04320   -0.2010   0.11500      24.5  0.591000       0.9350
## PC(40:6)                  -0.02820   -0.1390   0.08290      23.9  0.619000       0.9350
## TG(50:1)                   0.04450   -0.1320   0.22100      24.3  0.621000       0.9350
## SM(d34:1)                  0.00817   -0.0258   0.04210      24.9  0.637000       0.9350
## PC(32:1)                   0.03550   -0.1130   0.18400      23.3  0.638000       0.9350
## TG(53:4)                  -0.04010   -0.2210   0.14100      19.7  0.663000       0.9350
## TG(50:3)                   0.03260   -0.1140   0.17900      23.5  0.664000       0.9350
## PC(32:2)                  -0.02900   -0.1630   0.10500      20.8  0.671000       0.9350
## TG(56:7)                   0.02750   -0.1020   0.15700      19.6  0.677000       0.9350
## PC(36:3)                  -0.01720   -0.0983   0.06400      25.6  0.678000       0.9350
## SM(42:2)                  -0.01200   -0.0710   0.04690      25.2  0.688000       0.9350
## PC(40:5)                  -0.01920   -0.1180   0.07950      22.0  0.703000       0.9350
## PC(32:0)                  -0.01000   -0.0619   0.04180      22.5  0.704000       0.9350
## TG(58:9)                   0.05100   -0.2160   0.31800      19.9  0.708000       0.9350
## PC(33:1)                  -0.01950   -0.1240   0.08520      20.9  0.715000       0.9350
## TG(46:0)                   0.03130   -0.1410   0.20300      20.4  0.721000       0.9350
## TG(52:4)                  -0.02130   -0.1390   0.09660      21.7  0.723000       0.9350
## TG(49:3)                  -0.00361   -0.0239   0.01670      20.7  0.727000       0.9350
## PC(38:3)                  -0.01550   -0.1040   0.07310      24.0  0.732000       0.9350
## TG(53:3)                  -0.02780   -0.1940   0.13800      20.7  0.742000       0.9360
## TG(54:2)                  -0.02610   -0.1910   0.13900      23.4  0.757000       0.9370
## SM(d16:1/18:1)            -0.01070   -0.0798   0.05830      22.8  0.760000       0.9370
## TG(49:2)                   0.01520   -0.1080   0.13900      20.4  0.809000       0.9710
## TG(18:1/18:1/22:6)         0.02830   -0.2420   0.29900      20.7  0.837000       0.9710
## SM(d18:2/24:1)             0.00707   -0.0609   0.07510      24.0  0.838000       0.9710
## TG(16:0/18:2/22:6)         0.02900   -0.2700   0.32800      21.0  0.849000       0.9710
## TG(56:5)                  -0.01350   -0.1590   0.13200      20.3  0.856000       0.9710
## TG(51:2)                   0.01390   -0.1460   0.17400      21.6  0.865000       0.9710
```

```
## TG(14:0/18:2/18:2)        0.01550   -0.1960    0.22700      21.2   0.885000      0.9710
## TG(56:3)                  0.01110   -0.1550    0.17700      19.9   0.896000      0.9710
## TG(49:1)                  0.00811   -0.1300    0.14600      20.9   0.908000      0.9710
## SM(d36:2)                -0.00347   -0.0695    0.06250      21.4   0.918000      0.9710
## TG(52:5)                 -0.00992   -0.2010    0.18100      19.8   0.919000      0.9710
## TG(50:0)                 -0.00995   -0.2160    0.19600      20.8   0.924000      0.9710
## TG(53:2)                 -0.00738   -0.1850    0.17000      20.7   0.935000      0.9710
## LPC(16:1)                -0.00448   -0.1140    0.10500      20.0   0.936000      0.9710
## TG(51:1)                 -0.00610   -0.1800    0.16800      20.3   0.945000      0.9710
## TG(16:0/18:0/18:1)       -0.00714   -0.2310    0.21700      23.1   0.950000      0.9710
## TG(51:3)                 -0.00517   -0.1690    0.15900      20.7   0.951000      0.9710
## TG(16:0/22:5/18:1)        0.00424   -0.1360    0.14500      22.7   0.953000      0.9710
## PC(38:4)                 -0.00123   -0.0575    0.05500      25.2   0.966000      0.9720
## TG(52:2)                 -0.00133   -0.0757    0.07310      22.6   0.972000      0.9720
##
##
## Table: Coefficients for  GroupT1D Macro
## Results of the crude model for albuminuria group variable.
## Shown in the table are name of the lipid (Name),
## model coefficient (Coefficient),
## its lower and upper confidence intervals (CI.L, CL.R),
## average value of the variable (AveExpr),
## p-value of the coefficient (P.Value) and
## the p-value after correction for multiple testing (adj.P.Val).
## The rows of the table are shown in an increasing order by the respective p-value.
##
## Name                   Coefficient        CI.L        CI.R   AveExpr    P.Value   adj.P.Val
## -------------------  -------------  ----------  ----------  --------  ---------  ----------
## PC(O-34:3)                -0.26200    -0.35200    -0.17300      21.9   0.00e+00    1.50e-06
## SM(d18:1/24:0)            -0.18100    -0.25600    -0.10600      23.6   2.50e-06    1.33e-04
## SM(d40:1)                 -0.16100    -0.23100    -0.08980      24.0   9.70e-06    3.44e-04
## PC(O-36:3)                -0.21200    -0.30700    -0.11600      20.4   1.47e-05    3.89e-04
## SM(d41:1)                 -0.17200    -0.25500    -0.08820      22.7   5.85e-05    1.24e-03
## PC(O-36:2)                -0.17500    -0.26100    -0.08890      20.5   7.44e-05    1.31e-03
## PC(16:0e/18:1(9Z)         -0.13200    -0.20800    -0.05650      21.8   6.47e-04    9.80e-03
## PC(34:2)                   0.22000     0.08720     0.35200      26.0   1.18e-03    1.44e-02
## PC(O-38:6)                -0.12400    -0.19900    -0.04910      20.4   1.22e-03    1.44e-02
## TG(45:0)                  -0.08900    -0.14500    -0.03270      23.1   2.00e-03    2.12e-02
## TG(56:3)                   0.23600     0.07690     0.39500      19.9   3.69e-03    3.55e-02
## PC(32:2)                  -0.18800    -0.31600    -0.05960      20.8   4.13e-03    3.65e-02
## PC(O-34:2)                -0.15700    -0.26800    -0.04580      21.5   5.73e-03    4.67e-02
## TG(18:0/18:1/20:4)         0.24400     0.06010     0.42800      21.3   9.39e-03    6.78e-02
## SM(d39:1)                 -0.12800    -0.22500    -0.03100      21.1   9.77e-03    6.78e-02
## PC(37:2)                  -0.14900    -0.26300    -0.03460      19.3   1.07e-02    6.78e-02
## LPC(18:2)                 -0.14000    -0.24800    -0.03170      24.0   1.14e-02    6.78e-02
## SM(d38:1)                 -0.08610    -0.15300    -0.01940      23.1   1.15e-02    6.78e-02
## TG(56:4)                   0.23100     0.04720     0.41400      20.0   1.38e-02    7.25e-02
## PC(32:0)                  -0.06220    -0.11200    -0.01260      22.5   1.41e-02    7.25e-02
## PC(38:6)                  -0.08800    -0.15800    -0.01760      25.3   1.44e-02    7.25e-02
## PC(O-36:5)                -0.09540    -0.17200    -0.01840      22.3   1.52e-02    7.31e-02
## TG(53:3)                   0.18700     0.02810     0.34500      20.7   2.11e-02    9.73e-02
## SM(d40:2)                 -0.07230    -0.13500    -0.00956      23.7   2.40e-02    1.06e-01
## TG(53:2)                   0.18600     0.01640     0.35600      20.7   3.17e-02    1.34e-01
## PC(36:5)                  -0.17300    -0.33500    -0.01100      24.3   3.64e-02    1.48e-01
```

```
## PC(38:4)               0.05710   0.00328   0.11100       25.2  3.76e-02    1.48e-01
## TG(54:3)               0.09910   0.00411   0.19400       21.7  4.09e-02    1.51e-01
## PC(35:2)              -0.09520  -0.18700  -0.00341       21.8  4.21e-02    1.51e-01
## SM(d41:2)             -0.08100  -0.15900  -0.00268       22.5  4.27e-02    1.51e-01
## TG(51:2)               0.15700   0.00374   0.31000       21.6  4.47e-02    1.53e-01
## PC(40:6)              -0.10200  -0.20800   0.00467       23.9  6.09e-02    1.96e-01
## TG(52:2)               0.06750  -0.00366   0.13900       22.6  6.30e-02    1.96e-01
## TG(52:4)               0.10700  -0.00582   0.22000       21.7  6.30e-02    1.96e-01
## PC(38:2)              -0.07250  -0.15000   0.00461       21.3  6.53e-02    1.97e-01
## TG(16:0/18:2/18:3)     0.20900  -0.01450   0.43200       22.0  6.68e-02    1.97e-01
## TG(52:5)               0.16800  -0.01430   0.35100       19.8  7.08e-02    2.03e-01
## TG(56:6)               0.10000  -0.00936   0.21000       19.9  7.30e-02    2.04e-01
## TG(53:4)               0.15600  -0.01700   0.32900       19.7  7.71e-02    2.10e-01
## PC(33:1)              -0.08850  -0.18900   0.01160       20.9  8.30e-02    2.15e-01
## PC(40:7)              -0.09110  -0.19400   0.01200       21.4  8.31e-02    2.15e-01
## PC(O-38:4)            -0.07950  -0.17100   0.01210       21.7  8.87e-02    2.21e-01
## SM(d38:2)             -0.06150  -0.13300   0.01000       21.3  9.17e-02    2.21e-01
## TG(16:0/22:5/18:1)     0.11500  -0.01910   0.25000       22.7  9.24e-02    2.21e-01
## TG(56:5)               0.11900  -0.02020   0.25900       20.3  9.36e-02    2.21e-01
## TG(51:3)               0.13200  -0.02500   0.28900       20.7  9.93e-02    2.29e-01
## TG(54:2)               0.12800  -0.02990   0.28600       23.4  1.12e-01    2.52e-01
## TG(54:6)               0.14300  -0.03630   0.32200       19.5  1.18e-01    2.57e-01
## TG(16:0/18:2/18:2)     0.12000  -0.03100   0.27100       24.5  1.19e-01    2.57e-01
## TG(14:0/18:1/18:1)     0.10200  -0.02800   0.23300       24.4  1.24e-01    2.57e-01
## TG(50:2)               0.08450  -0.02320   0.19200       21.2  1.24e-01    2.57e-01
## TG(18:2/18:2/18:2)     0.13100  -0.04420   0.30600       22.1  1.43e-01    2.91e-01
## TG(18:1/18:1/18:1)     0.09030  -0.03150   0.21200       24.8  1.46e-01    2.92e-01
## TG(52:3)               0.05480  -0.02060   0.13000       22.3  1.54e-01    2.98e-01
## TG(50:3)               0.10200  -0.03860   0.24300       23.5  1.55e-01    2.98e-01
## PC(34:3)              -0.10100  -0.24400   0.04270       22.5  1.69e-01    3.19e-01
## TG(18:2/18:1/18:1)     0.08940  -0.03890   0.21800       24.4  1.72e-01    3.19e-01
## TG(54:4)               0.07300  -0.03680   0.18300       21.4  1.92e-01    3.49e-01
## PC(O-38:5)            -0.04560  -0.11500   0.02380       22.7  1.97e-01    3.49e-01
## PC(36:3)              -0.05100  -0.12900   0.02670       25.6  1.98e-01    3.49e-01
## SM(42:2)              -0.03490  -0.09130   0.02140       25.2  2.24e-01    3.78e-01
## PC(38:5)              -0.03090  -0.08080   0.01900       24.5  2.24e-01    3.78e-01
## PC(40:5)              -0.05820  -0.15300   0.03610       22.0  2.26e-01    3.78e-01
## TG(54:5)               0.08220  -0.05230   0.21700       20.6  2.31e-01    3.78e-01
## LPC(20:4)              0.06480  -0.04150   0.17100       21.4  2.32e-01    3.78e-01
## PC(O-36:4)            -0.04880  -0.13000   0.03270       22.9  2.40e-01    3.83e-01
## SM(d34:1)              0.01940  -0.01310   0.05190       24.9  2.42e-01    3.83e-01
## PC(32:1)              -0.08240  -0.22400   0.05950       23.3  2.55e-01    3.94e-01
## LPC(16:0)             -0.02100  -0.05760   0.01550       25.3  2.59e-01    3.94e-01
## TG(50:1)               0.09700  -0.07200   0.26600       24.3  2.60e-01    3.94e-01
## TG(14:0/18:2/18:2)     0.11400  -0.08760   0.31600       21.2  2.67e-01    3.98e-01
## TG(56:7)               0.06900  -0.05510   0.19300       19.6  2.75e-01    4.05e-01
## PC(35:1)              -0.04720  -0.13300   0.03860       21.8  2.81e-01    4.05e-01
## TG(18:1/18:2/18:2)     0.08630  -0.07140   0.24400       23.6  2.83e-01    4.05e-01
## SM(d33:1)             -0.03980  -0.11400   0.03460       21.3  2.94e-01    4.16e-01
## TG(51:1)               0.08310  -0.08300   0.24900       20.3  3.26e-01    4.55e-01
## LPC(18:0)             -0.03110  -0.09610   0.03380       24.0  3.47e-01    4.77e-01
## TG(48:3)               0.10900  -0.12200   0.34000       20.6  3.53e-01    4.77e-01
## TG(16:0/18:0/18:1)     0.10100  -0.11300   0.31500       23.1  3.56e-01    4.77e-01
## PC(36:4)               0.01880  -0.02500   0.06270       26.0  4.00e-01    5.30e-01
```

```
## TG(18:1/18:1/16:0)        0.04080   -0.05660    0.13800      25.6   4.11e-01    5.33e-01
## SM(d16:1/18:1)           -0.02750   -0.09350    0.03860      22.8   4.14e-01    5.33e-01
## SM(d36:1)                -0.02420   -0.08280    0.03440      23.4   4.17e-01    5.33e-01
## LPC(18:1)                -0.03350   -0.11600    0.04870      23.7   4.24e-01    5.35e-01
## TG(18:1/12:0/18:1)        0.06740   -0.10400    0.23900      22.5   4.40e-01    5.49e-01
## TG(49:3)                 -0.00742   -0.02680    0.01200      20.7   4.52e-01    5.58e-01
## SM(d36:2)                 0.02310   -0.04000    0.08620      21.4   4.73e-01    5.76e-01
## TG(49:2)                  0.04040   -0.07760    0.15800      20.4   5.02e-01    6.05e-01
## PC(36:2)                 -0.01740   -0.07330    0.03850      26.1   5.41e-01    6.41e-01
## TG(49:1)                  0.04000   -0.09170    0.17200      20.9   5.51e-01    6.41e-01
## TG(14:0/16:0/18:1)        0.05240   -0.12200    0.22700      22.8   5.55e-01    6.41e-01
## TG(47:1)                 -0.04140   -0.18000    0.09770      19.6   5.59e-01    6.41e-01
## TG(18:2/22:5/16:0)        0.06480   -0.15500    0.28400      22.1   5.62e-01    6.41e-01
## SM(d32:1)                 0.01700   -0.05520    0.08920      22.3   6.44e-01    7.24e-01
## TG(18:1/18:1/22:6)        0.06000   -0.19900    0.31900      20.7   6.49e-01    7.24e-01
## SM(d18:2/24:1)           -0.01470   -0.07970    0.05040      24.0   6.58e-01    7.27e-01
## TG(46:2)                  0.03090   -0.15500    0.21700      20.1   7.44e-01    8.05e-01
## TG(16:0/18:2/22:6)       -0.04760   -0.33400    0.23900      21.0   7.44e-01    8.05e-01
## TG(46:0)                 -0.02080   -0.18500    0.14400      20.4   8.04e-01    8.47e-01
## TG(18:2/18:1/16:0)        0.01080   -0.07540    0.09690      25.3   8.06e-01    8.47e-01
## PC(34:1)                  0.00815   -0.05730    0.07360      25.9   8.07e-01    8.47e-01
## TG(58:9)                  0.02810   -0.22700    0.28300      19.9   8.29e-01    8.61e-01
## LPC(16:1)                -0.00809   -0.11200    0.09630      20.0   8.79e-01    9.05e-01
## TG(50:0)                  0.01180   -0.18500    0.20900      20.8   9.06e-01    9.24e-01
## TG(46:1)                  0.00909   -0.16400    0.18200      21.3   9.18e-01    9.26e-01
## PC(38:3)                  0.00258   -0.08210    0.08730      24.0   9.52e-01    9.52e-01
##
##
## Table: Coefficients for  GroupHealthy Control
## Results of the crude model for albuminuria group variable.
## Shown in the table are name of the lipid (Name),
## model coefficient (Coefficient),
## its lower and upper confidence intervals (CI.L, CL.R),
## average value of the variable (AveExpr),
## p-value of the coefficient (P.Value) and
## the p-value after correction for multiple testing (adj.P.Val).
## The rows of the table are shown in an increasing order by the respective p-value.
##
## Name                   Coefficient       CI.L       CI.R   AveExpr    P.Value   adj.P.Val
## -------------------  ------------  ---------  ---------  --------  ---------  ----------
## PC(38:3)                  4.11e-01    0.26900    0.55200      24.0   0.00e+00    1.80e-06
## LPC(18:1)                -3.29e-01   -0.46600   -0.19200      23.7   2.90e-06    1.52e-04
## LPC(18:2)                -3.88e-01   -0.56900   -0.20800      24.0   2.67e-05    9.42e-04
## PC(O-36:2)               -2.53e-01   -0.39700   -0.10900      20.5   5.79e-04    1.50e-02
## TG(14:0/18:2/18:2)        5.79e-01    0.24300    0.91500      21.2   7.68e-04    1.50e-02
## PC(O-36:3)               -2.70e-01   -0.42900   -0.11100      20.4   8.76e-04    1.50e-02
## TG(50:3)                  3.94e-01    0.16000    0.62800      23.5   9.93e-04    1.50e-02
## PC(16:0e/18:1(9Z)        -2.03e-01   -0.32900   -0.07650      21.8   1.68e-03    2.23e-02
## PC(O-38:5)               -1.80e-01   -0.29500   -0.06400      22.7   2.36e-03    2.78e-02
## TG(16:0/18:2/18:2)        3.74e-01    0.12300    0.62600      24.5   3.60e-03    3.48e-02
## PC(36:2)                 -1.39e-01   -0.23200   -0.04540      26.1   3.61e-03    3.48e-02
## TG(51:3)                  3.76e-01    0.11500    0.63700      20.7   4.83e-03    4.27e-02
## TG(16:0/18:2/18:3)        5.11e-01    0.13900    0.88200      22.0   7.13e-03    5.82e-02
## TG(52:5)                  4.11e-01    0.10700    0.71500      19.8   8.21e-03    5.98e-02
```

```
## PC(32:2)               2.83e-01   0.06980   0.49600     20.8  9.35e-03   5.98e-02
## TG(18:1/12:0/18:1)     3.78e-01   0.09280   0.66300     22.5  9.47e-03   5.98e-02
## TG(48:3)               5.09e-01   0.12400   0.89300     20.6  9.60e-03   5.98e-02
## TG(14:0/18:1/18:1)     2.80e-01   0.06250   0.49700     24.4  1.17e-02   6.88e-02
## TG(52:4)               2.35e-01   0.04680   0.42200     21.7  1.44e-02   7.65e-02
## TG(51:2)               3.18e-01   0.06350   0.57300     21.6  1.44e-02   7.65e-02
## TG(53:4)               3.51e-01   0.06280   0.63900     19.7  1.71e-02   8.39e-02
## TG(18:2/18:2/18:2)     3.54e-01   0.06250   0.64600     22.1  1.74e-02   8.39e-02
## TG(45:0)              -1.10e-01  -0.20400  -0.01630     23.1  2.15e-02   9.91e-02
## TG(50:2)               2.05e-01   0.02540   0.38400     21.2  2.53e-02   1.03e-01
## TG(16:0/18:2/22:6)     5.43e-01   0.06650   1.02000     21.0  2.56e-02   1.03e-01
## PC(O-38:6)            -1.42e-01  -0.26700  -0.01710     20.4  2.59e-02   1.03e-01
## PC(40:6)               2.01e-01   0.02390   0.37800     23.9  2.62e-02   1.03e-01
## TG(49:2)               2.21e-01   0.02470   0.41800     20.4  2.74e-02   1.04e-01
## TG(18:2/18:1/16:0)     1.59e-01   0.01580   0.30300     25.3  2.97e-02   1.08e-01
## TG(14:0/16:0/18:1)     3.17e-01   0.02680   0.60700     22.8  3.23e-02   1.14e-01
## TG(18:2/22:5/16:0)     3.89e-01   0.02330   0.75400     22.1  3.71e-02   1.27e-01
## TG(52:3)               1.31e-01   0.00522   0.25700     22.3  4.12e-02   1.37e-01
## TG(54:6)               2.92e-01  -0.00639   0.59100     19.5  5.51e-02   1.77e-01
## PC(36:3)               1.23e-01  -0.00588   0.25300     25.6  6.13e-02   1.91e-01
## SM(d41:1)              1.31e-01  -0.00755   0.27000     22.7  6.38e-02   1.92e-01
## PC(38:5)              -7.79e-02  -0.16100   0.00524     24.5  6.62e-02   1.92e-01
## TG(53:3)               2.47e-01  -0.01770   0.51100     20.7  6.74e-02   1.92e-01
## PC(32:0)              -7.62e-02  -0.15900   0.00640     22.5  7.05e-02   1.92e-01
## SM(d39:1)              1.49e-01  -0.01270   0.31000     21.1  7.08e-02   1.92e-01
## SM(d16:1/18:1)        -9.50e-02  -0.20500   0.01500     22.8  9.03e-02   2.39e-01
## TG(53:2)               2.41e-01  -0.04250   0.52400     20.7  9.57e-02   2.47e-01
## TG(49:3)              -2.70e-02  -0.05930   0.00527     20.7  1.01e-01   2.49e-01
## PC(O-34:3)            -1.25e-01  -0.27400   0.02450     21.9  1.01e-01   2.49e-01
## TG(52:2)               9.71e-02  -0.02140   0.21600     22.6  1.08e-01   2.60e-01
## PC(38:4)               7.16e-02  -0.01810   0.16100     25.2  1.17e-01   2.76e-01
## TG(46:2)               2.43e-01  -0.06670   0.55200     20.1  1.24e-01   2.85e-01
## TG(50:1)               2.20e-01  -0.06200   0.50100     24.3  1.26e-01   2.85e-01
## TG(51:1)               2.12e-01  -0.06430   0.48900     20.3  1.32e-01   2.92e-01
## TG(56:7)               1.49e-01  -0.05710   0.35600     19.6  1.56e-01   3.37e-01
## TG(46:1)               1.98e-01  -0.08910   0.48600     21.3  1.76e-01   3.67e-01
## TG(49:1)               1.51e-01  -0.06860   0.37000     20.9  1.78e-01   3.67e-01
## LPC(20:4)             -1.21e-01  -0.29800   0.05650     21.4  1.82e-01   3.67e-01
## PC(O-36:4)            -9.20e-02  -0.22800   0.04370     22.9  1.84e-01   3.67e-01
## PC(40:7)              -1.07e-01  -0.27900   0.06450     21.4  2.21e-01   4.33e-01
## PC(34:2)               1.36e-01  -0.08430   0.35700     26.0  2.26e-01   4.35e-01
## PC(36:4)               4.29e-02  -0.03020   0.11600     26.0  2.49e-01   4.72e-01
## TG(56:5)               1.33e-01  -0.09940   0.36500     20.3  2.62e-01   4.81e-01
## TG(16:0/22:5/18:1)     1.28e-01  -0.09640   0.35200     22.7  2.63e-01   4.81e-01
## TG(54:3)              -8.85e-02  -0.24700   0.06980     21.7  2.73e-01   4.85e-01
## TG(18:1/18:2/18:2)     1.46e-01  -0.11600   0.40900     23.6  2.75e-01   4.85e-01
## SM(d38:1)              6.00e-02  -0.05110   0.17100     23.1  2.89e-01   4.96e-01
## PC(O-34:2)            -9.99e-02  -0.28500   0.08530     21.5  2.90e-01   4.96e-01
## TG(18:1/18:1/16:0)     8.64e-02  -0.07580   0.24900     25.6  2.96e-01   4.96e-01
## SM(42:2)              -4.96e-02  -0.14400   0.04430     25.2  3.00e-01   4.96e-01
## SM(d40:1)              6.08e-02  -0.05710   0.17900     24.0  3.12e-01   5.08e-01
## TG(56:3)              -1.31e-01  -0.39500   0.13400     19.9  3.33e-01   5.35e-01
## PC(38:6)               5.72e-02  -0.06000   0.17400     25.3  3.38e-01   5.35e-01
## PC(O-38:4)            -7.19e-02  -0.22400   0.08070     21.7  3.55e-01   5.48e-01
```

```
## PC(40:5)                7.38e-02   -0.08340    0.23100       22.0   3.57e-01    5.48e-01
## SM(d34:1)              -2.47e-02   -0.07890    0.02940       24.9   3.70e-01    5.50e-01
## PC(35:1)               -6.40e-02   -0.20700    0.07900       21.8   3.80e-01    5.50e-01
## SM(d18:1/24:0)          5.57e-02   -0.06890    0.18000       23.6   3.81e-01    5.50e-01
## SM(d41:2)               5.80e-02   -0.07250    0.18800       22.5   3.83e-01    5.50e-01
## SM(d18:2/24:1)         -4.81e-02   -0.15600    0.06030       24.0   3.84e-01    5.50e-01
## TG(56:4)               -1.33e-01   -0.43900    0.17200       20.0   3.91e-01    5.51e-01
## TG(18:0/18:1/20:4)     -1.32e-01   -0.43800    0.17400       21.3   3.98e-01    5.51e-01
## PC(37:2)               -8.13e-02   -0.27100    0.10900       19.3   4.01e-01    5.51e-01
## PC(O-36:5)             -5.39e-02   -0.18200    0.07420       22.3   4.09e-01    5.56e-01
## PC(32:1)                9.43e-02   -0.14200    0.33100       23.3   4.34e-01    5.78e-01
## TG(18:1/18:1/18:1)     -8.05e-02   -0.28300    0.12200       24.8   4.36e-01    5.78e-01
## TG(16:0/18:0/18:1)      1.33e-01   -0.22300    0.49000       23.1   4.63e-01    6.00e-01
## PC(35:2)               -5.70e-02   -0.21000    0.09590       21.8   4.65e-01    6.00e-01
## TG(58:9)                1.46e-01   -0.27900    0.57100       19.9   5.00e-01    6.39e-01
## SM(d36:2)               3.29e-02   -0.07230    0.13800       21.4   5.39e-01    6.80e-01
## LPC(18:0)               2.98e-02   -0.07840    0.13800       24.0   5.89e-01    7.34e-01
## TG(47:1)                6.07e-02   -0.17100    0.29200       19.6   6.07e-01    7.48e-01
## SM(d33:1)              -3.19e-02   -0.15600    0.09210       21.3   6.14e-01    7.48e-01
## TG(54:4)               -3.87e-02   -0.22200    0.14400       21.4   6.78e-01    8.16e-01
## TG(18:1/18:1/22:6)      8.04e-02   -0.35100    0.51200       20.7   7.14e-01    8.44e-01
## PC(36:5)               -4.99e-02   -0.32000    0.22000       24.3   7.16e-01    8.44e-01
## LPC(16:1)               2.63e-02   -0.14800    0.20000       20.0   7.67e-01    8.74e-01
## PC(38:2)                1.94e-02   -0.10900    0.14800       21.3   7.67e-01    8.74e-01
## TG(18:2/18:1/18:1)      3.20e-02   -0.18200    0.24600       24.4   7.68e-01    8.74e-01
## PC(34:1)               -1.58e-02   -0.12500    0.09320       25.9   7.76e-01    8.74e-01
## SM(d40:2)               1.46e-02   -0.08990    0.11900       23.7   7.83e-01    8.74e-01
## SM(d32:1)              -1.61e-02   -0.13600    0.10400       22.3   7.92e-01    8.75e-01
## TG(50:0)                4.06e-02   -0.28700    0.36800       20.8   8.08e-01    8.75e-01
## SM(d36:1)               1.20e-02   -0.08560    0.11000       23.4   8.09e-01    8.75e-01
## PC(33:1)                1.89e-02   -0.14800    0.18600       20.9   8.24e-01    8.83e-01
## TG(54:5)                1.91e-02   -0.20500    0.24300       20.6   8.67e-01    9.11e-01
## TG(54:2)               -2.24e-02   -0.28600    0.24100       23.4   8.68e-01    9.11e-01
## TG(46:0)                1.61e-02   -0.25800    0.29000       20.4   9.08e-01    9.44e-01
## TG(56:6)               -8.82e-03   -0.19100    0.17300       19.9   9.24e-01    9.51e-01
## SM(d38:2)               4.36e-03   -0.11500    0.12300       21.3   9.43e-01    9.61e-01
## PC(34:3)               -6.02e-03   -0.24500    0.23200       22.5   9.60e-01    9.70e-01
## LPC(16:0)               8.15e-05   -0.06080    0.06100       25.3   9.98e-01    9.98e-01
```

1.1.1.1.2 Heatmaps of Model Coefficients

```
## Loading required package: stringr

##   Label Relation p.adj
## 1     #  p.adj <  0.01
## 2     x  p.adj <  0.05
## 3     *  p.adj <  0.10

##   Label Relation p.adj
## 1     #  p.adj <  0.01
## 2     x  p.adj <  0.05
## 3     *  p.adj <  0.10

##   Label Relation p.adj
## 1     #  p.adj <  0.01
## 2     x  p.adj <  0.05
## 3     *  p.adj <  0.10
```

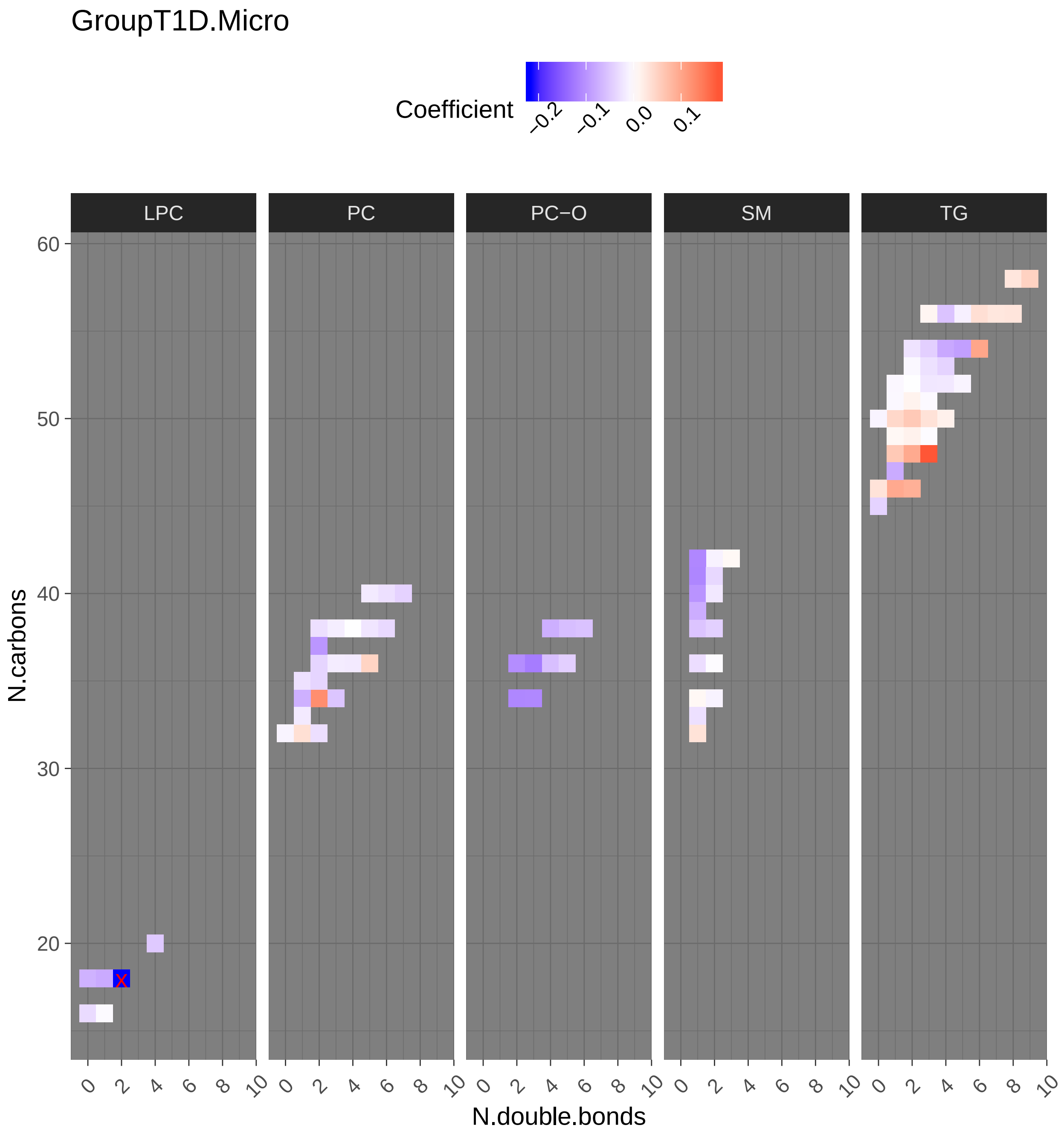

Figure 1: Heatmap of the lipid-specific model coefficents from the crude model for albuminuria groups. The name of the group is shown in the title of the figure in the top-left corner. Lipids are organized into panels (columns) by their class (from LPC to TG). One colored rectangle corresponds to one lipid species, where the coordinates in the x-axis and y-axis, respectively, show the number of double bonds (N.double.bonds) and the number of carbon atoms (N.carbons) in the fatty acid chains of the lipid. The number of double bonds corresponds to the number of unsaturated bonds in the fatty acid chains, and the number of carbon atoms corresponds to the length of the fatty acid chains and, thus, the size of the lipid. The magnitude of the lipid-specifc model coefficient is shown as the color of the rectangle (blue: negative; red: positive). Coefficient with statistical significance are annotated with symbols #, x and *, respectively, corresponding to $p < 0.01$, $p < 0.05$ and $p < 0.1$.

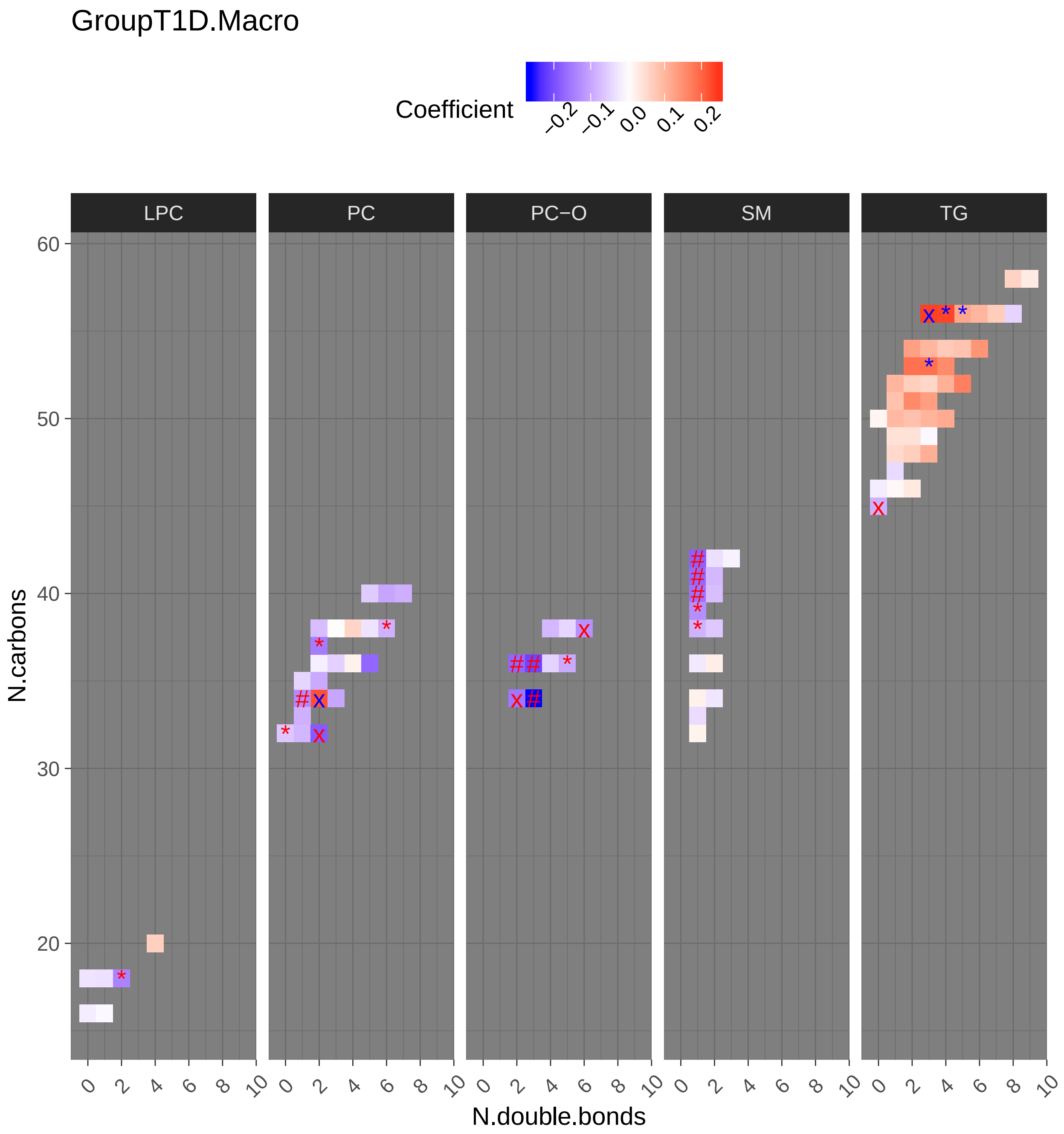

Figure 2: Heatmap of the lipid-specific model coefficents from the crude model for albuminuria groups. The name of the group is shown in the title of the figure in the top-left corner. Lipids are organized into panels (columns) by their class (from LPC to TG). One colored rectangle corresponds to one lipid species, where the coordinates in the x-axis and y-axis, respectively, show the number of double bonds (N.double.bonds) and the number of carbon atoms (N.carbons) in the fatty acid chains of the lipid. The number of double bonds corresponds to the number of unsaturated bonds in the fatty acid chains, and the number of carbon atoms corresponds to the length of the fatty acid chains and, thus, the size of the lipid. The magnitude of the lipid-specifc model coefficient is shown as the color of the rectangle (blue: negative; red: positive). Coefficient with statistical significance are annotated with symbols #, x and *, respectively, corresponding to $p < 0.01$, $p < 0.05$ and $p < 0.1$.

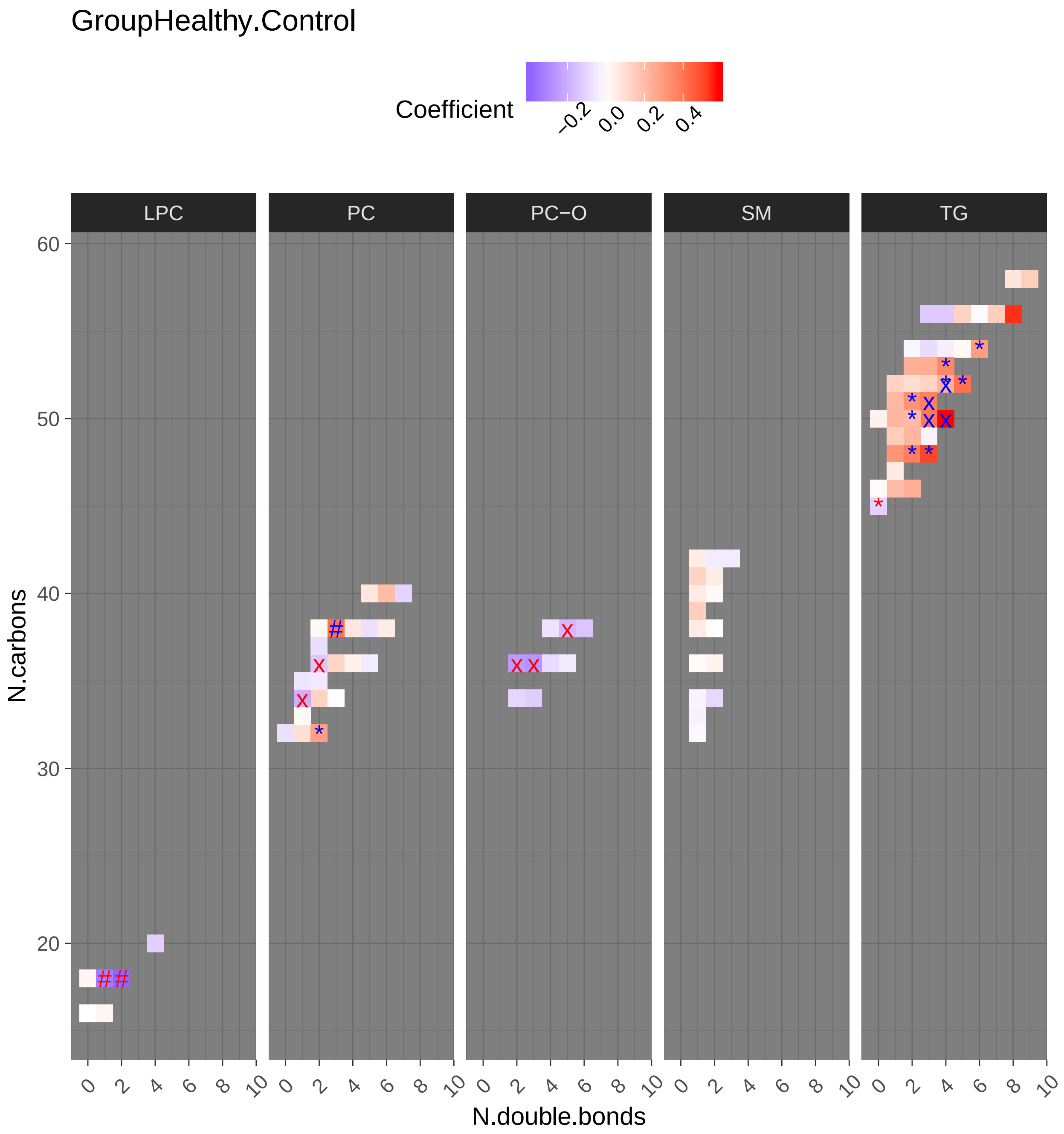

Figure 3: Heatmap of the lipid-specific model coefficents from the crude model for albuminuria groups. The name of the group is shown in the title of the figure in the top-left corner. Lipids are organized into panels (columns) by their class (from LPC to TG). One colored rectangle corresponds to one lipid species, where the coordinates in the x-axis and y-axis, respectively, show the number of double bonds (N.double.bonds) and the number of carbon atoms (N.carbons) in the fatty acid chains of the lipid. The number of double bonds corresponds to the number of unsaturated bonds in the fatty acid chains, and the number of carbon atoms corresponds to the length of the fatty acid chains and, thus, the size of the lipid. The magnitude of the lipid-specifc model coefficient is shown as the color of the rectangle (blue: negative; red: positive). Coefficient with statistical significance are annotated with symbols #, x and *, respectively, corresponding to $p < 0.01$, $p < 0.05$ and $p < 0.1$.

1.1.1.2 Adjusted Model

1.1.1.2.1 Table

```
##
##
## Table: Coefficients for GroupT1D Micro
## Results of the adjusted model for albuminuria group variable.
## Shown in the table are name of the lipid (Name),
## model coefficient (Coefficient),
## its lower and upper confidence intervals (CI.L, CL.R),
## average value of the variable (AveExpr),
## p-value of the coefficient (P.Value) and
## the p-value after correction for multiple testing (adj.P.Val).
## The rows of the table are shown in an increasing order by the respective p-value.
##
## Name                  Coefficient      CI.L        CI.R   AveExpr    P.Value   adj.P.Val
## -------------------  ------------  --------  ----------  --------  ---------  ----------
## LPC(18:2)                -0.193000   -0.3040   -8.25e-02      24.0   0.000648      0.0686
## TG(18:1/18:1/18:1)       -0.159000   -0.2580   -5.94e-02      24.8   0.001780      0.0946
## SM(d18:1/24:0)           -0.089100   -0.1520   -2.60e-02      23.6   0.005680      0.1690
## TG(54:3)                 -0.093900   -0.1630   -2.44e-02      21.7   0.008130      0.1690
## SM(d40:1)                -0.076500   -0.1340   -1.89e-02      24.0   0.009270      0.1690
## SM(d36:2)                -0.073900   -0.1310   -1.69e-02      21.4   0.011200      0.1690
## TG(54:2)                 -0.125000   -0.2230   -2.61e-02      23.4   0.013300      0.1690
## LPC(20:4)                -0.138000   -0.2480   -2.81e-02      21.4   0.013900      0.1690
## TG(56:5)                 -0.135000   -0.2430   -2.65e-02      20.3   0.014800      0.1690
## TG(54:4)                 -0.114000   -0.2060   -2.13e-02      21.4   0.016000      0.1690
## SM(d36:1)                -0.059100   -0.1090   -9.06e-03      23.4   0.020700      0.1880
## SM(d41:1)                -0.085200   -0.1580   -1.28e-02      22.7   0.021200      0.1880
## TG(56:4)                 -0.162000   -0.3030   -2.07e-02      20.0   0.024700      0.1900
## TG(16:0/22:5/18:1)       -0.123000   -0.2310   -1.43e-02      22.7   0.026600      0.1900
## TG(18:2/18:1/18:1)       -0.118000   -0.2230   -1.36e-02      24.4   0.026900      0.1900
## TG(18:1/18:2/18:2)       -0.142000   -0.2740   -1.01e-02      23.6   0.034900      0.2310
## LPC(18:1)                -0.086100   -0.1710   -1.58e-03      23.7   0.045900      0.2860
## TG(54:5)                 -0.118000   -0.2370   -4.78e-05      20.6   0.049900      0.2940
## PC(O-38:6)               -0.072700   -0.1470    1.29e-03      20.4   0.054100      0.3020
## SM(d38:1)                -0.055500   -0.1130    1.81e-03      23.1   0.057700      0.3060
## TG(52:2)                 -0.042800   -0.0880    2.42e-03      22.6   0.063500      0.3180
## SM(d38:2)                -0.060100   -0.1240    4.18e-03      21.3   0.066800      0.3180
## TG(52:3)                 -0.049600   -0.1030    3.89e-03      22.3   0.069100      0.3180
## TG(53:2)                 -0.102000   -0.2160    1.22e-02      20.7   0.080100      0.3460
## TG(48:3)                  0.159000   -0.0200    3.38e-01      20.6   0.081600      0.3460
## LPC(18:0)                -0.056900   -0.1220    8.08e-03      24.0   0.086000      0.3510
## TG(53:3)                 -0.096100   -0.2080    1.59e-02      20.7   0.092400      0.3630
## PC(O-38:5)               -0.060600   -0.1320    1.12e-02      22.7   0.097900      0.3710
## TG(18:1/18:1/16:0)       -0.082200   -0.1840    1.91e-02      25.6   0.112000      0.4080
## TG(56:6)                 -0.076800   -0.1730    1.98e-02      19.9   0.119000      0.4200
## PC(O-36:5)               -0.062100   -0.1420    1.80e-02      22.3   0.129000      0.4230
## TG(18:2/18:1/16:0)       -0.068100   -0.1570    2.05e-02      25.3   0.132000      0.4230
## SM(d16:1/18:1)           -0.036900   -0.0857    1.18e-02      22.8   0.137000      0.4230
## TG(18:1/12:0/18:1)        0.090500   -0.0307    2.12e-01      22.5   0.143000      0.4230
## PC(38:4)                 -0.039300   -0.0922    1.37e-02      25.2   0.146000      0.4230
## SM(d39:1)                -0.067100   -0.1590    2.44e-02      21.1   0.151000      0.4230
## TG(46:1)                  0.105000   -0.0382    2.48e-01      21.3   0.151000      0.4230
## PC(O-36:4)               -0.062000   -0.1470    2.28e-02      22.9   0.152000      0.4230
```

```
## TG(16:0/18:2/18:2)      -0.080700  -0.1930  3.13e-02   24.5  0.158000   0.4260
## LPC(16:0)               -0.025200  -0.0608  1.04e-02   25.3  0.165000   0.4260
## PC(O-36:2)              -0.058300  -0.1410  2.43e-02   20.5  0.166000   0.4260
## TG(45:0)                -0.023900  -0.0581  1.03e-02   23.1  0.170000   0.4260
## PC(32:2)                 0.085000  -0.0373  2.07e-01   20.8  0.173000   0.4260
## PC(O-36:3)              -0.058000  -0.1450  2.88e-02   20.4  0.190000   0.4430
## PC(O-38:4)              -0.063100  -0.1580  3.14e-02   21.7  0.190000   0.4430
## TG(56:3)                -0.077500  -0.1950  4.00e-02   19.9  0.196000   0.4430
## TG(16:0/18:2/18:3)      -0.122000  -0.3070  6.32e-02   22.0  0.196000   0.4430
## TG(16:0/18:0/18:1)      -0.101000  -0.2580  5.59e-02   23.1  0.207000   0.4450
## TG(46:2)                 0.093400  -0.0529  2.40e-01   20.1  0.211000   0.4450
## TG(18:0/18:1/20:4)      -0.111000  -0.2850  6.31e-02   21.3  0.211000   0.4450
## TG(53:4)                -0.086100  -0.2220  4.98e-02   19.7  0.214000   0.4450
## TG(50:2)                 0.042400  -0.0260  1.11e-01   21.2  0.224000   0.4560
## PC(36:4)                -0.025700  -0.0724  2.10e-02   26.0  0.280000   0.5410
## TG(56:7)                -0.064300  -0.1810  5.28e-02   19.6  0.281000   0.5410
## SM(d40:2)               -0.028200  -0.0795  2.31e-02   23.7  0.282000   0.5410
## PC(34:2)                 0.076000  -0.0637  2.16e-01   26.0  0.286000   0.5410
## TG(52:4)                -0.045400  -0.1310  4.06e-02   21.7  0.300000   0.5440
## TG(47:1)                -0.078000  -0.2270  7.05e-02   19.6  0.303000   0.5440
## PC(32:1)                 0.071000  -0.0660  2.08e-01   23.3  0.309000   0.5440
## TG(16:0/18:2/22:6)      -0.144000  -0.4240  1.35e-01   21.0  0.310000   0.5440
## TG(18:1/18:1/22:6)      -0.131000  -0.3890  1.27e-01   20.7  0.320000   0.5440
## PC(40:5)                -0.043700  -0.1300  4.29e-02   22.0  0.322000   0.5440
## PC(38:5)                -0.024300  -0.0725  2.40e-02   24.5  0.324000   0.5440
## SM(d41:2)               -0.034000  -0.1030  3.47e-02   22.5  0.331000   0.5490
## SM(d33:1)               -0.031600  -0.0984  3.53e-02   21.3  0.354000   0.5690
## TG(51:1)                -0.055500  -0.1730  6.21e-02   20.3  0.355000   0.5690
## PC(16:0e/18:1(9Z)       -0.032700  -0.1030  3.77e-02   21.8  0.362000   0.5710
## PC(40:6)                -0.044400  -0.1410  5.26e-02   23.9  0.369000   0.5710
## TG(14:0/18:1/18:1)       0.036400  -0.0436  1.16e-01   24.4  0.372000   0.5710
## PC(38:6)                -0.029300  -0.0954  3.67e-02   25.3  0.384000   0.5810
## TG(50:0)                -0.070200  -0.2310  9.10e-02   20.8  0.393000   0.5860
## TG(58:9)                -0.111000  -0.3710  1.49e-01   19.9  0.402000   0.5920
## TG(14:0/16:0/18:1)       0.053000  -0.0746  1.81e-01   22.8  0.415000   0.6020
## TG(18:2/22:5/16:0)      -0.081700  -0.2840  1.20e-01   22.1  0.427000   0.6110
## PC(O-34:3)              -0.032100  -0.1140  5.02e-02   21.9  0.444000   0.6280
## TG(18:2/18:2/18:2)      -0.055400  -0.2020  9.07e-02   22.1  0.457000   0.6370
## TG(51:2)                -0.034000  -0.1290  6.07e-02   21.6  0.481000   0.6560
## PC(35:1)                -0.028500  -0.1080  5.12e-02   21.8  0.483000   0.6560
## PC(36:2)                -0.020600  -0.0809  3.97e-02   26.1  0.502000   0.6740
## PC(34:1)                 0.022400  -0.0488  9.36e-02   25.9  0.537000   0.7110
## PC(O-34:2)              -0.033900  -0.1460  7.78e-02   21.5  0.551000   0.7150
## TG(46:0)                 0.042100  -0.0973  1.81e-01   20.4  0.553000   0.7150
## TG(52:5)                -0.046200  -0.2050  1.13e-01   19.8  0.568000   0.7260
## SM(d34:1)               -0.008390  -0.0388  2.20e-02   24.9  0.589000   0.7350
## SM(42:2)                -0.013500  -0.0624  3.55e-02   25.2  0.590000   0.7350
## TG(51:3)                -0.028900  -0.1400  8.23e-02   20.7  0.610000   0.7460
## PC(33:1)                 0.024000  -0.0688  1.17e-01   20.9  0.612000   0.7460
## PC(32:0)                 0.010800  -0.0364  5.80e-02   22.5  0.654000   0.7800
## SM(d32:1)                0.014200  -0.0480  7.63e-02   22.3  0.655000   0.7800
## PC(38:3)                -0.014800  -0.0914  6.18e-02   24.0  0.705000   0.8300
## PC(36:5)                -0.028300  -0.1820  1.25e-01   24.3  0.717000   0.8350
## PC(38:2)                 0.011600  -0.0590  8.22e-02   21.3  0.748000   0.8610
```

```
## TG(49:2)                 0.013700   -0.0791    1.06e-01      20.4   0.772000      0.8800
## PC(40:7)                -0.012500   -0.1090    8.45e-02      21.4   0.801000      0.9030
## PC(37:2)                -0.009400   -0.1190    1.00e-01      19.3   0.867000      0.9610
## TG(14:0/18:2/18:2)      -0.011200   -0.1470    1.25e-01      21.2   0.872000      0.9610
## LPC(16:1)                0.008170   -0.1000    1.17e-01      20.0   0.882000      0.9610
## TG(50:1)                 0.008810   -0.1150    1.32e-01      24.3   0.889000      0.9610
## TG(50:3)                 0.004710   -0.0734    8.28e-02      23.5   0.906000      0.9670
## PC(34:3)                 0.007930   -0.1330    1.49e-01      22.5   0.912000      0.9670
## SM(d18:2/24:1)          -0.002360   -0.0589    5.42e-02      24.0   0.935000      0.9750
## PC(36:3)                 0.002540   -0.0751    8.01e-02      25.6   0.949000      0.9750
## TG(54:6)                 0.005130   -0.1540    1.64e-01      19.5   0.949000      0.9750
## TG(49:3)                 0.000334   -0.0140    1.47e-02      20.7   0.963000      0.9750
## PC(35:2)                -0.001960   -0.0923    8.83e-02      21.8   0.966000      0.9750
## TG(49:1)                -0.001130   -0.1070    1.04e-01      20.9   0.983000      0.9830
##
##
## Table: Coefficients for GroupT1D Macro
## Results of the adjusted model for albuminuria group variable.
## Shown in the table are name of the lipid (Name),
## model coefficient (Coefficient),
## its lower and upper confidence intervals (CI.L, CL.R),
## average value of the variable (AveExpr),
## p-value of the coefficient (P.Value) and
## the p-value after correction for multiple testing (adj.P.Val).
## The rows of the table are shown in an increasing order by the respective p-value.
##
## Name                   Coefficient       CI.L       CI.R   AveExpr    P.Value   adj.P.Val
## -------------------  ------------  ---------  ---------  --------  ---------  ----------
## SM(d40:1)                 -0.15600   -0.22000   -0.09170      24.0   2.20e-06    0.000228
## SM(d18:1/24:0)            -0.15300   -0.22300   -0.08330      23.6   1.95e-05    0.001030
## SM(d41:1)                 -0.17000   -0.25000   -0.08920      22.7   3.93e-05    0.001390
## SM(d38:1)                 -0.13100   -0.19400   -0.06700      23.1   6.16e-05    0.001630
## SM(d40:2)                 -0.11200   -0.16900   -0.05470      23.7   1.29e-04    0.002740
## TG(54:2)                  -0.19600   -0.30500   -0.08640      23.4   4.72e-04    0.008340
## SM(d38:2)                 -0.12400   -0.19500   -0.05220      21.3   7.12e-04    0.009630
## SM(d39:1)                 -0.17600   -0.27700   -0.07400      21.1   7.27e-04    0.009630
## PC(O-34:3)                -0.14700   -0.23800   -0.05570      21.9   1.65e-03    0.019400
## TG(52:2)                  -0.07900   -0.12900   -0.02880      22.6   2.08e-03    0.022000
## PC(O-36:2)                -0.14100   -0.23300   -0.04940      20.5   2.62e-03    0.024600
## SM(d36:2)                 -0.09670   -0.16000   -0.03350      21.4   2.78e-03    0.024600
## TG(52:3)                  -0.08990   -0.14900   -0.03050      22.3   3.07e-03    0.025000
## SM(d36:1)                 -0.08050   -0.13600   -0.02490      23.4   4.60e-03    0.034800
## SM(d41:2)                 -0.10700   -0.18300   -0.03040      22.5   6.21e-03    0.043900
## TG(54:3)                  -0.10600   -0.18300   -0.02850      21.7   7.32e-03    0.047100
## TG(53:2)                  -0.17300   -0.29900   -0.04620      20.7   7.55e-03    0.047100
## PC(O-38:6)                -0.10900   -0.19100   -0.02650      20.4   9.58e-03    0.054300
## SM(d16:1/18:1)            -0.07150   -0.12600   -0.01740      22.8   9.72e-03    0.054300
## PC(O-38:4)                -0.13400   -0.23900   -0.02890      21.7   1.25e-02    0.064900
## PC(O-36:5)                -0.11300   -0.20200   -0.02400      22.3   1.29e-02    0.064900
## PC(O-36:3)                -0.12100   -0.21700   -0.02450      20.4   1.40e-02    0.066800
## TG(18:2/18:1/18:1)        -0.14500   -0.26100   -0.02880      24.4   1.46e-02    0.066800
## TG(18:1/18:1/18:1)        -0.13700   -0.24700   -0.02650      24.8   1.51e-02    0.066800
## TG(56:5)                  -0.13900   -0.25900   -0.01850      20.3   2.38e-02    0.101000
## TG(16:0/22:5/18:1)        -0.13800   -0.25900   -0.01760      22.7   2.47e-02    0.101000
```

```
## TG(54:4)               -0.11400   -0.21600   -0.01120      21.4   2.97e-02    0.117000
## TG(56:6)               -0.10900   -0.21700   -0.00229      19.9   4.53e-02    0.172000
## PC(35:1)               -0.08650   -0.17500    0.00197      21.8   5.53e-02    0.198000
## TG(51:1)               -0.12700   -0.25800    0.00326      20.3   5.60e-02    0.198000
## TG(16:0/18:0/18:1)     -0.15600   -0.33000    0.01800      23.1   7.89e-02    0.263000
## SM(d33:1)              -0.06590   -0.14000    0.00828      21.3   8.16e-02    0.263000
## TG(16:0/18:2/18:2)     -0.11000   -0.23500    0.01400      24.5   8.20e-02    0.263000
## TG(50:0)               -0.15700   -0.33600    0.02190      20.8   8.54e-02    0.266000
## PC(34:2)                0.13500   -0.02000    0.29000      26.0   8.78e-02    0.266000
## PC(35:2)               -0.08590   -0.18600    0.01430      21.8   9.28e-02    0.267000
## TG(52:4)               -0.08160   -0.17700    0.01390      21.7   9.37e-02    0.267000
## TG(53:3)               -0.10600   -0.23000    0.01870      20.7   9.56e-02    0.267000
## PC(38:3)               -0.07020   -0.15500    0.01490      24.0   1.06e-01    0.284000
## TG(50:3)               -0.07080   -0.15800    0.01590      23.5   1.09e-01    0.284000
## PC(36:3)               -0.07010   -0.15600    0.01600      25.6   1.10e-01    0.284000
## TG(18:0/18:1/20:4)     -0.15600   -0.34900    0.03690      21.3   1.13e-01    0.284000
## PC(38:4)               -0.04720   -0.10600    0.01160      25.2   1.15e-01    0.284000
## PC(16:0e/18:1(9Z)      -0.06180   -0.14000    0.01630      21.8   1.21e-01    0.289000
## TG(56:4)               -0.12400   -0.28100    0.03340      20.0   1.23e-01    0.289000
## TG(51:2)               -0.08060   -0.18600    0.02450      21.6   1.33e-01    0.304000
## TG(18:1/18:2/18:2)     -0.11200   -0.25800    0.03480      23.6   1.35e-01    0.304000
## PC(O-36:4)             -0.06740   -0.16200    0.02680      22.9   1.61e-01    0.355000
## TG(54:5)               -0.09120   -0.22300    0.04010      20.6   1.73e-01    0.374000
## TG(53:4)               -0.10300   -0.25400    0.04760      19.7   1.79e-01    0.380000
## PC(O-38:5)             -0.05360   -0.13300    0.02610      22.7   1.87e-01    0.389000
## PC(38:5)               -0.03420   -0.08780    0.01930      24.5   2.10e-01    0.428000
## LPC(20:4)              -0.07590   -0.19800    0.04620      21.4   2.23e-01    0.445000
## TG(51:3)               -0.07460   -0.19800    0.04890      20.7   2.36e-01    0.459000
## PC(36:5)               -0.10200   -0.27300    0.06800      24.3   2.38e-01    0.459000
## LPC(18:2)              -0.07270   -0.19600    0.05020      24.0   2.46e-01    0.459000
## TG(18:2/18:1/16:0)     -0.05800   -0.15600    0.04040      25.3   2.48e-01    0.459000
## LPC(16:1)               0.06960   -0.05080    0.19000      20.0   2.57e-01    0.459000
## TG(18:1/18:1/16:0)     -0.06500   -0.17800    0.04750      25.6   2.57e-01    0.459000
## TG(49:1)               -0.06720   -0.18400    0.04980      20.9   2.60e-01    0.459000
## TG(56:3)               -0.06900   -0.19900    0.06140      19.9   2.99e-01    0.520000
## TG(14:0/18:1/18:1)     -0.04600   -0.13500    0.04280      24.4   3.10e-01    0.529000
## TG(16:0/18:2/22:6)     -0.15400   -0.46400    0.15600      21.0   3.29e-01    0.554000
## TG(49:3)                0.00773   -0.00817    0.02360      20.7   3.40e-01    0.563000
## SM(42:2)               -0.02470   -0.07900    0.02970      25.2   3.73e-01    0.608000
## PC(33:1)               -0.04620   -0.14900    0.05680      20.9   3.79e-01    0.609000
## TG(14:0/18:2/18:2)     -0.06500   -0.21600    0.08580      21.2   3.98e-01    0.621000
## SM(d18:2/24:1)         -0.02680   -0.08950    0.03600      24.0   4.03e-01    0.621000
## TG(50:1)               -0.05820   -0.19500    0.07880      24.3   4.04e-01    0.621000
## TG(49:2)               -0.04150   -0.14400    0.06150      20.4   4.29e-01    0.650000
## PC(37:2)               -0.04710   -0.16900    0.07480      19.3   4.48e-01    0.669000
## PC(40:5)               -0.03600   -0.13200    0.06010      22.0   4.62e-01    0.680000
## SM(d32:1)              -0.02510   -0.09410    0.04400      22.3   4.76e-01    0.691000
## TG(45:0)                0.01350   -0.02450    0.05140      23.1   4.86e-01    0.696000
## PC(32:0)                0.01720   -0.03520    0.06960      22.5   5.20e-01    0.735000
## PC(38:6)               -0.02340   -0.09680    0.04990      25.3   5.31e-01    0.740000
## PC(36:2)               -0.02050   -0.08750    0.04640      26.1   5.48e-01    0.754000
## PC(40:6)               -0.03230   -0.14000    0.07540      23.9   5.56e-01    0.755000
## TG(50:2)               -0.02210   -0.09790    0.05380      21.2   5.68e-01    0.763000
## TG(18:2/18:2/18:2)     -0.04380   -0.20600    0.11800      22.1   5.96e-01    0.782000
```

```
## TG(52:5)                 -0.04690   -0.22300    0.13000      19.8   6.02e-01    0.782000
## SM(d34:1)                 0.00869   -0.02510    0.04250      24.9   6.14e-01    0.782000
## TG(46:1)                 -0.03820   -0.19700    0.12000      21.3   6.36e-01    0.782000
## TG(16:0/18:2/18:3)       -0.04950   -0.25500    0.15600      22.0   6.37e-01    0.782000
## TG(56:7)                 -0.03090   -0.16100    0.09920      19.6   6.41e-01    0.782000
## TG(18:1/12:0/18:1)       -0.03200   -0.16700    0.10300      22.5   6.41e-01    0.782000
## TG(54:6)                 -0.04180   -0.21800    0.13500      19.5   6.42e-01    0.782000
## TG(47:1)                 -0.03710   -0.20200    0.12800      19.6   6.59e-01    0.794000
## TG(46:2)                 -0.03420   -0.19700    0.12800      20.1   6.79e-01    0.809000
## TG(14:0/16:0/18:1)       -0.02720   -0.16900    0.11400      22.8   7.07e-01    0.832000
## PC(O-34:2)               -0.02290   -0.14700    0.10100      21.5   7.17e-01    0.835000
## PC(38:2)                 -0.01340   -0.09180    0.06500      21.3   7.37e-01    0.849000
## PC(32:2)                 -0.02220   -0.15800    0.11400      20.8   7.48e-01    0.851000
## TG(46:0)                 -0.02410   -0.17900    0.13100      20.4   7.60e-01    0.851000
## TG(18:2/22:5/16:0)       -0.03340   -0.25700    0.19100      22.1   7.70e-01    0.851000
## PC(32:1)                  0.02230   -0.13000    0.17400      23.3   7.74e-01    0.851000
## TG(48:3)                 -0.02680   -0.22600    0.17200      20.6   7.91e-01    0.851000
## TG(18:1/18:1/22:6)        0.03850   -0.24800    0.32500      20.7   7.92e-01    0.851000
## LPC(18:1)                -0.01240   -0.10600    0.08140      23.7   7.95e-01    0.851000
## LPC(16:0)                 0.00421   -0.03530    0.04370      25.3   8.34e-01    0.884000
## TG(58:9)                 -0.02750   -0.31600    0.26100      19.9   8.52e-01    0.886000
## PC(34:1)                  0.00749   -0.07160    0.08660      25.9   8.52e-01    0.886000
## LPC(18:0)                 0.00534   -0.06680    0.07750      24.0   8.85e-01    0.910000
## PC(36:4)                 -0.00304   -0.05490    0.04880      26.0   9.08e-01    0.926000
## PC(40:7)                 -0.00257   -0.11000    0.10500      21.4   9.63e-01    0.972000
## PC(34:3)                 -0.00029   -0.15700    0.15600      22.5   9.97e-01    0.997000
##
##
## Table: Coefficients for GroupHealthy Control
## Results of the adjusted model for albuminuria group variable.
## Shown in the table are name of the lipid (Name),
## model coefficient (Coefficient),
## its lower and upper confidence intervals (CI.L, CL.R),
## average value of the variable (AveExpr),
## p-value of the coefficient (P.Value) and
## the p-value after correction for multiple testing (adj.P.Val).
## The rows of the table are shown in an increasing order by the respective p-value.
##
## Name                   Coefficient        CI.L        CI.R   AveExpr    P.Value    adj.P.Val
## -------------------  ------------  ----------  ----------  --------  ---------  ----------
## LPC(18:1)               -0.432000   -0.584000   -0.27900      23.7   0.00e+00    4.40e-06
## PC(16:0e/18:1(9Z)       -0.316000   -0.443000   -0.18900      21.8   1.30e-06    6.70e-05
## PC(32:0)                -0.189000   -0.274000   -0.10300      22.5   1.62e-05    4.99e-04
## PC(O-36:3)              -0.344000   -0.501000   -0.18700      20.4   1.88e-05    4.99e-04
## LPC(18:2)               -0.426000   -0.626000   -0.22500      24.0   3.36e-05    7.13e-04
## PC(38:5)                -0.179000   -0.266000   -0.09200      24.5   6.07e-05    1.07e-03
## PC(O-36:2)              -0.299000   -0.449000   -0.15000      20.5   9.08e-05    1.26e-03
## PC(38:3)                 0.277000    0.138000    0.41500      24.0   9.54e-05    1.26e-03
## PC(40:7)                -0.333000   -0.508000   -0.15800      21.4   2.06e-04    2.43e-03
## PC(O-34:3)              -0.277000   -0.425000   -0.12800      21.9   2.80e-04    2.97e-03
## PC(O-38:5)              -0.236000   -0.366000   -0.10700      22.7   3.73e-04    3.60e-03
## TG(50:3)                 0.249000    0.108000    0.39000      23.5   5.58e-04    4.93e-03
## PC(36:2)                -0.185000   -0.294000   -0.07580      26.1   9.18e-04    7.49e-03
## PC(O-38:6)              -0.223000   -0.357000   -0.08960      20.4   1.09e-03    8.25e-03
```

```
## TG(16:0/18:2/18:2)        0.327000    0.124000    0.52900        24.5   1.58e-03    1.12e-02
## TG(51:3)                  0.309000    0.108000    0.51000        20.7   2.59e-03    1.72e-02
## TG(53:3)                  0.304000    0.102000    0.50600        20.7   3.27e-03    2.04e-02
## SM(d16:1/18:1)           -0.131000   -0.219000   -0.04300        22.8   3.59e-03    2.11e-02
## TG(52:4)                  0.226000    0.070400    0.38100        21.7   4.46e-03    2.40e-02
## TG(14:0/18:2/18:2)        0.356000    0.111000    0.60200        21.2   4.53e-03    2.40e-02
## SM(d39:1)                 0.229000    0.063300    0.39400        21.1   6.79e-03    3.04e-02
## TG(51:2)                  0.236000    0.065100    0.40700        21.6   6.89e-03    3.04e-02
## TG(53:4)                  0.338000    0.092500    0.58400        19.7   7.04e-03    3.04e-02
## SM(42:2)                 -0.122000   -0.210000   -0.03330        25.2   7.05e-03    3.04e-02
## TG(45:0)                 -0.084900   -0.147000   -0.02310        23.1   7.17e-03    3.04e-02
## TG(53:2)                  0.281000    0.074400    0.48700        20.7   7.71e-03    3.14e-02
## TG(52:5)                  0.387000    0.099900    0.67400        19.8   8.32e-03    3.22e-02
## PC(38:2)                 -0.172000   -0.299000   -0.04390        21.3   8.49e-03    3.22e-02
## TG(16:0/18:2/18:3)        0.440000    0.105000    0.77500        22.0   1.01e-02    3.69e-02
## TG(52:3)                  0.122000    0.025700    0.21900        22.3   1.32e-02    4.67e-02
## TG(18:2/18:2/18:2)        0.328000    0.064300    0.59200        22.1   1.49e-02    5.09e-02
## SM(d36:2)                 0.125000    0.022400    0.22800        21.4   1.71e-02    5.66e-02
## SM(d18:2/24:1)           -0.122000   -0.224000   -0.02010        24.0   1.91e-02    6.01e-02
## PC(37:2)                 -0.237000   -0.436000   -0.03870        19.3   1.93e-02    6.01e-02
## LPC(16:0)                -0.073200   -0.138000   -0.00888        25.3   2.58e-02    7.81e-02
## PC(O-34:2)               -0.228000   -0.430000   -0.02640        21.5   2.67e-02    7.87e-02
## LPC(16:1)                -0.208000   -0.404000   -0.01220        20.0   3.74e-02    1.07e-01
## TG(16:0/18:2/22:6)        0.514000    0.009420    1.02000        21.0   4.59e-02    1.28e-01
## TG(14:0/18:1/18:1)        0.145000    0.000898    0.29000        24.4   4.86e-02    1.32e-01
## SM(d41:1)                 0.130000   -0.000909    0.26100        22.7   5.16e-02    1.37e-01
## TG(52:2)                  0.077800   -0.003870    0.16000        22.6   6.19e-02    1.54e-01
## PC(O-36:4)               -0.146000   -0.299000    0.00775        22.9   6.27e-02    1.54e-01
## PC(36:5)                 -0.263000   -0.540000    0.01450        24.3   6.32e-02    1.54e-01
## TG(48:3)                  0.306000   -0.017700    0.63000        20.6   6.39e-02    1.54e-01
## PC(34:2)                  0.234000   -0.018300    0.48600        26.0   6.90e-02    1.63e-01
## PC(40:5)                 -0.144000   -0.301000    0.01240        22.0   7.11e-02    1.64e-01
## TG(18:2/22:5/16:0)        0.334000   -0.030900    0.69800        22.1   7.28e-02    1.64e-01
## TG(16:0/22:5/18:1)        0.178000   -0.018500    0.37400        22.7   7.58e-02    1.67e-01
## TG(56:5)                  0.170000   -0.026100    0.36500        20.3   8.93e-02    1.93e-01
## TG(18:2/18:1/16:0)        0.138000   -0.022400    0.29800        25.3   9.18e-02    1.95e-01
## PC(33:1)                 -0.141000   -0.309000    0.02640        20.9   9.86e-02    2.03e-01
## PC(38:4)                  0.080000   -0.015700    0.17600        25.2   1.01e-01    2.03e-01
## TG(46:0)                 -0.210000   -0.462000    0.04170        20.4   1.02e-01    2.03e-01
## SM(d38:1)                 0.085800   -0.017700    0.18900        23.1   1.04e-01    2.04e-01
## TG(56:3)                 -0.173000   -0.386000    0.03900        19.9   1.09e-01    2.11e-01
## TG(54:6)                  0.221000   -0.065800    0.50800        19.5   1.31e-01    2.47e-01
## PC(34:3)                 -0.195000   -0.450000    0.05960        22.5   1.33e-01    2.47e-01
## PC(32:1)                 -0.188000   -0.435000    0.06000        23.3   1.37e-01    2.51e-01
## TG(18:1/12:0/18:1)        0.164000   -0.055100    0.38300        22.5   1.42e-01    2.55e-01
## PC(35:1)                 -0.106000   -0.250000    0.03810        21.8   1.49e-01    2.64e-01
## PC(38:6)                 -0.084200   -0.204000    0.03530        25.3   1.67e-01    2.89e-01
## TG(18:1/18:1/16:0)        0.128000   -0.054700    0.31200        25.6   1.69e-01    2.89e-01
## TG(18:1/18:2/18:2)        0.156000   -0.082800    0.39400        23.6   2.00e-01    3.37e-01
## TG(54:3)                 -0.080400   -0.206000    0.04510        21.7   2.09e-01    3.46e-01
## TG(56:7)                  0.132000   -0.079900    0.34300        19.6   2.22e-01    3.62e-01
## TG(18:0/18:1/20:4)        0.193000   -0.122000    0.50700        21.3   2.29e-01    3.68e-01
## PC(O-36:5)               -0.085700   -0.230000    0.05900        22.3   2.45e-01    3.88e-01
## TG(50:2)                  0.068100   -0.055400    0.19200        21.2   2.79e-01    4.35e-01
```

```
## TG(51:1)              0.109000  -0.103000  0.32200     20.3  3.13e-01  4.81e-01
## LPC(18:0)            -0.058000  -0.175000  0.05940     24.0  3.32e-01  5.03e-01
## PC(35:2)             -0.078600  -0.242000  0.08450     21.8  3.44e-01  5.08e-01
## SM(d36:1)             0.043600  -0.046900  0.13400     23.4  3.45e-01  5.08e-01
## SM(d41:2)             0.058600  -0.065600  0.18300     22.5  3.55e-01  5.15e-01
## TG(49:2)              0.076100  -0.091500  0.24400     20.4  3.73e-01  5.34e-01
## TG(49:3)             -0.011300  -0.037200  0.01460     20.7  3.92e-01  5.54e-01
## TG(56:4)             -0.110000  -0.365000  0.14600     20.0  4.00e-01  5.58e-01
## LPC(20:4)            -0.080700  -0.279000  0.11800     21.4  4.25e-01  5.84e-01
## SM(d40:1)             0.041900  -0.062100  0.14600     24.0  4.29e-01  5.84e-01
## TG(58:9)              0.182000  -0.288000  0.65300     19.9  4.47e-01  5.99e-01
## TG(47:1)             -0.097400  -0.366000  0.17100     19.6  4.77e-01  6.32e-01
## TG(14:0/16:0/18:1)    0.075300  -0.155000  0.30600     22.8  5.21e-01  6.82e-01
## TG(50:1)              0.070700  -0.152000  0.29400     24.3  5.34e-01  6.83e-01
## PC(36:3)              0.044300  -0.095900  0.18500     25.6  5.35e-01  6.83e-01
## TG(54:5)              0.064500  -0.149000  0.27800     20.6  5.53e-01  6.98e-01
## TG(18:2/18:1/18:1)    0.049600  -0.140000  0.23900     24.4  6.07e-01  7.39e-01
## PC(O-38:4)           -0.044100  -0.215000  0.12700     21.7  6.13e-01  7.39e-01
## TG(56:6)              0.044900  -0.130000  0.21900     19.9  6.13e-01  7.39e-01
## PC(36:4)              0.021700  -0.062700  0.10600     26.0  6.14e-01  7.39e-01
## TG(18:1/18:1/18:1)   -0.043200  -0.223000  0.13600     24.8  6.37e-01  7.58e-01
## TG(54:2)             -0.041900  -0.220000  0.13600     23.4  6.45e-01  7.59e-01
## TG(50:0)             -0.051900  -0.343000  0.23900     20.8  7.26e-01  8.46e-01
## PC(32:2)              0.035500  -0.186000  0.25700     20.8  7.53e-01  8.67e-01
## SM(d34:1)            -0.008540  -0.063500  0.04650     24.9  7.61e-01  8.67e-01
## SM(d40:2)             0.012200  -0.080500  0.10500     23.7  7.96e-01  8.98e-01
## PC(40:6)              0.016400  -0.159000  0.19200     23.9  8.54e-01  9.53e-01
## SM(d18:1/24:0)       -0.009170  -0.123000  0.10500     23.6  8.74e-01  9.66e-01
## PC(34:1)             -0.009240  -0.138000  0.11900     25.9  8.88e-01  9.70e-01
## TG(46:1)             -0.016800  -0.275000  0.24100     21.3  8.99e-01  9.72e-01
## TG(18:1/18:1/22:6)    0.025400  -0.441000  0.49200     20.7  9.15e-01  9.79e-01
## SM(d38:2)            -0.004960  -0.121000  0.11100     21.3  9.33e-01  9.80e-01
## TG(46:2)              0.011200  -0.253000  0.27600     20.1  9.34e-01  9.80e-01
## TG(54:4)             -0.005690  -0.173000  0.16100     21.4  9.47e-01  9.81e-01
## TG(16:0/18:0/18:1)    0.007630  -0.276000  0.29100     23.1  9.58e-01  9.81e-01
## SM(d33:1)            -0.002860  -0.124000  0.11800     21.3  9.63e-01  9.81e-01
## SM(d32:1)             0.001890  -0.110000  0.11400     22.3  9.74e-01  9.83e-01
## TG(49:1)             -0.000983  -0.191000  0.19000     20.9  9.92e-01  9.92e-01
```

1.1.1.2.2 Heatmaps

```
## [1] "map_lipid_names has been created by Tommi Suvitaival"
## [1] "tommi.raimo.leo.suvitaival@regionh.dk"
## [1] "2019-05-06"
```

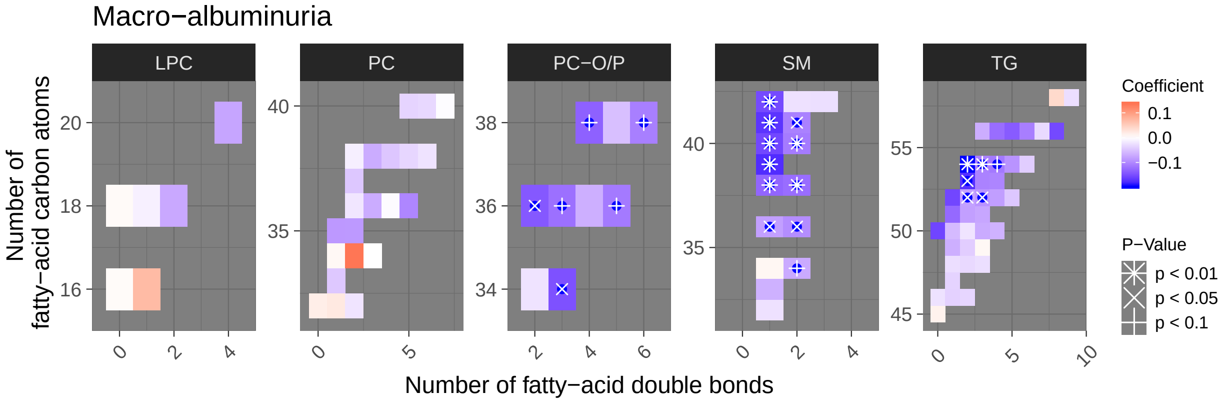


```
##   Label Relation p.adj
## 1     #  p.adj <  0.01
## 2     x  p.adj <  0.05
## 3     *  p.adj <  0.10

##   Label Relation p.adj
## 1     #  p.adj <  0.01
## 2     x  p.adj <  0.05
## 3     *  p.adj <  0.10

##   Label Relation p.adj
## 1     #  p.adj <  0.01
## 2     x  p.adj <  0.05
## 3     *  p.adj <  0.10
```

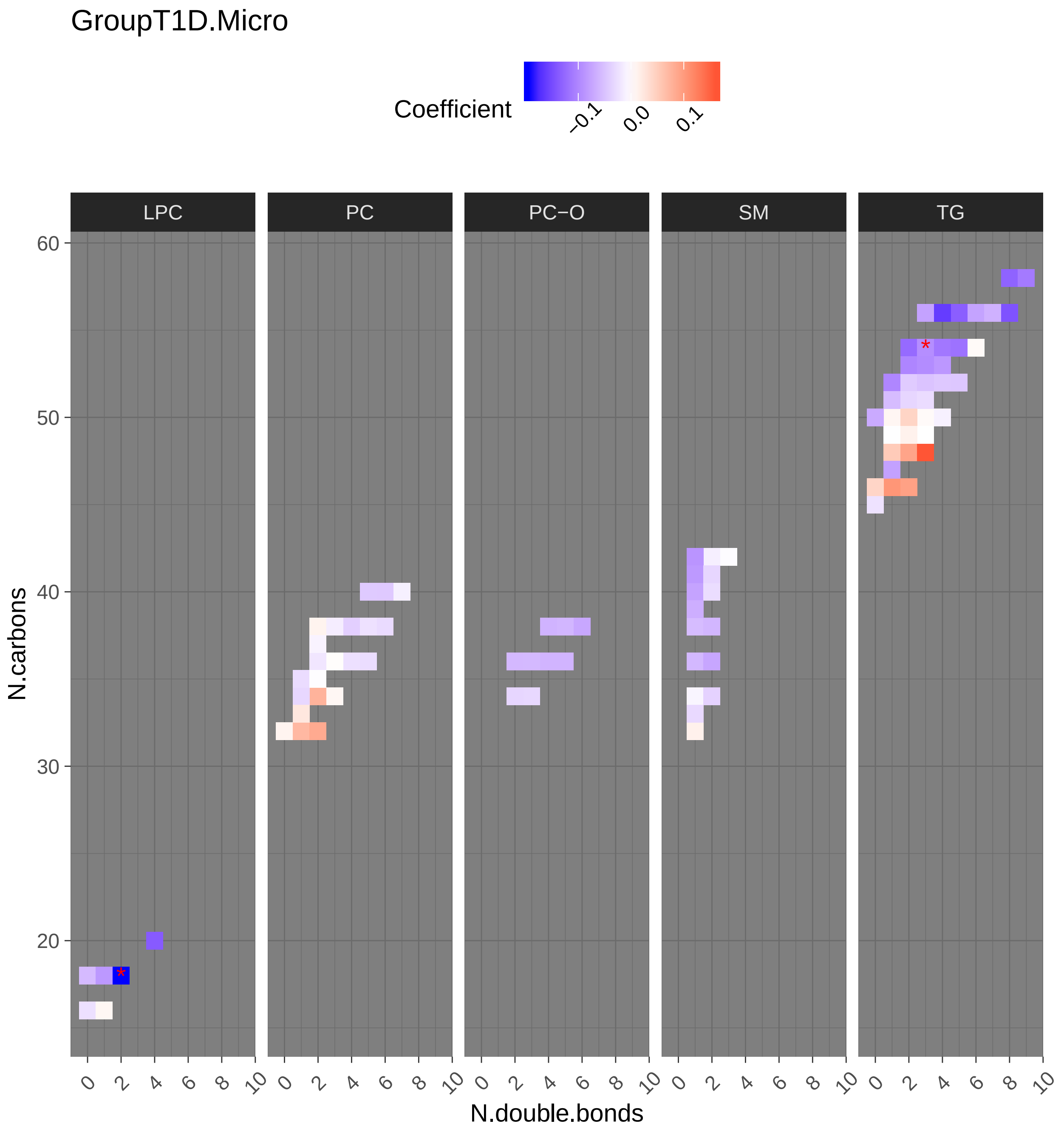

Figure 4: Heatmap of the lipid-specific model coefficents from the adjusted model for albuminuria groups. The name of the group is shown in the title of the figure in the top-left corner. Lipids are organized into panels (columns) by their class (from LPC to TG). One colored rectangle corresponds to one lipid species, where the coordinates in the x-axis and y-axis, respectively, show the number of double bonds (N.double.bonds) and the number of carbon atoms (N.carbons) in the fatty acid chains of the lipid. The number of double bonds corresponds to the number of unsaturated bonds in the fatty acid chains, and the number of carbon atoms corresponds to the length of the fatty acid chains and, thus, the size of the lipid. The magnitude of the lipid-specifc model coefficient is shown as the color of the rectangle (blue: negative; red: positive). Coefficient with statistical significance are annotated with symbols #, x and *, respectively, corresponding to $p < 0.01$, $p < 0.05$ and $p < 0.1$.

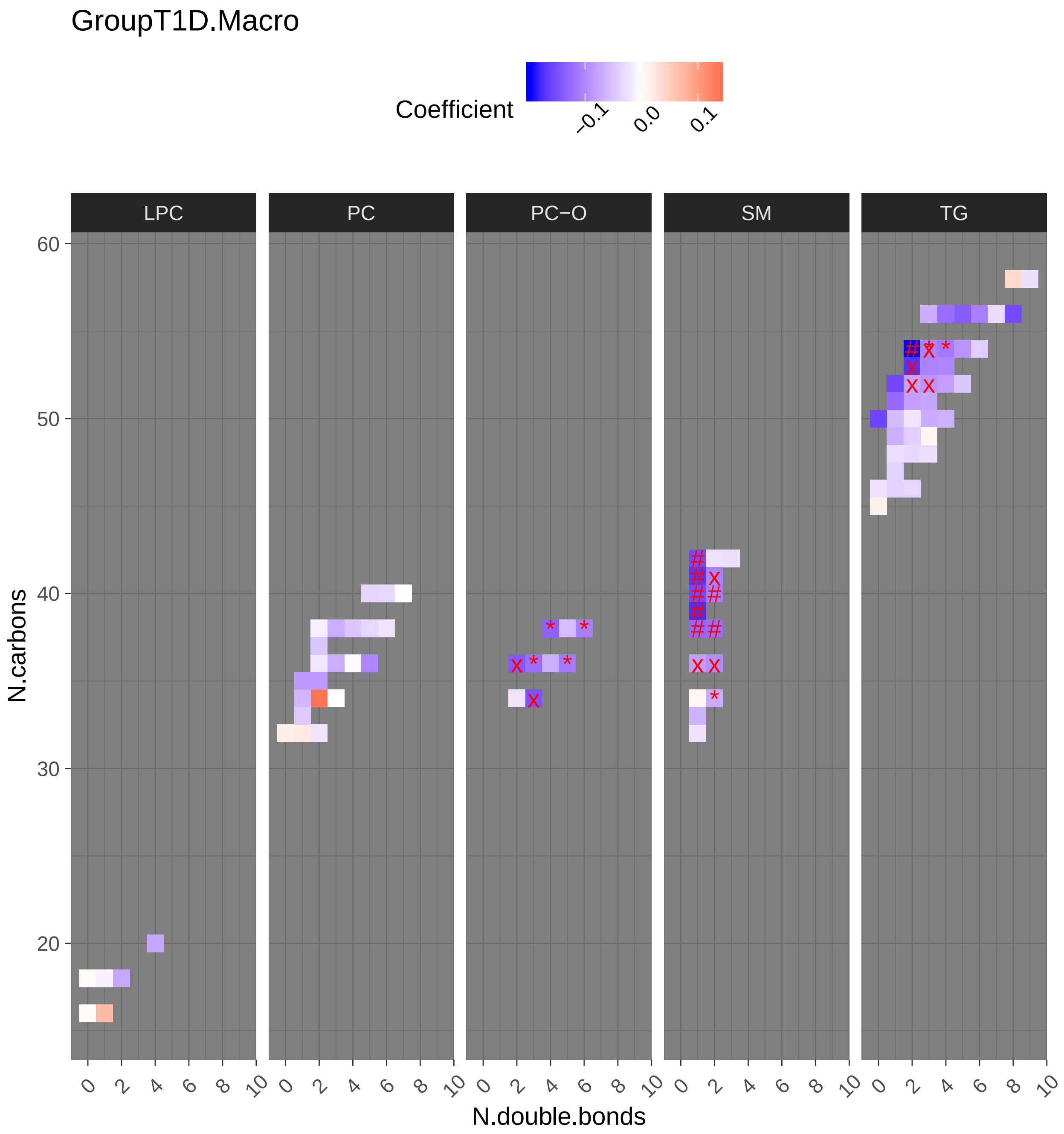

Figure 5: Heatmap of the lipid-specific model coefficents from the adjusted model for albuminuria groups. The name of the group is shown in the title of the figure in the top-left corner. Lipids are organized into panels (columns) by their class (from LPC to TG). One colored rectangle corresponds to one lipid species, where the coordinates in the x-axis and y-axis, respectively, show the number of double bonds (N.double.bonds) and the number of carbon atoms (N.carbons) in the fatty acid chains of the lipid. The number of double bonds corresponds to the number of unsaturated bonds in the fatty acid chains, and the number of carbon atoms corresponds to the length of the fatty acid chains and, thus, the size of the lipid. The magnitude of the lipid-specifc model coefficient is shown as the color of the rectangle (blue: negative; red: positive). Coefficient with statistical significance are annotated with symbols #, x and *, respectively, corresponding to $p < 0.01$, $p < 0.05$ and $p < 0.1$.

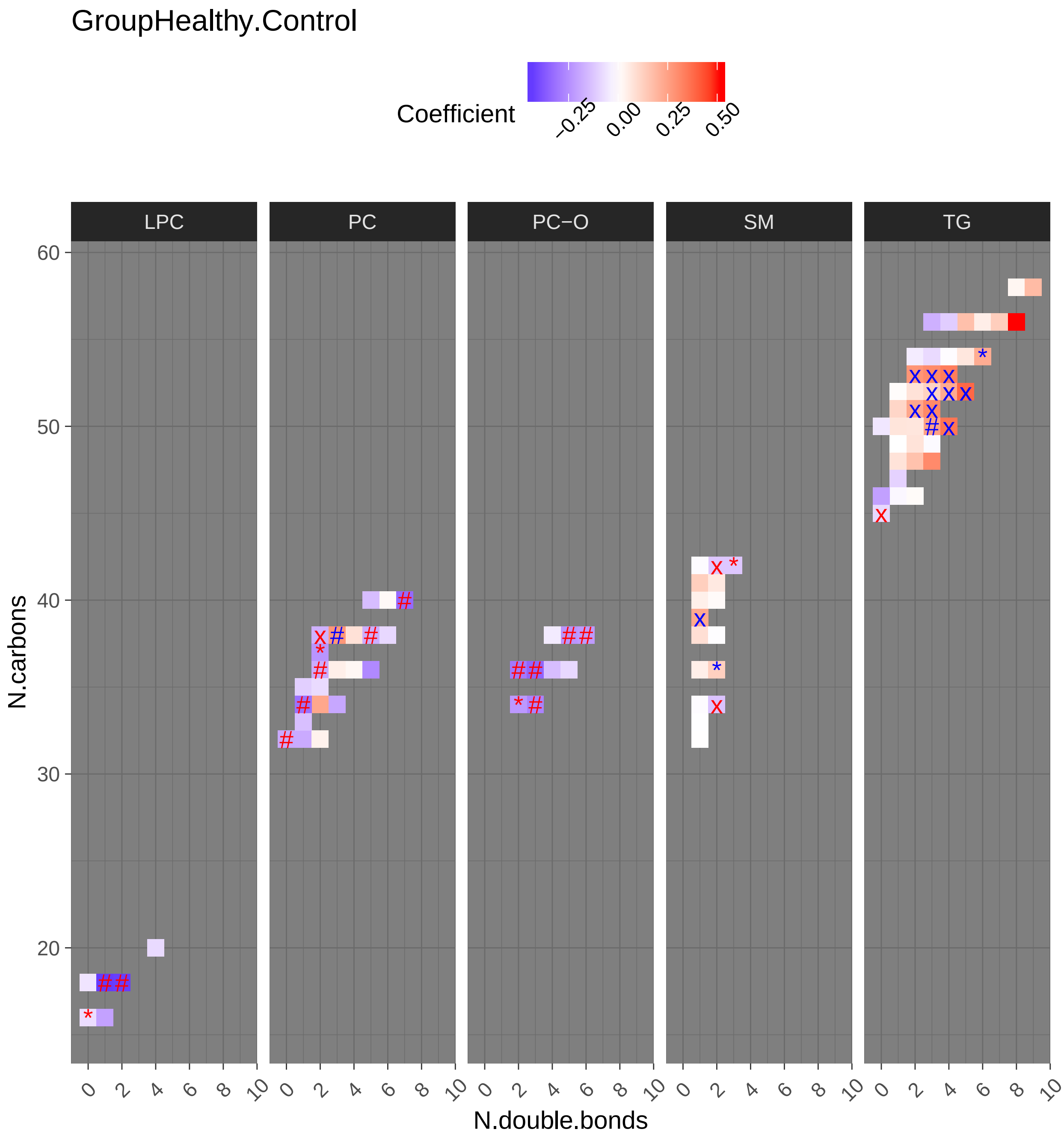

Figure 6: Heatmap of the lipid-specific model coefficents from the adjusted model for albuminuria groups. The name of the group is shown in the title of the figure in the top-left corner. Lipids are organized into panels (columns) by their class (from LPC to TG). One colored rectangle corresponds to one lipid species, where the coordinates in the x-axis and y-axis, respectively, show the number of double bonds (N.double.bonds) and the number of carbon atoms (N.carbons) in the fatty acid chains of the lipid. The number of double bonds corresponds to the number of unsaturated bonds in the fatty acid chains, and the number of carbon atoms corresponds to the length of the fatty acid chains and, thus, the size of the lipid. The magnitude of the lipid-specifc model coefficient is shown as the color of the rectangle (blue: negative; red: positive). Coefficient with statistical significance are annotated with symbols #, x and *, respectively, corresponding to $p < 0.01$, $p < 0.05$ and $p < 0.1$.

1.1.2 eGFR

1.1.2.1 Crude Model

1.1.2.1.1 Table

```
##
##
## Table: egfr.intervals
##
## Name                   Coefficient        CI.L        CI.R   AveExpr    P.Value   adj.P.Val
## -------------------  ------------  ----------  ----------  --------  ---------  ----------
## SM(d18:1/24:0)            4.99e-02    0.027900    0.071800      23.5   9.50e-06     0.00101
## SM(d36:2)                -3.95e-02   -0.057600   -0.021400      21.4   2.07e-05     0.00110
## SM(d34:1)                -1.75e-02   -0.026800   -0.008130      24.9   2.59e-04     0.00914
## SM(d40:1)                 3.75e-02    0.016600    0.058400      23.9   4.63e-04     0.01230
## LPC(18:2)                 5.60e-02    0.024000    0.088100      24.0   6.30e-04     0.01340
## TG(18:0/18:1/20:4)       -8.50e-02   -0.136000   -0.034500      21.3   1.01e-03     0.01780
## PC(38:4)                 -2.52e-02   -0.040700   -0.009640      25.2   1.53e-03     0.02320
## SM(d32:1)                -3.35e-02   -0.054500   -0.012600      22.3   1.78e-03     0.02360
## SM(d16:1/18:1)           -2.97e-02   -0.048800   -0.010600      22.8   2.37e-03     0.02790
## PC(32:2)                  5.74e-02    0.019900    0.094900      20.7   2.76e-03     0.02930
## LPC(16:0)                 1.55e-02    0.004800    0.026100      25.3   4.53e-03     0.04370
## PC(O-34:3)                3.81e-02    0.011300    0.064800      21.9   5.34e-03     0.04720
## TG(54:3)                 -3.88e-02   -0.066500   -0.011000      21.7   6.28e-03     0.05120
## SM(d33:1)                -2.98e-02   -0.051500   -0.008060      21.3   7.29e-03     0.05520
## SM(d18:2/24:1)           -2.58e-02   -0.044800   -0.006820      24.0   7.81e-03     0.05520
## TG(53:2)                 -6.54e-02   -0.115000   -0.015700      20.7   1.01e-02     0.06670
## SM(d41:1)                 3.23e-02    0.007470    0.057100      22.7   1.08e-02     0.06760
## TG(52:3)                 -2.53e-02   -0.047600   -0.003000      22.3   2.62e-02     0.14700
## TG(54:4)                 -3.64e-02   -0.068400   -0.004300      21.4   2.63e-02     0.14700
## TG(52:2)                 -2.30e-02   -0.043900   -0.002020      22.6   3.17e-02     0.16600
## TG(53:3)                 -5.03e-02   -0.097100   -0.003530      20.7   3.51e-02     0.16600
## TG(56:3)                 -5.03e-02   -0.097200   -0.003300      20.0   3.60e-02     0.16600
## TG(53:4)                 -5.46e-02   -0.106000   -0.003390      19.7   3.67e-02     0.16600
## TG(18:2/18:1/18:1)       -3.95e-02   -0.077200   -0.001880      24.4   3.96e-02     0.16600
## TG(56:4)                 -5.62e-02   -0.110000   -0.002470      20.0   4.04e-02     0.16600
## TG(52:4)                 -3.46e-02   -0.067800   -0.001460      21.6   4.08e-02     0.16600
## PC(34:2)                 -3.92e-02   -0.077200   -0.001170      26.0   4.34e-02     0.17000
## LPC(18:1)                 2.42e-02    0.000366    0.048000      23.7   4.66e-02     0.17600
## TG(51:2)                 -4.37e-02   -0.088300    0.000787      21.6   5.42e-02     0.19800
## PC(36:5)                  4.60e-02   -0.001160    0.093200      24.3   5.59e-02     0.19800
## TG(54:2)                 -4.29e-02   -0.088600    0.002750      23.4   6.54e-02     0.22400
## TG(45:0)                  1.55e-02   -0.001290    0.032300      23.1   7.03e-02     0.23300
## LPC(16:1)                 2.76e-02   -0.003240    0.058400      20.0   7.93e-02     0.25500
## TG(18:1/18:1/18:1)       -3.19e-02   -0.067900    0.004160      24.8   8.29e-02     0.25800
## TG(56:6)                 -2.72e-02   -0.058700    0.004350      19.9   9.11e-02     0.27300
## LPC(18:0)                 1.63e-02   -0.002830    0.035400      23.9   9.48e-02     0.27300
## TG(18:1/18:1/16:0)       -2.41e-02   -0.052500    0.004360      25.6   9.68e-02     0.27300
## TG(16:0/22:5/18:1)       -3.28e-02   -0.071700    0.006050      22.7   9.78e-02     0.27300
## PC(O-36:5)                1.84e-02   -0.003820    0.040700      22.3   1.04e-01     0.28300
## TG(16:0/18:2/18:2)       -3.66e-02   -0.081400    0.008260      24.5   1.10e-01     0.28900
## PC(34:3)                  3.43e-02   -0.007980    0.076600      22.5   1.12e-01     0.28900
## TG(51:3)                 -3.67e-02   -0.082600    0.009220      20.7   1.17e-01     0.29500
## PC(38:6)                  1.61e-02   -0.004440    0.036700      25.3   1.24e-01     0.30400
## SM(d41:2)                -1.80e-02   -0.041200    0.005090      22.5   1.26e-01     0.30400
## PC(O-34:2)                2.37e-02   -0.008900    0.056300      21.5   1.54e-01     0.36200
## TG(16:0/18:2/18:3)       -4.74e-02   -0.114000    0.018600      22.0   1.59e-01     0.36700
```

```
## PC(32:0)               1.01e-02  -0.004620  0.024800    22.5  1.78e-01    0.40300
## PC(38:2)               1.49e-02  -0.007570  0.037300    21.2  1.94e-01    0.42800
## PC(32:1)               2.73e-02  -0.014500  0.069200    23.3  2.00e-01    0.43100
## TG(50:3)              -2.68e-02  -0.068200  0.014700    23.5  2.06e-01    0.43100
## TG(18:2/18:1/16:0)    -1.62e-02  -0.041700  0.009230    25.3  2.11e-01    0.43100
## TG(52:5)              -3.37e-02  -0.087100  0.019800    19.8  2.16e-01    0.43100
## SM(d38:2)             -1.32e-02  -0.034100  0.007770    21.3  2.17e-01    0.43100
## SM(d36:1)             -1.06e-02  -0.027400  0.006320    23.4  2.20e-01    0.43100
## TG(56:5)              -2.50e-02  -0.065200  0.015300    20.3  2.24e-01    0.43200
## TG(54:5)              -2.39e-02  -0.063100  0.015400    20.6  2.33e-01    0.43600
## SM(d38:1)              1.18e-02  -0.007730  0.031400    23.1  2.35e-01    0.43600
## TG(18:1/18:2/18:2)    -2.78e-02  -0.074200  0.018500    23.6  2.39e-01    0.43600
## TG(51:1)              -2.81e-02  -0.075600  0.019300    20.3  2.45e-01    0.44000
## SM(d40:2)             -1.06e-02  -0.029000  0.007800    23.7  2.58e-01    0.45600
## PC(37:2)               1.93e-02  -0.014700  0.053300    19.3  2.66e-01    0.46200
## TG(14:0/18:1/18:1)    -2.09e-02  -0.059400  0.017600    24.4  2.87e-01    0.49000
## TG(49:2)              -1.72e-02  -0.051400  0.016900    20.4  3.22e-01    0.54000
## TG(49:1)              -1.90e-02  -0.056900  0.018900    20.9  3.26e-01    0.54000
## TG(50:2)              -1.54e-02  -0.047100  0.016300    21.1  3.40e-01    0.55400
## TG(18:1/18:1/22:6)     3.08e-02  -0.044000  0.106000    20.7  4.19e-01    0.67300
## TG(14:0/18:2/18:2)    -2.39e-02  -0.083300  0.035500    21.2  4.30e-01    0.67500
## TG(56:7)              -1.43e-02  -0.050400  0.021800    19.6  4.36e-01    0.67500
## PC(38:5)              -5.68e-03  -0.020100  0.008740    24.6  4.39e-01    0.67500
## PC(O-38:5)            -7.79e-03  -0.027800  0.012300    22.8  4.46e-01    0.67500
## PC(O-36:4)             8.91e-03  -0.014800  0.032600    22.9  4.61e-01    0.68900
## TG(46:0)               1.66e-02  -0.030300  0.063500    20.4  4.88e-01    0.70800
## PC(O-36:3)             1.00e-02  -0.018400  0.038400    20.4  4.88e-01    0.70800
## PC(35:1)              -8.50e-03  -0.033700  0.016700    21.8  5.08e-01    0.72700
## TG(50:1)              -1.63e-02  -0.066100  0.033400    24.3  5.19e-01    0.72700
## TG(47:1)              -1.33e-02  -0.054100  0.027500    19.6  5.21e-01    0.72700
## LPC(20:4)              9.80e-03  -0.021700  0.041300    21.4  5.41e-01    0.74500
## PC(33:1)               8.44e-03  -0.021000  0.037900    20.9  5.74e-01    0.78000
## TG(16:0/18:0/18:1)    -1.75e-02  -0.080000  0.045100    23.1  5.84e-01    0.78300
## PC(34:1)              -4.92e-03  -0.024400  0.014600    26.0  6.20e-01    0.82200
## PC(40:6)               7.53e-03  -0.023500  0.038500    23.9  6.33e-01    0.82900
## PC(36:3)               5.14e-03  -0.017800  0.028000    25.6  6.60e-01    0.85300
## TG(58:9)               1.56e-02  -0.058800  0.090000    19.9  6.81e-01    0.86900
## TG(16:0/18:2/22:6)     1.68e-02  -0.065700  0.099300    21.0  6.90e-01    0.87100
## PC(O-38:6)             4.03e-03  -0.017900  0.026000    20.4  7.18e-01    0.89600
## SM(d39:1)              4.24e-03  -0.024600  0.033100    21.1  7.73e-01    0.94500
## PC(16:0e/18:1(9Z)     -3.23e-03  -0.025500  0.019000    21.8  7.75e-01    0.94500
## PC(36:2)               1.82e-03  -0.012500  0.016100    26.1  8.02e-01    0.94900
## TG(54:6)              -6.60e-03  -0.059300  0.046100    19.5  8.06e-01    0.94900
## TG(18:1/12:0/18:1)    -6.27e-03  -0.056300  0.043800    22.5  8.06e-01    0.94900
## TG(48:3)              -7.66e-03  -0.074700  0.059400    20.5  8.23e-01    0.95500
## TG(46:2)              -5.66e-03  -0.059000  0.047700    20.1  8.35e-01    0.95500
## PC(O-36:2)            -2.65e-03  -0.028100  0.022800    20.5  8.38e-01    0.95500
## PC(40:5)               2.60e-03  -0.024500  0.029700    22.0  8.50e-01    0.95900
## PC(40:7)               2.19e-03  -0.028100  0.032400    21.4  8.87e-01    0.98700
## TG(14:0/16:0/18:1)     3.42e-03  -0.046800  0.053700    22.8  8.94e-01    0.98700
## PC(O-38:4)            -1.34e-03  -0.028000  0.025400    21.7  9.22e-01    0.99900
## TG(50:0)              -1.95e-03  -0.058700  0.054800    20.8  9.46e-01    0.99900
## SM(42:2)              -4.21e-04  -0.016800  0.016000    25.2  9.60e-01    0.99900
## PC(35:2)              -6.49e-04  -0.027700  0.026400    21.8  9.63e-01    0.99900
```

```
## PC(38:3)             -5.76e-04   -0.025300    0.024100      24.0   9.63e-01     0.99900
## TG(49:3)             -4.00e-05   -0.005670    0.005590      20.7   9.89e-01     0.99900
## PC(36:4)              6.80e-05   -0.012600    0.012700      25.9   9.92e-01     0.99900
## TG(46:1)              2.32e-04   -0.049500    0.050000      21.3   9.93e-01     0.99900
## TG(18:2/22:5/16:0)   -2.18e-04   -0.064100    0.063700      22.1   9.95e-01     0.99900
## TG(18:2/18:2/18:2)    2.08e-05   -0.051000    0.051000      22.1   9.99e-01     0.99900
##
##
## Table: Results of the crude model for eGFR variable.
## Shown in the table are name of the lipid (Name),
## model coefficient (Coefficient),
## its lower and upper confidence intervals (CI.L, CL.R),
## average value of the variable (AveExpr),
## p-value of the coefficient (P.Value) and
## the p-value after correction for multiple testing (adj.P.Val).
## The rows of the table are shown in an increasing order by the respective p-value.
##
## Name                   Coefficient        CI.L        CI.R   AveExpr    P.Value   adj.P.Val
## -------------------  ------------  ----------  ----------  --------  ---------  ----------
## SM(d18:1/24:0)           4.99e-02    0.027900    0.071800      23.5   9.50e-06     0.00101
## SM(d36:2)               -3.95e-02   -0.057600   -0.021400      21.4   2.07e-05     0.00110
## SM(d34:1)               -1.75e-02   -0.026800   -0.008130      24.9   2.59e-04     0.00914
## SM(d40:1)                3.75e-02    0.016600    0.058400      23.9   4.63e-04     0.01230
## LPC(18:2)                5.60e-02    0.024000    0.088100      24.0   6.30e-04     0.01340
## TG(18:0/18:1/20:4)      -8.50e-02   -0.136000   -0.034500      21.3   1.01e-03     0.01780
## PC(38:4)                -2.52e-02   -0.040700   -0.009640      25.2   1.53e-03     0.02320
## SM(d32:1)               -3.35e-02   -0.054500   -0.012600      22.3   1.78e-03     0.02360
## SM(d16:1/18:1)          -2.97e-02   -0.048800   -0.010600      22.8   2.37e-03     0.02790
## PC(32:2)                 5.74e-02    0.019900    0.094900      20.7   2.76e-03     0.02930
## LPC(16:0)                1.55e-02    0.004800    0.026100      25.3   4.53e-03     0.04370
## PC(O-34:3)               3.81e-02    0.011300    0.064800      21.9   5.34e-03     0.04720
## TG(54:3)                -3.88e-02   -0.066500   -0.011000      21.7   6.28e-03     0.05120
## SM(d33:1)               -2.98e-02   -0.051500   -0.008060      21.3   7.29e-03     0.05520
## SM(d18:2/24:1)          -2.58e-02   -0.044800   -0.006820      24.0   7.81e-03     0.05520
## TG(53:2)                -6.54e-02   -0.115000   -0.015700      20.7   1.01e-02     0.06670
## SM(d41:1)                3.23e-02    0.007470    0.057100      22.7   1.08e-02     0.06760
## TG(52:3)                -2.53e-02   -0.047600   -0.003000      22.3   2.62e-02     0.14700
## TG(54:4)                -3.64e-02   -0.068400   -0.004300      21.4   2.63e-02     0.14700
## TG(52:2)                -2.30e-02   -0.043900   -0.002020      22.6   3.17e-02     0.16600
## TG(53:3)                -5.03e-02   -0.097100   -0.003530      20.7   3.51e-02     0.16600
## TG(56:3)                -5.03e-02   -0.097200   -0.003300      20.0   3.60e-02     0.16600
## TG(53:4)                -5.46e-02   -0.106000   -0.003390      19.7   3.67e-02     0.16600
## TG(18:2/18:1/18:1)      -3.95e-02   -0.077200   -0.001880      24.4   3.96e-02     0.16600
## TG(56:4)                -5.62e-02   -0.110000   -0.002470      20.0   4.04e-02     0.16600
## TG(52:4)                -3.46e-02   -0.067800   -0.001460      21.6   4.08e-02     0.16600
## PC(34:2)                -3.92e-02   -0.077200   -0.001170      26.0   4.34e-02     0.17000
## LPC(18:1)                2.42e-02    0.000366    0.048000      23.7   4.66e-02     0.17600
## TG(51:2)                -4.37e-02   -0.088300    0.000787      21.6   5.42e-02     0.19800
## PC(36:5)                 4.60e-02   -0.001160    0.093200      24.3   5.59e-02     0.19800
## TG(54:2)                -4.29e-02   -0.088600    0.002750      23.4   6.54e-02     0.22400
## TG(45:0)                 1.55e-02   -0.001290    0.032300      23.1   7.03e-02     0.23300
## LPC(16:1)                2.76e-02   -0.003240    0.058400      20.0   7.93e-02     0.25500
## TG(18:1/18:1/18:1)      -3.19e-02   -0.067900    0.004160      24.8   8.29e-02     0.25800
## TG(56:6)                -2.72e-02   -0.058700    0.004350      19.9   9.11e-02     0.27300
```

```
## LPC(18:0)              1.63e-02   -0.002830    0.035400       23.9   9.48e-02     0.27300
## TG(18:1/18:1/16:0)    -2.41e-02   -0.052500    0.004360       25.6   9.68e-02     0.27300
## TG(16:0/22:5/18:1)    -3.28e-02   -0.071700    0.006050       22.7   9.78e-02     0.27300
## PC(O-36:5)             1.84e-02   -0.003820    0.040700       22.3   1.04e-01     0.28300
## TG(16:0/18:2/18:2)    -3.66e-02   -0.081400    0.008260       24.5   1.10e-01     0.28900
## PC(34:3)               3.43e-02   -0.007980    0.076600       22.5   1.12e-01     0.28900
## TG(51:3)              -3.67e-02   -0.082600    0.009220       20.7   1.17e-01     0.29500
## PC(38:6)               1.61e-02   -0.004440    0.036700       25.3   1.24e-01     0.30400
## SM(d41:2)             -1.80e-02   -0.041200    0.005090       22.5   1.26e-01     0.30400
## PC(O-34:2)             2.37e-02   -0.008900    0.056300       21.5   1.54e-01     0.36200
## TG(16:0/18:2/18:3)    -4.74e-02   -0.114000    0.018600       22.0   1.59e-01     0.36700
## PC(32:0)               1.01e-02   -0.004620    0.024800       22.5   1.78e-01     0.40300
## PC(38:2)               1.49e-02   -0.007570    0.037300       21.2   1.94e-01     0.42800
## PC(32:1)               2.73e-02   -0.014500    0.069200       23.3   2.00e-01     0.43100
## TG(50:3)              -2.68e-02   -0.068200    0.014700       23.5   2.06e-01     0.43100
## TG(18:2/18:1/16:0)    -1.62e-02   -0.041700    0.009230       25.3   2.11e-01     0.43100
## TG(52:5)              -3.37e-02   -0.087100    0.019800       19.8   2.16e-01     0.43100
## SM(d38:2)             -1.32e-02   -0.034100    0.007770       21.3   2.17e-01     0.43100
## SM(d36:1)             -1.06e-02   -0.027400    0.006320       23.4   2.20e-01     0.43100
## TG(56:5)              -2.50e-02   -0.065200    0.015300       20.3   2.24e-01     0.43200
## TG(54:5)              -2.39e-02   -0.063100    0.015400       20.6   2.33e-01     0.43600
## SM(d38:1)              1.18e-02   -0.007730    0.031400       23.1   2.35e-01     0.43600
## TG(18:1/18:2/18:2)    -2.78e-02   -0.074200    0.018500       23.6   2.39e-01     0.43600
## TG(51:1)              -2.81e-02   -0.075600    0.019300       20.3   2.45e-01     0.44000
## SM(d40:2)             -1.06e-02   -0.029000    0.007800       23.7   2.58e-01     0.45600
## PC(37:2)               1.93e-02   -0.014700    0.053300       19.3   2.66e-01     0.46200
## TG(14:0/18:1/18:1)    -2.09e-02   -0.059400    0.017600       24.4   2.87e-01     0.49000
## TG(49:2)              -1.72e-02   -0.051400    0.016900       20.4   3.22e-01     0.54000
## TG(49:1)              -1.90e-02   -0.056900    0.018900       20.9   3.26e-01     0.54000
## TG(50:2)              -1.54e-02   -0.047100    0.016300       21.1   3.40e-01     0.55400
## TG(18:1/18:1/22:6)     3.08e-02   -0.044000    0.106000       20.7   4.19e-01     0.67300
## TG(14:0/18:2/18:2)    -2.39e-02   -0.083300    0.035500       21.2   4.30e-01     0.67500
## TG(56:7)              -1.43e-02   -0.050400    0.021800       19.6   4.36e-01     0.67500
## PC(38:5)              -5.68e-03   -0.020100    0.008740       24.6   4.39e-01     0.67500
## PC(O-38:5)            -7.79e-03   -0.027800    0.012300       22.8   4.46e-01     0.67500
## PC(O-36:4)             8.91e-03   -0.014800    0.032600       22.9   4.61e-01     0.68900
## TG(46:0)               1.66e-02   -0.030300    0.063500       20.4   4.88e-01     0.70800
## PC(O-36:3)             1.00e-02   -0.018400    0.038400       20.4   4.88e-01     0.70800
## PC(35:1)              -8.50e-03   -0.033700    0.016700       21.8   5.08e-01     0.72700
## TG(50:1)              -1.63e-02   -0.066100    0.033400       24.3   5.19e-01     0.72700
## TG(47:1)              -1.33e-02   -0.054100    0.027500       19.6   5.21e-01     0.72700
## LPC(20:4)              9.80e-03   -0.021700    0.041300       21.4   5.41e-01     0.74500
## PC(33:1)               8.44e-03   -0.021000    0.037900       20.9   5.74e-01     0.78000
## TG(16:0/18:0/18:1)    -1.75e-02   -0.080000    0.045100       23.1   5.84e-01     0.78300
## PC(34:1)              -4.92e-03   -0.024400    0.014600       26.0   6.20e-01     0.82200
## PC(40:6)               7.53e-03   -0.023500    0.038500       23.9   6.33e-01     0.82900
## PC(36:3)               5.14e-03   -0.017800    0.028000       25.6   6.60e-01     0.85300
## TG(58:9)               1.56e-02   -0.058800    0.090000       19.9   6.81e-01     0.86900
## TG(16:0/18:2/22:6)     1.68e-02   -0.065700    0.099300       21.0   6.90e-01     0.87100
## PC(O-38:6)             4.03e-03   -0.017900    0.026000       20.4   7.18e-01     0.89600
## SM(d39:1)              4.24e-03   -0.024600    0.033100       21.1   7.73e-01     0.94500
## PC(16:0e/18:1(9Z)     -3.23e-03   -0.025500    0.019000       21.8   7.75e-01     0.94500
## PC(36:2)               1.82e-03   -0.012500    0.016100       26.1   8.02e-01     0.94900
## TG(54:6)              -6.60e-03   -0.059300    0.046100       19.5   8.06e-01     0.94900
```

## TG(18:1/12:0/18:1)	-6.27e-03	-0.056300	0.043800	22.5	8.06e-01	0.94900
## TG(48:3)	-7.66e-03	-0.074700	0.059400	20.5	8.23e-01	0.95500
## TG(46:2)	-5.66e-03	-0.059000	0.047700	20.1	8.35e-01	0.95500
## PC(O-36:2)	-2.65e-03	-0.028100	0.022800	20.5	8.38e-01	0.95500
## PC(40:5)	2.60e-03	-0.024500	0.029700	22.0	8.50e-01	0.95900
## PC(40:7)	2.19e-03	-0.028100	0.032400	21.4	8.87e-01	0.98700
## TG(14:0/16:0/18:1)	3.42e-03	-0.046800	0.053700	22.8	8.94e-01	0.98700
## PC(O-38:4)	-1.34e-03	-0.028000	0.025400	21.7	9.22e-01	0.99900
## TG(50:0)	-1.95e-03	-0.058700	0.054800	20.8	9.46e-01	0.99900
## SM(42:2)	-4.21e-04	-0.016800	0.016000	25.2	9.60e-01	0.99900
## PC(35:2)	-6.49e-04	-0.027700	0.026400	21.8	9.63e-01	0.99900
## PC(38:3)	-5.76e-04	-0.025300	0.024100	24.0	9.63e-01	0.99900
## TG(49:3)	-4.00e-05	-0.005670	0.005590	20.7	9.89e-01	0.99900
## PC(36:4)	6.80e-05	-0.012600	0.012700	25.9	9.92e-01	0.99900
## TG(46:1)	2.32e-04	-0.049500	0.050000	21.3	9.93e-01	0.99900
## TG(18:2/22:5/16:0)	-2.18e-04	-0.064100	0.063700	22.1	9.95e-01	0.99900
## TG(18:2/18:2/18:2)	2.08e-05	-0.051000	0.051000	22.1	9.99e-01	0.99900

1.1.2.1.2 Heatmaps

```
##   Label Relation p.adj
## 1     #  p.adj <  0.01
## 2     x  p.adj <  0.05
## 3     *  p.adj <  0.10
```

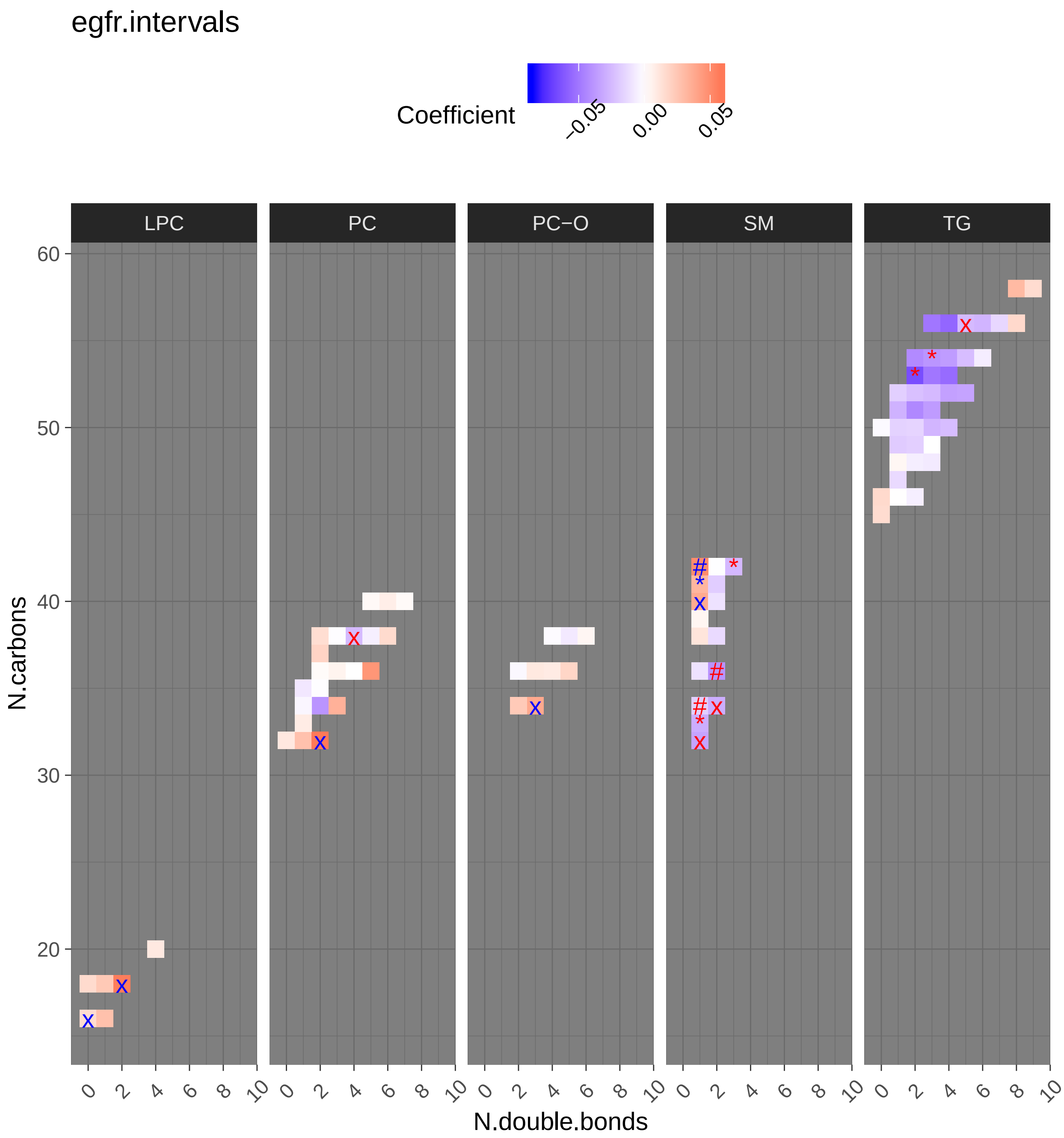

Figure 7: Heatmap of the lipid-specific model coefficents from the crude model for eGFR. Lipids are organized into panels (columns) by their class (from LPC to TG). One colored rectangle corresponds to one lipid species, where the coordinates in the x-axis and y-axis, respectively, show the number of double bonds (N.double.bonds) and the number of carbon atoms (N.carbons) in the fatty acid chains of the lipid. The number of double bonds corresponds to the number of unsaturated bonds in the fatty acid chains, and the number of carbon atoms corresponds to the length of the fatty acid chains and, thus, the size of the lipid. The magnitude of the lipid-specifc model coefficient is shown as the color of the rectangle (blue: negative; red: positive). Coefficient with statistical significance are annotated with symbols #, x and *, respectively, corresponding to $p < 0.01$, $p < 0.05$ and $p < 0.1$.

1.1.2.2 Adjusted Model

1.1.2.2.1 Table

```
##
##
## Table: Results of the adjustred model for eGFR variable.
## Shown in the table are name of the lipid (Name),
## model coefficient (Coefficient),
## its lower and upper confidence intervals (CI.L, CL.R),
## average value of the variable (AveExpr),
## p-value of the coefficient (P.Value) and
## the p-value after correction for multiple testing (adj.P.Val).
## The rows of the table are shown in an increasing order by the respective p-value.
##
## Name                   Coefficient         CI.L         CI.R   AveExpr    P.Value   adj.P.Val
## -------------------  ------------  ----------  ----------  --------  ---------  ----------
## PC(36:5)                 0.106000    0.059400    0.153000      24.3   1.05e-05     0.00112
## PC(38:6)                 0.041800    0.021400    0.062100      25.3   6.17e-05     0.00327
## TG(18:1/18:1/22:6)       0.154000    0.075800    0.233000      20.7   1.25e-04     0.00442
## SM(d34:1)               -0.017800   -0.027300   -0.008350      24.9   2.39e-04     0.00633
## PC(40:6)                 0.053700    0.023800    0.083600      23.9   4.49e-04     0.00951
## SM(d33:1)               -0.035100   -0.056200   -0.014000      21.3   1.16e-03     0.01920
## TG(18:2/18:2/18:2)       0.074600    0.029400    0.120000      22.1   1.27e-03     0.01920
## PC(32:2)                 0.058400    0.020400    0.096500      20.7   2.66e-03     0.03070
## TG(18:2/22:5/16:0)       0.095600    0.033200    0.158000      22.1   2.74e-03     0.03070
## TG(16:0/18:2/22:6)       0.128000    0.043500    0.213000      21.0   3.08e-03     0.03070
## SM(d16:1/18:1)          -0.023000   -0.038300   -0.007750      22.8   3.19e-03     0.03070
## TG(58:9)                 0.117000    0.036800    0.196000      19.9   4.25e-03     0.03760
## PC(O-36:2)              -0.037000   -0.062700   -0.011300      20.5   4.81e-03     0.03920
## PC(32:1)                 0.061200    0.018300    0.104000      23.3   5.24e-03     0.03960
## LPC(16:1)                0.046500    0.012800    0.080300      20.0   6.91e-03     0.04880
## PC(40:7)                 0.040600    0.010600    0.070600      21.4   8.02e-03     0.05320
## TG(14:0/16:0/18:1)       0.052500    0.013200    0.091800      22.8   8.93e-03     0.05570
## PC(O-38:4)              -0.037400   -0.066500   -0.008220      21.7   1.21e-02     0.07030
## SM(d18:2/24:1)          -0.022500   -0.040300   -0.004800      24.0   1.28e-02     0.07030
## SM(d32:1)               -0.024700   -0.044300   -0.005180      22.3   1.33e-02     0.07030
## LPC(16:0)                0.013100    0.002090    0.024100      25.3   1.98e-02     0.09980
## TG(46:0)                 0.049100    0.006210    0.091900      20.4   2.49e-02     0.12000
## PC(40:5)                 0.029400    0.003180    0.055700      22.0   2.81e-02     0.12500
## PC(O-38:5)              -0.024600   -0.046600   -0.002610      22.8   2.84e-02     0.12500
## TG(18:1/12:0/18:1)       0.040200    0.002350    0.078100      22.5   3.74e-02     0.15200
## TG(54:3)                -0.022600   -0.044000   -0.001290      21.7   3.77e-02     0.15200
## SM(d36:2)               -0.018800   -0.036600   -0.000984      21.4   3.86e-02     0.15200
## SM(d41:2)               -0.022700   -0.044600   -0.000879      22.5   4.15e-02     0.15700
## PC(16:0e/18:1(9Z)       -0.022100   -0.043700   -0.000456      21.8   4.54e-02     0.16600
## TG(54:6)                 0.049500    0.000222    0.098800      19.5   4.90e-02     0.17300
## SM(d40:2)               -0.015800   -0.032000    0.000265      23.7   5.39e-02     0.17700
## TG(54:4)                -0.027900   -0.056300    0.000512      21.4   5.43e-02     0.17700
## SM(d18:1/24:0)           0.019000   -0.000487    0.038500      23.5   5.60e-02     0.17700
## TG(14:0/18:2/18:2)       0.041000   -0.001180    0.083200      21.2   5.67e-02     0.17700
## TG(49:3)                -0.004000   -0.008450    0.000451      20.7   7.81e-02     0.23700
## PC(38:4)                -0.014600   -0.030900    0.001830      25.2   8.15e-02     0.24000
## TG(48:3)                 0.048200   -0.006770    0.103000      20.5   8.56e-02     0.24500
## TG(46:2)                 0.037200   -0.007500    0.081800      20.1   1.03e-01     0.27800
## TG(14:0/18:1/18:1)       0.020600   -0.004450    0.045700      24.4   1.07e-01     0.27800
```

```
## TG(50:2)               0.017400  -0.003920  0.038800     21.1  1.09e-01    0.27800
## PC(38:2)               0.017700  -0.003980  0.039300     21.2  1.10e-01    0.27800
## PC(O-36:3)            -0.021900  -0.048800  0.004980     20.4  1.10e-01    0.27800
## TG(50:3)               0.019400  -0.004770  0.043500     23.5  1.16e-01    0.28500
## TG(18:0/18:1/20:4)    -0.037700  -0.087500  0.012200     21.3  1.39e-01    0.33400
## TG(56:7)               0.026500  -0.009270  0.062300     19.6  1.46e-01    0.34000
## TG(46:1)               0.032600  -0.011600  0.076800     21.3  1.48e-01    0.34000
## TG(52:4)              -0.019100  -0.045500  0.007360     21.6  1.57e-01    0.35100
## TG(52:3)              -0.011900  -0.028500  0.004680     22.3  1.59e-01    0.35100
## TG(50:1)               0.025900  -0.012800  0.064500     24.3  1.89e-01    0.40300
## TG(18:2/18:1/18:1)    -0.021700  -0.054300  0.011000     24.4  1.93e-01    0.40300
## PC(32:0)               0.009630  -0.005010  0.024300     22.5  1.97e-01    0.40300
## PC(36:2)              -0.010600  -0.026900  0.005590     26.1  1.99e-01    0.40300
## TG(50:0)               0.032100  -0.017200  0.081300     20.8  2.02e-01    0.40300
## PC(35:2)              -0.017800  -0.045800  0.010200     21.8  2.13e-01    0.41900
## TG(16:0/18:0/18:1)     0.030600  -0.018400  0.079700     23.1  2.21e-01    0.42600
## PC(O-34:2)            -0.019400  -0.053600  0.014700     21.5  2.64e-01    0.50100
## TG(56:5)               0.017400  -0.015500  0.050200     20.3  3.00e-01    0.55900
## SM(d40:1)              0.009320  -0.008600  0.027200     23.9  3.08e-01    0.56200
## TG(53:2)              -0.017800  -0.053700  0.018000     20.7  3.29e-01    0.59000
## PC(38:3)               0.011000  -0.012700  0.034800     24.0  3.63e-01    0.63300
## PC(33:1)               0.013300  -0.015500  0.042100     20.9  3.64e-01    0.63300
## LPC(18:0)              0.008960  -0.011200  0.029100     23.9  3.83e-01    0.64500
## PC(34:2)              -0.018500  -0.060700  0.023700     26.0  3.89e-01    0.64500
## SM(d38:2)             -0.008840  -0.029000  0.011300     21.3  3.90e-01    0.64500
## PC(34:3)               0.018300  -0.025000  0.061500     22.5  4.06e-01    0.66300
## PC(O-36:4)            -0.009480  -0.035700  0.016700     22.9  4.77e-01    0.76300
## TG(52:2)              -0.004980  -0.019000  0.009050     22.6  4.86e-01    0.76300
## SM(d41:1)              0.007760  -0.015100  0.030700     22.7  5.06e-01    0.76300
## TG(54:5)              -0.012000  -0.048000  0.024000     20.6  5.14e-01    0.76300
## LPC(18:1)              0.008550  -0.017300  0.034400     23.7  5.16e-01    0.76300
## SM(d36:1)             -0.005100  -0.020700  0.010500     23.4  5.20e-01    0.76300
## TG(53:4)              -0.013200  -0.055900  0.029500     19.7  5.44e-01    0.76300
## TG(51:1)               0.010900  -0.024800  0.046500     20.3  5.50e-01    0.76300
## SM(d39:1)             -0.008770  -0.037700  0.020200     21.1  5.52e-01    0.76300
## TG(49:2)               0.008660  -0.020000  0.037300     20.4  5.54e-01    0.76300
## LPC(18:2)              0.010100  -0.024600  0.044800     24.0  5.68e-01    0.76300
## TG(16:0/22:5/18:1)     0.009550  -0.023600  0.042700     22.7  5.71e-01    0.76300
## TG(56:3)              -0.010500  -0.047000  0.026000     20.0  5.72e-01    0.76300
## TG(56:4)              -0.012500  -0.056200  0.031200     20.0  5.74e-01    0.76300
## TG(18:1/18:1/18:1)    -0.008830  -0.039800  0.022200     24.8  5.76e-01    0.76300
## SM(42:2)              -0.004040  -0.019200  0.011100     25.2  6.01e-01    0.78600
## TG(45:0)               0.002500  -0.008120  0.013100     23.1  6.45e-01    0.83300
## PC(36:3)               0.005020  -0.019100  0.029200     25.6  6.83e-01    0.87200
## TG(53:3)              -0.006790  -0.041900  0.028300     20.7  7.04e-01    0.88900
## TG(54:2)              -0.005460  -0.035700  0.024700     23.4  7.23e-01    0.89900
## TG(56:6)               0.005100  -0.023900  0.034100     19.9  7.30e-01    0.89900
## PC(O-38:6)            -0.003680  -0.026300  0.018900     20.4  7.49e-01    0.89900
## TG(18:2/18:1/16:0)     0.004320  -0.023200  0.031800     25.3  7.58e-01    0.89900
## SM(d38:1)              0.002780  -0.015100  0.020600     23.1  7.60e-01    0.89900
## TG(16:0/18:2/18:2)    -0.004980  -0.040000  0.030000     24.5  7.80e-01    0.89900
## TG(18:1/18:1/16:0)    -0.004410  -0.035400  0.026600     25.6  7.80e-01    0.89900
## PC(O-36:5)             0.003480  -0.021000  0.028000     22.3  7.81e-01    0.89900
## PC(38:5)               0.001830  -0.012800  0.016500     24.6  8.06e-01    0.90900
```

```
## TG(49:1)                0.004060   -0.028500   0.036600     20.9   8.07e-01     0.90900
## TG(18:1/18:2/18:2)     -0.003850   -0.044700   0.037000     23.6   8.53e-01     0.95200
## TG(51:3)                0.002930   -0.031600   0.037400     20.7   8.68e-01     0.95300
## TG(51:2)                0.002370   -0.027000   0.031800     21.6   8.74e-01     0.95300
## PC(35:1)               -0.001730   -0.026500   0.023000     21.8   8.91e-01     0.95300
## PC(36:4)                0.000956   -0.013300   0.015300     25.9   8.96e-01     0.95300
## TG(16:0/18:2/18:3)      0.003450   -0.054300   0.061200     22.0   9.07e-01     0.95300
## PC(34:1)               -0.001310   -0.023600   0.021000     26.0   9.08e-01     0.95300
## LPC(20:4)              -0.001550   -0.036000   0.032900     21.4   9.30e-01     0.96600
## PC(37:2)               -0.001130   -0.035400   0.033200     19.3   9.48e-01     0.97100
## TG(47:1)                0.001390   -0.044700   0.047500     19.6   9.53e-01     0.97100
## PC(O-34:3)              0.000326   -0.025000   0.025700     21.9   9.80e-01     0.98400
## TG(52:5)                0.000486   -0.048400   0.049400     19.8   9.84e-01     0.98400
```

1.1.2.2.2 Heatmaps

```
## [1] "map_lipid_names has been created by Tommi Suvitaival"
## [1] "tommi.raimo.leo.suvitaival@regionh.dk"
## [1] "2019-05-06"
```

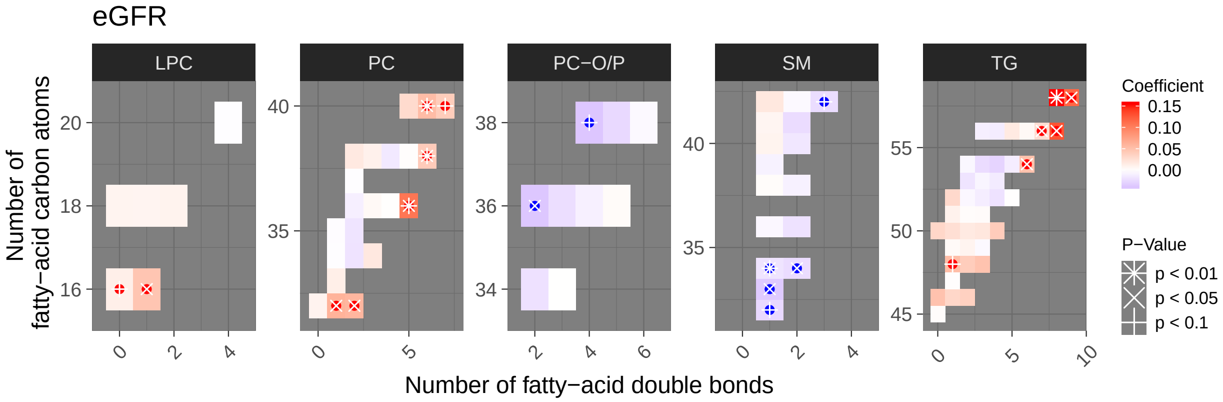

1.1.3 logUAER – Continuous Albuminuria

1.1.3.1 Crude Model

1.1.3.1.1 Table

```
##
##
## Table: Results of the crude model for continuous albuminuria variable (logUAER).
## Shown in the table are name of the lipid (Name),
## model coefficient (Coefficient),
## its lower and upper confidence intervals (CI.L, CL.R),
## average value of the variable (AveExpr),
## p-value of the coefficient (P.Value) and
## the p-value after correction for multiple testing (adj.P.Val).
## The rows of the table are shown in an increasing order by the respective p-value.
##
## Name                    Coefficient        CI.L        CI.R   AveExpr    P.Value   adj.P.Val
## -------------------    ------------  ----------  ----------  --------  ---------  ----------
## PC(O-34:3)                -0.047000   -6.39e-02   -0.030000      21.9   1.00e-07    7.90e-06
## PC(O-36:3)                -0.041900   -5.99e-02   -0.023900      20.4   5.80e-06    3.09e-04
## PC(O-38:6)                -0.026600   -4.06e-02   -0.012600      20.4   2.03e-04    5.62e-03
## PC(O-36:2)                -0.030800   -4.70e-02   -0.014600      20.5   2.12e-04    5.62e-03
## TG(53:2)                   0.058900    2.70e-02    0.090800      20.7   3.13e-04    5.65e-03
## TG(45:0)                  -0.019700   -3.05e-02   -0.008970      23.1   3.39e-04    5.65e-03
## TG(52:2)                   0.024400    1.10e-02    0.037800      22.6   3.73e-04    5.65e-03
## TG(51:2)                   0.048400    1.99e-02    0.077000      21.6   9.03e-04    1.10e-02
## TG(56:3)                   0.051000    2.09e-02    0.081100      20.0   9.38e-04    1.10e-02
## SM(d18:1/24:0)            -0.023500   -3.77e-02   -0.009240      23.5   1.27e-03    1.29e-02
## TG(54:2)                   0.048000    1.88e-02    0.077300      23.4   1.34e-03    1.29e-02
## PC(O-34:2)                -0.033900   -5.48e-02   -0.013100      21.5   1.48e-03    1.31e-02
## PC(16:0e/18:1(9Z)         -0.022200   -3.64e-02   -0.007930      21.8   2.32e-03    1.89e-02
## TG(53:3)                   0.045500    1.55e-02    0.075500      20.7   3.05e-03    2.31e-02
## SM(d41:1)                 -0.023200   -3.92e-02   -0.007250      22.7   4.43e-03    3.13e-02
## SM(d40:1)                 -0.018800   -3.24e-02   -0.005290      23.9   6.48e-03    4.27e-02
## TG(56:4)                   0.047700    1.32e-02    0.082200      20.0   6.85e-03    4.27e-02
## TG(54:3)                   0.024300    6.45e-03    0.042200      21.7   7.73e-03    4.55e-02
## TG(50:2)                   0.026900    6.59e-03    0.047200      21.1   9.51e-03    4.99e-02
## TG(14:0/18:1/18:1)         0.032500    7.84e-03    0.057200      24.4   9.89e-03    4.99e-02
## TG(50:1)                   0.041900    1.01e-02    0.073800      24.3   1.00e-02    4.99e-02
## TG(56:6)                   0.026300    6.07e-03    0.046600      19.9   1.09e-02    4.99e-02
## PC(32:0)                  -0.012200   -2.17e-02   -0.002800      22.5   1.11e-02    4.99e-02
## TG(51:1)                   0.039400    8.95e-03    0.069800      20.3   1.13e-02    4.99e-02
## TG(52:3)                   0.018200    3.86e-03    0.032500      22.3   1.29e-02    5.30e-02
## TG(16:0/22:5/18:1)         0.031700    6.71e-03    0.056700      22.7   1.30e-02    5.30e-02
## TG(16:0/18:0/18:1)         0.050300    1.01e-02    0.090400      23.1   1.41e-02    5.55e-02
## TG(52:4)                   0.026500    5.17e-03    0.047900      21.6   1.50e-02    5.64e-02
## TG(54:6)                   0.041800    8.00e-03    0.075600      19.5   1.54e-02    5.64e-02
## TG(51:3)                   0.036000    6.45e-03    0.065500      20.7   1.70e-02    6.01e-02
## TG(18:1/18:1/16:0)         0.022000    3.71e-03    0.040300      25.6   1.85e-02    6.32e-02
## TG(56:5)                   0.030700    4.84e-03    0.056600      20.3   2.01e-02    6.65e-02
## PC(O-36:5)                -0.016300   -3.06e-02   -0.001960      22.3   2.59e-02    8.32e-02
## TG(50:3)                   0.029700    3.09e-03    0.056400      23.5   2.88e-02    8.98e-02
## TG(18:2/18:2/18:2)         0.035900    3.21e-03    0.068700      22.1   3.15e-02    9.52e-02
## PC(32:2)                  -0.026500   -5.08e-02   -0.002240      20.7   3.24e-02    9.53e-02
## PC(40:7)                  -0.020300   -3.98e-02   -0.000904      21.4   4.03e-02    1.15e-01
## LPC(18:2)                 -0.021600   -4.23e-02   -0.000798      24.0   4.18e-02    1.17e-01
## PC(34:2)                   0.024400   -9.28e-05    0.048900      26.0   5.09e-02    1.37e-01
```

```
## TG(18:2/22:5/16:0)      0.040700  -3.06e-04  0.081800     22.1  5.17e-02   1.37e-01
## TG(16:0/18:2/18:2)      0.028300  -5.36e-04  0.057200     24.5  5.44e-02   1.41e-01
## TG(18:1/18:1/18:1)      0.022400  -7.57e-04  0.045600     24.8  5.79e-02   1.44e-01
## TG(49:1)                0.023500  -8.22e-04  0.047900     20.9  5.82e-02   1.44e-01
## TG(53:4)                0.030900  -2.06e-03  0.063900     19.7  6.61e-02   1.57e-01
## TG(14:0/16:0/18:1)      0.030200  -2.09e-03  0.062500     22.8  6.67e-02   1.57e-01
## TG(18:0/18:1/20:4)      0.030400  -2.37e-03  0.063200     21.3  6.90e-02   1.59e-01
## TG(56:7)                0.021200  -1.97e-03  0.044500     19.6  7.28e-02   1.64e-01
## TG(49:3)               -0.003280  -6.89e-03  0.000341     20.7  7.58e-02   1.67e-01
## PC(37:2)               -0.019400  -4.13e-02  0.002520     19.3  8.27e-02   1.78e-01
## TG(16:0/18:2/18:3)      0.037500  -5.06e-03  0.080100     22.0  8.41e-02   1.78e-01
## TG(52:5)                0.029600  -4.75e-03  0.064000     19.8  9.11e-02   1.89e-01
## SM(d39:1)              -0.015900  -3.45e-02  0.002650     21.1  9.28e-02   1.89e-01
## TG(14:0/18:2/18:2)      0.032500  -5.69e-03  0.070700     21.2  9.52e-02   1.89e-01
## TG(18:1/12:0/18:1)      0.027300  -4.87e-03  0.059500     22.5  9.61e-02   1.89e-01
## PC(38:4)                0.008360  -1.71e-03  0.018400     25.2  1.04e-01   2.00e-01
## PC(38:6)               -0.010900  -2.42e-02  0.002350     25.3  1.07e-01   2.02e-01
## TG(18:2/18:1/18:1)      0.019200  -5.06e-03  0.043500     24.4  1.20e-01   2.24e-01
## TG(49:2)                0.017000  -5.01e-03  0.038900     20.4  1.30e-01   2.38e-01
## TG(54:4)                0.014900  -5.80e-03  0.035600     21.4  1.58e-01   2.83e-01
## SM(d41:2)              -0.010400  -2.53e-02  0.004500     22.5  1.71e-01   3.00e-01
## SM(d34:1)               0.004200  -1.87e-03  0.010300     24.9  1.75e-01   3.00e-01
## PC(35:2)               -0.012000  -2.94e-02  0.005430     21.8  1.77e-01   3.00e-01
## SM(d38:2)              -0.009200  -2.27e-02  0.004300     21.3  1.81e-01   3.00e-01
## PC(36:5)               -0.020800  -5.12e-02  0.009700     24.3  1.81e-01   3.00e-01
## PC(36:2)               -0.006110  -1.53e-02  0.003070     26.1  1.92e-01   3.13e-01
## TG(46:2)                0.022200  -1.21e-02  0.056600     20.1  2.04e-01   3.28e-01
## SM(d38:1)              -0.007900  -2.05e-02  0.004710     23.1  2.19e-01   3.45e-01
## LPC(20:4)               0.012600  -7.63e-03  0.032900     21.4  2.21e-01   3.45e-01
## SM(d40:2)              -0.007170  -1.90e-02  0.004690     23.7  2.36e-01   3.62e-01
## TG(50:0)                0.021500  -1.50e-02  0.058000     20.8  2.47e-01   3.72e-01
## TG(18:1/18:1/22:6)      0.028200  -2.00e-02  0.076300     20.7  2.51e-01   3.72e-01
## TG(54:5)                0.014700  -1.05e-02  0.040000     20.6  2.52e-01   3.72e-01
## PC(38:3)                0.009080  -6.82e-03  0.025000     24.0  2.63e-01   3.81e-01
## PC(40:6)               -0.011200  -3.11e-02  0.008770     23.9  2.72e-01   3.89e-01
## TG(48:3)                0.023700  -1.94e-02  0.066900     20.5  2.81e-01   3.95e-01
## TG(46:1)                0.017500  -1.45e-02  0.049500     21.3  2.83e-01   3.95e-01
## SM(d32:1)               0.007230  -6.39e-03  0.020800     22.3  2.97e-01   4.07e-01
## TG(18:1/18:2/18:2)      0.015800  -1.41e-02  0.045600     23.6  3.00e-01   4.07e-01
## TG(18:2/18:1/16:0)      0.007520  -8.91e-03  0.023900     25.3  3.69e-01   4.95e-01
## PC(34:1)                0.005490  -7.07e-03  0.018000     26.0  3.91e-01   5.18e-01
## PC(O-38:5)             -0.005040  -1.80e-02  0.007870     22.8  4.44e-01   5.80e-01
## SM(d18:2/24:1)         -0.004320  -1.66e-02  0.007990     24.0  4.91e-01   6.34e-01
## PC(36:4)                0.002820  -5.35e-03  0.011000     25.9  4.98e-01   6.35e-01
## PC(38:5)               -0.003130  -1.24e-02  0.006160     24.6  5.08e-01   6.35e-01
## SM(d36:2)               0.003980  -7.85e-03  0.015800     21.4  5.09e-01   6.35e-01
## SM(42:2)               -0.003370  -1.39e-02  0.007190     25.2  5.31e-01   6.54e-01
## TG(46:0)                0.008220  -2.20e-02  0.038500     20.4  5.93e-01   7.23e-01
## TG(58:9)                0.010900  -3.71e-02  0.058800     19.9  6.56e-01   7.90e-01
## TG(16:0/18:2/22:6)      0.011000  -4.21e-02  0.064200     21.0  6.84e-01   8.15e-01
## PC(O-38:4)             -0.003460  -2.07e-02  0.013700     21.7  6.93e-01   8.17e-01
## TG(47:1)               -0.004750  -3.11e-02  0.021600     19.6  7.23e-01   8.33e-01
## SM(d16:1/18:1)         -0.002230  -1.46e-02  0.010200     22.8  7.23e-01   8.33e-01
## PC(38:2)               -0.002440  -1.69e-02  0.012000     21.2  7.41e-01   8.44e-01
```

```
## SM(d33:1)           -0.002270  -1.64e-02   0.011800     21.3  7.52e-01   8.48e-01
## PC(34:3)             0.004120  -2.32e-02   0.031400     22.5  7.67e-01   8.56e-01
## PC(35:1)             0.002320  -1.39e-02   0.018600     21.8  7.79e-01   8.60e-01
## LPC(18:0)           -0.001360  -1.37e-02   0.011000     23.9  8.28e-01   9.02e-01
## LPC(18:1)            0.001640  -1.38e-02   0.017100     23.7  8.34e-01   9.02e-01
## PC(36:3)            -0.001290  -1.60e-02   0.013500     25.6  8.64e-01   9.23e-01
## PC(32:1)             0.002220  -2.48e-02   0.029200     23.3  8.72e-01   9.23e-01
## LPC(16:0)           -0.000536  -7.44e-03   0.006370     25.3  8.79e-01   9.23e-01
## PC(40:5)            -0.001080  -1.85e-02   0.016400     22.0  9.03e-01   9.36e-01
## LPC(16:1)            0.001060  -1.88e-02   0.021000     20.0  9.17e-01   9.36e-01
## SM(d36:1)           -0.000566  -1.15e-02   0.010300     23.4  9.19e-01   9.36e-01
## PC(33:1)             0.000188  -1.88e-02   0.019200     20.9  9.85e-01   9.85e-01
## PC(O-36:4)           0.000145  -1.52e-02   0.015400     22.9  9.85e-01   9.85e-01
```

1.1.3.1.2 Heatmaps

```
##   Label Relation p.adj
## 1     #  p.adj <  0.01
## 2     x  p.adj <  0.05
## 3     *  p.adj <  0.10
```

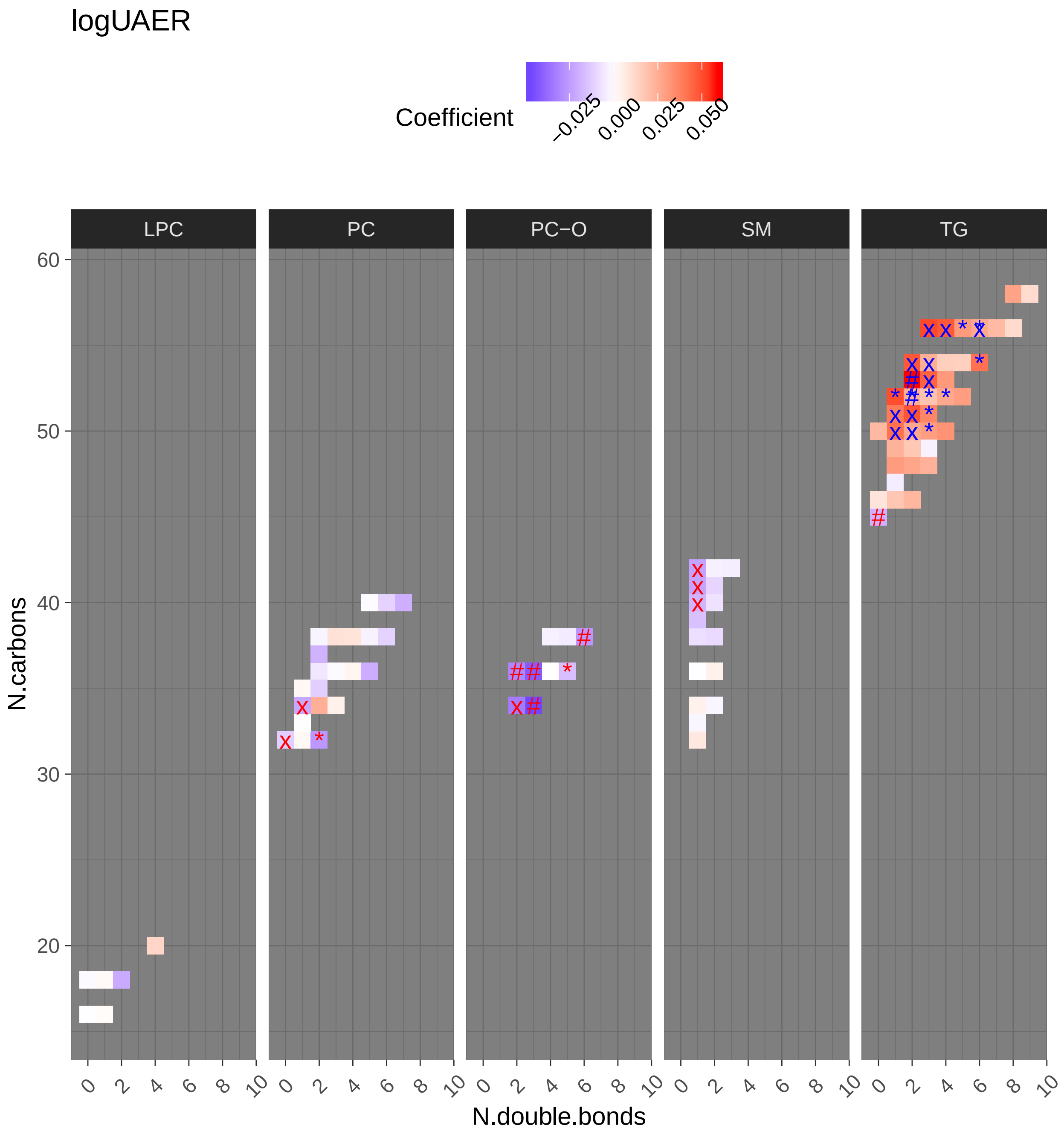

Figure 8: Heatmap of the lipid-specific model coefficents from the crude model for continuous albuminuria (logUAER). The name of the group is shown in the title of the figure in the top-left corner. Lipids are organized into panels (columns) by their class (from LPC to TG). One colored rectangle corresponds to one lipid species, where the coordinates in the x-axis and y-axis, respectively, show the number of double bonds (N.double.bonds) and the number of carbon atoms (N.carbons) in the fatty acid chains of the lipid. The number of double bonds corresponds to the number of unsaturated bonds in the fatty acid chains, and the number of carbon atoms corresponds to the length of the fatty acid chains and, thus, the size of the lipid. The magnitude of the lipid-specifc model coefficient is shown as the color of the rectangle (blue: negative; red: positive). Coefficient with statistical significance are annotated with symbols #, x and *, respectively, corresponding to $p < 0.01$, $p < 0.05$ and $p < 0.1$.

1.1.3.2 Adjusted Model

1.1.3.2.1 Table

```
##
##
## Table: Results of the adjusted model for the continuous albuminuria variable (logUAER).
## Shown in the table are name of the lipid (Name),
## model coefficient (Coefficient),
## its lower and upper confidence intervals (CI.L, CL.R),
## average value of the variable (AveExpr),
## p-value of the coefficient (P.Value) and
## the p-value after correction for multiple testing (adj.P.Val).
## The rows of the table are shown in an increasing order by the respective p-value.
##
## Name                  Coefficient       CI.L        CI.R   AveExpr    P.Value   adj.P.Val
## -------------------  ------------  ---------  ----------  --------  ---------  ----------
## SM(d18:1/24:0)           -2.84e-02   -0.04170   -0.015200      23.5   2.95e-05     0.00264
## SM(d40:1)                -2.53e-02   -0.03750   -0.013200      23.9   4.97e-05     0.00264
## SM(d41:1)                -2.63e-02   -0.04180   -0.010800      22.7   9.32e-04     0.03290
## TG(18:0/18:1/20:4)       -5.12e-02   -0.08500   -0.017300      21.3   3.09e-03     0.08180
## SM(d38:1)                -1.77e-02   -0.02980   -0.005630      23.1   4.14e-03     0.08770
## PC(O-34:3)               -2.45e-02   -0.04170   -0.007370      21.9   5.16e-03     0.09110
## SM(d38:2)                -1.80e-02   -0.03160   -0.004340      21.3   9.85e-03     0.13100
## SM(d40:2)                -1.43e-02   -0.02520   -0.003460      23.7   9.89e-03     0.13100
## SM(d39:1)                -2.26e-02   -0.04220   -0.002950      21.1   2.43e-02     0.27100
## PC(O-38:6)               -1.74e-02   -0.03270   -0.002100      20.4   2.59e-02     0.27100
## PC(O-36:5)               -1.85e-02   -0.03520   -0.001920      22.3   2.89e-02     0.27100
## PC(O-36:3)               -1.99e-02   -0.03800   -0.001670      20.4   3.25e-02     0.27100
## PC(O-36:2)               -1.88e-02   -0.03610   -0.001500      20.5   3.32e-02     0.27100
## LPC(20:4)                -2.44e-02   -0.04770   -0.001010      21.4   4.09e-02     0.31000
## SM(d16:1/18:1)           -1.02e-02   -0.02060    0.000109      22.8   5.25e-02     0.37100
## PC(36:2)                 -9.97e-03   -0.02090    0.001000      26.1   7.49e-02     0.46300
## LPC(18:2)                -2.08e-02   -0.04440    0.002760      24.0   8.35e-02     0.46300
## TG(56:5)                 -1.97e-02   -0.04200    0.002640      20.3   8.38e-02     0.46300
## SM(d36:2)                -1.06e-02   -0.02260    0.001440      21.4   8.43e-02     0.46300
## SM(d36:1)                -9.20e-03   -0.01980    0.001350      23.4   8.73e-02     0.46300
## TG(18:2/18:1/18:1)       -1.86e-02   -0.04070    0.003430      24.4   9.78e-02     0.49000
## SM(d41:2)                -1.22e-02   -0.02690    0.002600      22.5   1.06e-01     0.49000
## TG(54:4)                 -1.58e-02   -0.03510    0.003390      21.4   1.06e-01     0.49000
## PC(38:4)                 -8.23e-03   -0.01930    0.002840      25.2   1.45e-01     0.63600
## TG(56:6)                 -1.43e-02   -0.03400    0.005380      19.9   1.54e-01     0.63600
## PC(O-34:2)               -1.66e-02   -0.03970    0.006510      21.5   1.59e-01     0.63600
## TG(54:3)                 -1.03e-02   -0.02480    0.004150      21.7   1.62e-01     0.63600
## TG(16:0/22:5/18:1)       -1.56e-02   -0.03810    0.006880      22.7   1.74e-01     0.64400
## TG(52:3)                 -7.75e-03   -0.01900    0.003490      22.3   1.76e-01     0.64400
## TG(18:1/18:2/18:2)       -1.84e-02   -0.04610    0.009340      23.6   1.93e-01     0.66500
## TG(50:0)                 -2.21e-02   -0.05550    0.011400      20.8   1.95e-01     0.66500
## TG(18:1/18:1/18:1)       -1.37e-02   -0.03470    0.007350      24.8   2.02e-01     0.66500
## TG(54:5)                 -1.53e-02   -0.03980    0.009090      20.6   2.18e-01     0.66500
## PC(O-38:4)               -1.22e-02   -0.03190    0.007480      21.7   2.24e-01     0.66500
## TG(18:1/18:1/22:6)        3.32e-02   -0.02030    0.086700      20.7   2.24e-01     0.66500
## TG(16:0/18:2/18:2)       -1.47e-02   -0.03840    0.009090      24.5   2.26e-01     0.66500
## PC(32:1)                  1.76e-02   -0.01150    0.046600      23.3   2.36e-01     0.67500
## PC(38:2)                  8.75e-03   -0.00596    0.023500      21.2   2.43e-01     0.67800
## SM(d18:2/24:1)           -7.03e-03   -0.01900    0.004950      24.0   2.50e-01     0.67800
```

```
## TG(18:1/18:1/16:0)        1.22e-02   -0.00887    0.033300       25.6   2.56e-01       0.67900
## TG(52:4)                 -9.75e-03   -0.02760    0.008140       21.6   2.85e-01       0.73700
## TG(48:3)                 -1.97e-02   -0.05690    0.017600       20.5   3.00e-01       0.73900
## TG(54:2)                 -1.07e-02   -0.03120    0.009810       23.4   3.06e-01       0.73900
## PC(40:6)                  1.06e-02   -0.00976    0.031000       23.9   3.07e-01       0.73900
## TG(18:2/22:5/16:0)        2.16e-02   -0.02080    0.064000       22.1   3.18e-01       0.74000
## SM(42:2)                 -5.15e-03   -0.01540    0.005120       25.2   3.25e-01       0.74000
## PC(40:5)                  8.90e-03   -0.00896    0.026800       22.0   3.28e-01       0.74000
## SM(d34:1)                 3.05e-03   -0.00338    0.009480       24.9   3.52e-01       0.76500
## TG(50:3)                 -7.59e-03   -0.02400    0.008780       23.5   3.63e-01       0.76500
## PC(34:3)                  1.35e-02   -0.01580    0.042900       22.5   3.65e-01       0.76500
## PC(34:1)                  6.94e-03   -0.00819    0.022100       26.0   3.68e-01       0.76500
## PC(33:1)                  8.63e-03   -0.01090    0.028200       20.9   3.87e-01       0.77000
## PC(35:2)                 -8.36e-03   -0.02730    0.010600       21.8   3.87e-01       0.77000
## PC(40:7)                  8.86e-03   -0.01160    0.029300       21.4   3.95e-01       0.77000
## TG(56:4)                 -1.27e-02   -0.04230    0.016900       20.0   3.99e-01       0.77000
## PC(36:3)                 -6.78e-03   -0.02320    0.009610       25.6   4.17e-01       0.77800
## LPC(16:1)                 9.42e-03   -0.01340    0.032200       20.0   4.18e-01       0.77800
## TG(52:5)                 -1.31e-02   -0.04630    0.020100       19.8   4.38e-01       0.79600
## PC(38:3)                 -6.30e-03   -0.02240    0.009830       24.0   4.43e-01       0.79600
## PC(36:4)                 -3.72e-03   -0.01340    0.005990       25.9   4.52e-01       0.79900
## TG(49:3)                  1.08e-03   -0.00194    0.004100       20.7   4.84e-01       0.84100
## PC(34:2)                  9.87e-03   -0.01880    0.038500       26.0   4.98e-01       0.84100
## PC(38:6)                  4.76e-03   -0.00910    0.018600       25.3   5.00e-01       0.84100
## TG(53:4)                 -8.61e-03   -0.03750    0.020300       19.7   5.59e-01       0.92000
## TG(52:2)                 -2.80e-03   -0.01230    0.006730       22.6   5.64e-01       0.92000
## PC(O-36:4)               -4.97e-03   -0.02270    0.012800       22.9   5.82e-01       0.93500
## TG(56:3)                 -6.53e-03   -0.03130    0.018200       20.0   6.04e-01       0.94700
## TG(45:0)                  1.85e-03   -0.00534    0.009050       23.1   6.14e-01       0.94700
## PC(32:0)                  2.54e-03   -0.00740    0.012500       22.5   6.16e-01       0.94700
## TG(16:0/18:2/18:3)       -9.63e-03   -0.04880    0.029600       22.0   6.30e-01       0.95200
## TG(51:2)                  4.73e-03   -0.01520    0.024700       21.6   6.42e-01       0.95200
## PC(O-38:5)               -3.25e-03   -0.01810    0.011600       22.8   6.68e-01       0.95200
## TG(18:2/18:1/16:0)        3.95e-03   -0.01470    0.022600       25.3   6.78e-01       0.95200
## SM(d33:1)                -2.99e-03   -0.01730    0.011300       21.3   6.81e-01       0.95200
## TG(14:0/16:0/18:1)        4.95e-03   -0.02160    0.031500       22.8   7.15e-01       0.95200
## PC(16:0e/18:1(9Z)        -2.72e-03   -0.01740    0.011900       21.8   7.15e-01       0.95200
## TG(58:9)                  1.01e-02   -0.04420    0.064400       19.9   7.16e-01       0.95200
## TG(16:0/18:0/18:1)       -6.11e-03   -0.03940    0.027200       23.1   7.19e-01       0.95200
## PC(32:2)                 -4.70e-03   -0.03050    0.021100       20.7   7.20e-01       0.95200
## TG(14:0/18:2/18:2)       -5.05e-03   -0.03370    0.023600       21.2   7.29e-01       0.95200
## TG(50:1)                  4.37e-03   -0.02180    0.030600       24.3   7.43e-01       0.95200
## TG(46:0)                 -4.62e-03   -0.03360    0.024400       20.4   7.55e-01       0.95200
## TG(18:2/18:2/18:2)       -4.63e-03   -0.03550    0.026200       22.1   7.68e-01       0.95200
## TG(49:2)                 -2.66e-03   -0.02210    0.016800       20.4   7.88e-01       0.95200
## TG(14:0/18:1/18:1)       -2.28e-03   -0.01930    0.014700       24.4   7.92e-01       0.95200
## TG(46:1)                 -3.95e-03   -0.03390    0.026000       21.3   7.95e-01       0.95200
## TG(53:3)                 -3.11e-03   -0.02690    0.020700       20.7   7.98e-01       0.95200
## TG(18:1/12:0/18:1)       -3.15e-03   -0.02880    0.022500       22.5   8.09e-01       0.95200
## TG(51:3)                 -2.84e-03   -0.02630    0.020600       20.7   8.12e-01       0.95200
## TG(51:1)                 -2.65e-03   -0.02690    0.021600       20.3   8.30e-01       0.95200
## PC(36:5)                 -3.35e-03   -0.03550    0.028800       24.3   8.38e-01       0.95200
## LPC(16:0)                -7.46e-04   -0.00821    0.006720       25.3   8.44e-01       0.95200
## LPC(18:0)                 1.24e-03   -0.01240    0.014900       23.9   8.59e-01       0.95200
```

```
## TG(16:0/18:2/22:6)       -5.16e-03   -0.06280    0.052500       21.0   8.61e-01      0.95200
## PC(38:5)                  8.69e-04   -0.00909    0.010800       24.6   8.64e-01      0.95200
## SM(d32:1)                 1.11e-03   -0.01220    0.014400       22.3   8.69e-01      0.95200
## TG(56:7)                  1.92e-03   -0.02240    0.026200       19.6   8.77e-01      0.95200
## TG(47:1)                  2.39e-03   -0.02890    0.033700       19.6   8.81e-01      0.95200
## LPC(18:1)                -1.20e-03   -0.01870    0.016300       23.7   8.93e-01      0.95200
## PC(37:2)                  1.44e-03   -0.02180    0.024700       19.3   9.03e-01      0.95200
## TG(49:1)                 -1.31e-03   -0.02340    0.020800       20.9   9.07e-01      0.95200
## TG(50:2)                  7.19e-04   -0.01370    0.015200       21.1   9.22e-01      0.95800
## TG(53:2)                 -9.74e-04   -0.02520    0.023300       20.7   9.37e-01      0.96200
## PC(35:1)                  5.15e-04   -0.01630    0.017300       21.8   9.52e-01      0.96200
## TG(46:2)                 -9.01e-04   -0.03110    0.029300       20.1   9.53e-01      0.96200
## TG(54:6)                 -6.16e-05   -0.03360    0.033500       19.5   9.97e-01      0.99700
```

1.1.3.2.2 Heatmaps

```
## [1] "map_lipid_names has been created by Tommi Suvitaival"
## [1] "tommi.raimo.leo.suvitaival@regionh.dk"
## [1] "2019-05-06"
```

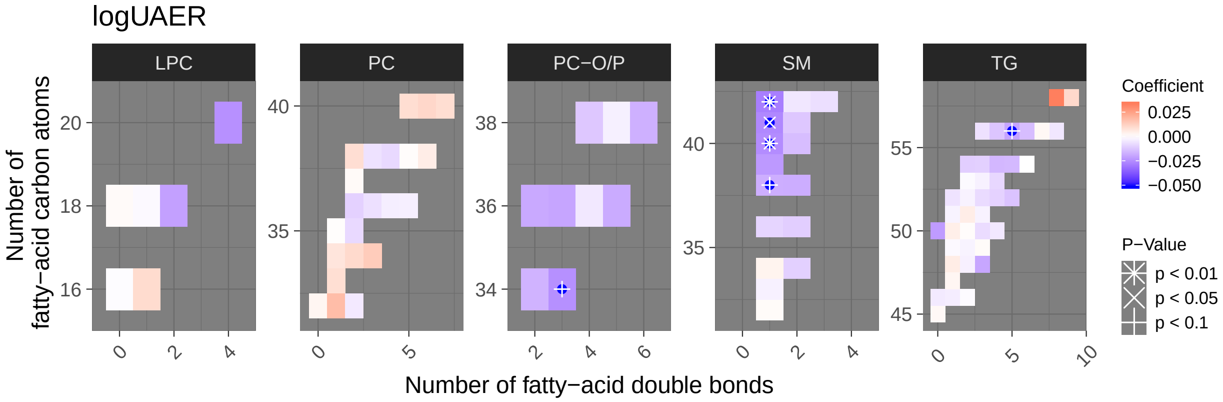

1.1.4 Combined Heatmaps from Adjusted Cross-sectional Analyses

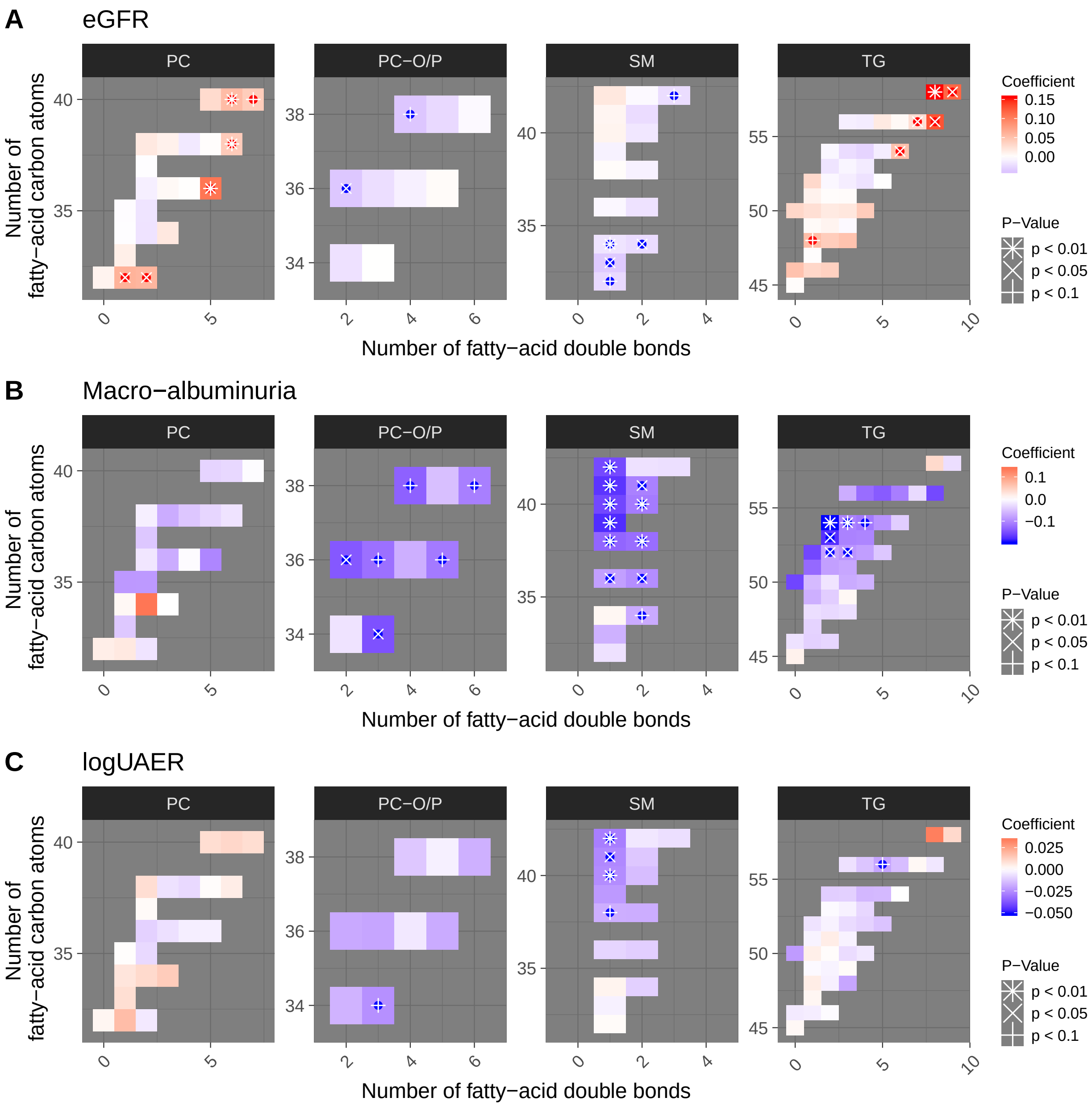
A
eGFR
B
Macro−albuminuria
C
logUAER
PC
PC−O/P
SM
TG
Number of fatty−acid carbon atoms
Number of fatty−acid double bonds
Coefficient
P−Value
p < 0.01
p < 0.05
p < 0.1

1.2 Step 1B: Crude Associations of All Lipids to Clinical Changes Over Time

1.2.1 eGFR Slope

1.2.1.1 Table

```
##
##
## Table: Results of the crude model for the eGFR slope.
## Shown in the table are name of the lipid (Name),
## model coefficient (Coefficient),
## its lower and upper confidence intervals (CI.L, CL.R),
## average value of the variable (AveExpr),
## p-value of the coefficient (P.Value) and
## the p-value after correction for multiple testing (adj.P.Val).
## The rows of the table are shown in an increasing order by the respective p-value.
##
## Name                   Coefficient        CI.L        CI.R   AveExpr    P.Value   adj.P.Val
## -------------------  ------------  ----------  ----------  --------  ---------  ----------
## PC(36:4)                 -0.012800   -0.018600   -0.006930      26.0   2.14e-05     0.00227
## TG(14:0/16:0/18:1)       -0.035400   -0.058100   -0.012700      22.8   2.26e-03     0.06860
## LPC(16:0)                -0.007400   -0.012200   -0.002640      25.3   2.35e-03     0.06860
## PC(O-38:6)                0.015000    0.005030    0.024900      20.4   3.22e-03     0.06860
## TG(51:3)                 -0.030200   -0.050700   -0.009640      20.7   4.04e-03     0.06860
## TG(51:2)                 -0.029400   -0.049700   -0.009180      21.5   4.47e-03     0.06860
## TG(50:2)                 -0.021000   -0.035600   -0.006330      21.1   5.08e-03     0.06860
## TG(14:0/18:1/18:1)       -0.025000   -0.042500   -0.007520      24.4   5.18e-03     0.06860
## TG(50:0)                 -0.035200   -0.061500   -0.008980      20.8   8.62e-03     0.08520
## TG(18:2/18:2/18:2)       -0.031500   -0.055300   -0.007820      22.1   9.27e-03     0.08520
## TG(51:1)                 -0.028700   -0.050300   -0.007010      20.3   9.59e-03     0.08520
## TG(46:0)                 -0.027600   -0.048600   -0.006740      20.4   9.64e-03     0.08520
## TG(50:3)                 -0.023500   -0.042700   -0.004340      23.4   1.64e-02     0.13100
## TG(52:2)                 -0.012000   -0.021800   -0.002120      22.6   1.74e-02     0.13100
## TG(49:1)                 -0.021000   -0.038500   -0.003490      20.8   1.88e-02     0.13300
## TG(18:1/12:0/18:1)       -0.027400   -0.050700   -0.004130      22.4   2.11e-02     0.14000
## PC(32:1)                 -0.022500   -0.042100   -0.002910      23.3   2.45e-02     0.15000
## TG(46:1)                 -0.026100   -0.049000   -0.003210      21.2   2.55e-02     0.15000
## TG(50:1)                 -0.026100   -0.049400   -0.002820      24.3   2.81e-02     0.15000
## TG(16:0/18:0/18:1)       -0.033000   -0.062800   -0.003200      23.1   3.01e-02     0.15000
## PC(38:3)                 -0.012400   -0.023800   -0.001050      24.0   3.23e-02     0.15000
## TG(14:0/18:2/18:2)       -0.029900   -0.057400   -0.002490      21.1   3.26e-02     0.15000
## PC(O-36:2)                0.012600    0.001040    0.024100      20.5   3.26e-02     0.15000
## TG(49:2)                 -0.016800   -0.032400   -0.001220      20.4   3.47e-02     0.15200
## TG(53:2)                 -0.024500   -0.047500   -0.001620      20.6   3.60e-02     0.15200
## TG(48:3)                 -0.032900   -0.064000   -0.001720      20.5   3.87e-02     0.15600
## TG(56:5)                 -0.019500   -0.038200   -0.000833      20.3   4.07e-02     0.15600
## PC(36:3)                 -0.011100   -0.021700   -0.000450      25.6   4.11e-02     0.15600
## PC(33:1)                 -0.014400   -0.028400   -0.000378      20.8   4.42e-02     0.15800
## TG(16:0/22:5/18:1)       -0.018700   -0.036900   -0.000453      22.7   4.46e-02     0.15800
## TG(46:2)                 -0.024800   -0.049400   -0.000318      20.1   4.71e-02     0.16100
## TG(53:3)                 -0.019900   -0.041300    0.001400      20.7   6.70e-02     0.22200
## LPC(16:1)                -0.013100   -0.027400    0.001300      20.0   7.45e-02     0.23900
## PC(34:3)                 -0.017700   -0.037700    0.002240      22.4   8.17e-02     0.24700
## PC(O-38:5)                0.008360   -0.001100    0.017800      22.8   8.30e-02     0.24700
## TG(18:2/22:5/16:0)       -0.026400   -0.056300    0.003540      22.1   8.39e-02     0.24700
## PC(32:2)                 -0.015500   -0.033200    0.002220      20.7   8.64e-02     0.24700
## TG(52:3)                 -0.008570   -0.018900    0.001720      22.3   1.03e-01     0.28100
## TG(18:0/18:1/20:4)       -0.020000   -0.044400    0.004420      21.3   1.08e-01     0.28100
```

```
## TG(45:0)                  0.006260   -0.001450   0.014000    23.1   1.11e-01    0.28100
## PC(O-34:3)                0.009890   -0.002290   0.022100    21.9   1.11e-01    0.28100
## PC(36:2)                 -0.006190   -0.013900   0.001470    26.1   1.13e-01    0.28100
## TG(49:3)                  0.002120   -0.000512   0.004760    20.7   1.14e-01    0.28100
## TG(54:2)                 -0.017100   -0.038800   0.004580    23.4   1.22e-01    0.29300
## PC(O-34:2)                0.011200   -0.003900   0.026300    21.5   1.46e-01    0.34300
## TG(16:0/18:2/18:2)       -0.014900   -0.035500   0.005600    24.5   1.54e-01    0.35400
## PC(16:0e/18:1(9Z)         0.007230   -0.002810   0.017300    21.8   1.58e-01    0.35600
## TG(52:5)                 -0.017100   -0.041200   0.006940    19.8   1.63e-01    0.36000
## TG(56:6)                 -0.010400   -0.025100   0.004430    19.9   1.69e-01    0.36600
## TG(56:7)                 -0.010700   -0.027500   0.006090    19.6   2.11e-01    0.44700
## LPC(18:0)                -0.005540   -0.014300   0.003240    24.0   2.16e-01    0.44800
## TG(18:1/18:1/16:0)       -0.007830   -0.021000   0.005300    25.6   2.42e-01    0.48800
## TG(54:6)                 -0.015200   -0.040900   0.010400    19.5   2.44e-01    0.48800
## PC(40:7)                  0.007890   -0.005830   0.021600    21.4   2.59e-01    0.50300
## PC(34:1)                  0.005220   -0.003920   0.014400    25.9   2.63e-01    0.50300
## SM(d41:2)                 0.006020   -0.004590   0.016600    22.5   2.66e-01    0.50300
## PC(40:5)                 -0.006980   -0.019600   0.005660    22.0   2.79e-01    0.51800
## SM(d39:1)                 0.007040   -0.005970   0.020000    21.1   2.88e-01    0.52200
## PC(35:1)                 -0.006450   -0.018400   0.005530    21.8   2.91e-01    0.52200
## TG(16:0/18:2/18:3)       -0.016500   -0.047600   0.014600    22.0   2.98e-01    0.52600
## LPC(18:1)                -0.005800   -0.017100   0.005520    23.7   3.15e-01    0.54700
## TG(18:1/18:1/22:6)       -0.017200   -0.051300   0.017000    20.7   3.23e-01    0.55200
## SM(d41:1)                 0.005540   -0.005750   0.016800    22.7   3.35e-01    0.56400
## PC(O-36:3)                0.006160   -0.006810   0.019100    20.4   3.51e-01    0.57500
## TG(53:4)                 -0.010900   -0.034100   0.012300    19.7   3.58e-01    0.57500
## PC(35:2)                  0.005610   -0.006570   0.017800    21.8   3.66e-01    0.57500
## PC(O-36:5)                0.004720   -0.005550   0.015000    22.3   3.67e-01    0.57500
## SM(d32:1)                -0.004430   -0.014100   0.005260    22.3   3.69e-01    0.57500
## PC(38:4)                 -0.003100   -0.010100   0.003920    25.2   3.86e-01    0.58400
## TG(52:4)                 -0.006750   -0.022200   0.008660    21.6   3.90e-01    0.58400
## PC(38:5)                 -0.002930   -0.009650   0.003780    24.6   3.91e-01    0.58400
## SM(d34:1)                 0.001860   -0.002470   0.006190    24.9   3.99e-01    0.58700
## SM(d33:1)                 0.004150   -0.005770   0.014100    21.3   4.12e-01    0.59800
## TG(56:3)                 -0.008500   -0.030900   0.013900    20.0   4.56e-01    0.65300
## TG(18:1/18:1/18:1)       -0.006390   -0.023600   0.010800    24.8   4.65e-01    0.65600
## TG(16:0/18:2/22:6)       -0.014200   -0.053100   0.024700    21.0   4.74e-01    0.65600
## TG(54:3)                 -0.004750   -0.017900   0.008360    21.7   4.77e-01    0.65600
## PC(38:2)                 -0.003190   -0.013500   0.007130    21.3   5.44e-01    0.73900
## PC(40:6)                  0.004160   -0.009910   0.018200    23.9   5.62e-01    0.75400
## TG(58:9)                 -0.009850   -0.044500   0.024800    19.9   5.76e-01    0.75500
## SM(d18:2/24:1)            0.002410   -0.006080   0.010900    24.0   5.77e-01    0.75500
## TG(18:2/18:1/18:1)       -0.004280   -0.021500   0.013000    24.4   6.27e-01    0.80100
## SM(d40:2)                 0.002030   -0.006190   0.010300    23.7   6.28e-01    0.80100
## SM(d38:1)                 0.002100   -0.006660   0.010900    23.1   6.38e-01    0.80500
## TG(18:1/18:2/18:2)       -0.004810   -0.026700   0.017000    23.6   6.65e-01    0.81300
## PC(O-38:4)               -0.002830   -0.015700   0.010000    21.7   6.65e-01    0.81300
## SM(d38:2)                 0.002040   -0.007320   0.011400    21.4   6.68e-01    0.81300
## SM(42:2)                  0.001580   -0.005800   0.008960    25.2   6.75e-01    0.81300
## PC(38:6)                  0.001880   -0.007210   0.011000    25.3   6.85e-01    0.81600
## SM(d40:1)                 0.001860   -0.007510   0.011200    24.0   6.97e-01    0.82100
## SM(d36:2)                 0.001370   -0.006720   0.009450    21.4   7.40e-01    0.86100
## PC(34:2)                  0.003020   -0.015400   0.021400    26.0   7.48e-01    0.86100
## TG(47:1)                 -0.002760   -0.021900   0.016400    19.5   7.78e-01    0.88600
```

```
## LPC(20:4)              -0.002000   -0.016700    0.012700      21.4   7.89e-01     0.89000
## SM(d18:1/24:0)         -0.001260   -0.011200    0.008640      23.6   8.03e-01     0.89100
## TG(54:5)                0.002260   -0.016100    0.020600      20.6   8.09e-01     0.89100
## SM(d16:1/18:1)         -0.001010   -0.009550    0.007530      22.8   8.16e-01     0.89100
## PC(32:0)                0.000682   -0.005980    0.007340      22.5   8.41e-01     0.90900
## PC(36:5)               -0.002100   -0.024300    0.020100      24.3   8.52e-01     0.91300
## SM(d36:1)              -0.000595   -0.008270    0.007080      23.4   8.79e-01     0.93200
## TG(56:4)               -0.001740   -0.026800    0.023300      20.0   8.92e-01     0.93600
## LPC(18:2)              -0.000865   -0.015500    0.013800      24.0   9.08e-01     0.94300
## PC(37:2)                0.000590   -0.015200    0.016300      19.3   9.41e-01     0.95500
## TG(18:2/18:1/16:0)      0.000421   -0.011600    0.012500      25.3   9.45e-01     0.95500
## PC(O-36:4)             -0.000376   -0.011400    0.010600      22.9   9.46e-01     0.95500
## TG(54:4)               -0.000244   -0.015300    0.014800      21.4   9.75e-01     0.97500
##
##
## Table: Results of the crude model for the eGFR slope.
## Shown in the table are name of the lipid (Name),
## model coefficient (Coefficient),
## its lower and upper confidence intervals (CI.L, CL.R),
## average value of the variable (AveExpr),
## p-value of the coefficient (P.Value) and
## the p-value after correction for multiple testing (adj.P.Val).
## The rows of the table are shown in an increasing order by the respective p-value.
##
## Name                    Coefficient        CI.L        CI.R   AveExpr    P.Value   adj.P.Val
## -------------------  ------------  ----------  ----------  --------  ---------  ----------
## SM(d36:2)                -3.15e-03   -4.22e-03   -2.08e-03      21.4   0.00e+00    1.30e-06
## SM(d34:1)                -1.37e-03   -1.94e-03   -7.96e-04      24.9   3.40e-06    1.36e-04
## SM(d16:1/18:1)           -2.68e-03   -3.81e-03   -1.55e-03      22.8   3.90e-06    1.36e-04
## PC(38:4)                 -2.12e-03   -3.05e-03   -1.19e-03      25.2   8.70e-06    2.31e-04
## TG(18:0/18:1/20:4)       -6.80e-03   -1.00e-02   -3.58e-03      21.3   3.98e-05    8.44e-04
## SM(d18:2/24:1)           -2.31e-03   -3.43e-03   -1.18e-03      24.0   6.19e-05    1.09e-03
## LPC(18:2)                 3.87e-03    1.94e-03    5.80e-03      24.0   9.59e-05    1.45e-03
## SM(d32:1)                -2.45e-03   -3.73e-03   -1.17e-03      22.3   1.89e-04    2.51e-03
## SM(d18:1/24:0)            2.20e-03    8.95e-04    3.51e-03      23.6   1.00e-03    1.18e-02
## SM(d33:1)                -2.08e-03   -3.39e-03   -7.67e-04      21.3   1.95e-03    2.06e-02
## SM(d38:2)                -1.87e-03   -3.11e-03   -6.36e-04      21.4   3.08e-03    2.96e-02
## LPC(16:0)                 9.24e-04    2.95e-04    1.55e-03      25.3   4.06e-03    3.59e-02
## SM(d36:1)                -1.39e-03   -2.41e-03   -3.77e-04      23.4   7.27e-03    5.93e-02
## PC(32:2)                  3.10e-03    7.61e-04    5.44e-03      20.7   9.49e-03    7.18e-02
## SM(d41:2)                -1.69e-03   -3.09e-03   -2.87e-04      22.5   1.83e-02    1.29e-01
## SM(d40:2)                -1.26e-03   -2.35e-03   -1.78e-04      23.7   2.26e-02    1.47e-01
## SM(d40:1)                 1.43e-03    1.94e-04    2.67e-03      24.0   2.35e-02    1.47e-01
## TG(53:2)                 -3.20e-03   -6.23e-03   -1.75e-04      20.6   3.82e-02    2.15e-01
## PC(38:5)                 -9.37e-04   -1.82e-03   -4.94e-05      24.6   3.86e-02    2.15e-01
## TG(16:0/22:5/18:1)       -2.45e-03   -4.86e-03   -4.27e-05      22.7   4.61e-02    2.44e-01
## TG(56:6)                 -1.93e-03   -3.89e-03    2.04e-05      19.9   5.24e-02    2.65e-01
## TG(52:2)                 -1.24e-03   -2.54e-03    6.17e-05      22.6   6.19e-02    2.89e-01
## LPC(18:1)                 1.42e-03   -7.53e-05    2.92e-03      23.7   6.27e-02    2.89e-01
## LPC(18:0)                 1.07e-03   -8.74e-05    2.23e-03      24.0   6.99e-02    3.09e-01
## TG(56:5)                 -2.23e-03   -4.70e-03    2.39e-04      20.3   7.66e-02    3.14e-01
## PC(O-34:3)                1.45e-03   -1.58e-04    3.06e-03      21.9   7.70e-02    3.14e-01
## PC(16:0e/18:1(9Z)        -1.18e-03   -2.51e-03    1.43e-04      21.8   8.01e-02    3.15e-01
## PC(40:6)                 -1.64e-03   -3.50e-03    2.19e-04      23.9   8.36e-02    3.16e-01
```

## PC(38:3)	-1.27e-03	-2.78e-03	2.30e-04	24.0	9.66e-02	3.43e-01
## TG(54:3)	-1.47e-03	-3.20e-03	2.67e-04	21.7	9.72e-02	3.43e-01
## PC(40:5)	-1.37e-03	-3.05e-03	2.97e-04	22.0	1.07e-01	3.56e-01
## PC(35:1)	-1.30e-03	-2.88e-03	2.84e-04	21.8	1.08e-01	3.56e-01
## TG(51:2)	-2.12e-03	-4.80e-03	5.57e-04	21.5	1.20e-01	3.87e-01
## TG(54:2)	-2.23e-03	-5.10e-03	6.33e-04	23.4	1.26e-01	3.92e-01
## SM(d41:1)	1.14e-03	-3.49e-04	2.64e-03	22.7	1.33e-01	3.92e-01
## PC(37:2)	1.59e-03	-4.87e-04	3.68e-03	19.3	1.33e-01	3.92e-01
## PC(O-38:6)	-9.80e-04	-2.29e-03	3.32e-04	20.4	1.43e-01	4.07e-01
## PC(34:2)	-1.80e-03	-4.23e-03	6.29e-04	26.0	1.46e-01	4.07e-01
## TG(56:3)	-2.11e-03	-5.07e-03	8.48e-04	20.0	1.62e-01	4.36e-01
## TG(45:0)	7.22e-04	-2.96e-04	1.74e-03	23.1	1.64e-01	4.36e-01
## SM(42:2)	-6.60e-04	-1.64e-03	3.15e-04	25.2	1.84e-01	4.66e-01
## TG(52:3)	-9.16e-04	-2.28e-03	4.44e-04	22.3	1.86e-01	4.66e-01
## PC(34:3)	1.77e-03	-8.73e-04	4.41e-03	22.4	1.89e-01	4.66e-01
## PC(35:2)	1.01e-03	-5.97e-04	2.62e-03	21.8	2.17e-01	5.16e-01
## TG(53:3)	-1.77e-03	-4.58e-03	1.05e-03	20.7	2.19e-01	5.16e-01
## TG(51:1)	-1.75e-03	-4.61e-03	1.11e-03	20.3	2.31e-01	5.31e-01
## LPC(16:1)	1.14e-03	-7.56e-04	3.04e-03	20.0	2.37e-01	5.35e-01
## TG(49:3)	2.00e-04	-1.48e-04	5.48e-04	20.7	2.60e-01	5.39e-01
## TG(53:4)	-1.76e-03	-4.83e-03	1.31e-03	19.7	2.60e-01	5.39e-01
## TG(18:1/18:1/16:0)	-9.35e-04	-2.67e-03	8.01e-04	25.6	2.90e-01	5.39e-01
## TG(50:2)	-1.04e-03	-2.97e-03	8.94e-04	21.1	2.91e-01	5.39e-01
## TG(54:4)	-1.06e-03	-3.04e-03	9.26e-04	21.4	2.95e-01	5.39e-01
## PC(36:4)	-4.13e-04	-1.19e-03	3.61e-04	26.0	2.95e-01	5.39e-01
## TG(18:1/18:1/18:1)	-1.21e-03	-3.48e-03	1.06e-03	24.8	2.96e-01	5.39e-01
## PC(O-34:2)	1.06e-03	-9.36e-04	3.05e-03	21.5	2.98e-01	5.39e-01
## PC(O-38:5)	-6.62e-04	-1.91e-03	5.88e-04	22.8	2.99e-01	5.39e-01
## TG(52:4)	-1.08e-03	-3.12e-03	9.59e-04	21.6	2.99e-01	5.39e-01
## PC(O-36:2)	-8.03e-04	-2.33e-03	7.21e-04	20.5	3.01e-01	5.39e-01
## TG(14:0/18:1/18:1)	-1.22e-03	-3.53e-03	1.10e-03	24.4	3.02e-01	5.39e-01
## TG(56:7)	-1.16e-03	-3.38e-03	1.06e-03	19.6	3.05e-01	5.39e-01
## TG(18:2/18:2/18:2)	-1.51e-03	-4.65e-03	1.62e-03	22.1	3.43e-01	5.96e-01
## TG(16:0/18:0/18:1)	-1.79e-03	-5.72e-03	2.15e-03	23.1	3.73e-01	6.35e-01
## TG(18:2/22:5/16:0)	-1.78e-03	-5.73e-03	2.18e-03	22.1	3.78e-01	6.35e-01
## TG(49:1)	-9.96e-04	-3.31e-03	1.32e-03	20.8	3.97e-01	6.35e-01
## TG(51:3)	-1.17e-03	-3.88e-03	1.54e-03	20.7	3.98e-01	6.35e-01
## TG(49:2)	-8.76e-04	-2.93e-03	1.18e-03	20.4	4.03e-01	6.35e-01
## PC(O-38:4)	-7.19e-04	-2.41e-03	9.76e-04	21.7	4.05e-01	6.35e-01
## TG(18:2/18:1/18:1)	-9.62e-04	-3.24e-03	1.32e-03	24.4	4.08e-01	6.35e-01
## TG(18:2/18:1/16:0)	-6.62e-04	-2.25e-03	9.29e-04	25.3	4.14e-01	6.36e-01
## TG(16:0/18:2/18:2)	-1.08e-03	-3.80e-03	1.63e-03	24.5	4.33e-01	6.56e-01
## TG(50:3)	-9.59e-04	-3.49e-03	1.58e-03	23.4	4.58e-01	6.58e-01
## PC(40:7)	-6.86e-04	-2.50e-03	1.13e-03	21.4	4.58e-01	6.58e-01
## PC(33:1)	-7.00e-04	-2.55e-03	1.15e-03	20.8	4.58e-01	6.58e-01
## TG(56:4)	-1.23e-03	-4.54e-03	2.09e-03	20.0	4.67e-01	6.58e-01
## TG(50:1)	-1.14e-03	-4.21e-03	1.94e-03	24.3	4.69e-01	6.58e-01
## PC(32:0)	-3.23e-04	-1.20e-03	5.57e-04	22.5	4.72e-01	6.58e-01
## TG(54:6)	-1.21e-03	-4.60e-03	2.18e-03	19.5	4.83e-01	6.64e-01
## TG(16:0/18:2/18:3)	-1.39e-03	-5.50e-03	2.72e-03	22.0	5.07e-01	6.81e-01
## TG(50:0)	-1.17e-03	-4.64e-03	2.30e-03	20.8	5.08e-01	6.81e-01
## PC(36:3)	-4.38e-04	-1.84e-03	9.66e-04	25.6	5.40e-01	7.16e-01
## PC(34:1)	-3.59e-04	-1.57e-03	8.49e-04	25.9	5.60e-01	7.32e-01
## TG(16:0/18:2/22:6)	-1.40e-03	-6.55e-03	3.74e-03	21.0	5.92e-01	7.64e-01

```
## PC(38:6)              -3.22e-04  -1.52e-03  8.79e-04     25.3  5.99e-01  7.64e-01
## TG(52:5)              -7.78e-04  -3.96e-03  2.40e-03     19.8  6.31e-01  7.96e-01
## SM(d39:1)             -3.73e-04  -2.09e-03  1.35e-03     21.1  6.70e-01  8.28e-01
## TG(47:1)              -5.48e-04  -3.08e-03  1.99e-03     19.5  6.72e-01  8.28e-01
## TG(18:1/18:2/18:2)    -5.41e-04  -3.43e-03  2.35e-03     23.6  7.13e-01  8.69e-01
## PC(32:1)              -4.67e-04  -3.06e-03  2.12e-03     23.3  7.23e-01  8.71e-01
## TG(54:5)              -3.04e-04  -2.73e-03  2.12e-03     20.6  8.06e-01  9.60e-01
## TG(14:0/16:0/18:1)    -3.46e-04  -3.34e-03  2.65e-03     22.8  8.20e-01  9.63e-01
## LPC(20:4)              2.17e-04  -1.72e-03  2.16e-03     21.4  8.26e-01  9.63e-01
## PC(O-36:3)            -1.80e-04  -1.89e-03  1.53e-03     20.4  8.37e-01  9.64e-01
## TG(48:3)               3.90e-04  -3.73e-03  4.51e-03     20.5  8.53e-01  9.72e-01
## PC(36:5)              -2.48e-04  -3.18e-03  2.68e-03     24.3  8.68e-01  9.79e-01
## TG(46:0)               1.58e-04  -2.61e-03  2.92e-03     20.4  9.11e-01  9.92e-01
## TG(18:1/18:1/22:6)    -2.27e-04  -4.74e-03  4.28e-03     20.7  9.21e-01  9.92e-01
## TG(58:9)               2.28e-04  -4.35e-03  4.80e-03     19.9  9.22e-01  9.92e-01
## TG(18:1/12:0/18:1)    -9.00e-05  -3.17e-03  2.99e-03     22.4  9.54e-01  9.92e-01
## SM(d38:1)              3.02e-05  -1.13e-03  1.19e-03     23.1  9.59e-01  9.92e-01
## TG(14:0/18:2/18:2)    -9.44e-05  -3.72e-03  3.53e-03     21.1  9.59e-01  9.92e-01
## PC(36:2)               2.17e-05  -9.91e-04  1.03e-03     26.1  9.66e-01  9.92e-01
## PC(38:2)              -2.20e-05  -1.39e-03  1.34e-03     21.3  9.75e-01  9.92e-01
## PC(O-36:5)            -1.46e-05  -1.37e-03  1.34e-03     22.3  9.83e-01  9.92e-01
## PC(O-36:4)             8.80e-06  -1.45e-03  1.46e-03     22.9  9.90e-01  9.92e-01
## TG(46:1)              -1.82e-05  -3.05e-03  3.01e-03     21.2  9.91e-01  9.92e-01
## TG(46:2)              -1.67e-05  -3.26e-03  3.22e-03     20.1  9.92e-01  9.92e-01
```

1.2.1.2 Heatmaps

```
##   Label Relation p.adj
## 1     #  p.adj <  0.01
## 2     x  p.adj <  0.05
## 3     *  p.adj <  0.10
```

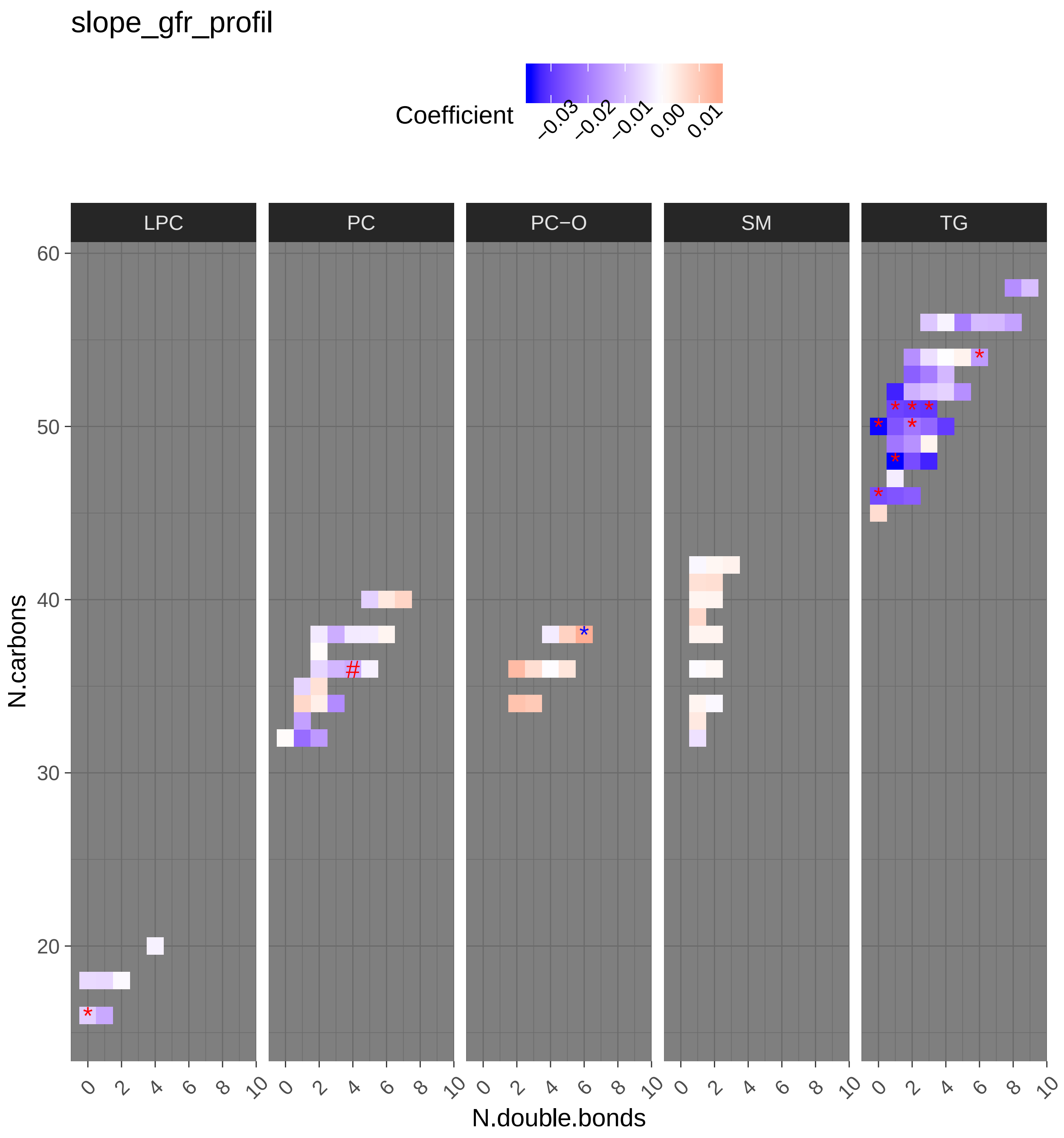

Figure 9: Heatmap of the lipid-specific model coefficents from the crude model for eGFR slope. The name of the group is shown in the title of the figure in the top-left corner. Lipids are organized into panels (columns) by their class (from LPC to TG). One colored rectangle corresponds to one lipid species, where the coordinates in the x-axis and y-axis, respectively, show the number of double bonds (N.double.bonds) and the number of carbon atoms (N.carbons) in the fatty acid chains of the lipid. The number of double bonds corresponds to the number of unsaturated bonds in the fatty acid chains, and the number of carbon atoms corresponds to the length of the fatty acid chains and, thus, the size of the lipid. The magnitude of the lipid-specifc model coefficient is shown as the color of the rectangle (blue: negative; red: positive). Coefficient with statistical significance are annotated with symbols #, x and *, respectively, corresponding to $p < 0.01$, $p < 0.05$ and $p < 0.1$.

1.2.2 Albuminuria Slope

1.2.2.1 Table

```
##
##
## Table: Results of the crude model for the continuous albuminuria (logUAER) slope.
## Shown in the table are name of the lipid (Name),
## model coefficient (Coefficient),
## its lower and upper confidence intervals (CI.L, CL.R),
## average value of the variable (AveExpr),
## p-value of the coefficient (P.Value) and
## the p-value after correction for multiple testing (adj.P.Val).
## The rows of the table are shown in an increasing order by the respective p-value.
##
## Name                  Coefficient      CI.L        CI.R   AveExpr    P.Value   adj.P.Val
## -------------------  ------------  --------  ----------  --------  ---------  ----------
## LPC(18:2)                -0.297000   -0.4640   -0.130000      24.0   0.000511      0.0315
## PC(O-36:5)               -0.193000   -0.3030   -0.083500      22.3   0.000595      0.0315
## PC(O-36:4)               -0.201000   -0.3200   -0.081300      22.9   0.001030      0.0364
## PC(O-38:5)               -0.164000   -0.2680   -0.060700      22.8   0.001940      0.0505
## PC(O-38:6)               -0.167000   -0.2760   -0.058800      20.4   0.002570      0.0505
## PC(O-38:4)               -0.210000   -0.3490   -0.071200      21.7   0.003090      0.0505
## SM(d18:1/24:0)           -0.168000   -0.2800   -0.056000      23.6   0.003340      0.0505
## PC(O-34:3)               -0.188000   -0.3190   -0.056700      21.9   0.005100      0.0676
## PC(O-34:2)               -0.232000   -0.3990   -0.066400      21.5   0.006180      0.0728
## SM(d40:1)                -0.138000   -0.2450   -0.031400      24.0   0.011300      0.1200
## LPC(20:4)                -0.200000   -0.3660   -0.034300      21.4   0.018200      0.1750
## SM(d38:1)                -0.113000   -0.2130   -0.012100      23.1   0.028200      0.2490
## LPC(18:1)                -0.137000   -0.2640   -0.009630      23.7   0.035000      0.2860
## SM(d41:1)                -0.129000   -0.2560   -0.000812      22.7   0.048600      0.3680
## LPC(16:0)                -0.049600   -0.1050    0.005320      25.3   0.076600      0.5130
## LPC(18:0)                -0.089500   -0.1890    0.009880      24.0   0.077400      0.5130
## SM(d39:1)                -0.130000   -0.2810    0.020500      21.1   0.090200      0.5630
## PC(O-36:3)               -0.112000   -0.2540    0.030700      20.4   0.124000      0.7310
## PC(36:2)                 -0.059700   -0.1390    0.019400      26.1   0.139000      0.7740
## PC(32:1)                  0.160000   -0.0605    0.381000      23.3   0.154000      0.8040
## TG(54:6)                  0.202000   -0.0843    0.489000      19.5   0.166000      0.8040
## TG(49:3)                 -0.021900   -0.0530    0.009180      20.7   0.167000      0.8040
## PC(32:0)                  0.051700   -0.0235    0.127000      22.5   0.177000      0.8040
## PC(34:1)                 -0.074200   -0.1830    0.034900      25.9   0.182000      0.8040
## PC(37:2)                 -0.118000   -0.2960    0.060600      19.3   0.195000      0.8050
## TG(50:2)                  0.110000   -0.0574    0.277000      21.1   0.197000      0.8050
## TG(14:0/18:1/18:1)        0.122000   -0.0781    0.323000      24.4   0.231000      0.8930
## PC(38:5)                 -0.045300   -0.1200    0.029700      24.6   0.236000      0.8930
## SM(d38:2)                -0.063700   -0.1710    0.043900      21.4   0.245000      0.8960
## SM(d32:1)                 0.061500   -0.0491    0.172000      22.3   0.275000      0.9570
## TG(50:0)                  0.160000   -0.1390    0.460000      20.8   0.293000      0.9570
## TG(14:0/16:0/18:1)        0.136000   -0.1230    0.395000      22.8   0.303000      0.9570
## TG(18:1/18:1/16:0)        0.076300   -0.0735    0.226000      25.6   0.317000      0.9570
## PC(O-36:2)               -0.065000   -0.1940    0.063700      20.5   0.322000      0.9570
## PC(36:3)                 -0.058200   -0.1770    0.060400      25.6   0.335000      0.9570
## TG(50:3)                  0.104000   -0.1160    0.323000      23.4   0.354000      0.9570
## SM(d40:2)                -0.043200   -0.1370    0.050500      23.7   0.366000      0.9570
## TG(14:0/18:2/18:2)        0.143000   -0.1710    0.457000      21.2   0.371000      0.9570
## SM(d18:2/24:1)            0.043600   -0.0538    0.141000      24.0   0.379000      0.9570
```

```
## PC(35:2)               -0.061400   -0.1990    0.076600      21.8   0.383000       0.9570
## SM(d16:1/18:1)          0.043500   -0.0547    0.142000      22.8   0.385000       0.9570
## TG(18:1/12:0/18:1)      0.118000   -0.1490    0.385000      22.5   0.386000       0.9570
## TG(18:2/18:1/16:0)      0.059500   -0.0759    0.195000      25.3   0.388000       0.9570
## TG(56:7)                0.078800   -0.1060    0.264000      19.6   0.403000       0.9570
## TG(18:2/18:2/18:2)      0.107000   -0.1590    0.373000      22.1   0.428000       0.9570
## SM(d34:1)               0.019400   -0.0300    0.068900      24.9   0.440000       0.9570
## TG(16:0/18:0/18:1)      0.132000   -0.2070    0.470000      23.1   0.444000       0.9570
## TG(18:0/18:1/20:4)      0.100000   -0.1680    0.369000      21.3   0.463000       0.9570
## TG(18:2/22:5/16:0)      0.125000   -0.2090    0.459000      22.1   0.464000       0.9570
## TG(49:2)                0.066200   -0.1130    0.245000      20.4   0.469000       0.9570
## PC(40:7)               -0.054800   -0.2090    0.099600      21.4   0.486000       0.9570
## PC(16:0e/18:1(9Z)      -0.038200   -0.1510    0.074900      21.8   0.507000       0.9570
## TG(46:0)                0.079600   -0.1610    0.320000      20.4   0.515000       0.9570
## LPC(16:1)               0.051300   -0.1130    0.215000      20.0   0.539000       0.9570
## PC(36:4)               -0.020300   -0.0874    0.046800      26.0   0.553000       0.9570
## PC(34:3)               -0.069400   -0.2990    0.160000      22.4   0.553000       0.9570
## TG(56:3)                0.075500   -0.1770    0.328000      20.0   0.558000       0.9570
## TG(56:6)               -0.048600   -0.2120    0.115000      19.9   0.559000       0.9570
## TG(45:0)               -0.025900   -0.1130    0.061600      23.1   0.561000       0.9570
## TG(16:0/18:2/18:2)      0.066300   -0.1690    0.302000      24.5   0.581000       0.9570
## PC(33:1)                0.044400   -0.1160    0.205000      20.9   0.587000       0.9570
## TG(48:3)                0.097500   -0.2640    0.459000      20.5   0.596000       0.9570
## TG(52:5)               -0.075100   -0.3540    0.204000      19.8   0.598000       0.9570
## TG(16:0/18:2/22:6)      0.115000   -0.3190    0.548000      21.0   0.603000       0.9570
## PC(38:4)               -0.020300   -0.0993    0.058700      25.2   0.614000       0.9570
## PC(38:6)               -0.024700   -0.1250    0.075700      25.4   0.629000       0.9570
## SM(d41:2)              -0.029200   -0.1500    0.091900      22.5   0.636000       0.9570
## TG(51:1)                0.059200   -0.1900    0.308000      20.3   0.641000       0.9570
## PC(40:6)                0.037000   -0.1200    0.194000      24.0   0.643000       0.9570
## PC(35:1)               -0.032000   -0.1680    0.104000      21.8   0.645000       0.9570
## TG(18:1/18:2/18:2)      0.056600   -0.1900    0.304000      23.6   0.652000       0.9570
## TG(54:2)                0.056100   -0.1890    0.302000      23.4   0.654000       0.9570
## TG(16:0/18:2/18:3)      0.079200   -0.2750    0.434000      22.0   0.661000       0.9570
## TG(51:2)                0.049200   -0.1840    0.283000      21.5   0.679000       0.9570
## SM(d36:2)               0.019600   -0.0749    0.114000      21.4   0.683000       0.9570
## TG(46:1)                0.052000   -0.2100    0.314000      21.2   0.697000       0.9570
## TG(53:4)                0.051700   -0.2150    0.318000      19.7   0.704000       0.9570
## PC(38:2)               -0.022100   -0.1380    0.093800      21.3   0.708000       0.9570
## TG(54:3)                0.027600   -0.1200    0.175000      21.7   0.714000       0.9570
## TG(46:2)                0.049700   -0.2310    0.330000      20.1   0.728000       0.9570
## TG(54:4)               -0.029300   -0.1980    0.139000      21.4   0.733000       0.9570
## TG(54:5)               -0.033900   -0.2410    0.173000      20.6   0.747000       0.9570
## PC(32:2)                0.031300   -0.1680    0.231000      20.7   0.758000       0.9570
## TG(49:1)                0.030600   -0.1710    0.233000      20.9   0.766000       0.9570
## TG(52:4)               -0.025900   -0.2000    0.148000      21.6   0.769000       0.9570
## TG(56:4)                0.041000   -0.2420    0.324000      20.0   0.776000       0.9570
## SM(d36:1)              -0.010700   -0.0989    0.077500      23.4   0.812000       0.9780
## TG(50:1)                0.029100   -0.2350    0.293000      24.3   0.829000       0.9780
## TG(47:1)                0.020700   -0.1990    0.240000      19.5   0.853000       0.9780
## PC(36:5)               -0.021000   -0.2650    0.223000      24.4   0.866000       0.9780
## TG(56:5)                0.016400   -0.1940    0.227000      20.3   0.879000       0.9780
## TG(52:2)                0.007520   -0.1030    0.118000      22.6   0.894000       0.9780
## PC(40:5)                0.009250   -0.1320    0.150000      22.0   0.898000       0.9780
```

```
## SM(d33:1)              0.007250   -0.1070    0.121000      21.3  0.901000      0.9780
## PC(34:2)               0.012200   -0.1910    0.216000      26.0  0.906000      0.9780
## TG(58:9)              -0.021700   -0.4130    0.370000      19.9  0.913000      0.9780
## TG(52:3)              -0.005900   -0.1220    0.110000      22.3  0.921000      0.9780
## TG(18:2/18:1/18:1)     0.009110   -0.1870    0.205000      24.4  0.927000      0.9780
## TG(53:2)               0.012000   -0.2510    0.275000      20.7  0.929000      0.9780
## TG(53:3)              -0.010800   -0.2560    0.235000      20.7  0.931000      0.9780
## SM(42:2)               0.003660   -0.0801    0.087400      25.2  0.932000      0.9780
## TG(51:3)              -0.003930   -0.2420    0.234000      20.7  0.974000      0.9910
## TG(18:1/18:1/18:1)     0.002960   -0.1920    0.197000      24.8  0.976000      0.9910
## TG(18:1/18:1/22:6)     0.004370   -0.3760    0.385000      20.7  0.982000      0.9910
## TG(16:0/22:5/18:1)     0.002120   -0.2010    0.205000      22.7  0.984000      0.9910
## PC(38:3)               0.000719   -0.1280    0.129000      24.0  0.991000      0.9910
##
##
## Table: Results of the crude model for the continuous albuminuria (logUAER) slope.
## Shown in the table are name of the lipid (Name),
## model coefficient (Coefficient),
## its lower and upper confidence intervals (CI.L, CL.R),
## average value of the variable (AveExpr),
## p-value of the coefficient (P.Value) and
## the p-value after correction for multiple testing (adj.P.Val).
## The rows of the table are shown in an increasing order by the respective p-value.
##
## Name                   Coefficient        CI.L        CI.R   AveExpr     P.Value   adj.P.Val
## -------------------  ------------  ----------  ----------  --------  ----------  ----------
## PC(O-34:3)              -5.47e-02   -0.073200   -0.036100      21.9    0.00e+00    1.40e-06
## PC(O-36:3)              -4.66e-02   -0.066700   -0.026500      20.4    6.80e-06    3.62e-04
## PC(O-36:2)              -3.84e-02   -0.056700   -0.020200      20.5    3.96e-05    1.40e-03
## PC(O-34:2)              -4.72e-02   -0.070700   -0.023700      21.5    9.11e-05    2.41e-03
## TG(45:0)                -2.30e-02   -0.035300   -0.010600      23.1    3.00e-04    6.35e-03
## SM(d18:1/24:0)          -2.65e-02   -0.042300   -0.010700      23.6    1.07e-03    1.89e-02
## TG(52:2)                 2.58e-02    0.010100    0.041500      22.6    1.32e-03    2.00e-02
## SM(d41:1)               -2.91e-02   -0.047200   -0.011000      22.7    1.67e-03    2.22e-02
## SM(d40:1)               -2.35e-02   -0.038600   -0.008370      24.0    2.40e-03    2.82e-02
## TG(14:0/18:1/18:1)       4.16e-02    0.013200    0.069900      24.4    4.14e-03    4.13e-02
## TG(50:2)                 3.43e-02    0.010600    0.057900      21.1    4.57e-03    4.13e-02
## PC(16:0e/18:1(9Z)       -2.31e-02   -0.039200   -0.007140      21.8    4.68e-03    4.13e-02
## LPC(18:2)               -3.32e-02   -0.056800   -0.009560      24.0    5.99e-03    4.61e-02
## PC(O-38:6)              -2.15e-02   -0.036900   -0.006170      20.4    6.09e-03    4.61e-02
## TG(56:3)                 4.96e-02    0.013900    0.085400      20.0    6.63e-03    4.69e-02
## TG(49:3)                -5.97e-03   -0.010400   -0.001580      20.7    7.85e-03    5.05e-02
## TG(53:2)                 5.03e-02    0.013100    0.087600      20.7    8.19e-03    5.05e-02
## TG(51:2)                 4.41e-02    0.011100    0.077100      21.5    8.99e-03    5.05e-02
## TG(18:2/22:5/16:0)       6.30e-02    0.015800    0.110000      22.1    9.06e-03    5.05e-02
## TG(54:6)                 5.34e-02    0.012800    0.093900      19.5    9.97e-03    5.17e-02
## TG(56:6)                 3.01e-02    0.007050    0.053200      19.9    1.06e-02    5.17e-02
## TG(54:2)                 4.53e-02    0.010500    0.080000      23.4    1.07e-02    5.17e-02
## TG(18:1/18:1/16:0)       2.74e-02    0.006170    0.048500      25.6    1.15e-02    5.29e-02
## TG(56:7)                 3.32e-02    0.006980    0.059300      19.6    1.32e-02    5.81e-02
## TG(18:2/18:2/18:2)       4.69e-02    0.009230    0.084500      22.1    1.48e-02    6.26e-02
## TG(56:5)                 3.61e-02    0.006300    0.066000      20.3    1.77e-02    7.21e-02
## PC(37:2)                -3.02e-02   -0.055500   -0.005020      19.3    1.89e-02    7.41e-02
## SM(d39:1)               -2.52e-02   -0.046600   -0.003880      21.1    2.07e-02    7.48e-02
```

## PC(36:2)	-1.32e-02	-0.024400	-0.002010	26.1	2.08e-02	7.48e-02
## PC(38:4)	1.32e-02	0.001980	0.024300	25.2	2.12e-02	7.48e-02
## PC(35:2)	-2.28e-02	-0.042300	-0.003280	21.8	2.22e-02	7.58e-02
## TG(50:1)	4.24e-02	0.005050	0.079700	24.3	2.62e-02	8.47e-02
## TG(16:0/22:5/18:1)	3.26e-02	0.003850	0.061400	22.7	2.64e-02	8.47e-02
## TG(50:3)	3.44e-02	0.003410	0.065500	23.4	2.97e-02	9.25e-02
## TG(16:0/18:0/18:1)	5.28e-02	0.004870	0.101000	23.1	3.09e-02	9.36e-02
## TG(54:3)	2.25e-02	0.001580	0.043400	21.7	3.51e-02	1.03e-01
## PC(32:2)	-2.95e-02	-0.057700	-0.001290	20.7	4.04e-02	1.15e-01
## TG(51:1)	3.67e-02	0.001470	0.072000	20.3	4.12e-02	1.15e-01
## PC(40:7)	-2.22e-02	-0.044000	-0.000309	21.4	4.69e-02	1.27e-01
## TG(53:3)	3.44e-02	-0.000368	0.069200	20.7	5.25e-02	1.39e-01
## TG(14:0/16:0/18:1)	3.59e-02	-0.000813	0.072500	22.8	5.53e-02	1.43e-01
## TG(52:3)	1.57e-02	-0.000783	0.032100	22.3	6.19e-02	1.55e-01
## TG(18:1/12:0/18:1)	3.58e-02	-0.001920	0.073600	22.5	6.28e-02	1.55e-01
## SM(d36:2)	1.21e-02	-0.001220	0.025500	21.4	7.49e-02	1.78e-01
## TG(56:4)	3.63e-02	-0.003790	0.076400	20.0	7.59e-02	1.78e-01
## TG(52:4)	2.21e-02	-0.002430	0.046700	21.6	7.73e-02	1.78e-01
## TG(18:0/18:1/20:4)	3.31e-02	-0.004890	0.071200	21.3	8.75e-02	1.97e-01
## SM(d38:1)	-1.23e-02	-0.026500	0.001910	23.1	8.96e-02	1.97e-01
## PC(38:3)	1.57e-02	-0.002520	0.033800	24.0	9.13e-02	1.97e-01
## TG(49:1)	2.42e-02	-0.004330	0.052800	20.9	9.62e-02	2.04e-01
## PC(O-36:5)	-1.29e-02	-0.028500	0.002630	22.3	1.03e-01	2.14e-01
## SM(d34:1)	5.65e-03	-0.001340	0.012600	24.9	1.13e-01	2.28e-01
## LPC(20:4)	1.89e-02	-0.004560	0.042400	21.4	1.14e-01	2.28e-01
## SM(d41:2)	-1.37e-02	-0.030800	0.003430	22.5	1.17e-01	2.29e-01
## TG(49:2)	2.02e-02	-0.005190	0.045600	20.4	1.19e-01	2.29e-01
## TG(50:0)	3.31e-02	-0.009280	0.075400	20.8	1.26e-01	2.36e-01
## TG(51:3)	2.62e-02	-0.007450	0.059800	20.7	1.27e-01	2.36e-01
## PC(32:0)	-8.09e-03	-0.018700	0.002550	22.5	1.36e-01	2.48e-01
## PC(34:2)	2.17e-02	-0.007100	0.050400	26.0	1.40e-01	2.51e-01
## TG(46:2)	2.79e-02	-0.011700	0.067600	20.1	1.67e-01	2.95e-01
## TG(46:1)	2.53e-02	-0.011800	0.062300	21.2	1.81e-01	3.14e-01
## TG(46:0)	2.27e-02	-0.011300	0.056700	20.4	1.90e-01	3.25e-01
## PC(38:6)	-9.29e-03	-0.023500	0.004910	25.4	1.99e-01	3.35e-01
## SM(d40:2)	-8.41e-03	-0.021700	0.004850	23.7	2.13e-01	3.53e-01
## LPC(16:1)	1.39e-02	-0.009360	0.037100	20.0	2.41e-01	3.94e-01
## TG(14:0/18:2/18:2)	2.63e-02	-0.018100	0.070700	21.2	2.45e-01	3.94e-01
## SM(d33:1)	-9.27e-03	-0.025400	0.006890	21.3	2.60e-01	4.11e-01
## TG(16:0/18:2/18:2)	1.90e-02	-0.014400	0.052300	24.5	2.64e-01	4.11e-01
## PC(O-38:5)	-7.95e-03	-0.022600	0.006720	22.8	2.87e-01	4.42e-01
## PC(32:1)	1.67e-02	-0.014600	0.047900	23.3	2.96e-01	4.46e-01
## TG(16:0/18:2/18:3)	2.66e-02	-0.023600	0.076700	22.0	2.99e-01	4.46e-01
## PC(O-38:4)	-1.03e-02	-0.029900	0.009370	21.7	3.05e-01	4.49e-01
## SM(d38:2)	-7.68e-03	-0.022900	0.007550	21.4	3.22e-01	4.68e-01
## TG(48:3)	2.44e-02	-0.026600	0.075500	20.5	3.48e-01	4.98e-01
## PC(40:5)	9.34e-03	-0.010600	0.029300	22.0	3.58e-01	5.06e-01
## TG(18:1/18:1/22:6)	2.47e-02	-0.029100	0.078500	20.7	3.67e-01	5.12e-01
## TG(53:4)	1.70e-02	-0.020700	0.054700	19.7	3.77e-01	5.19e-01
## TG(18:1/18:1/18:1)	1.17e-02	-0.015800	0.039200	24.8	4.04e-01	5.50e-01
## TG(52:5)	1.57e-02	-0.023800	0.055200	19.8	4.34e-01	5.83e-01
## PC(36:5)	-1.26e-02	-0.047100	0.022000	24.4	4.74e-01	6.28e-01
## SM(d32:1)	5.53e-03	-0.010100	0.021200	22.3	4.88e-01	6.38e-01
## TG(54:4)	7.72e-03	-0.016100	0.031600	21.4	5.25e-01	6.79e-01

```
## TG(54:5)                   8.30e-03   -0.020900   0.037500       20.6   5.77e-01    7.37e-01
## TG(18:2/18:1/18:1)         5.40e-03   -0.022400   0.033200       24.4   7.03e-01    8.70e-01
## TG(16:0/18:2/22:6)         1.18e-02   -0.049500   0.073100       21.0   7.05e-01    8.70e-01
## PC(38:2)                  -3.07e-03   -0.019500   0.013300       21.3   7.13e-01    8.70e-01
## LPC(18:1)                  3.32e-03   -0.014700   0.021300       23.7   7.17e-01    8.70e-01
## PC(34:3)                  -5.83e-03   -0.038300   0.026700       22.4   7.25e-01    8.70e-01
## SM(d36:1)                  2.19e-03   -0.010300   0.014700       23.4   7.30e-01    8.70e-01
## SM(d16:1/18:1)             2.20e-03   -0.011700   0.016100       22.8   7.56e-01    8.83e-01
## TG(18:1/18:2/18:2)         5.47e-03   -0.029500   0.040400       23.6   7.58e-01    8.83e-01
## SM(42:2)                  -1.63e-03   -0.013500   0.010200       25.2   7.88e-01    8.94e-01
## PC(36:4)                   1.20e-03   -0.008290   0.010700       26.0   8.04e-01    8.94e-01
## PC(38:5)                  -1.30e-03   -0.011900   0.009310       24.6   8.10e-01    8.94e-01
## TG(47:1)                  -3.70e-03   -0.034800   0.027400       19.5   8.15e-01    8.94e-01
## PC(34:1)                   1.74e-03   -0.013700   0.017200       25.9   8.25e-01    8.94e-01
## TG(58:9)                   6.24e-03   -0.049200   0.061600       19.9   8.25e-01    8.94e-01
## PC(36:3)                  -1.84e-03   -0.018600   0.015000       25.6   8.30e-01    8.94e-01
## LPC(16:0)                  8.10e-04   -0.006960   0.008580       25.3   8.38e-01    8.94e-01
## LPC(18:0)                 -1.41e-03   -0.015500   0.012700       24.0   8.44e-01    8.94e-01
## PC(40:6)                  -1.74e-03   -0.023900   0.020400       24.0   8.78e-01    9.14e-01
## TG(18:2/18:1/16:0)        -1.48e-03   -0.020600   0.017700       25.3   8.79e-01    9.14e-01
## PC(O-36:4)                -1.04e-03   -0.017900   0.015900       22.9   9.04e-01    9.30e-01
## PC(33:1)                   9.94e-04   -0.021700   0.023700       20.9   9.32e-01    9.46e-01
## SM(d18:2/24:1)             5.51e-04   -0.013200   0.014300       24.0   9.37e-01    9.46e-01
## PC(35:1)                  -1.71e-05   -0.019300   0.019300       21.8   9.99e-01    9.99e-01
```

1.2.2.2 Heatmaps

```
##   Label Relation p.adj
## 1     #  p.adj <  0.01
## 2     x  p.adj <  0.05
## 3     *  p.adj <  0.10
```

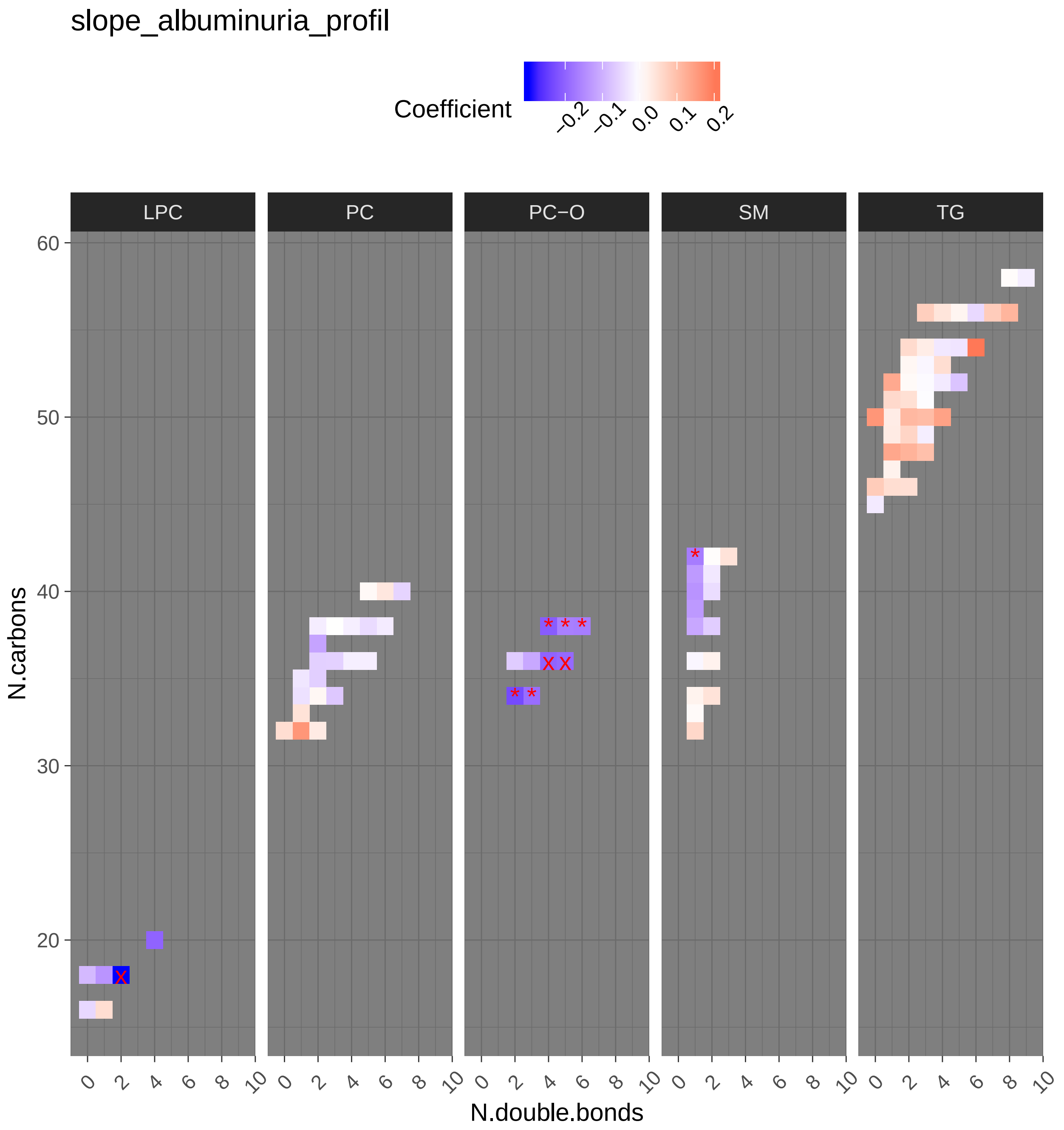

Figure 10: Heatmap of the lipid-specific model coefficents from the crude model for albuminuria (logUAER) slope. The name of the group is shown in the title of the figure in the top-left corner. Lipids are organized into panels (columns) by their class (from LPC to TG). One colored rectangle corresponds to one lipid species, where the coordinates in the x-axis and y-axis, respectively, show the number of double bonds (N.double.bonds) and the number of carbon atoms (N.carbons) in the fatty acid chains of the lipid. The number of double bonds corresponds to the number of unsaturated bonds in the fatty acid chains, and the number of carbon atoms corresponds to the length of the fatty acid chains and, thus, the size of the lipid. The magnitude of the lipid-specifc model coefficient is shown as the color of the rectangle (blue: negative; red: positive). Coefficient with statistical significance are annotated with symbols #, x and *, respectively, corresponding to $p < 0.01$, $p < 0.05$ and $p < 0.1$.

1.3 Combined Heatmaps from Crude Models for Slopes

```
##   Label Relation p.adj
## 1     #  p.adj <  0.01
## 2     x  p.adj <  0.05
## 3     *  p.adj <  0.10
```

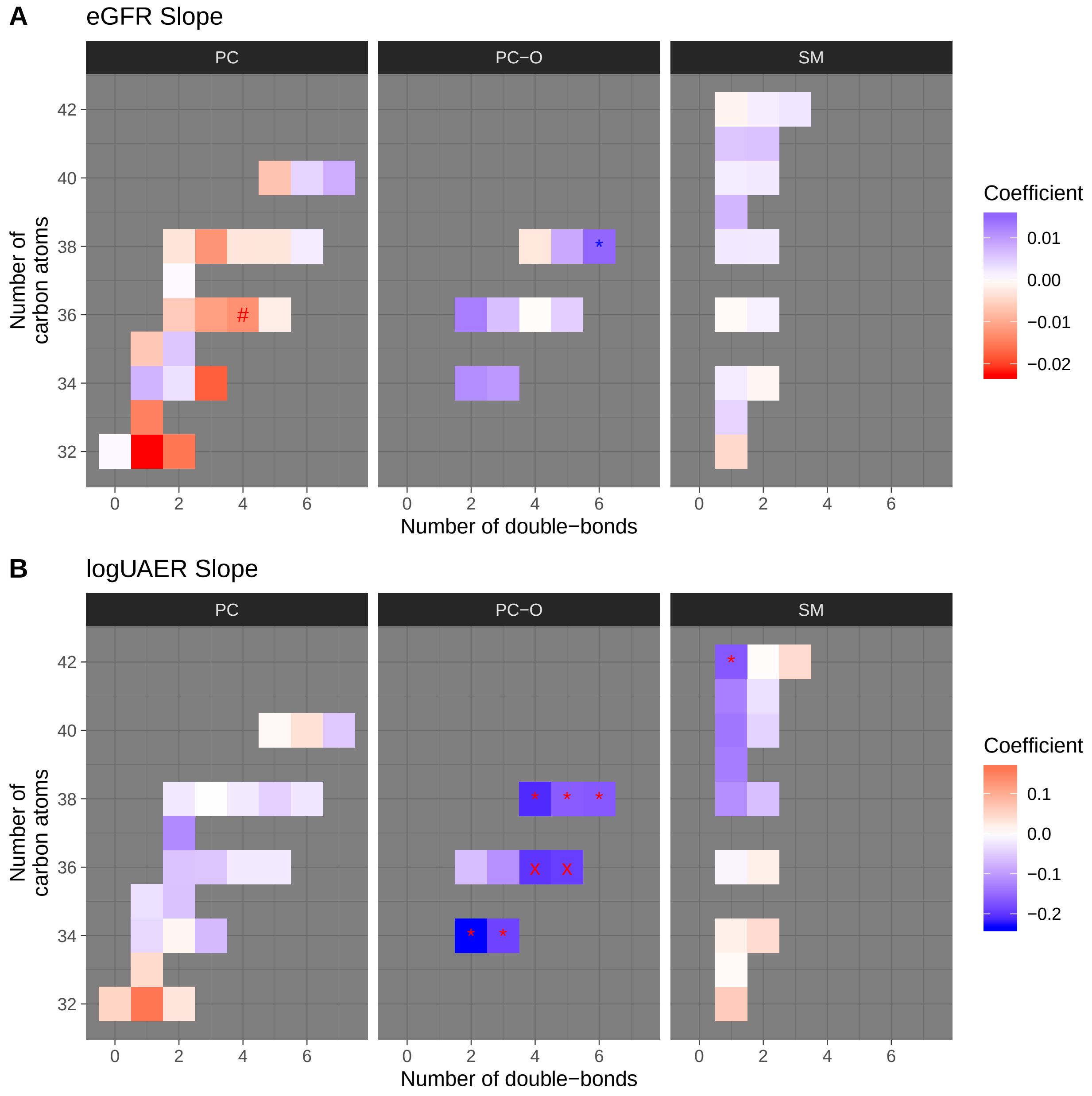

Figure 11: Heatmap of the lipid-specific model coefficents from the crude models for eGFR slope (A) and albuminuria (logUAER) slope (B). The name of the group is shown in the title of the figure in the top-left corner. Lipids are organized into panels (columns) by their class (from PC to SM). One colored rectangle corresponds to one lipid species, where the coordinates in the x-axis and y-axis, respectively, show the number of double bonds (N.double.bonds) and the number of carbon atoms (N.carbons) in the fatty acid chains of the lipid. The number of double bonds corresponds to the number of unsaturated bonds in the fatty acid chains, and the number of carbon atoms corresponds to the length of the fatty acid chains and, thus, the size of the lipid. The magnitude of the lipid-specifc model coefficient is shown as the color of the rectangle (blue: negative; red: positive). Coefficient with statistical significance are annotated with symbols #, x and *, respectively, corresponding to $p < 0.01$, $p < 0.05$ and $p < 0.1$.

2 Step 2: Adjusted Associations to Clinical Changes Over Time with Prioritized Lipids from Steps 1A-B

2.1 Step 2A: Adjusted Model for Prioritized Lipids from the Adjusted Cross-Sectional Models

2.1.1 Albuminuria Slope

2.1.1.1 Table

```
##
##
## Table: Results of the adjusted model for the continuous albuminuria (logUAER) slope
## and with lipids prioritized from the cross-sectional models.
## Shown in the table are name of the lipid (Name),
## model coefficient (Coefficient),
## its lower and upper confidence intervals (CI.L, CL.R),
## average value of the variable (AveExpr),
## p-value of the coefficient (P.Value) and
## the p-value after correction for multiple testing (adj.P.Val).
## The rows of the table are shown in an increasing order by the respective p-value.
##
## Name                    Coefficient     CI.L      CI.R   AveExpr    P.Value   adj.P.Val
## -------------------  ------------  -------  --------  --------  ---------  ----------
## SM(d38:1)                  -0.195   -0.281   -0.1090      23.1   1.11e-05    6.67e-05
## SM(d40:1)                  -0.187   -0.272   -0.1010      24.0   2.36e-05    7.07e-05
## SM(d18:1/24:0)             -0.193   -0.286   -0.0992      23.6   5.93e-05    1.19e-04
## SM(d41:1)                  -0.196   -0.307   -0.0837      22.7   6.41e-04    9.62e-04
## PC(O-34:3)                 -0.161   -0.283   -0.0389      21.9   9.87e-03    1.18e-02
## TG(18:0/18:1/20:4)         -0.314   -0.563   -0.0656      21.3   1.33e-02    1.33e-02
```

2.1.1.2 Heatmaps

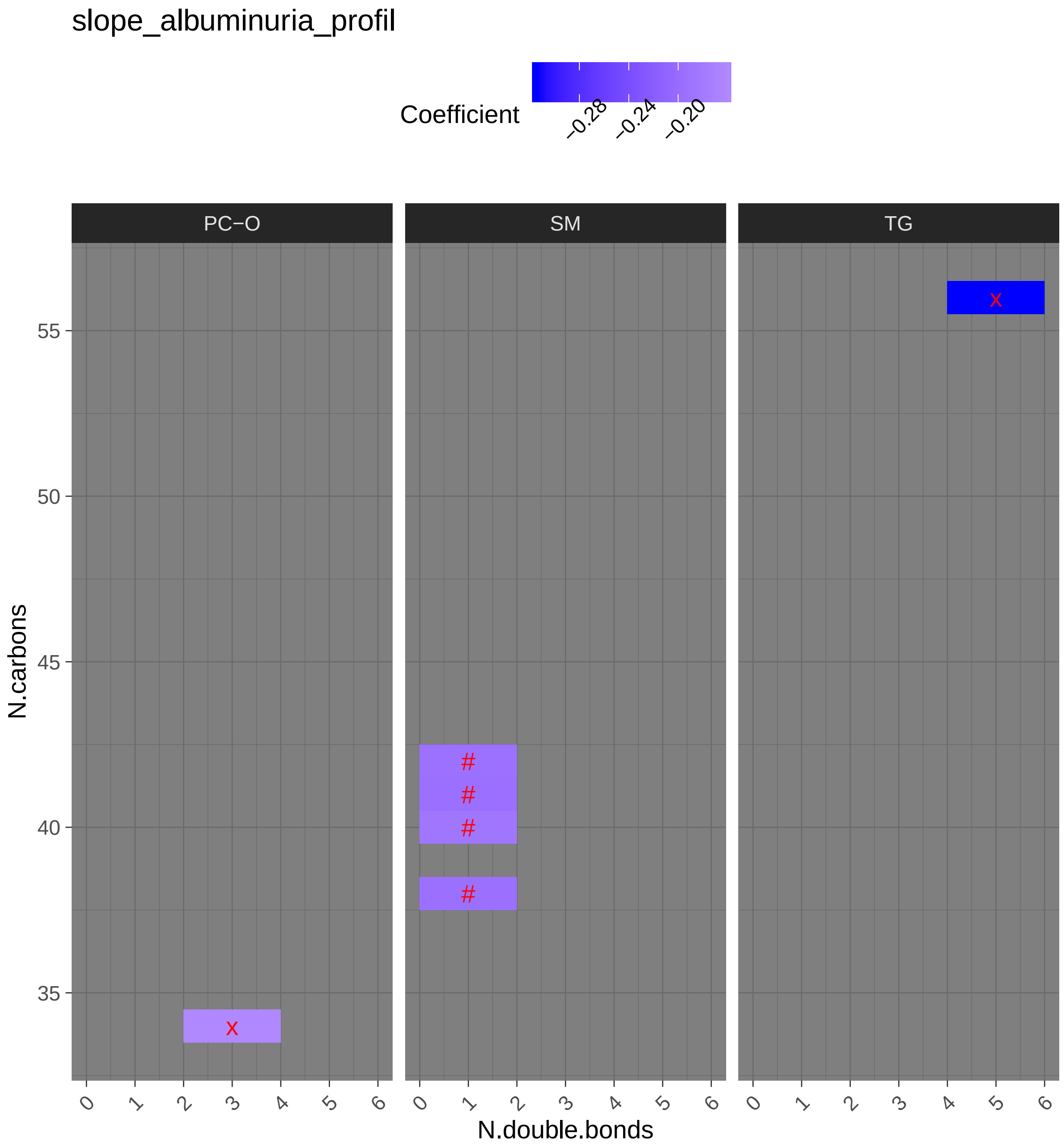


```
##   Label Relation p.adj
## 1     #  p.adj <  0.01
## 2     x  p.adj <  0.05
## 3     *  p.adj <  0.10
```

2.1.2 eGFR Slope

2.1.2.1 Table

```
##
##
## Table: Results of the adjusted model for the eGFR slope
## with lipids prioritized from the cross-sectional models.
## Shown in the table are name of the lipid (Name),
## model coefficient (Coefficient),
## its lower and upper confidence intervals (CI.L, CL.R),
## average value of the variable (AveExpr),
## p-value of the coefficient (P.Value) and
## the p-value after correction for multiple testing (adj.P.Val).
## The rows of the table are shown in an increasing order by the respective p-value.
##
## Name                   Coefficient       CI.L       CI.R   AveExpr   P.Value   adj.P.Val
## -------------------  ------------  ---------  ---------  --------  --------  ----------
## PC(32:2)                -0.016700   -0.03410   0.000693      20.7    0.0598       0.860
## LPC(16:0)               -0.004370   -0.00929   0.000556      25.3    0.0819       0.860
## TG(14:0/16:0/18:1)      -0.012700   -0.03000   0.004700      22.8    0.1530       0.984
## PC(32:1)                -0.012700   -0.03220   0.006850      23.3    0.2030       0.984
## LPC(16:1)               -0.008500   -0.02380   0.006850      20.0    0.2770       0.984
## SM(d34:1)                0.002180   -0.00218   0.006540      24.9    0.3260       0.984
## SM(d33:1)                0.004260   -0.00526   0.013800      21.3    0.3800       0.984
## PC(40:6)                 0.004770   -0.00845   0.018000      24.0    0.4790       0.984
## PC(O-36:2)               0.003420   -0.00813   0.015000      20.5    0.5610       0.984
## TG(18:2/18:2/18:2)      -0.006130   -0.02720   0.014900      22.1    0.5680       0.984
## PC(38:6)                 0.001860   -0.00682   0.010500      25.4    0.6730       0.984
## PC(O-38:4)              -0.002850   -0.01650   0.010800      21.7    0.6810       0.984
## TG(16:0/18:2/22:6)       0.005660   -0.03300   0.044300      21.0    0.7730       0.984
## PC(40:7)                 0.001840   -0.01130   0.015000      21.4    0.7840       0.984
## SM(d16:1/18:1)          -0.000797   -0.00778   0.006190      22.8    0.8230       0.984
## SM(d32:1)               -0.000827   -0.00982   0.008160      22.3    0.8570       0.984
## TG(18:1/18:1/22:6)      -0.002700   -0.03710   0.031700      20.7    0.8780       0.984
## SM(d18:2/24:1)           0.000381   -0.00745   0.008220      24.0    0.9240       0.984
## TG(58:9)                 0.000924   -0.03490   0.036800      19.9    0.9600       0.984
## TG(18:2/22:5/16:0)       0.000334   -0.02860   0.029300      22.1    0.9820       0.984
## PC(36:5)                 0.000226   -0.02140   0.021900      24.4    0.9840       0.984
```

2.1.2.2 Heatmaps

```
##   Label Relation p.adj
## 1     #  p.adj <  0.01
## 2     x  p.adj <  0.05
## 3     *  p.adj <  0.10
```

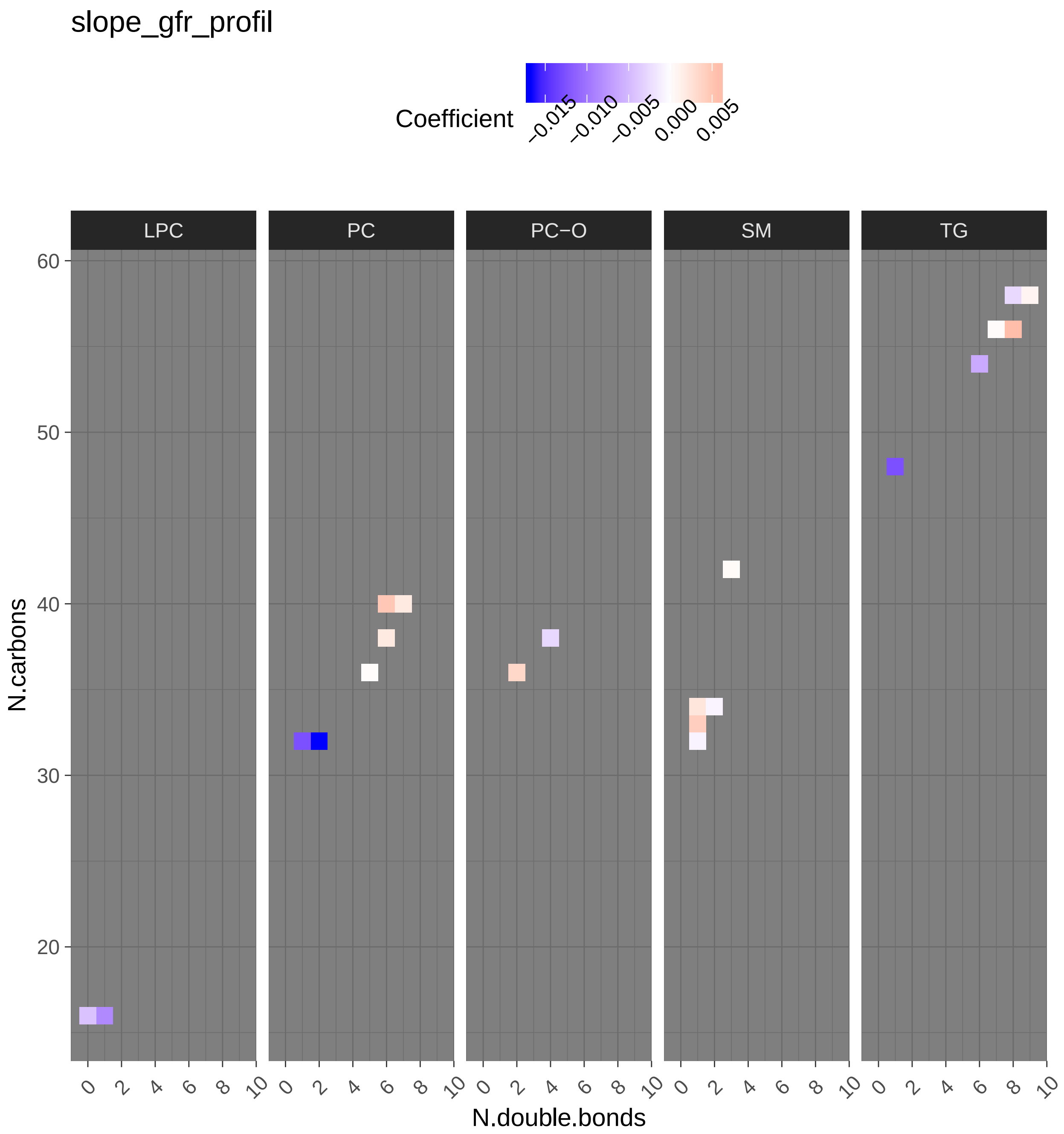

Figure 12: Heatmap of the lipid-specific model coefficents from the adjusted model for eGFR slope with lipids prioritized from the cross-sectional models. Lipids are organized into panels (columns) by their class (from LPC to TG). One colored rectangle corresponds to one lipid species, where the coordinates in the x-axis and y-axis, respectively, show the number of double bonds (N.double.bonds) and the number of carbon atoms (N.carbons) in the fatty acid chains of the lipid. The number of double bonds corresponds to the number of unsaturated bonds in the fatty acid chains, and the number of carbon atoms corresponds to the length of the fatty acid chains and, thus, the size of the lipid. The magnitude of the lipid-specifc model coefficient is shown as the color of the rectangle (blue: negative; red: positive). Coefficient with statistical significance are annotated with symbols #, x and *, respectively, corresponding to $p < 0.01$, $p < 0.05$ and $p < 0.1$.

2.2 Step 2B: Adjusted Model for Prioritized Lipids from the Crude Models for Slopes

2.2.1 eGFR Slope

2.2.1.1 Table

```
##
##
## Table: Results of the adjusted model for eGFR slope
## with the lipids prioritized from the crude slope models.
## Shown in the table are name of the lipid (Name),
## model coefficient (Coefficient),
## its lower and upper confidence intervals (CI.L, CL.R),
## average value of the variable (AveExpr),
## p-value of the coefficient (P.Value) and
## the p-value after correction for multiple testing (adj.P.Val).
## The rows of the table are shown in an increasing order by the respective p-value.
##
## Name                  Coefficient       CI.L        CI.R   AveExpr    P.Value   adj.P.Val
## -------------------  ------------  ---------  ----------  --------  ---------  ----------
## PC(36:4)                 -0.01400   -0.02060   -0.007370      26.0   3.79e-05    0.000455
## LPC(16:0)                -0.00437   -0.00931    0.000576      25.3   8.32e-02    0.305000
## PC(O-38:6)                0.00823   -0.00177    0.018200      20.4   1.06e-01    0.305000
## TG(51:3)                 -0.01250   -0.02780    0.002810      20.7   1.09e-01    0.305000
## TG(46:0)                 -0.01470   -0.03360    0.004220      20.4   1.28e-01    0.305000
## TG(14:0/16:0/18:1)       -0.01270   -0.03000    0.004690      22.8   1.52e-01    0.305000
## TG(51:2)                 -0.00629   -0.01980    0.007190      21.5   3.60e-01    0.617000
## TG(50:0)                 -0.00680   -0.02940    0.015800      20.8   5.54e-01    0.691000
## TG(18:2/18:2/18:2)       -0.00613   -0.02720    0.014900      22.1   5.68e-01    0.691000
## TG(51:1)                 -0.00463   -0.02090    0.011600      20.3   5.76e-01    0.691000
## TG(50:2)                 -0.00140   -0.01110    0.008270      21.1   7.76e-01    0.830000
## TG(14:0/18:1/18:1)       -0.00122   -0.01240    0.009910      24.4   8.30e-01    0.830000
```

2.2.1.2 Heatmaps

```
##   Label Relation p.adj
## 1     #  p.adj <  0.01
## 2     x  p.adj <  0.05
## 3     *  p.adj <  0.10
```

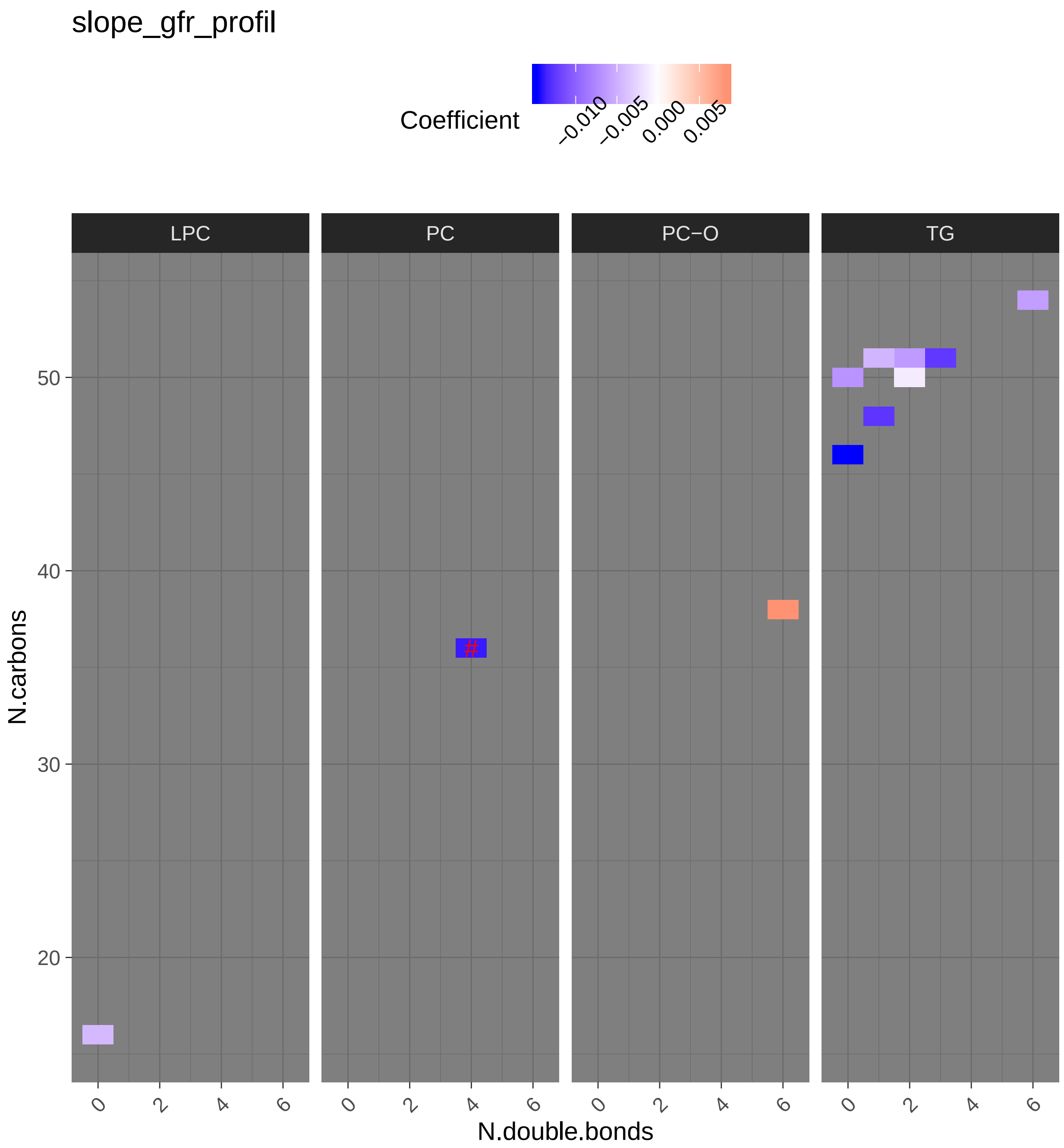

Figure 13: Heatmap of the lipid-specific model coefficents from the adjusted model for eGFR slope with lipids prioritized from the crude slope models. Lipids are organized into panels (columns) by their class (from LPC to TG). One colored rectangle corresponds to one lipid species, where the coordinates in the x-axis and y-axis, respectively, show the number of double bonds (N.double.bonds) and the number of carbon atoms (N.carbons) in the fatty acid chains of the lipid. The number of double bonds corresponds to the number of unsaturated bonds in the fatty acid chains, and the number of carbon atoms corresponds to the length of the fatty acid chains and, thus, the size of the lipid. The magnitude of the lipid-specifc model coefficient is shown as the color of the rectangle (blue: negative; red: positive). Coefficient with statistical significance are annotated with symbols #, x and *, respectively, corresponding to $p < 0.01$, $p < 0.05$ and $p < 0.1$.

2.2.2 Albuminuria Slope

2.2.2.1 Table

```
##
##
## Table: Results of the adjusted model for the continuous albuminuria (logUAER) slope
## with the lipids prioritized from the crude slope models.
## Shown in the table are name of the lipid (Name),
## model coefficient (Coefficient),
## its lower and upper confidence intervals (CI.L, CL.R),
## average value of the variable (AveExpr),
## p-value of the coefficient (P.Value) and
## the p-value after correction for multiple testing (adj.P.Val).
## The rows of the table are shown in an increasing order by the respective p-value.
##
## Name               Coefficient     CI.L      CI.R   AveExpr    P.Value   adj.P.Val
## ---------------  ------------  -------  --------  --------  ---------  ----------
## PC(O-38:6)             -0.229   -0.335   -0.1220      20.4   2.94e-05    0.000203
## PC(O-36:5)             -0.243   -0.359   -0.1270      22.3   4.66e-05    0.000203
## SM(d18:1/24:0)         -0.193   -0.287   -0.0984      23.6   6.75e-05    0.000203
## PC(O-36:4)             -0.225   -0.351   -0.0987      22.9   5.08e-04    0.001140
## PC(O-38:4)             -0.249   -0.394   -0.1050      21.7   7.28e-04    0.001310
## PC(O-38:5)             -0.173   -0.282   -0.0645      22.8   1.85e-03    0.002770
## PC(O-34:3)             -0.161   -0.284   -0.0388      21.9   9.94e-03    0.012800
## PC(O-34:2)             -0.188   -0.356   -0.0205      21.5   2.80e-02    0.031500
## LPC(18:2)              -0.134   -0.307    0.0387      24.0   1.28e-01    0.128000
```

2.2.2.2 Heatmaps

```
##   Label Relation p.adj
## 1     #  p.adj <  0.01
## 2     x  p.adj <  0.05
## 3     *  p.adj <  0.10
```

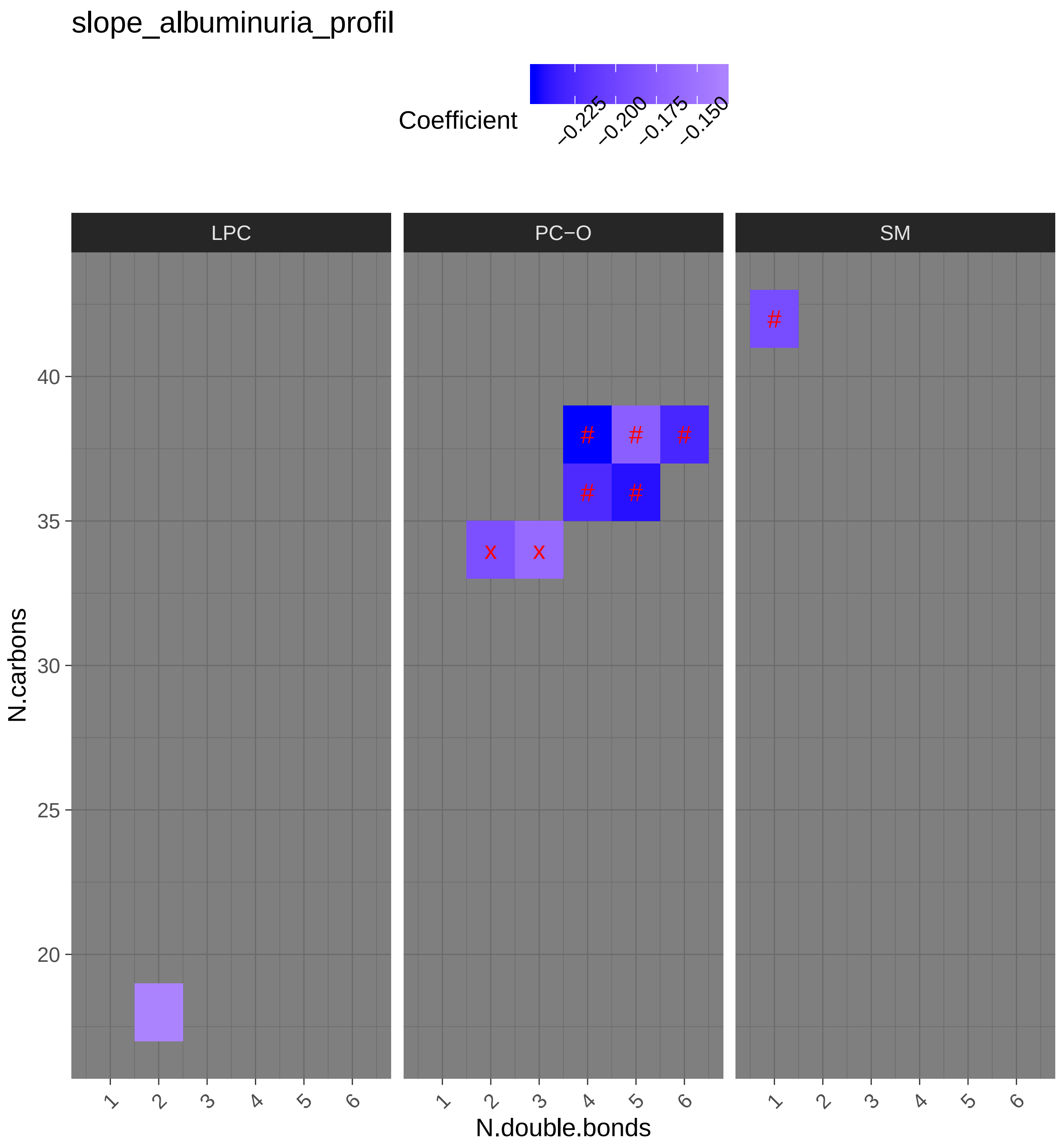

Figure 14: Heatmap of the lipid-specific model coefficents from the adjusted model for albuminuria (logUAER) slope with lipids prioritized from the crude slope models. Lipids are organized into panels (columns) by their class (from LPC to TG). One colored rectangle corresponds to one lipid species, where the coordinates in the x-axis and y-axis, respectively, show the number of double bonds (N.double.bonds) and the number of carbon atoms (N.carbons) in the fatty acid chains of the lipid. The number of double bonds corresponds to the number of unsaturated bonds in the fatty acid chains, and the number of carbon atoms corresponds to the length of the fatty acid chains and, thus, the size of the lipid. The magnitude of the lipid-specifc model coefficient is shown as the color of the rectangle (blue: negative; red: positive). Coefficient with statistical significance are annotated with symbols #, x and *, respectively, corresponding to $p < 0.01$, $p < 0.05$ and $p < 0.1$.

3 Step 3: Survival Analysis of Combined Renal Endpoint in Relation to Prioritized Lipids from Step 2A-B

3.1 Step 3A: Crude Model

```
## Warning in lapply(X = x, FUN = .Generic, ...): NaNs produced
```

3.1.1 Table

Table 1: Crude survival model for the combined renal endpoint. Shown in the table are the name of the lipid (Name), the hazard ratio (exp(coef)) and its lower and upper condidence intervals (lower .95 and upper .95, respectively), p-value of the coefficient (Pr(>|z|), and the p-value after correction for multiple testing (p.adj).

Name	exp(coef)	lower .95	upper .95	Pr(>|z|)	p.adj
PC(O-34:3)	0.404	0.289	0.566	1.00e-07	1.60e-06
PC(O-34:2)	0.583	0.441	0.770	1.47e-04	1.76e-03
SM(d41:1)	0.533	0.383	0.742	1.91e-04	2.10e-03
SM(d40:1)	0.472	0.308	0.724	5.79e-04	5.79e-03
SM(d18:1/24:0)	0.525	0.347	0.795	2.31e-03	2.08e-02
TG(18:0/18:1/20:4)	1.250	1.030	1.530	2.41e-02	1.93e-01
SM(d38:1)	0.615	0.384	0.983	4.22e-02	2.95e-01
PC(O-38:4)	0.738	0.526	1.040	7.83e-02	4.70e-01
PC(O-36:5)	0.704	0.466	1.060	9.53e-02	4.76e-01
PC(O-38:6)	0.737	0.483	1.120	1.57e-01	6.29e-01
PC(O-36:4)	0.791	0.535	1.170	2.40e-01	7.19e-01
PC(36:4)	1.620	0.709	3.700	2.52e-01	7.19e-01
PC(O-38:5)	0.866	0.548	1.370	5.37e-01	7.19e-01

3.1.2 Heatmaps

```
##   Label Relation p.adj
## 1     #  p.adj <  0.01
## 2     x  p.adj <  0.05
## 3     *  p.adj <  0.10
```

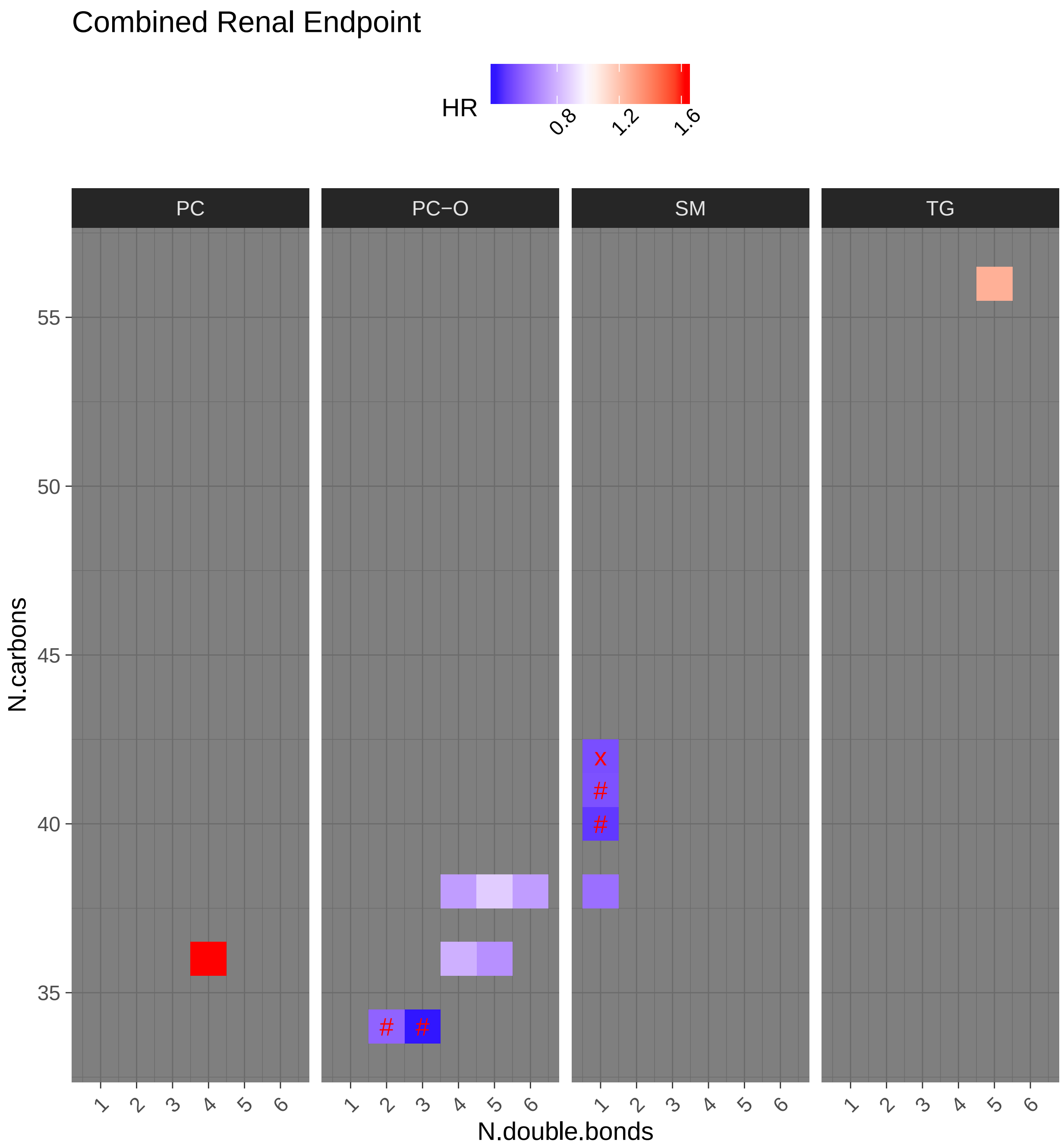

Figure 15: Heatmap of the lipid-specific hazard ratios (HRs) from the crude survival model for the combined renal endpoint. Lipids are organized into panels (columns) by their class (from PC to TG). One colored rectangle corresponds to one lipid species, where the coordinates in the x-axis and y-axis, respectively, show the number of double bonds (N.double.bonds) and the number of carbon atoms (N.carbons) in the fatty acid chains of the lipid. The number of double bonds corresponds to the number of unsaturated bonds in the fatty acid chains, and the number of carbon atoms corresponds to the length of the fatty acid chains and, thus, the size of the lipid. The magnitude of the lipid-specifc hazard ratio is shown as the color of the rectangle (blue: inverse association; red: positive association). Associations with statistical significance are annotated with symbols #, x and *, respectively, corresponding to $p < 0.01$, $p < 0.05$ and $p < 0.1$.

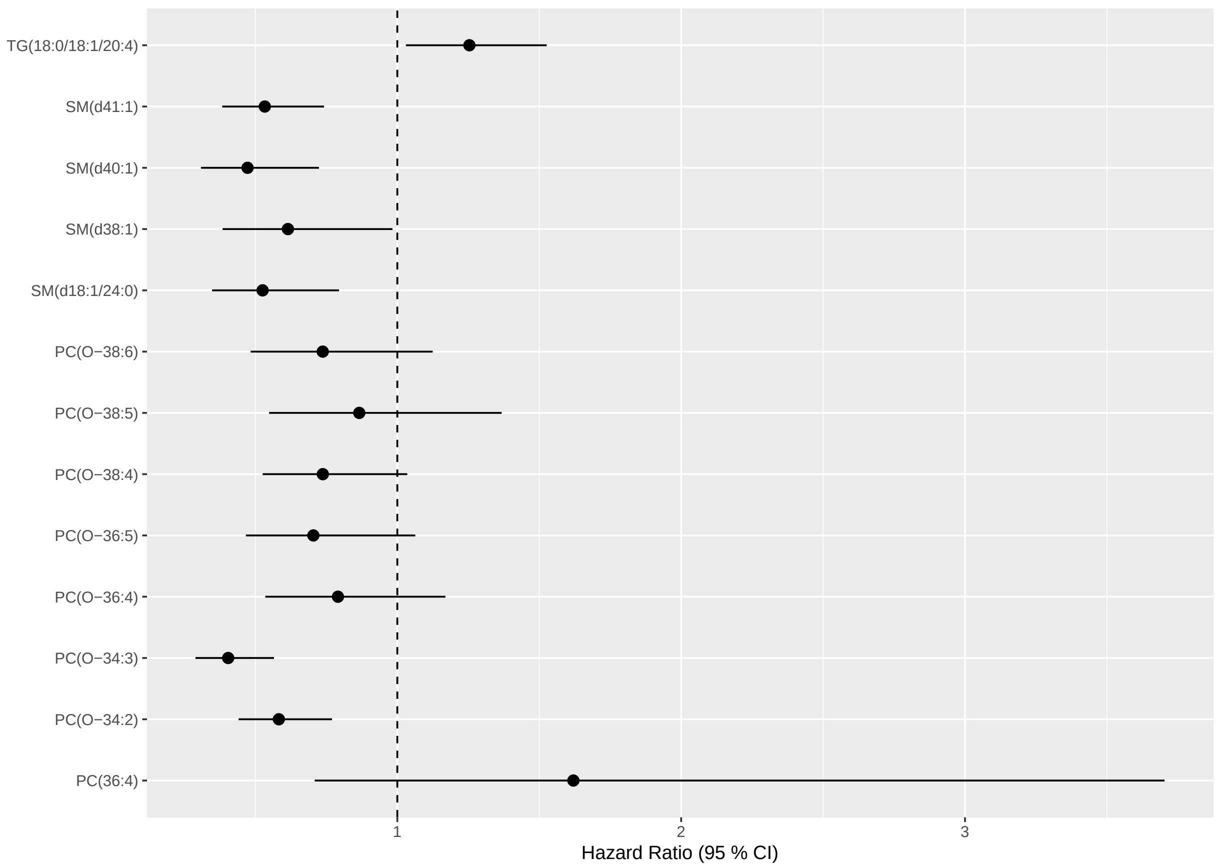

Figure 16: Forest plot of hazard ratios from the crude survival model for the combined renal event.

3.1.3 Forest Plot

3.2 Step 3B: Adjusted Model

```
## Warning in lapply(X = x, FUN = .Generic, ...): NaNs produced
```

3.2.1 Table

Table 2: Adjusted survival model for the combined renal endpoint. Shown in the table are the name of the lipid (Name), the hazard ratio (exp(coef)) and its lower and upper condidence intervals (lower .95 and upper .95, respectively), p-value of the coefficient (Pr(>|z|), and the p-value after correction for multiple testing (p.adj).

Name	exp(coef)	lower .95	upper .95	Pr(>|z|)	p.adj
SM(d38:1)	0.399	0.225	0.709	0.00173	0.0225
SM(d41:1)	0.490	0.303	0.791	0.00348	0.0418
SM(d40:1)	0.433	0.242	0.774	0.00477	0.0525
PC(O-34:3)	0.561	0.355	0.886	0.01310	0.1310
PC(O-38:4)	0.618	0.418	0.915	0.01620	0.1460
PC(O-34:2)	0.680	0.489	0.945	0.02170	0.1740
PC(O-36:4)	0.703	0.448	1.100	0.12500	0.8720
SM(d18:1/24:0)	0.682	0.395	1.180	0.17000	1.0000
PC(O-38:5)	0.742	0.437	1.260	0.26700	1.0000
TG(18:0/18:1/20:4)	0.914	0.722	1.160	0.45300	1.0000
PC(O-36:5)	0.835	0.513	1.360	0.47000	1.0000
PC(O-38:6)	0.898	0.533	1.510	0.68600	1.0000
PC(36:4)	1.120	0.545	2.300	0.75800	1.0000

3.2.2 Heatmaps

```
##   Label Relation p.adj
## 1     #  p.adj <  0.01
## 2     x  p.adj <  0.05
## 3     *  p.adj <  0.10
```

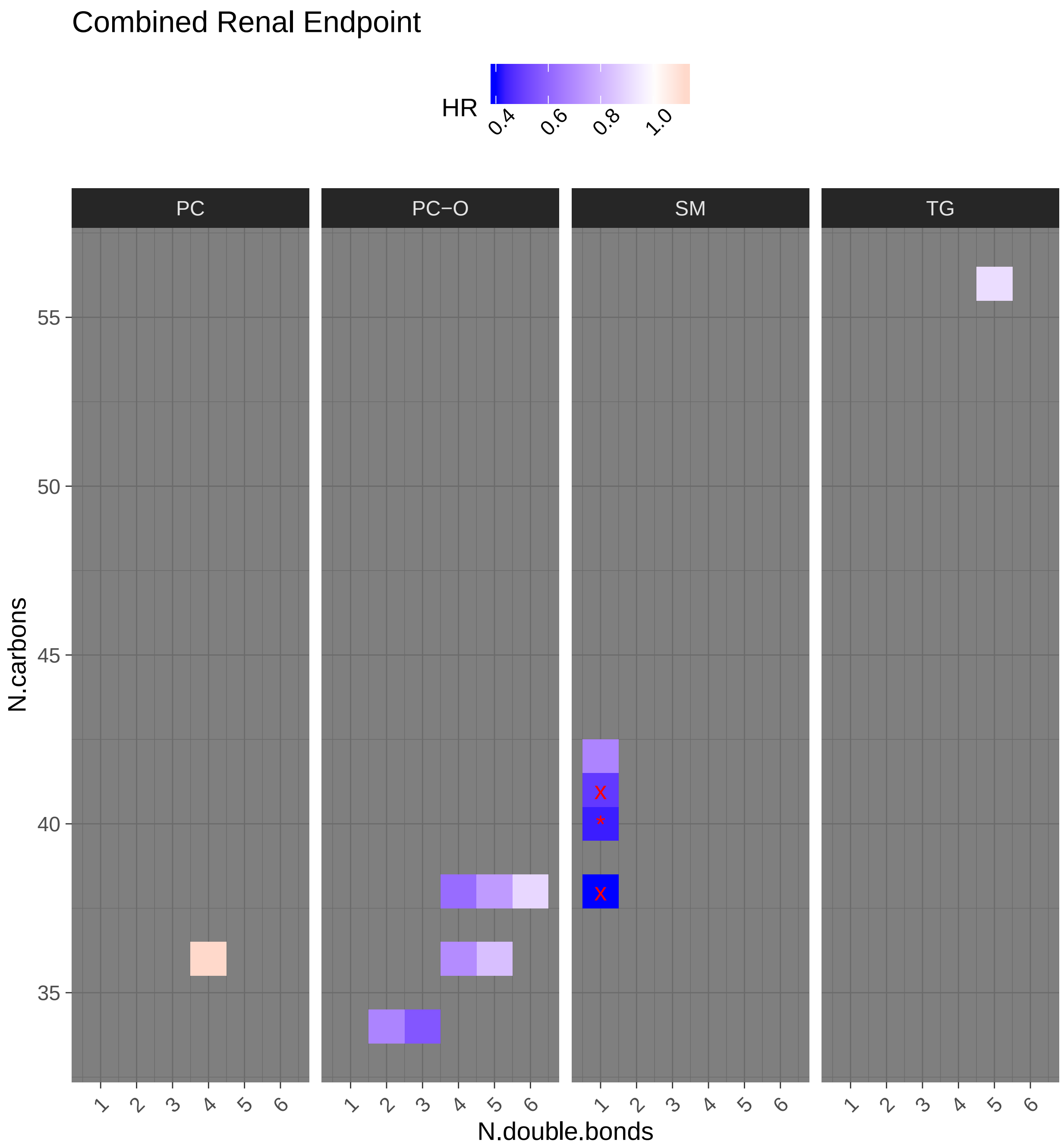

Figure 17: Heatmap of the lipid-specific hazard ratios (HRs) from the adjusted survival model for the combined renal endpoint. Lipids are organized into panels (columns) by their class (from PC to TG). One colored rectangle corresponds to one lipid species, where the coordinates in the x-axis and y-axis, respectively, show the number of double bonds (N.double.bonds) and the number of carbon atoms (N.carbons) in the fatty acid chains of the lipid. The number of double bonds corresponds to the number of unsaturated bonds in the fatty acid chains, and the number of carbon atoms corresponds to the length of the fatty acid chains and, thus, the size of the lipid. The magnitude of the lipid-specifc hazard ratio is shown as the color of the rectangle (blue: inverse association; red: positive association). Associations with statistical significance are annotated with symbols #, x and *, respectively, corresponding to $p < 0.01$, $p < 0.05$ and $p < 0.1$.

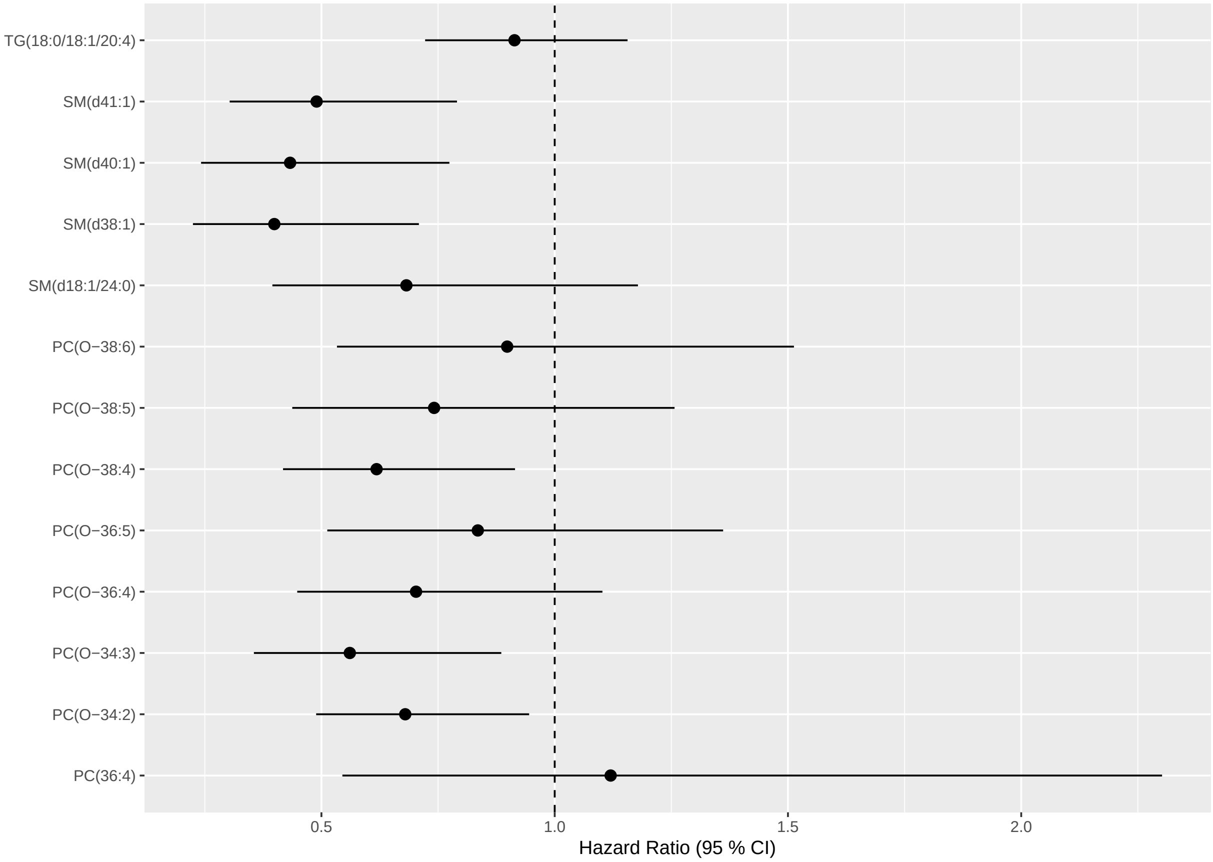

Figure 18: Forest plot of hazard ratios from the adjusted survival model for the combined renal event.

3.2.3 Forest Plot

3.3 Combined Forest Plot from Crude and Adjusted Models

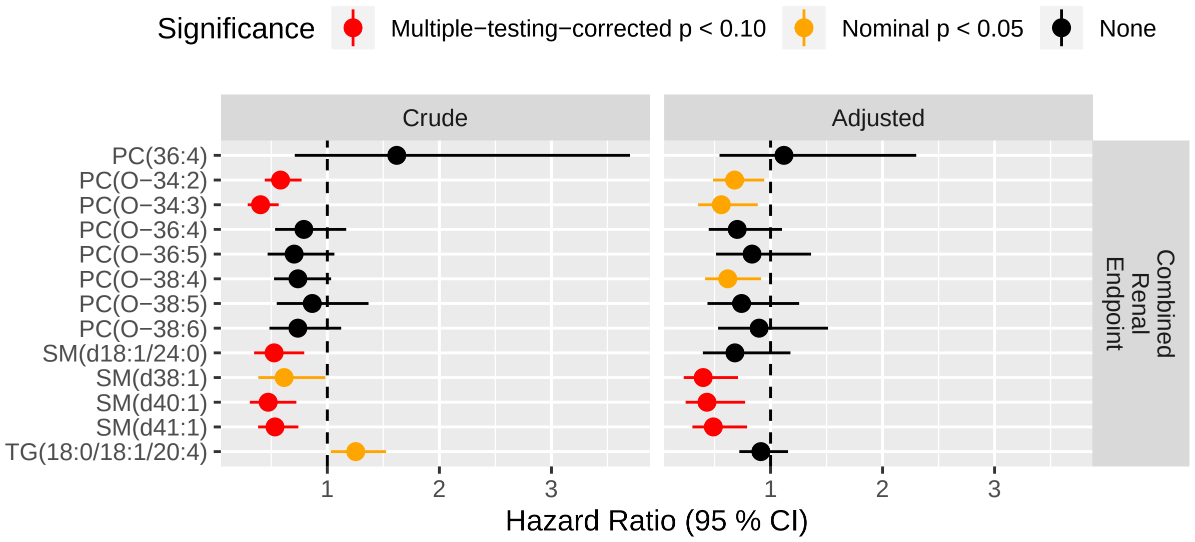

4 Step 4: Survival Analysis of Specific Renal Endpoints in Relation to Prioritized Lipids from Step 3A

4.1 Step 4A: Albuminuria Group Progression

4.1.1 Crude Model

4.1.1.1 Table

Table 3: Crude survival model for albuminuria group progression. Shown in the table are the name of the lipid (Name), the hazard ratio (exp(coef)) and its lower and upper condidence intervals (lower .95 and upper .95, respectively), p-value of the coefficient (Pr(>|z|), and the p-value after correction for multiple testing (p.adj).

Name	exp(coef)	lower .95	upper .95	Pr(>|z|)	p.adj
SM(d18:1/24:0)	0.516	0.236	1.13	0.0981	0.491
SM(d40:1)	0.601	0.267	1.35	0.2190	0.874
SM(d41:1)	0.727	0.376	1.41	0.3440	1.000
PC(O-34:2)	0.873	0.517	1.47	0.6100	1.000
PC(O-34:3)	0.888	0.464	1.70	0.7200	1.000

4.1.1.2 Heatmaps

```
##   Label Relation p.adj
## 1     #  p.adj <  0.01
## 2     x  p.adj <  0.05
## 3     *  p.adj <  0.10
```

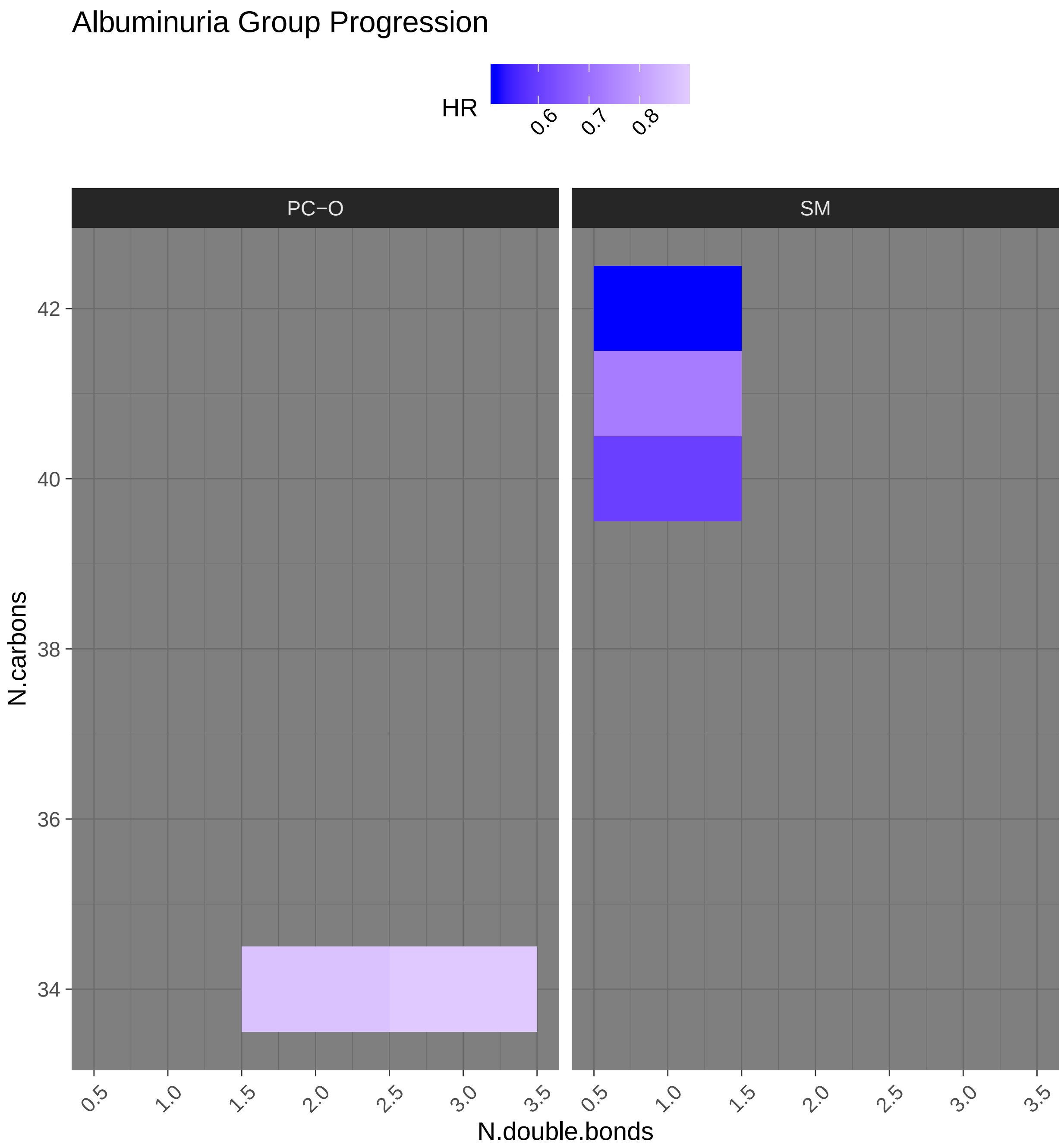

Figure 19: Heatmap of the lipid-specific hazard ratios (HRs) from the crude survival model for the albuminuria group progression. Lipids are organized into panels (columns) by their class (from PC-O to SM). One colored rectangle corresponds to one lipid species, where the coordinates in the x-axis and y-axis, respectively, show the number of double bonds (N.double.bonds) and the number of carbon atoms (N.carbons) in the fatty acid chains of the lipid. The number of double bonds corresponds to the number of unsaturated bonds in the fatty acid chains, and the number of carbon atoms corresponds to the length of the fatty acid chains and, thus, the size of the lipid. The magnitude of the lipid-specifc hazard ratio is shown as the color of the rectangle (blue: inverse association; red: positive association). Associations with statistical significance are annotated with symbols #, x and *, respectively, corresponding to $p < 0.01$, $p < 0.05$ and $p < 0.1$.

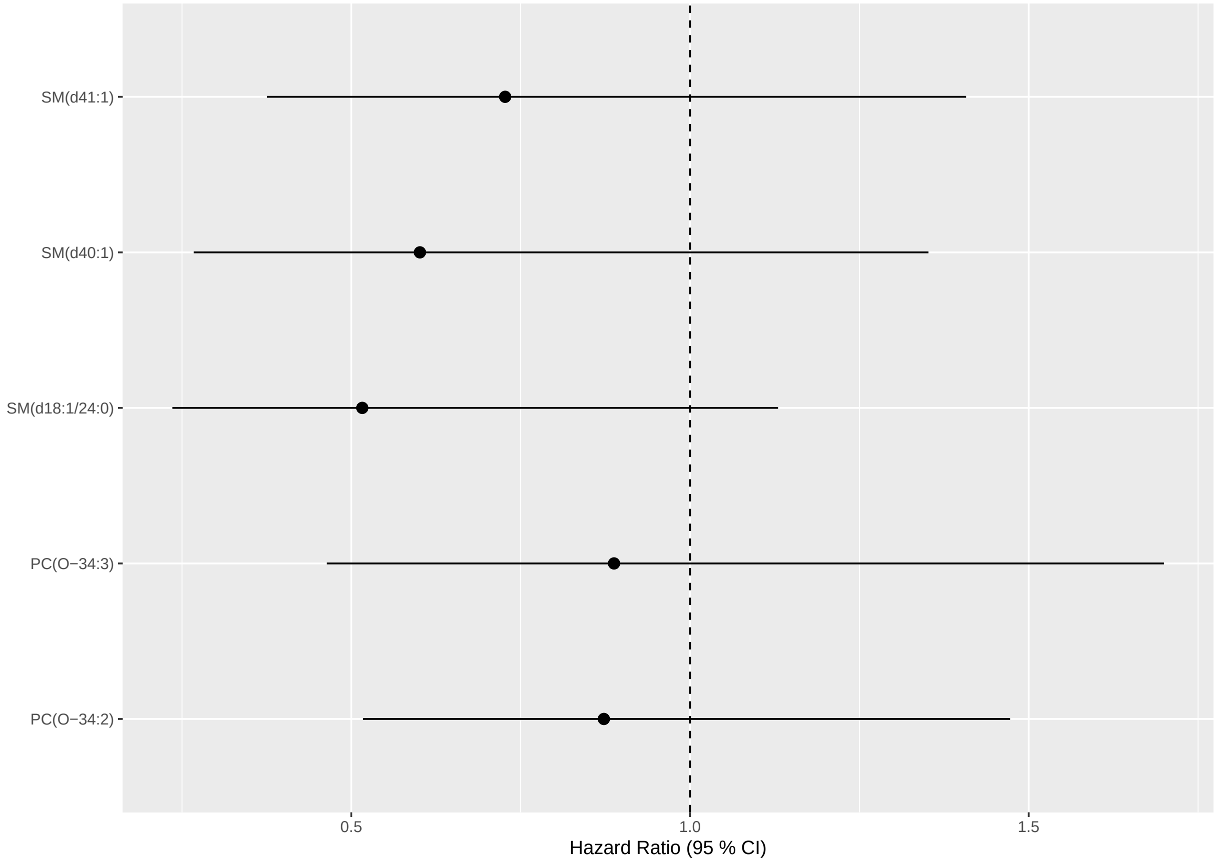

Figure 20: Forest plot of hazard ratios from the crude survival model for the albuminuria group progression. The hazard ratio and its 95 % confidence intervals (x-axis) are shown for each lipid (rows).

4.1.1.3 Forest Plot

4.1.2 Adjusted Model

4.1.2.1 Table

Table 4: Adjusted survival model for albuminuria group progression. Shown in the table are the name of the lipid (Name), the hazard ratio (exp(coef)) and its lower and upper condidence intervals (lower .95 and upper .95, respectively), p-value of the coefficient (Pr(>|z|), and the p-value after correction for multiple testing (p.adj).

Name	exp(coef)	lower .95	upper .95	Pr(>|z|)	p.adj
SM(d18:1/24:0)	0.270	0.0904	0.808	0.0191	0.0957
SM(d40:1)	0.286	0.0884	0.924	0.0365	0.1460
SM(d41:1)	0.480	0.1990	1.160	0.1040	0.3110
PC(O-34:3)	0.740	0.2960	1.850	0.5200	1.0000
PC(O-34:2)	0.854	0.4420	1.650	0.6400	1.0000

4.1.2.2 Heatmaps

```
##   Label Relation p.adj
## 1     #  p.adj <  0.01
## 2     x  p.adj <  0.05
## 3     *  p.adj <  0.10
```

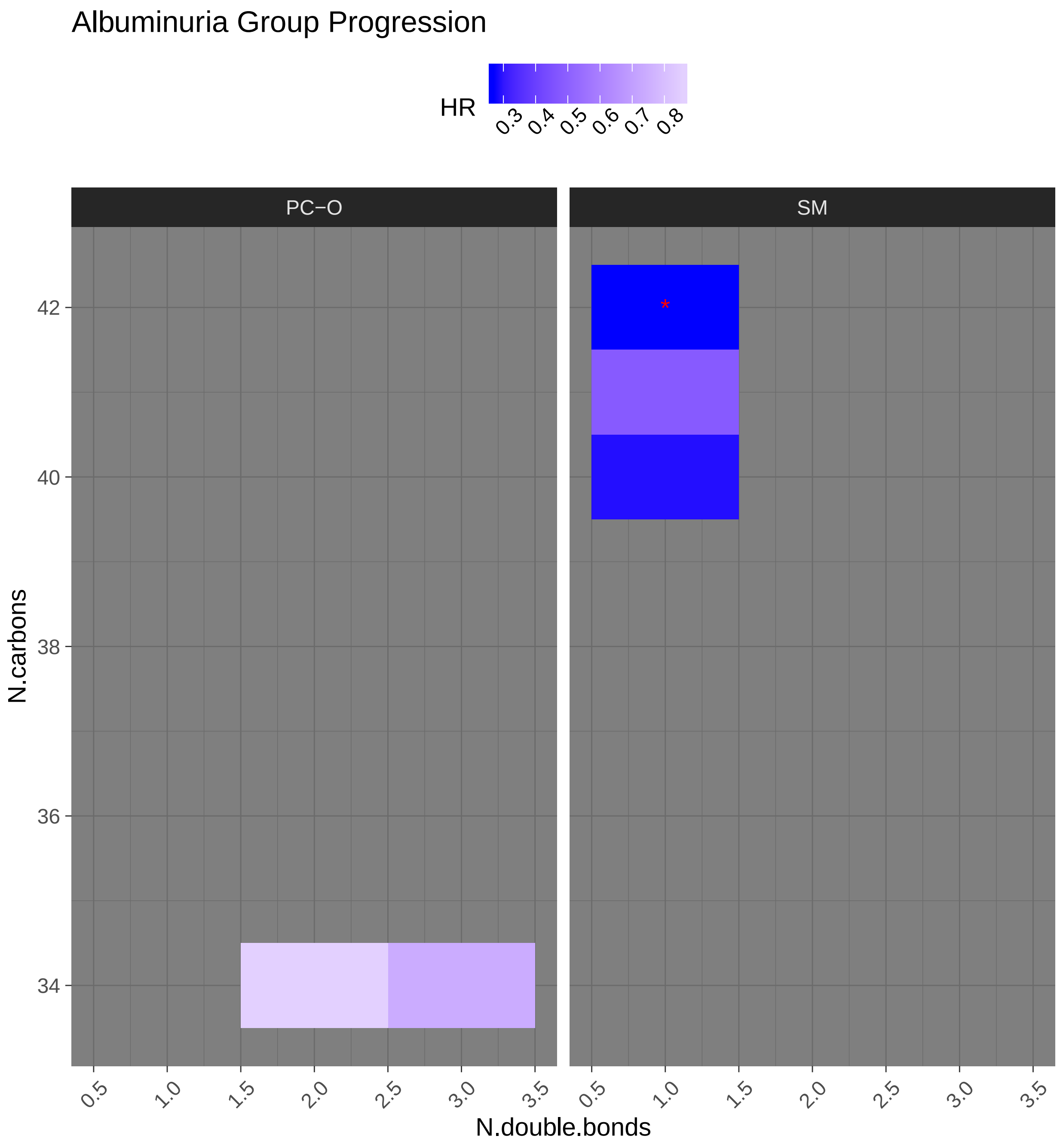

Figure 21: Heatmap of the lipid-specific hazard ratios (HRs) from the adjusted survival model for albuminuria group progression. Lipids are organized into panels (columns) by their class (from PC-O to SM). One colored rectangle corresponds to one lipid species, where the coordinates in the x-axis and y-axis, respectively, show the number of double bonds (N.double.bonds) and the number of carbon atoms (N.carbons) in the fatty acid chains of the lipid. The number of double bonds corresponds to the number of unsaturated bonds in the fatty acid chains, and the number of carbon atoms corresponds to the length of the fatty acid chains and, thus, the size of the lipid. The magnitude of the lipid-specifc hazard ratio is shown as the color of the rectangle (blue: inverse association; red: positive association). Associations with statistical significance are annotated with symbols #, x and *, respectively, corresponding to $p < 0.01$, $p < 0.05$ and $p < 0.1$.

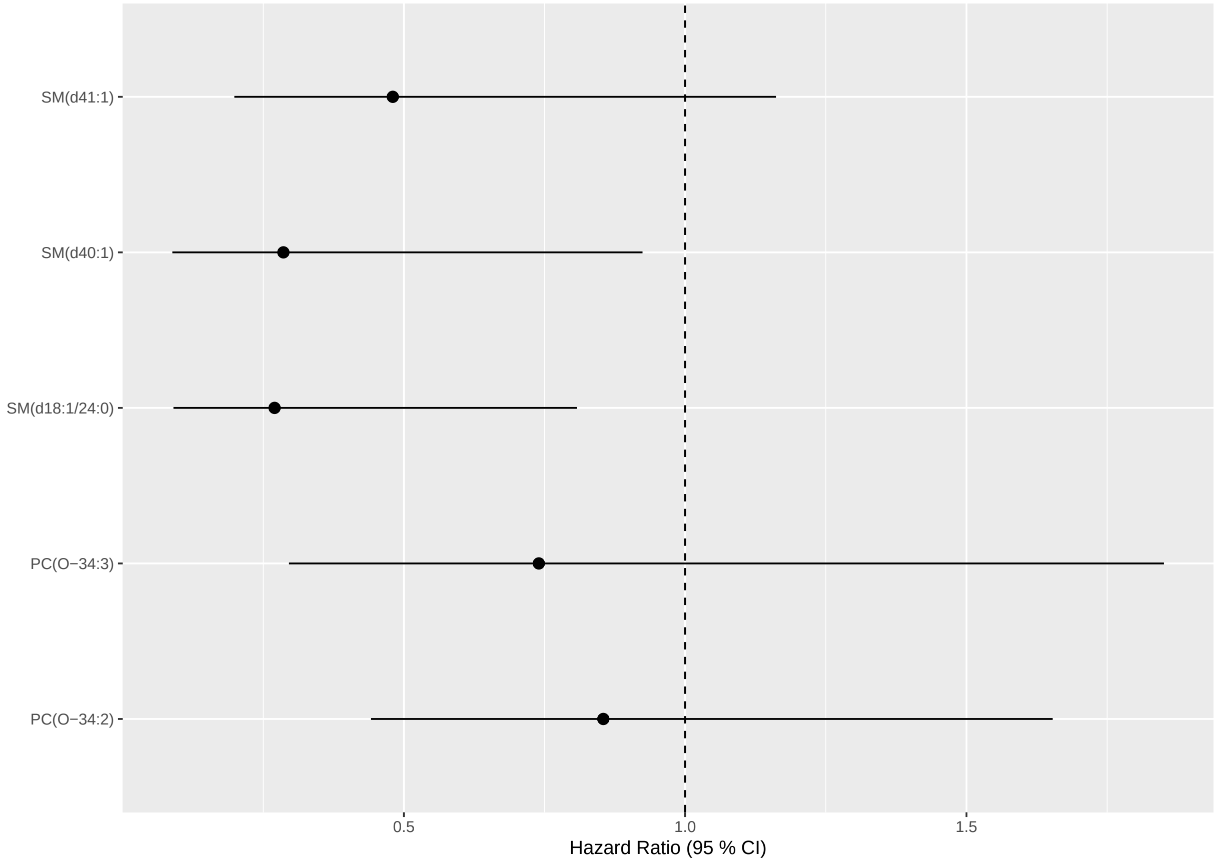

Figure 22: Forest plot of hazard ratios from the adjusted survival model for the albuminuria group progression. The hazard ratio and its 95 % confidence intervals (x-axis) are shown for each lipid (rows).

4.1.2.3 Forest Plot

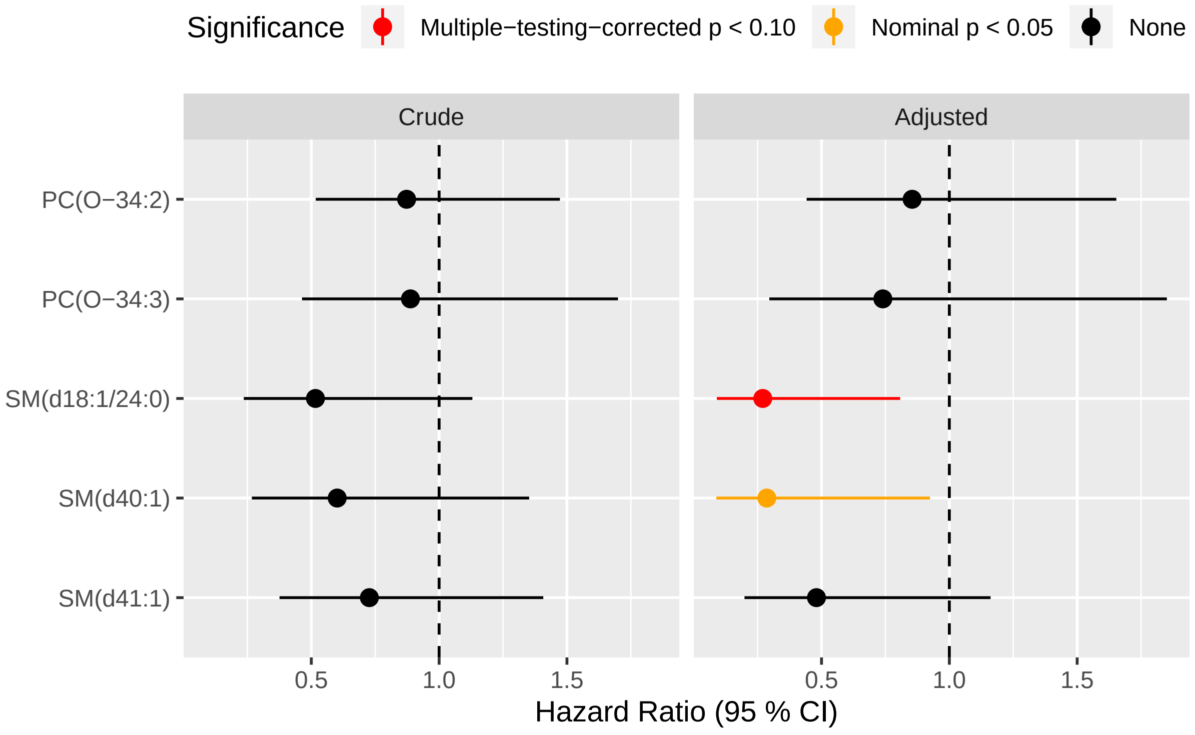

Figure 23: Combined Forest plot of hazard ratios from the crude and adjusted survival models (left and right, respectively) for the albuminuria group progression. The hazard ratio and its 95 % confidence intervals (x-axis) are shown for each lipid (rows).

4.1.3 Combined Forest Plot from Crude and Adjusted Models

4.2 Step 4B: All-Cause Mortality

4.2.1 Crude Model

4.2.1.1 Table

Table 5: Crude survival model for all-cause mortality. Shown in the table are the name of the lipid (Name), the hazard ratio (exp(coef)) and its lower and upper condidence intervals (lower .95 and upper .95, respectively), p-value of the coefficient (Pr(>|z|), and the p-value after correction for multiple testing (p.adj).

Name	exp(coef)	lower .95	upper .95	Pr(>|z|)	p.adj
SM(d41:1)	0.327	0.221	0.483	0.00e+00	1.00e-07
SM(d40:1)	0.232	0.130	0.416	9.00e-07	3.60e-06
PC(O-34:3)	0.364	0.223	0.592	4.67e-05	1.40e-04
SM(d18:1/24:0)	0.318	0.176	0.575	1.49e-04	2.99e-04
PC(O-34:2)	0.475	0.317	0.710	2.91e-04	2.99e-04

4.2.1.2 Heatmaps

```
##   Label Relation p.adj
## 1     #  p.adj <  0.01
## 2     x  p.adj <  0.05
## 3     *  p.adj <  0.10
```

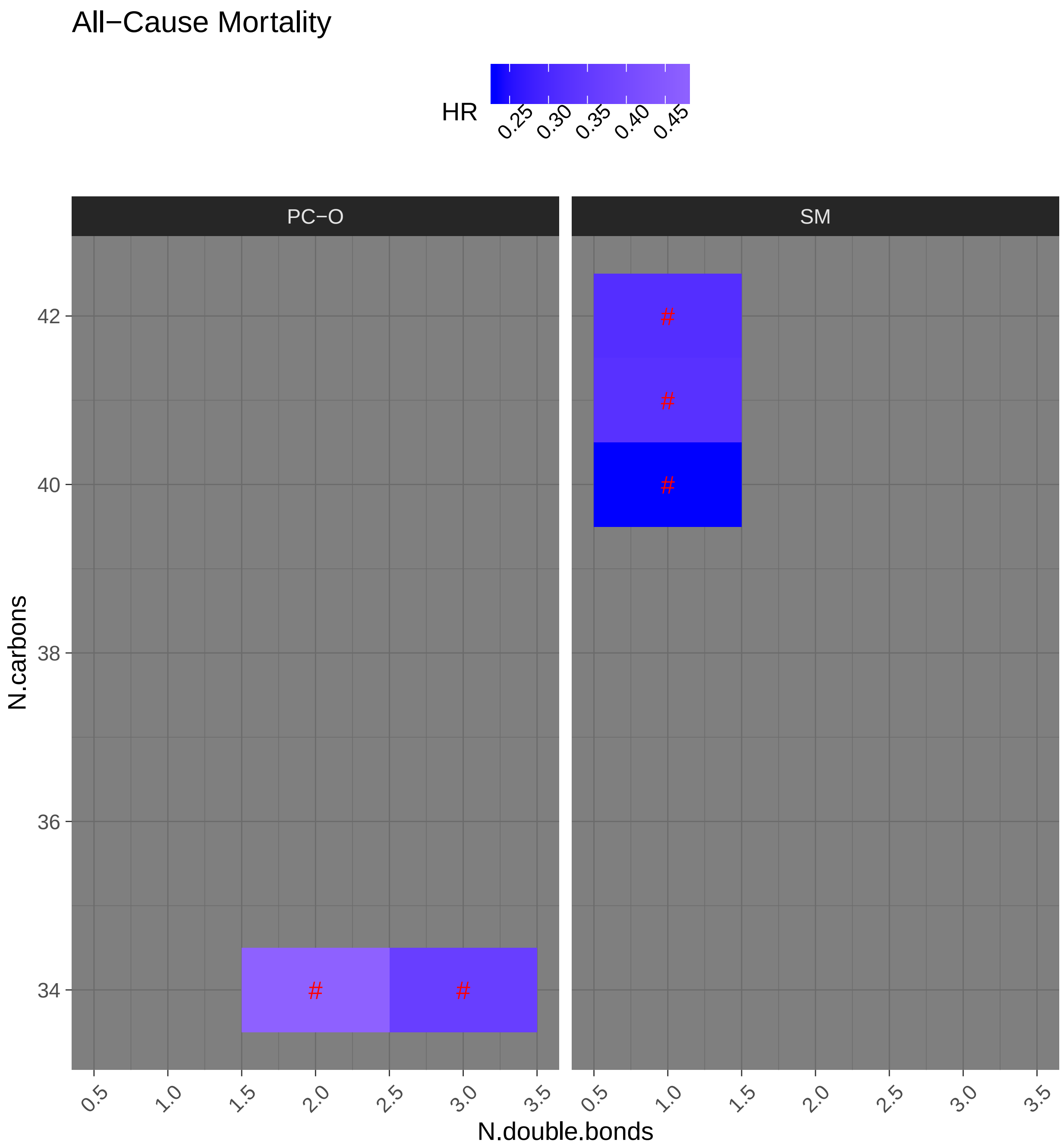

Figure 24: Heatmap of the lipid-specific hazard ratios (HRs) from the crude survival model for all-cause mortality. Lipids are organized into panels (columns) by their class (from PC-O to SM). One colored rectangle corresponds to one lipid species, where the coordinates in the x-axis and y-axis, respectively, show the number of double bonds (N.double.bonds) and the number of carbon atoms (N.carbons) in the fatty acid chains of the lipid. The number of double bonds corresponds to the number of unsaturated bonds in the fatty acid chains, and the number of carbon atoms corresponds to the length of the fatty acid chains and, thus, the size of the lipid. The magnitude of the lipid-specifc hazard ratio is shown as the color of the rectangle (blue: inverse association; red: positive association). Associations with statistical significance are annotated with symbols #, x and *, respectively, corresponding to $p < 0.01$, $p < 0.05$ and $p < 0.1$.

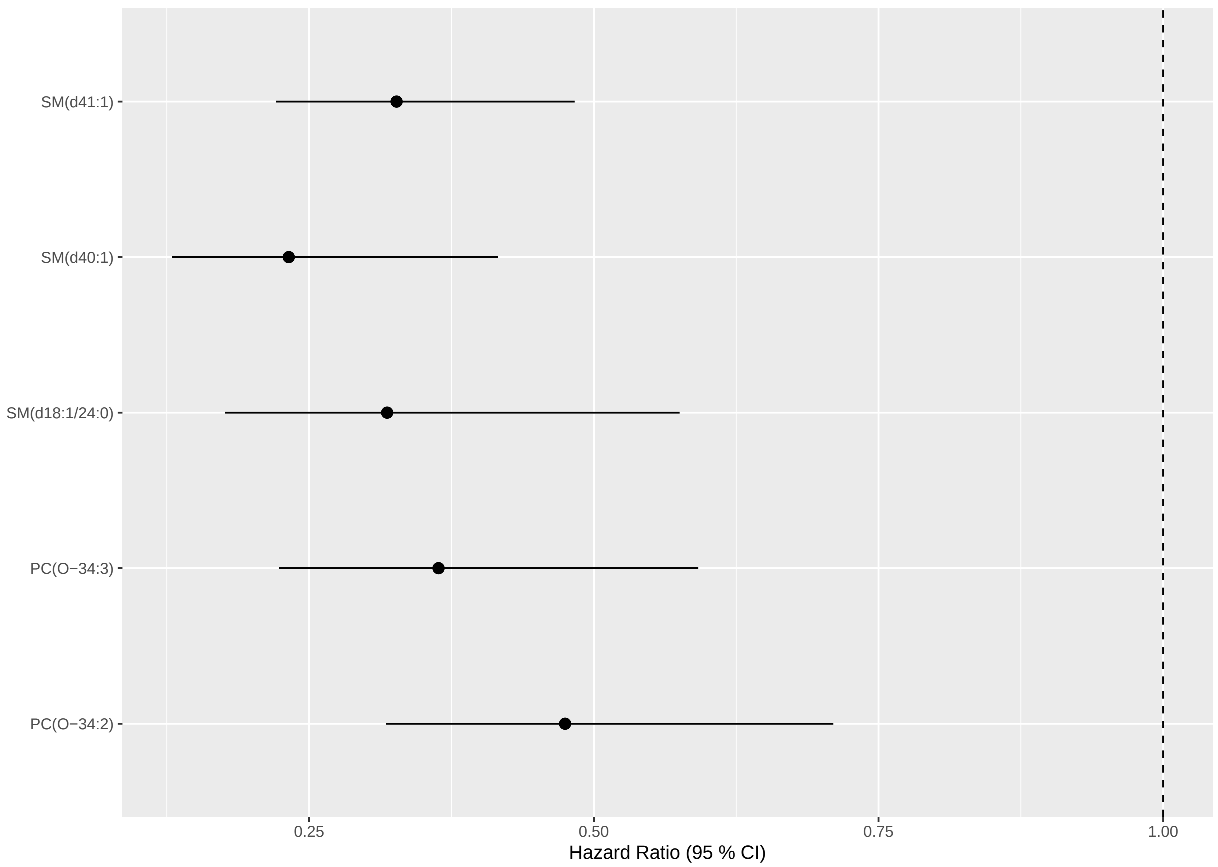

Figure 25: Forest plot of hazard ratios from the crude survival model for all-cause mortality. The hazard ratio and its 95 % confidence intervals (x-axis) are shown for each lipid (rows).

4.2.1.3 Forest Plot

4.2.2 Adjusted Model

4.2.2.1 Table

Table 6: Adjusted survival model for all-cause mortality. Shown in the table are the name of the lipid (Name), the hazard ratio (exp(coef)) and its lower and upper condidence intervals (lower .95 and upper .95, respectively), p-value of the coefficient (Pr(>|z|), and the p-value after correction for multiple testing (p.adj).

Name	exp(coef)	lower .95	upper .95	Pr(>|z|)	p.adj
SM(d41:1)	0.354	0.193	0.649	0.000803	0.00401
SM(d40:1)	0.327	0.143	0.747	0.008050	0.03220
PC(O-34:3)	0.469	0.239	0.920	0.027700	0.08310
PC(O-34:2)	0.623	0.385	1.010	0.053600	0.10700
SM(d18:1/24:0)	0.577	0.257	1.300	0.184000	0.18400

4.2.2.2 Heatmaps

```
##   Label Relation p.adj
## 1     #  p.adj <  0.01
## 2     x  p.adj <  0.05
## 3     *  p.adj <  0.10
```

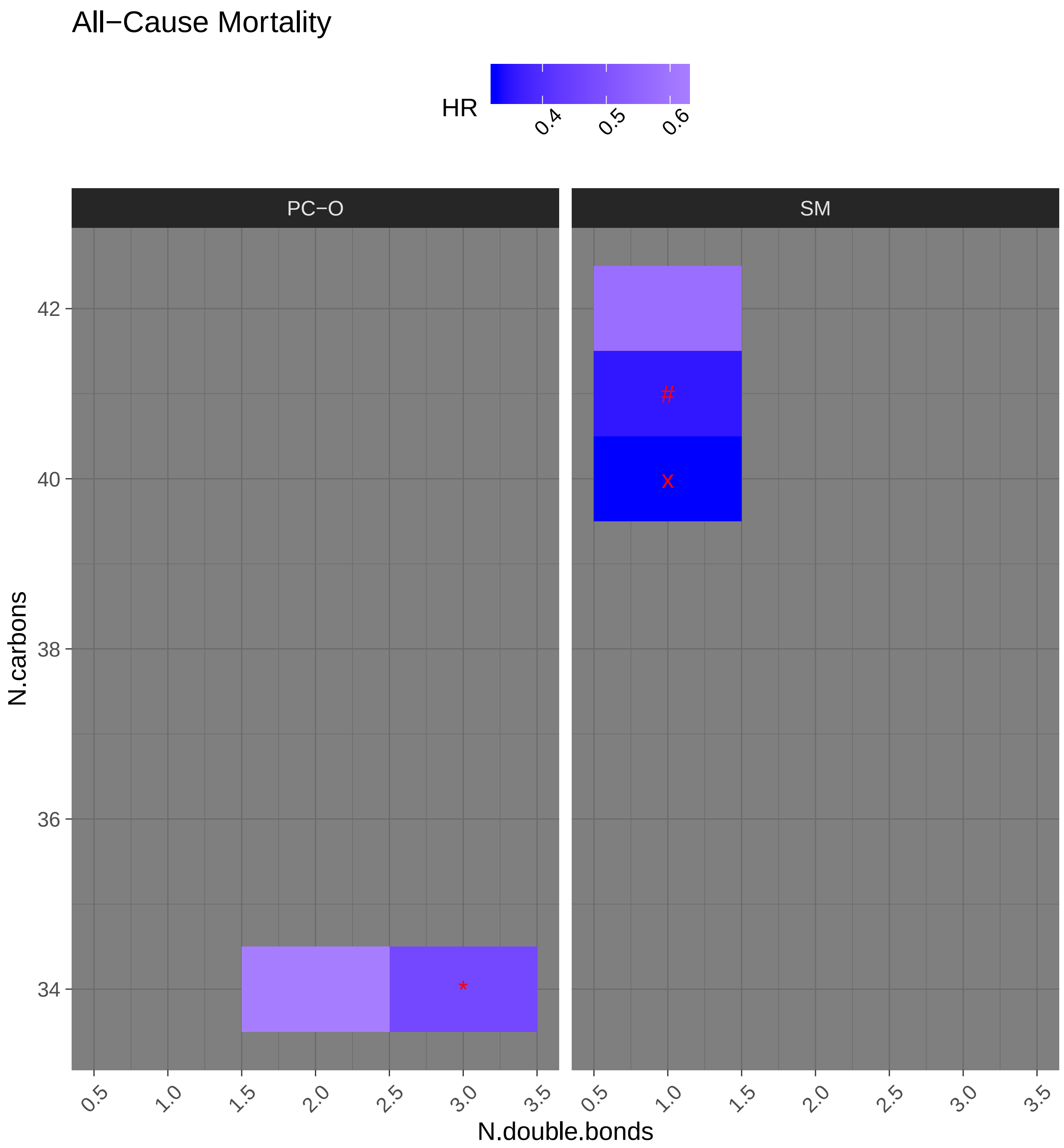

Figure 26: Heatmap of the lipid-specific hazard ratios (HRs) from the adjusted survival model for all-cause mortality. Lipids are organized into panels (columns) by their class (from PC-O to SM). One colored rectangle corresponds to one lipid species, where the coordinates in the x-axis and y-axis, respectively, show the number of double bonds (N.double.bonds) and the number of carbon atoms (N.carbons) in the fatty acid chains of the lipid. The number of double bonds corresponds to the number of unsaturated bonds in the fatty acid chains, and the number of carbon atoms corresponds to the length of the fatty acid chains and, thus, the size of the lipid. The magnitude of the lipid-specifc hazard ratio is shown as the color of the rectangle (blue: inverse association; red: positive association). Associations with statistical significance are annotated with symbols #, x and *, respectively, corresponding to $p < 0.01$, $p < 0.05$ and $p < 0.1$.

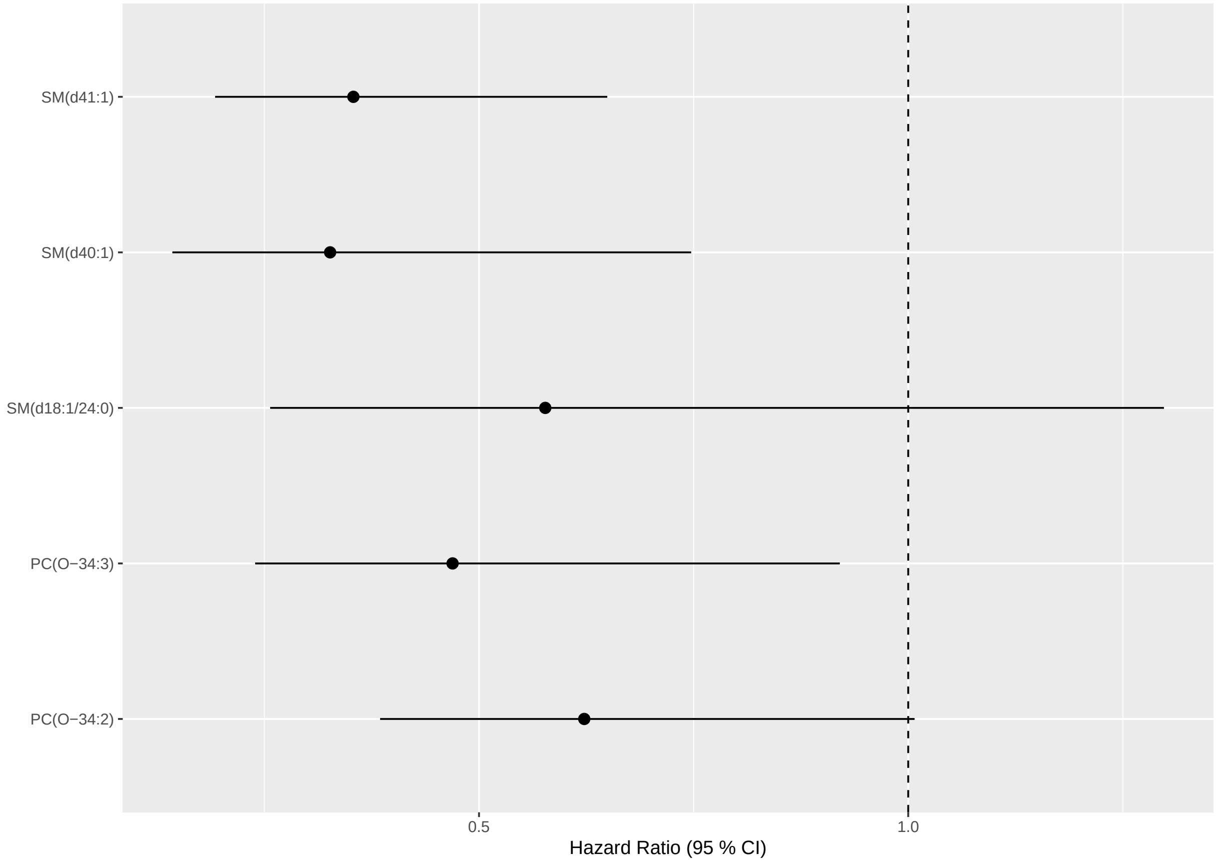

Figure 27: Forest plot of hazard ratios from the adjusted survival model for all-cause mortality. The hazard ratio and its 95 % confidence intervals (x-axis) are shown for each lipid (rows).

4.2.2.3 Forest Plot

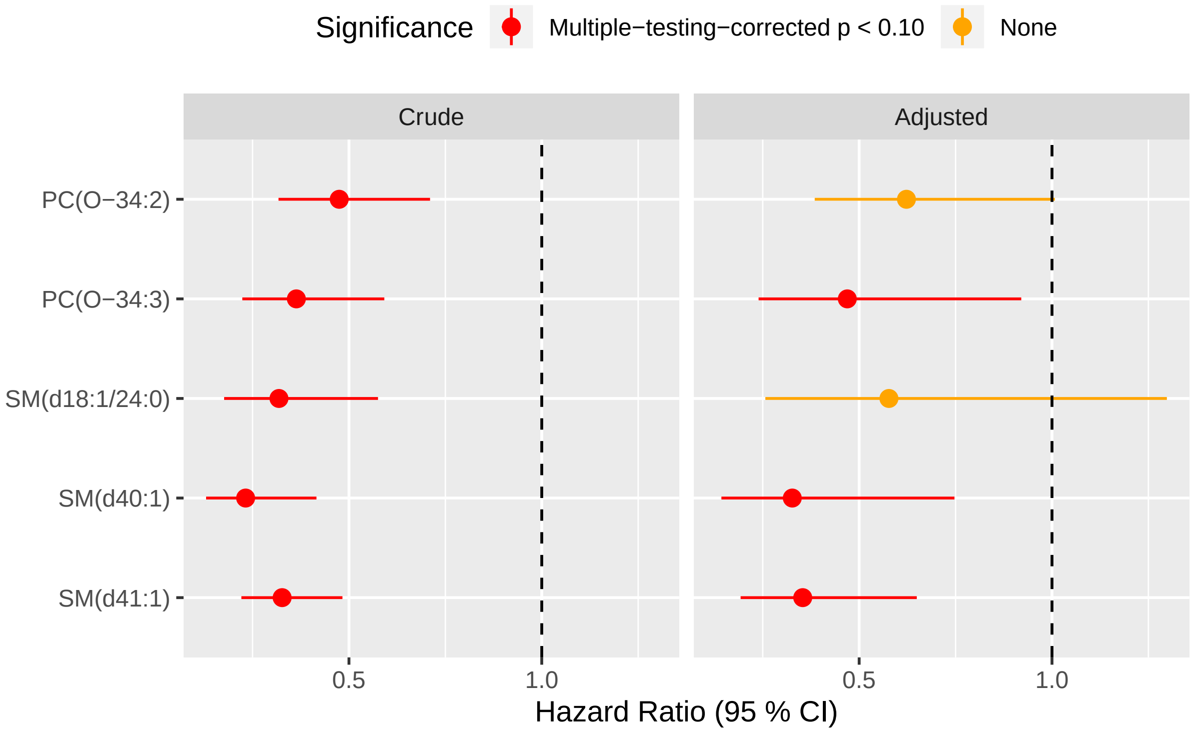

Figure 28: Combined forest plot of hazard ratios from the crude and adjusted survival models (left and right, respectively) for all-cause mortality. The hazard ratio and its 95 % confidence intervals (x-axis) are shown for each lipid (rows).

4.2.3 Combined Forest Plot

4.3 Step 4C: eGFR Decline > 30 %

4.3.1 Crude Model

4.3.1.1 Table

Table 7: Crude survival model for eGFR recline > 30 %. Shown in the table are the name of the lipid (Name), the hazard ratio (exp(coef)) and its lower and upper condidence intervals (lower .95 and upper .95, respectively), p-value of the coefficient (Pr(>|z|), and the p-value after correction for multiple testing (p.adj).

Name	exp(coef)	lower .95	upper .95	Pr(>|z|)	p.adj
PC(O-34:3)	0.544	0.363	0.817	0.00332	0.0166
PC(O-34:2)	0.722	0.521	1.000	0.04980	0.1990
SM(d40:1)	0.773	0.457	1.310	0.33800	1.0000
SM(d18:1/24:0)	0.794	0.482	1.310	0.36600	1.0000
SM(d41:1)	0.839	0.541	1.300	0.43300	1.0000

4.3.1.2 Heatmaps

```
##   Label Relation p.adj
## 1     #  p.adj <  0.01
## 2     x  p.adj <  0.05
## 3     *  p.adj <  0.10
```

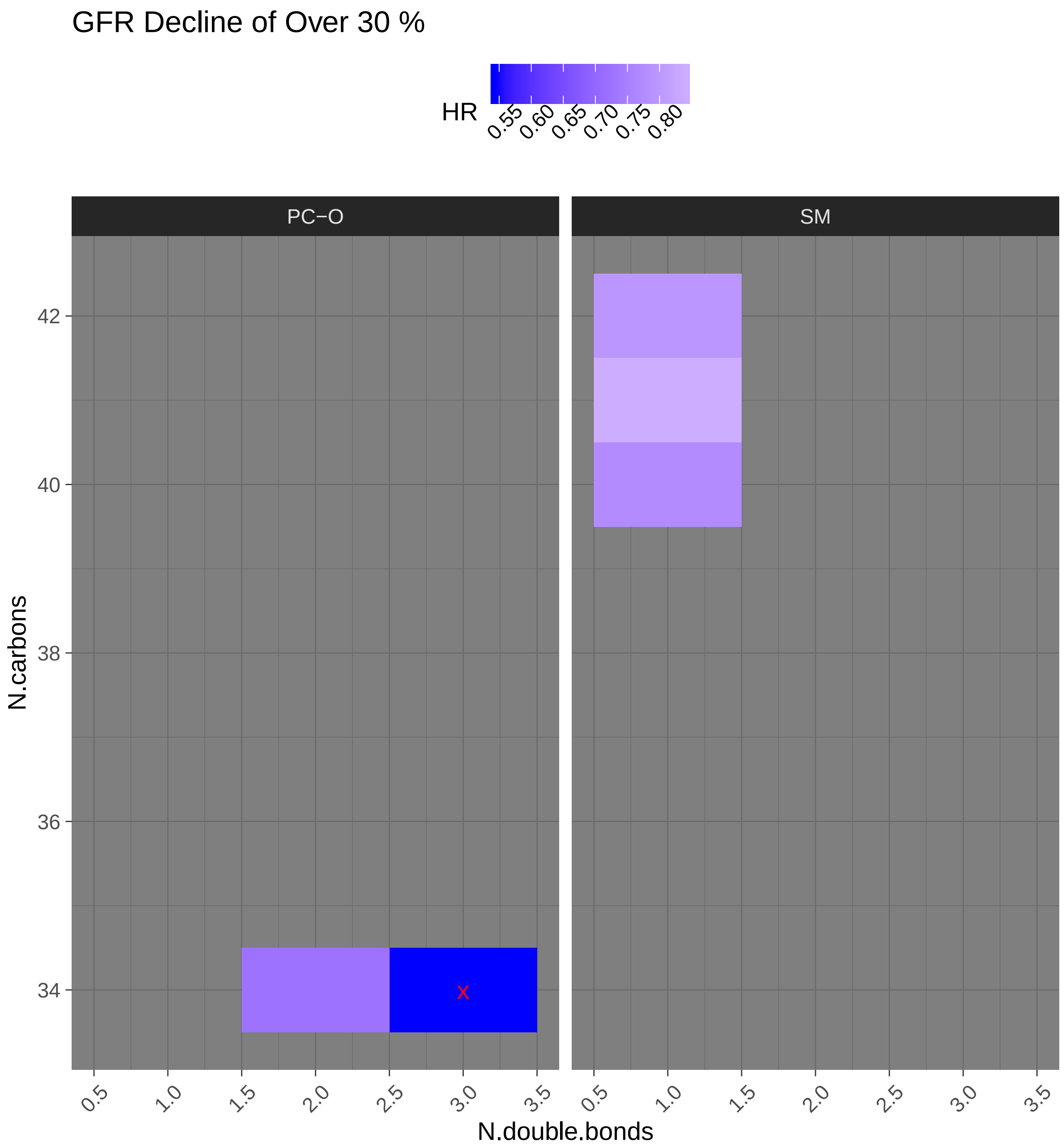

Figure 29: Heatmap of the lipid-specific hazard ratios (HRs) from the crude survival model for eGFR decline (> 30 %). Lipids are organized into panels (columns) by their class (from PC-O to SM). One colored rectangle corresponds to one lipid species, where the coordinates in the x-axis and y-axis, respectively, show the number of double bonds (N.double.bonds) and the number of carbon atoms (N.carbons) in the fatty acid chains of the lipid. The number of double bonds corresponds to the number of unsaturated bonds in the fatty acid chains, and the number of carbon atoms corresponds to the length of the fatty acid chains and, thus, the size of the lipid. The magnitude of the lipid-specifc hazard ratio is shown as the color of the rectangle (blue: inverse association; red: positive association). Associations with statistical significance are annotated with symbols #, x and *, respectively, corresponding to $p < 0.01$, $p < 0.05$ and $p < 0.1$.

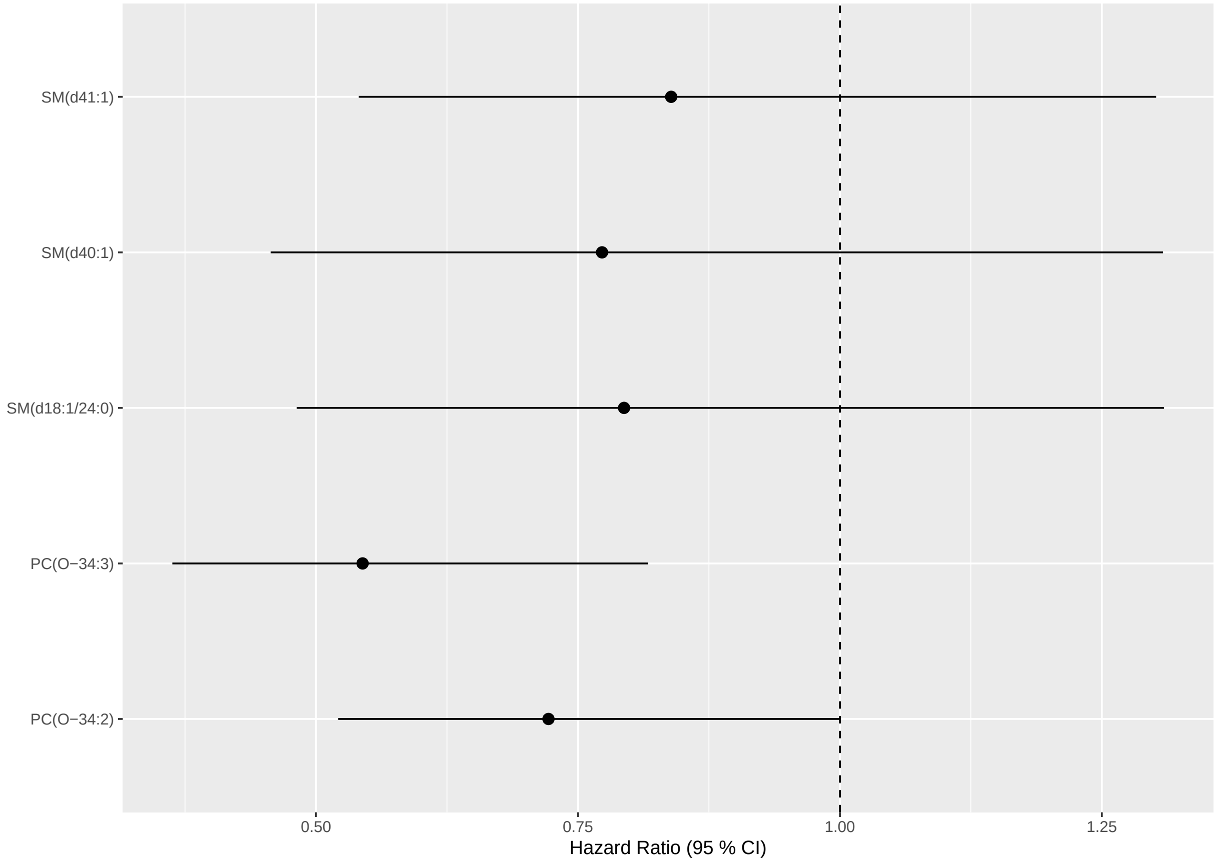

Figure 30: Forest plot of hazard ratios from the crude survival model for eGFR decline (> 30 %). The hazard ratio and its 95 % confidence intervals (x-axis) are shown for each lipid (rows).

4.3.1.3 Forest Plot

4.3.2 Adjusted Model

4.3.2.1 Table

Table 8: Adjusted survival model for eGFR decline > 30 %. Shown in the table are the name of the lipid (Name), the hazard ratio (exp(coef)) and its lower and upper condidence intervals (lower .95 and upper .95, respectively), p-value of the coefficient (Pr(>|z|), and the p-value after correction for multiple testing (p.adj).

Name	exp(coef)	lower .95	upper .95	Pr(>|z|)	p.adj
SM(d40:1)	0.598	0.293	1.22	0.159	0.794
PC(O-34:2)	0.866	0.597	1.26	0.448	1.000
SM(d18:1/24:0)	0.774	0.390	1.54	0.466	1.000
SM(d41:1)	0.819	0.455	1.47	0.506	1.000
PC(O-34:3)	0.909	0.547	1.51	0.714	1.000

4.3.2.2 Heatmaps

```
##   Label Relation p.adj
## 1     #  p.adj <  0.01
## 2     x  p.adj <  0.05
## 3     *  p.adj <  0.10
```

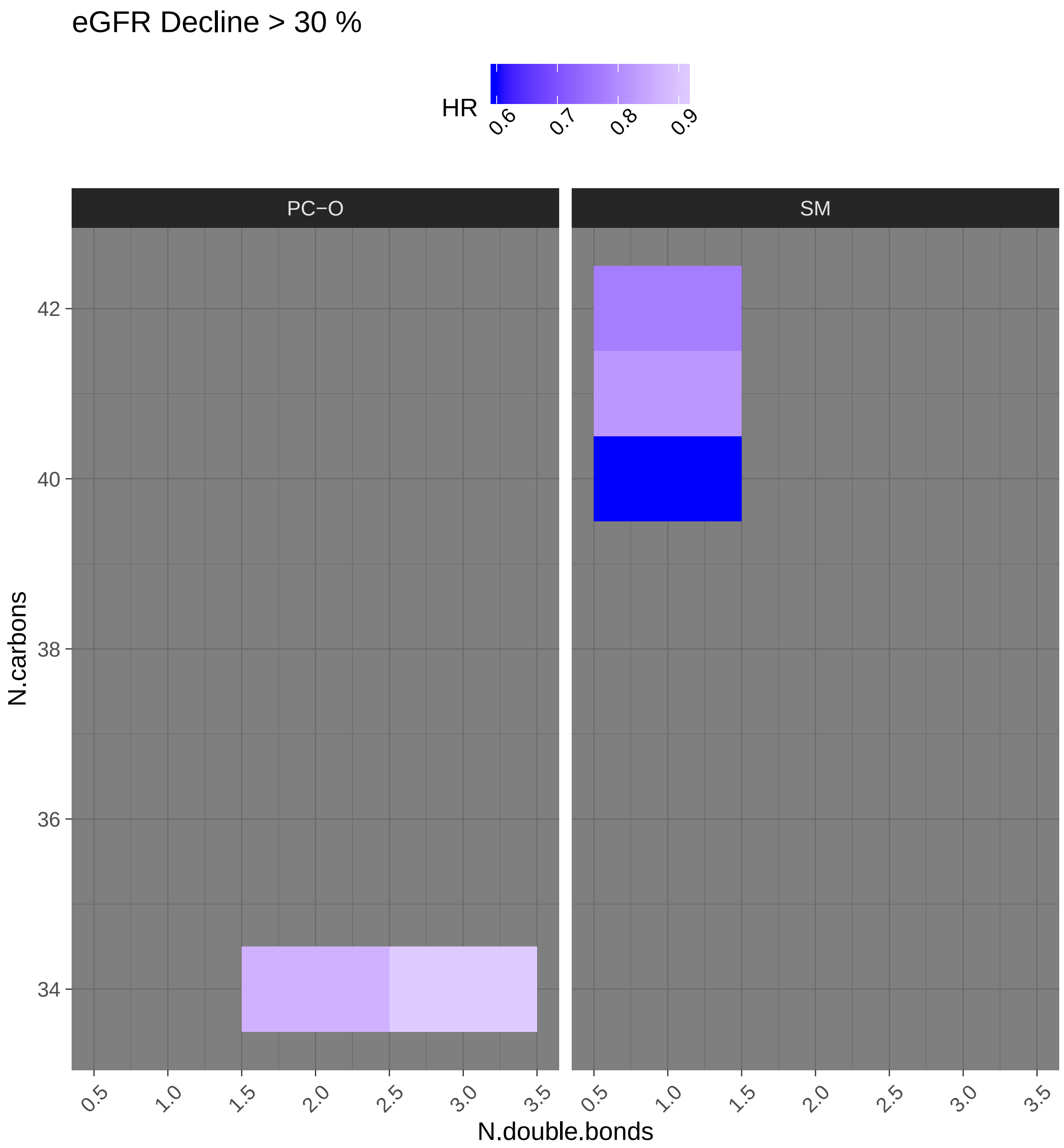

Figure 31: Heatmap of the lipid-specific hazard ratios (HRs) from the adjusted survival model for eGFR decline (> 30 %). Lipids are organized into panels (columns) by their class (from PC-O to SM). One colored rectangle corresponds to one lipid species, where the coordinates in the x-axis and y-axis, respectively, show the number of double bonds (N.double.bonds) and the number of carbon atoms (N.carbons) in the fatty acid chains of the lipid. The number of double bonds corresponds to the number of unsaturated bonds in the fatty acid chains, and the number of carbon atoms corresponds to the length of the fatty acid chains and, thus, the size of the lipid. The magnitude of the lipid-specifc hazard ratio is shown as the color of the rectangle (blue: inverse association; red: positive association). Associations with statistical significance are annotated with symbols #, x and *, respectively, corresponding to $p < 0.01$, $p < 0.05$ and $p < 0.1$.

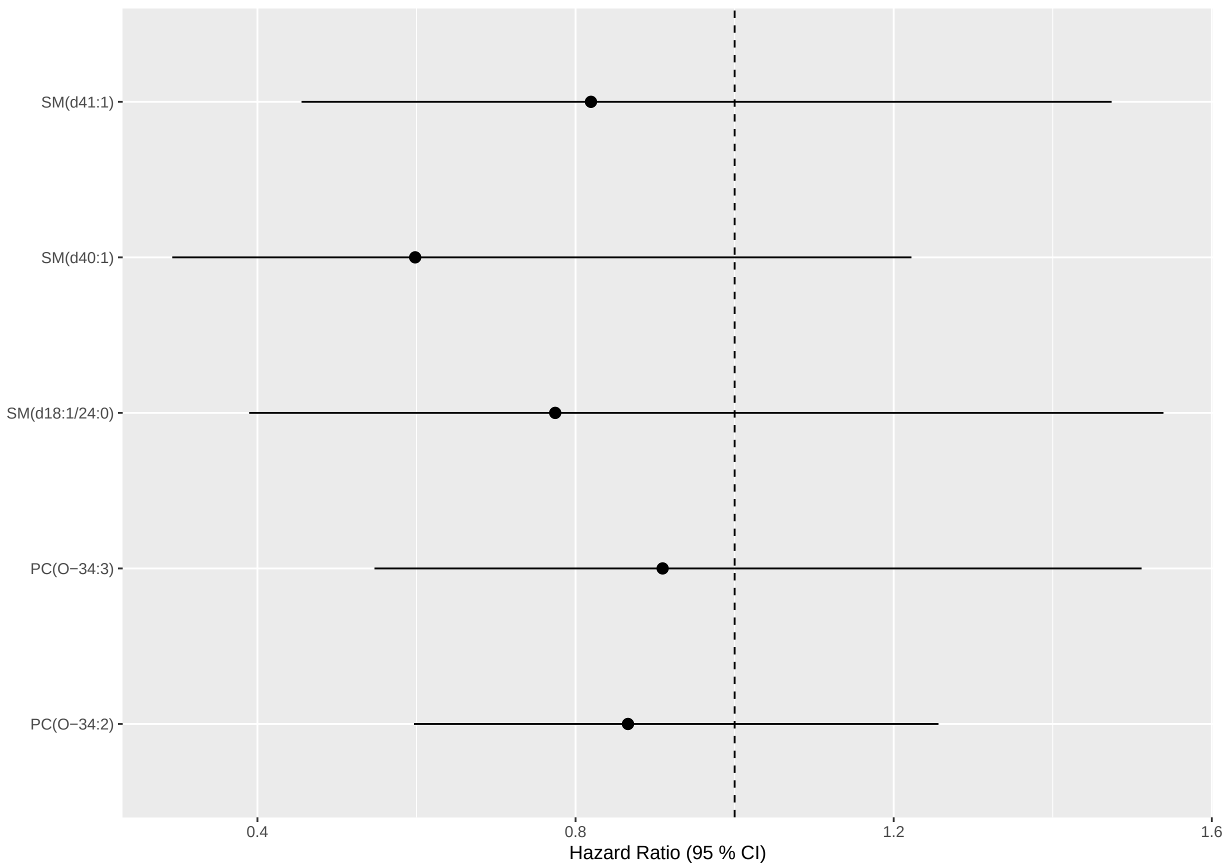

Figure 32: Forest plot of hazard ratios from the adjusted survival model for eGFR decline (> 30 %). The hazard ratio and its 95 % confidence intervals (x-axis) are shown for each lipid (rows).

4.3.2.3 Forest Plot

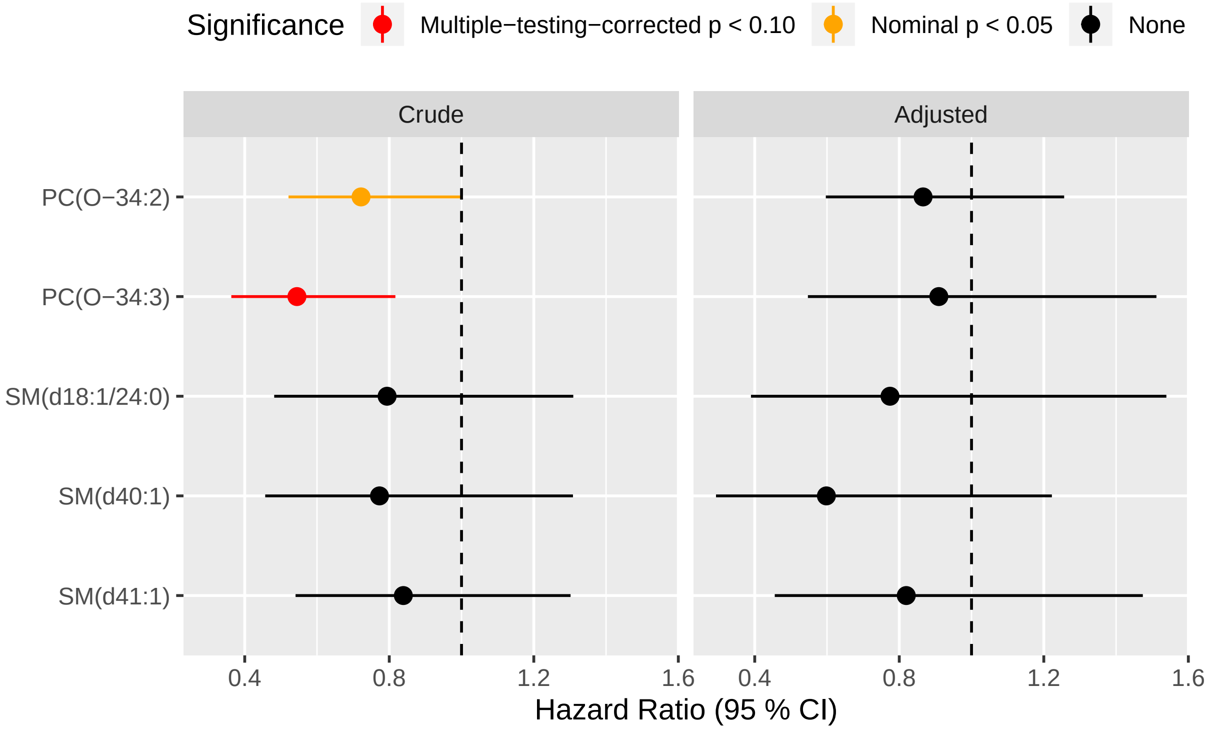

Figure 33: Combined forest plot of hazard ratios from the crude and adjusted survival models (left and right, respectively) for eGFR decline (>30 %). The hazard ratio and its 95 % confidence intervals (x-axis) are shown for each lipid (rows).

4.3.3 Combined Forest Plot from Crude and Adjusted Models

4.4 Step 4D: End-Stage Renal Disease

4.4.1 Crude Model

4.4.1.1 Table

Table 9: Crude survival model for end-stage renal disease. Shown in the table are the name of the lipid (Name), the hazard ratio (exp(coef)) and its lower and upper condidence intervals (lower .95 and upper .95, respectively), p-value of the coefficient (Pr(>|z|), and the p-value after correction for multiple testing (p.adj).

Name	exp(coef)	lower .95	upper .95	Pr(>|z|)	p.adj
PC(O-34:3)	0.192	0.0908	0.404	1.43e-05	7.15e-05
SM(d18:1/24:0)	0.203	0.0810	0.509	6.76e-04	2.70e-03
SM(d40:1)	0.279	0.1090	0.717	8.03e-03	2.41e-02
SM(d41:1)	0.404	0.2050	0.796	8.87e-03	2.41e-02
PC(O-34:2)	0.543	0.2850	1.040	6.36e-02	6.36e-02

4.4.1.2 Heatmaps

```
##   Label Relation p.adj
## 1     #  p.adj <  0.01
## 2     x  p.adj <  0.05
## 3     *  p.adj <  0.10
```

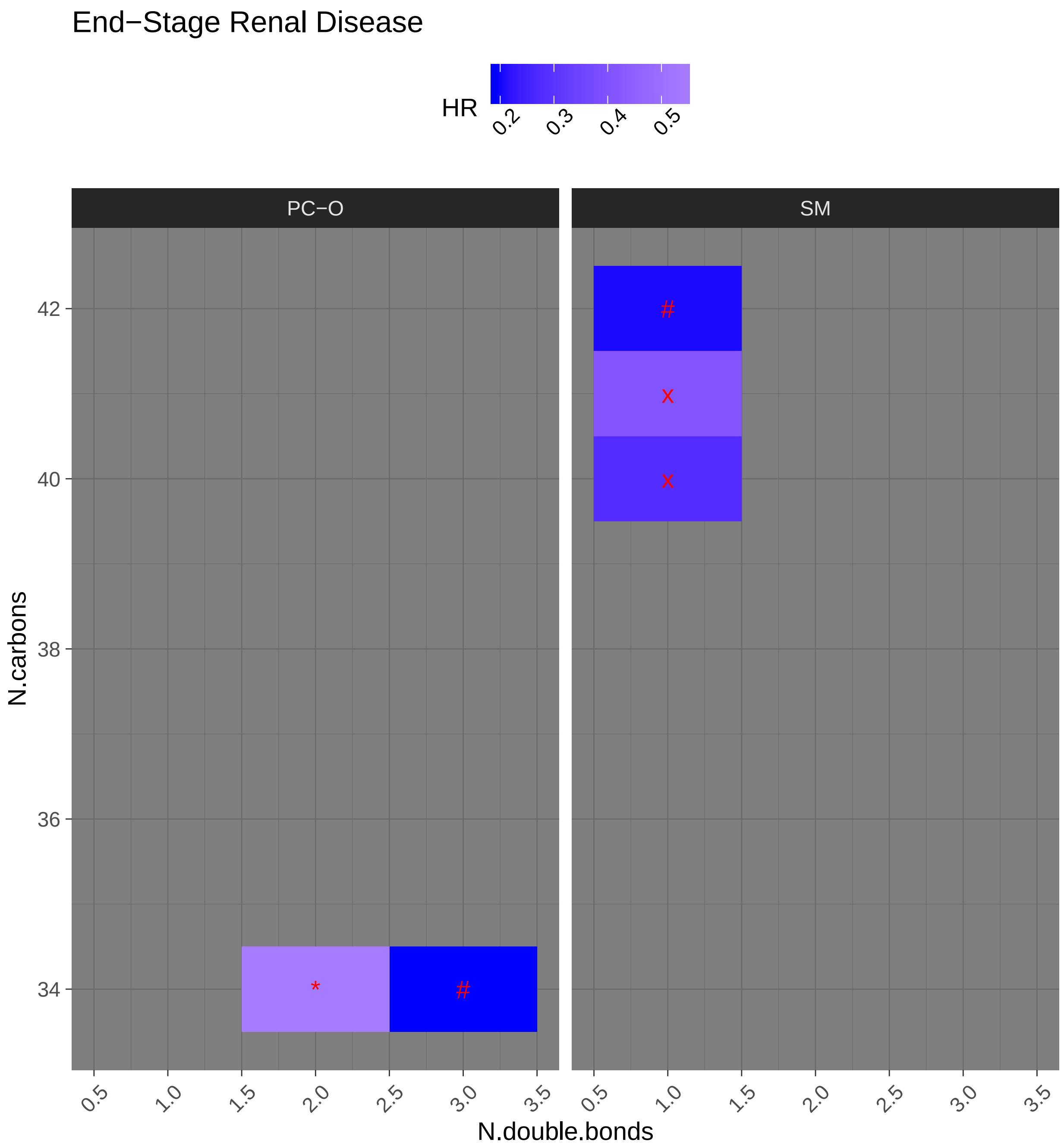

Figure 34: Heatmap of the lipid-specific hazard ratios (HRs) from the crude survival model for end-stage renal disease. Lipids are organized into panels (columns) by their class (from PC-O to SM). One colored rectangle corresponds to one lipid species, where the coordinates in the x-axis and y-axis, respectively, show the number of double bonds (N.double.bonds) and the number of carbon atoms (N.carbons) in the fatty acid chains of the lipid. The number of double bonds corresponds to the number of unsaturated bonds in the fatty acid chains, and the number of carbon atoms corresponds to the length of the fatty acid chains and, thus, the size of the lipid. The magnitude of the lipid-specifc hazard ratio is shown as the color of the rectangle (blue: inverse association; red: positive association). Associations with statistical significance are annotated with symbols #, x and *, respectively, corresponding to $p < 0.01$, $p < 0.05$ and $p < 0.1$.

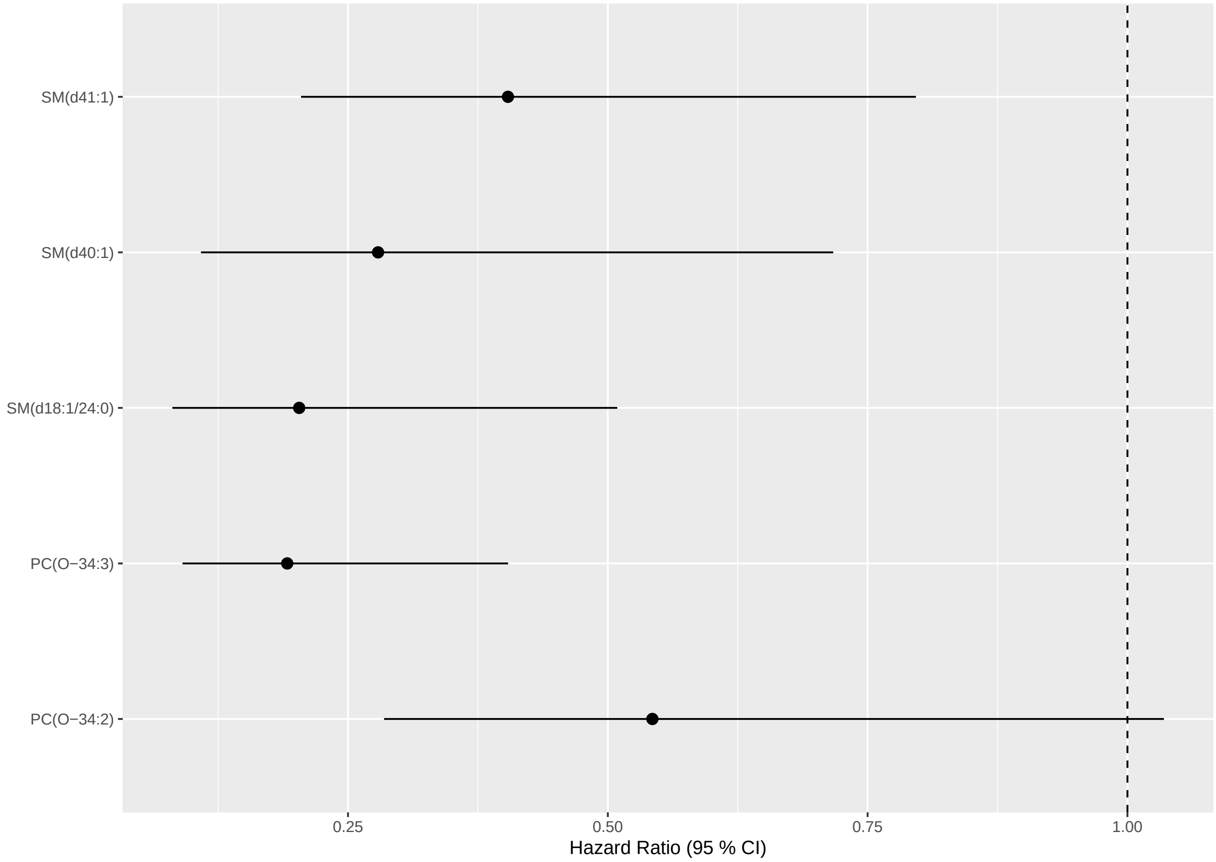

Figure 35: Forest plot of hazard ratios from the crude survival model for end-stage renal disease. The hazard ratio and its 95 % confidence intervals (x-axis) are shown for each lipid (rows).

4.4.1.3 Forest Plot

4.4.2 Adjusted Model

4.4.2.1 Table

Table 10: Adjusted survival model for end stage renal disease. Shown in the table are the name of the lipid (Name), the hazard ratio (exp(coef)) and its lower and upper condidence intervals (lower .95 and upper .95, respectively), p-value of the coefficient (Pr(>|z|), and the p-value after correction for multiple testing (p.adj).

Name	exp(coef)	lower .95	upper .95	Pr(>|z|)	p.adj
SM(d41:1)	0.531	0.147	1.92	0.335	1
SM(d40:1)	0.524	0.107	2.57	0.426	1
SM(d18:1/24:0)	0.687	0.178	2.66	0.586	1
PC(O-34:3)	0.785	0.251	2.45	0.676	1
PC(O-34:2)	0.917	0.389	2.16	0.842	1

4.4.2.2 Heatmaps

```
##   Label Relation p.adj
## 1     #  p.adj <  0.01
## 2     x  p.adj <  0.05
## 3     *  p.adj <  0.10
```

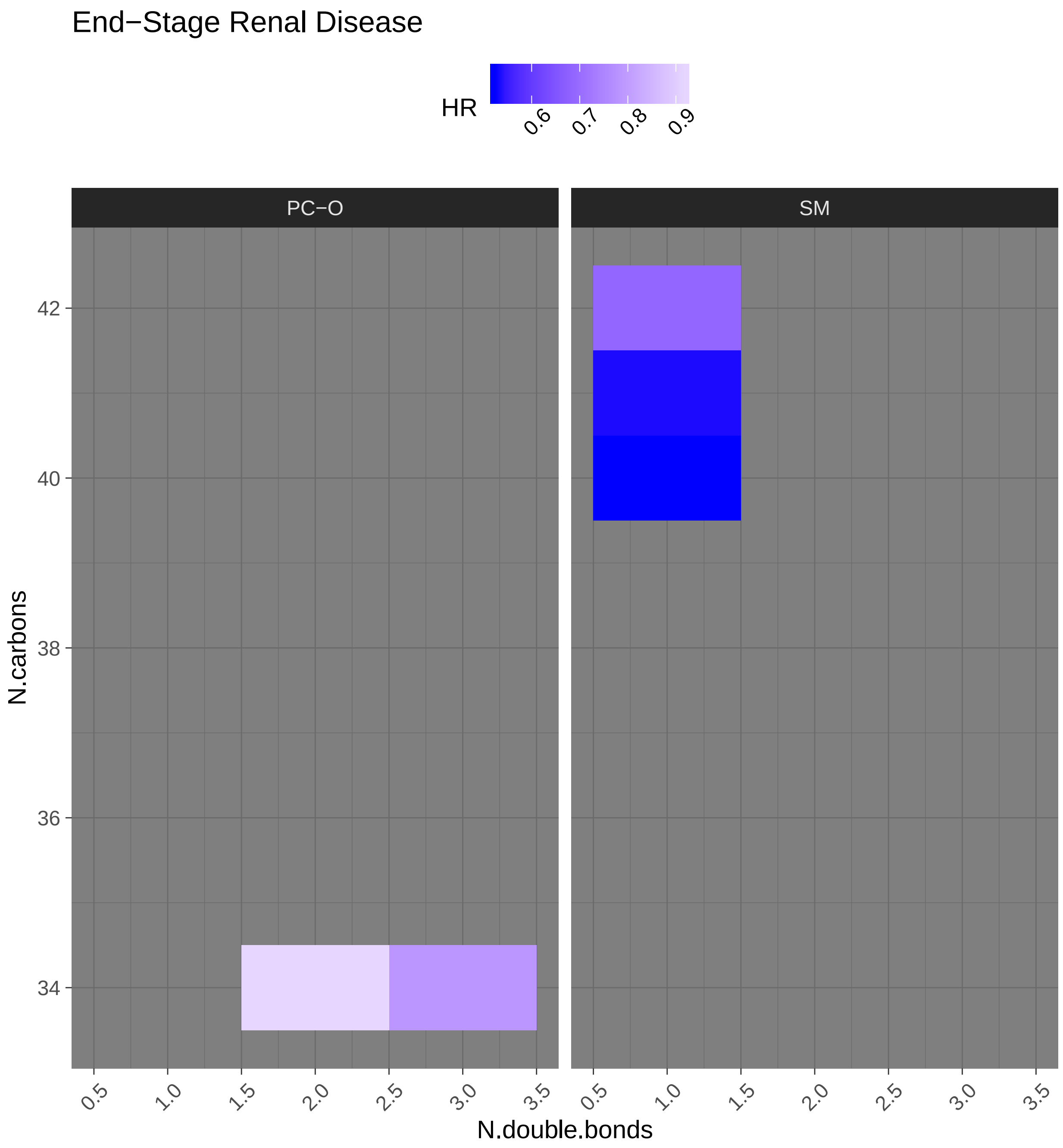

Figure 36: Heatmap of the lipid-specific hazard ratios (HRs) from the adjusted survival model for end-stage renal disease. Lipids are organized into panels (columns) by their class (from PC-O to SM). One colored rectangle corresponds to one lipid species, where the coordinates in the x-axis and y-axis, respectively, show the number of double bonds (N.double.bonds) and the number of carbon atoms (N.carbons) in the fatty acid chains of the lipid. The number of double bonds corresponds to the number of unsaturated bonds in the fatty acid chains, and the number of carbon atoms corresponds to the length of the fatty acid chains and, thus, the size of the lipid. The magnitude of the lipid-specifc hazard ratio is shown as the color of the rectangle (blue: inverse association; red: positive association). Associations with statistical significance are annotated with symbols #, x and *, respectively, corresponding to $p < 0.01$, $p < 0.05$ and $p < 0.1$.

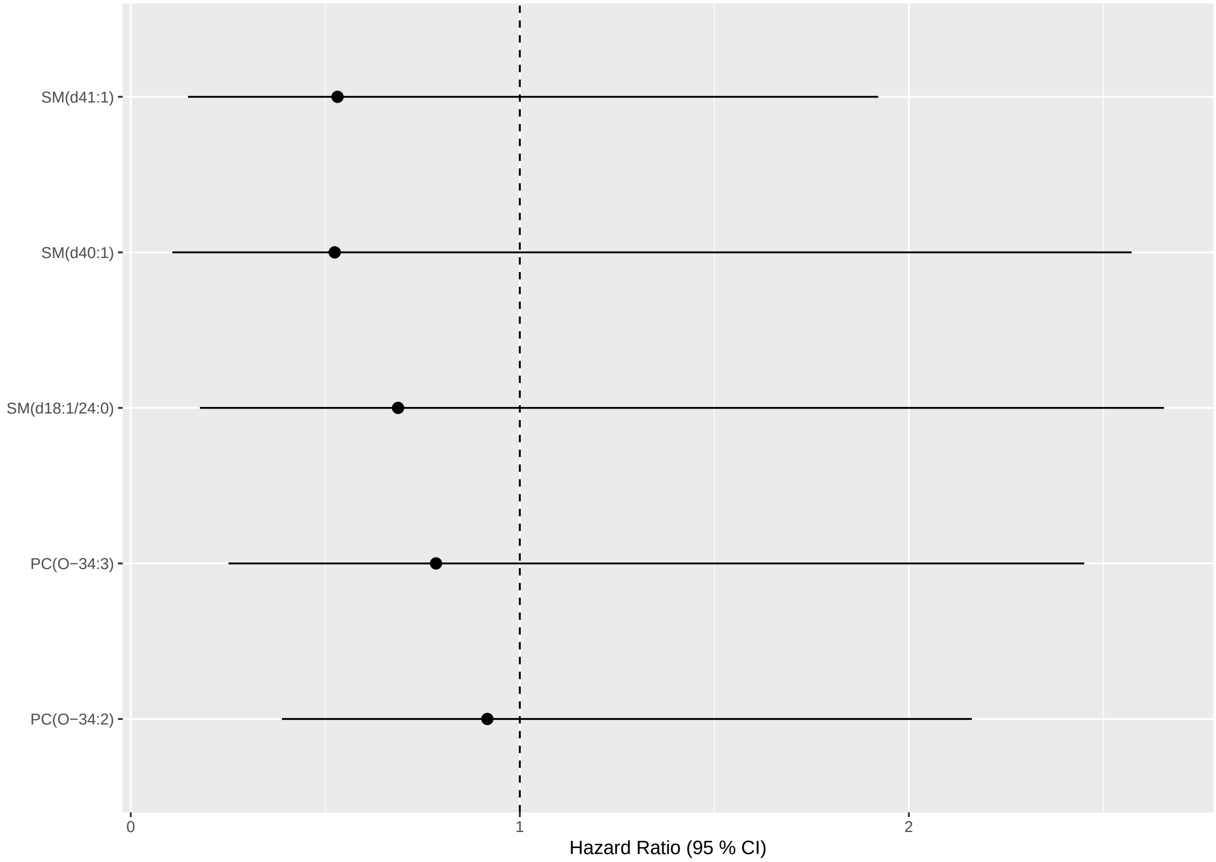

Figure 37: Forest plot of hazard ratios from the adjusted survival model for end-stage renal disease. The hazard ratio and its 95 % confidence intervals (x-axis) are shown for each lipid (rows).

4.4.2.3 Forest Plot

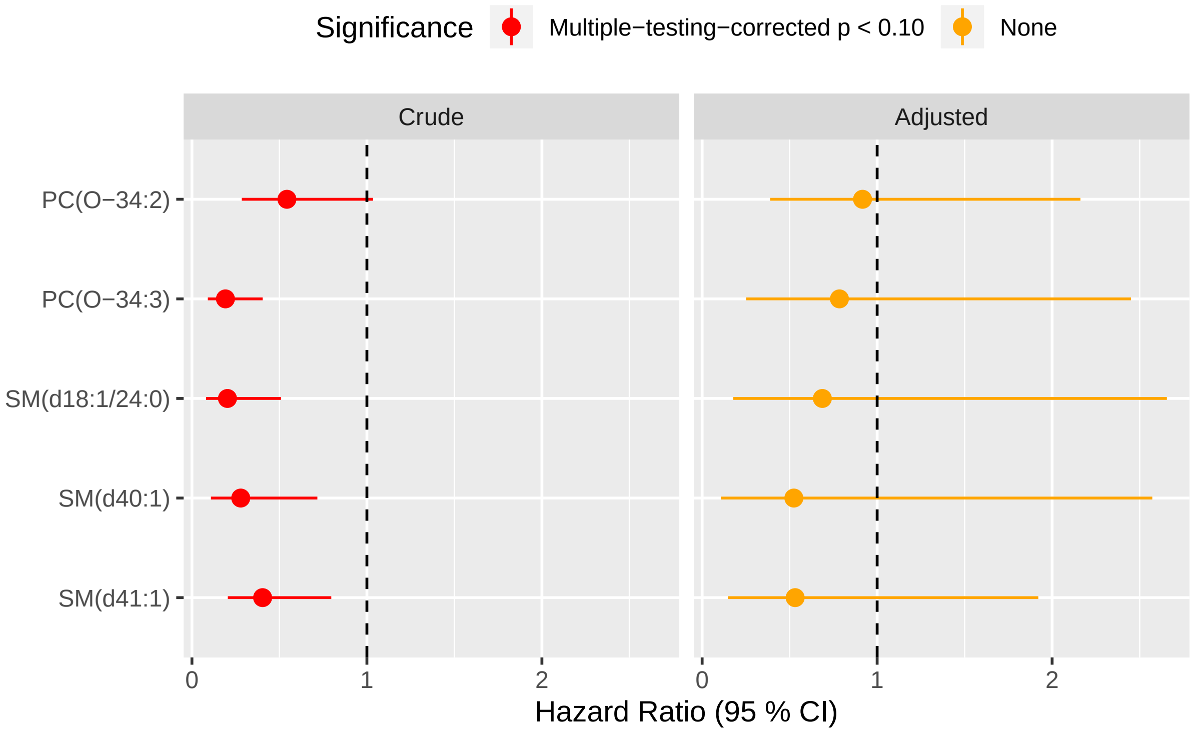

Figure 38: Combined forest plot of hazard ratios from the crude and adjusted survival models for end-stage renal disease. The hazard ratio and its 95 % confidence intervals (x-axis) are shown for each lipid (rows).

4.4.3 Combined Forest Plot from Crude and Adjusted Models

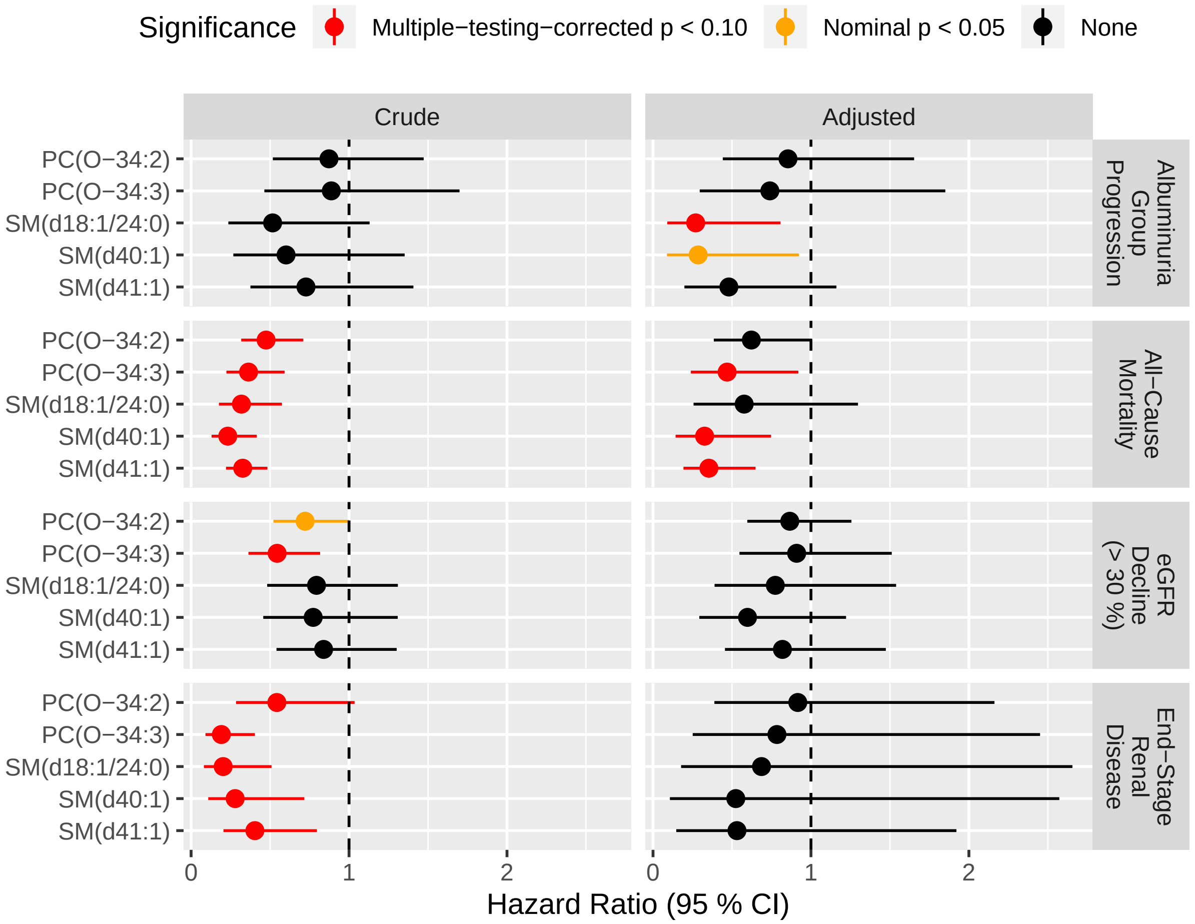

Figure 39: Compilation forest plot of hazard ratios from the crude and adjusted (left and right, respectively) survival models for the four endpoints (row-wise panels). The endpoints are albuminuria group progression, all-cause mortality, eGFR decline (> 30 %) and end-stage renal disease. The hazard ratio and its 95 % confidence intervals (x-axis) are shown for each lipid (rows).

4.5 Compilation Forest Plot from Steps 4A-D

5 Step 5: Detailed Assessment of the Top-Lipid in Relation to All-Cause Mortality

5.1 Step 5A: Analysis of Full Cohort

5.1.1 Survival Model with Details

```
## Call:
## survival::coxph(formula = survival::Surv(time = t_doed_profil,
##     event = censor_doed_profil.reversed.numeric) ~ SM.d41.1 +
##     Age + BMI + BP_Systolic + Cholesterol + eGFR + HbA1c + Medication_Statins +
##     Sex + Smoking + TG_total_log + UAER_log, data = data.survival)
##
##   n= 615, number of events= 54
##    (54 observations deleted due to missingness)
##
##                         coef exp(coef)  se(coef)      z Pr(>|z|)
## SM.d41.1           -1.039477  0.353639  0.310116 -3.352 0.000803 ***
## Age                 0.084875  1.088581  0.015360  5.526 3.28e-08 ***
## BMI                -0.075662  0.927129  0.040516 -1.867 0.061835 .
## BP_Systolic         0.001151  1.001152  0.008047  0.143 0.886259
## Cholesterol        -0.030785  0.969684  0.220076 -0.140 0.888753
## eGFR               -0.011654  0.988413  0.005703 -2.043 0.041009 *
## HbA1c               0.204795  1.227274  0.136686  1.498 0.134057
## Medication_Statins  0.229558  1.258044  0.356499  0.644 0.519624
## Sex                -0.062330  0.939573  0.322626 -0.193 0.846805
## Smoking             0.457539  1.580180  0.321395  1.424 0.154561
## TG_total_log        0.128373  1.136977  0.234372  0.548 0.583876
## UAER_log            0.116122  1.123133  0.069343  1.675 0.094013 .
## ---
## Signif. codes:  0 '***' 0.001 '**' 0.01 '*' 0.05 '.' 0.1 ' ' 1
##
##                    exp(coef) exp(-coef) lower .95 upper .95
## SM.d41.1              0.3536     2.8277    0.1926    0.6494
## Age                   1.0886     0.9186    1.0563    1.1219
## BMI                   0.9271     1.0786    0.8564    1.0038
## BP_Systolic           1.0012     0.9988    0.9855    1.0171
## Cholesterol           0.9697     1.0313    0.6299    1.4927
## eGFR                  0.9884     1.0117    0.9774    0.9995
## HbA1c                 1.2273     0.8148    0.9388    1.6043
## Medication_Statins    1.2580     0.7949    0.6255    2.5302
## Sex                   0.9396     1.0643    0.4992    1.7683
## Smoking               1.5802     0.6328    0.8417    2.9667
## TG_total_log          1.1370     0.8795    0.7182    1.7999
## UAER_log              1.1231     0.8904    0.9804    1.2866
##
## Concordance= 0.824  (se = 0.04 )
## Rsquare= 0.123   (max possible= 0.669 )
## Likelihood ratio test= 80.61  on 12 df,   p=3.16e-12
## Wald test            = 65.62  on 12 df,   p=2.092e-09
## Score (logrank) test = 74.8  on 12 df,   p=4.002e-11
```

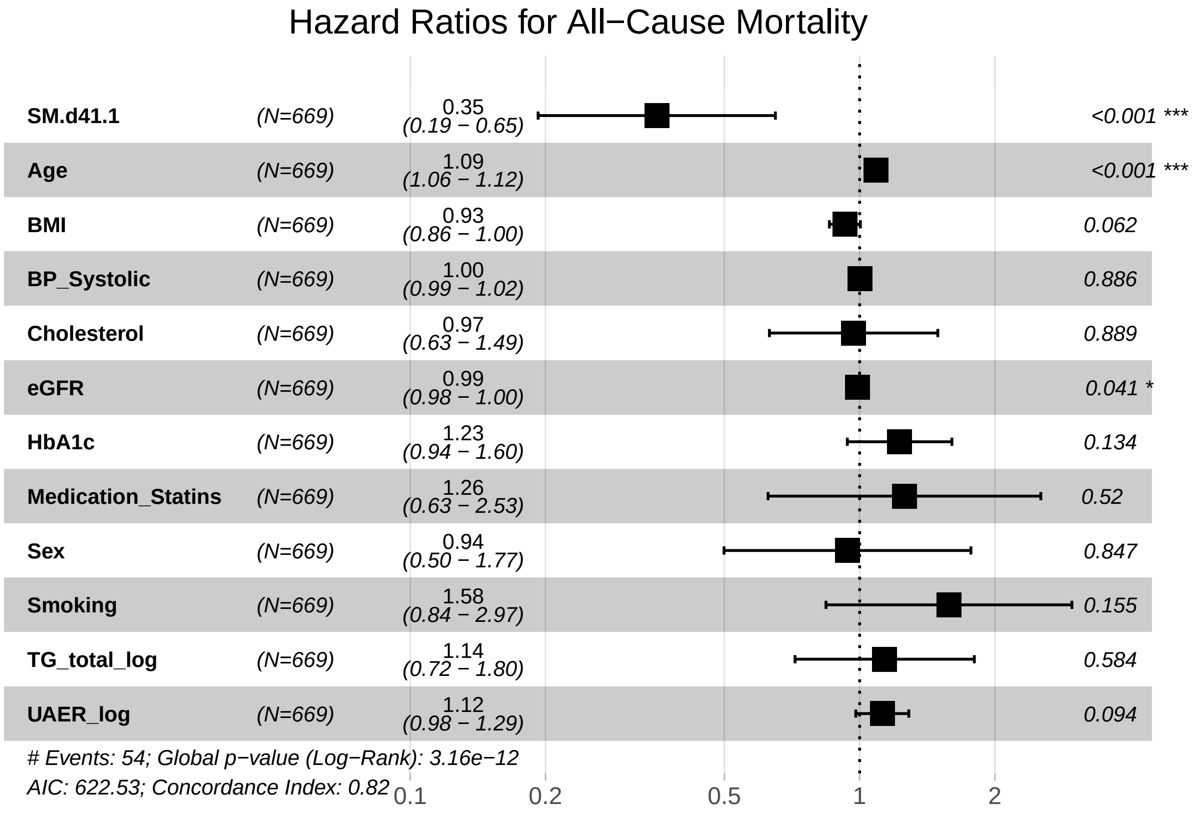

Figure 40: Forest plot of all the coefficients in the adjusted survival model for all-cause mortality. Shown in the figure are the name of the coefficient, number of observations (N), hazard ratio with 95 % confidence interval and the p-value of the association.

5.1.1.1 Forest Plot with Clinical Variables

5.1.1.2 Diagnostics of the Survival Model

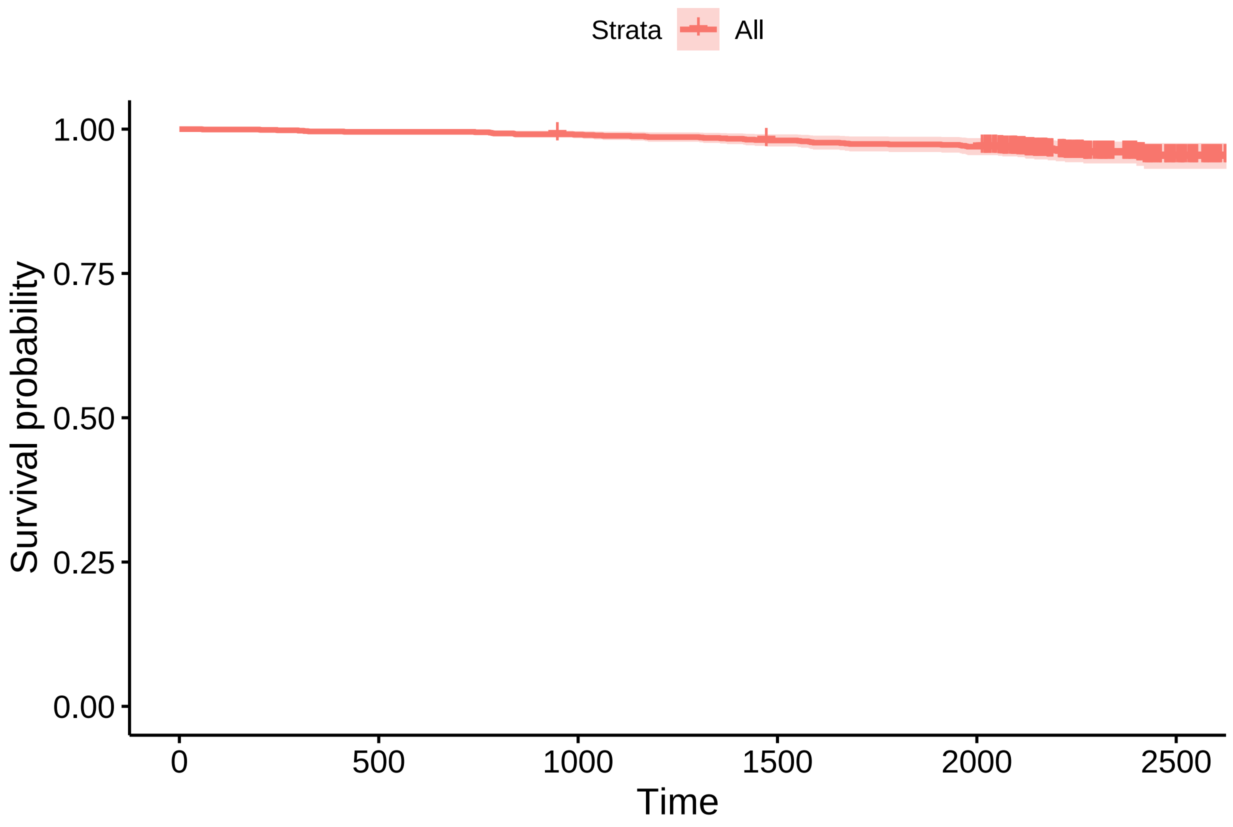
Strata
All
Survival probability
1.00
0.75
0.50
0.25
0.00
0
500
1000
1500
2000
2500
Time

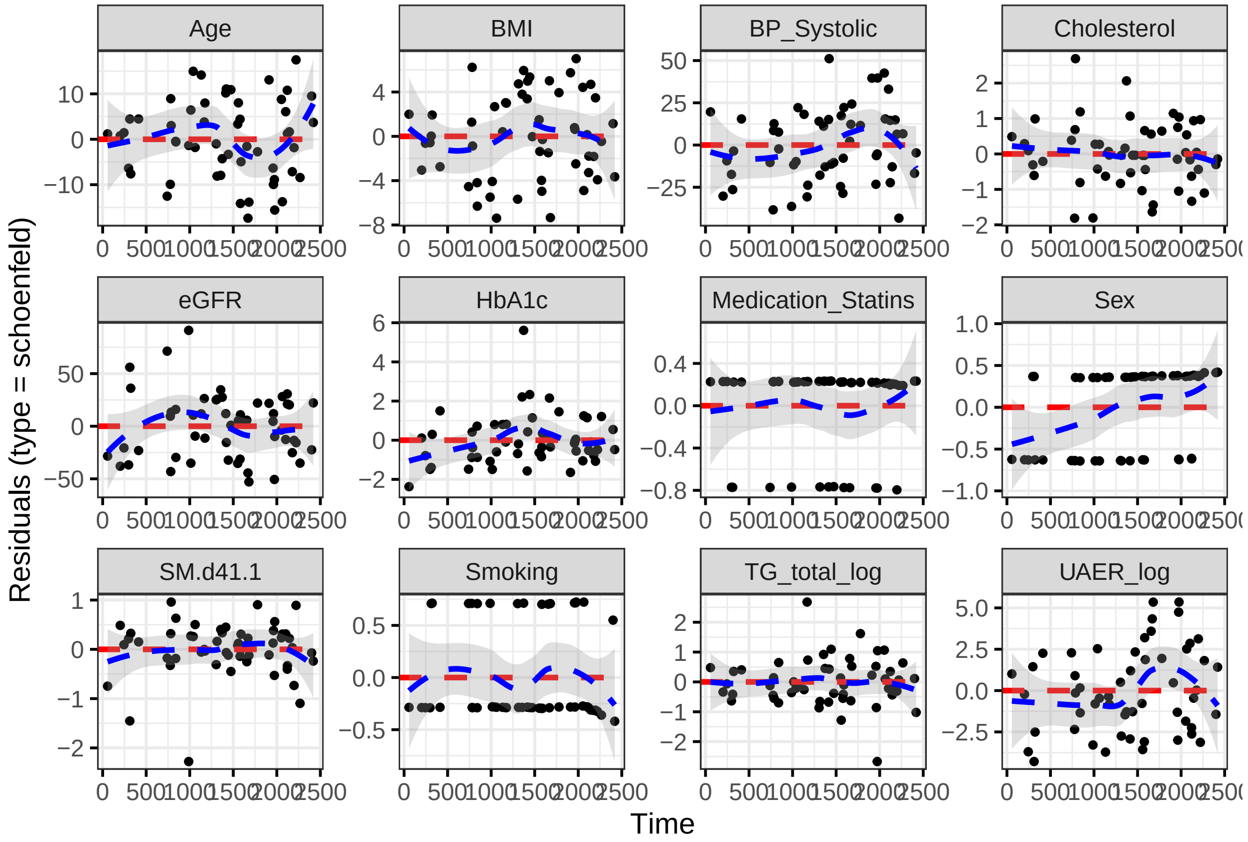
Residuals (type = schoenfeld)
Age
BMI
BP_Systolic
Cholesterol
eGFR
HbA1c
Medication_Statins
Sex
SM.d41.1
Smoking
TG_total_log
UAER_log
Time

5.1.1.3 Kaplan-Maier Curve with Median Cutpoint

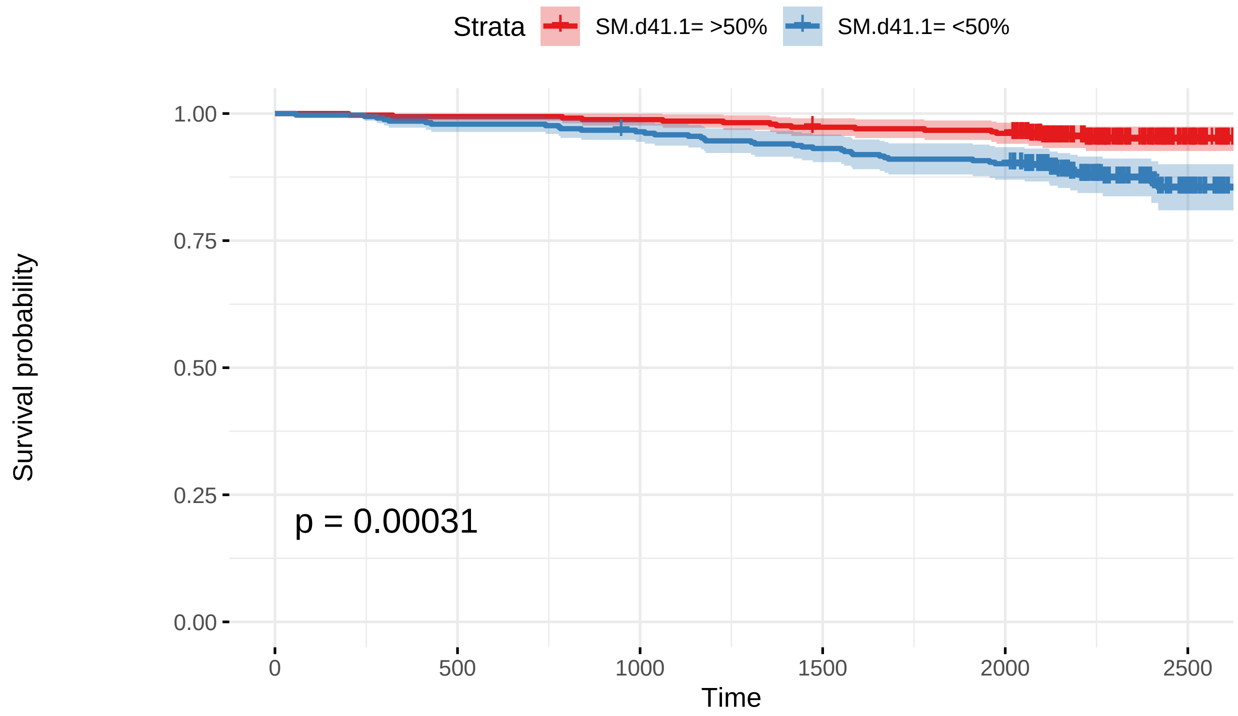

Number at risk

SM.d41.1= >50%	334	332	330	324	320	64
SM.d41.1= <50%	335	328	322	311	301	45

Cumulative number of events

SM.d41.1= >50%	0	2	4	9	13	16
SM.d41.1= <50%	0	7	12	23	33	42

Figure 41: Kaplan-Meier figure of all-cause mortality in participants with lipid SM(41:1) above or below its median level (red and blue curves, respectively). Time of follow-up and survival probablity, respectively, are shown on the x-axis and y-axis. Cumulative number of events and the number of remaining participants are shown for the two groups in the table below the figure.

5.1.2 Other Model Fits

```
## Call:
## survival::coxph(formula = survival::Surv(time = t_doed_profil,
##     event = censor_doed_profil.reversed.numeric) ~ SM.d41.1,
##     data = data.km)
##
##   n= 669, number of events= 58
##
##                  coef exp(coef) se(coef)     z Pr(>|z|)
## SM.d41.1 <50% 1.0152    2.7598   0.2939 3.454 0.000552 ***
## ---
## Signif. codes:  0 '***' 0.001 '**' 0.01 '*' 0.05 '.' 0.1 ' ' 1
##
##               exp(coef) exp(-coef) lower .95 upper .95
## SM.d41.1 <50%      2.76     0.3623     1.551      4.91
##
## Concordance= 0.614  (se = 0.033 )
## Rsquare= 0.02   (max possible= 0.669 )
## Likelihood ratio test= 13.41  on 1 df,   p=0.0002508
## Wald test            = 11.93  on 1 df,   p=0.0005523
## Score (logrank) test = 12.99  on 1 df,   p=0.0003135
```

```
##
## Call:
## glm(formula = censor_doed_profil.reversed.numeric ~ SM.d41.1 +
##     Age + BMI + BP_Systolic + Cholesterol + eGFR + HbA1c + Medication_Statins +
##     Sex + Smoking + TG_total_log + UAER_log, data = data.survival)
##
## Deviance Residuals:
##      Min        1Q    Median        3Q       Max
## -0.33989  -0.13218  -0.06706   0.00555   0.95561
##
## Coefficients:
##                      Estimate Std. Error t value Pr(>|t|)
## (Intercept)         2.1089761  0.6464134   3.263 0.001166 **
## SM.d41.1           -0.1003398  0.0294002  -3.413 0.000686 ***
## Age                 0.0047487  0.0010276   4.621 4.68e-06 ***
## BMI                -0.0055410  0.0029094  -1.904 0.057322 .
## BP_Systolic         0.0001645  0.0006808   0.242 0.809127
## Cholesterol        -0.0017448  0.0163895  -0.106 0.915254
## eGFR               -0.0008337  0.0004564  -1.827 0.068250 .
## HbA1c               0.0165920  0.0104610   1.586 0.113247
## Medication_Statins -0.0068740  0.0255750  -0.269 0.788192
## Sex                 0.0081634  0.0233883   0.349 0.727183
## Smoking             0.0365555  0.0276219   1.323 0.186195
## TG_total_log        0.0072646  0.0182897   0.397 0.691362
## UAER_log            0.0101679  0.0057618   1.765 0.078119 .
## ---
## Signif. codes:  0 '***' 0.001 '**' 0.01 '*' 0.05 '.' 0.1 ' ' 1
##
## (Dispersion parameter for gaussian family taken to be 0.07223018)
##
##     Null deviance: 49.259  on 614  degrees of freedom
```

```
## Residual deviance: 43.483  on 602  degrees of freedom
##   (54 observations deleted due to missingness)
## AIC: 144
##
## Number of Fisher Scoring iterations: 2

##
## Call:
## lm(formula = SM.d41.1 ~ censor_doed_profil.reversed.numeric +
##     Age + BMI + BP_Systolic + Cholesterol + eGFR + HbA1c + Medication_Statins +
##     Sex + Smoking + TG_total_log + UAER_log, data = data.survival)
##
## Residuals:
##      Min       1Q   Median       3Q      Max
## -2.36364 -0.19256  0.03596  0.22337  1.33335
##
## Coefficients:
##                                      Estimate Std. Error t value Pr(>|t|)
## (Intercept)                         21.3632619  0.2087609 102.334  < 2e-16
## censor_doed_profil.reversed.numeric -0.1891707  0.0554282  -3.413 0.000686
## Age                                 -0.0030716  0.0014304  -2.147 0.032158
## BMI                                 -0.0021123  0.0040059  -0.527 0.598183
## BP_Systolic                         -0.0002943  0.0009347  -0.315 0.752994
## Cholesterol                          0.3023688  0.0188298  16.058  < 2e-16
## eGFR                                -0.0006728  0.0006278  -1.072 0.284311
## HbA1c                                0.0522571  0.0142351   3.671 0.000263
## Medication_Statins                   0.0052398  0.0351176   0.149 0.881441
## Sex                                 -0.0754929  0.0319691  -2.361 0.018522
## Smoking                             -0.0448977  0.0379376  -1.183 0.237093
## TG_total_log                        -0.0260792  0.0250936  -1.039 0.299093
## UAER_log                            -0.0238898  0.0078718  -3.035 0.002510
##
## (Intercept)                         ***
## censor_doed_profil.reversed.numeric ***
## Age                                 *
## BMI
## BP_Systolic
## Cholesterol                         ***
## eGFR
## HbA1c                               ***
## Medication_Statins
## Sex                                 *
## Smoking
## TG_total_log
## UAER_log                            **
## ---
## Signif. codes:  0 '***' 0.001 '**' 0.01 '*' 0.05 '.' 0.1 ' ' 1
##
## Residual standard error: 0.369 on 602 degrees of freedom
##   (54 observations deleted due to missingness)
## Multiple R-squared:  0.3911, Adjusted R-squared:  0.379
## F-statistic: 32.23 on 12 and 602 DF,  p-value: < 2.2e-16
```

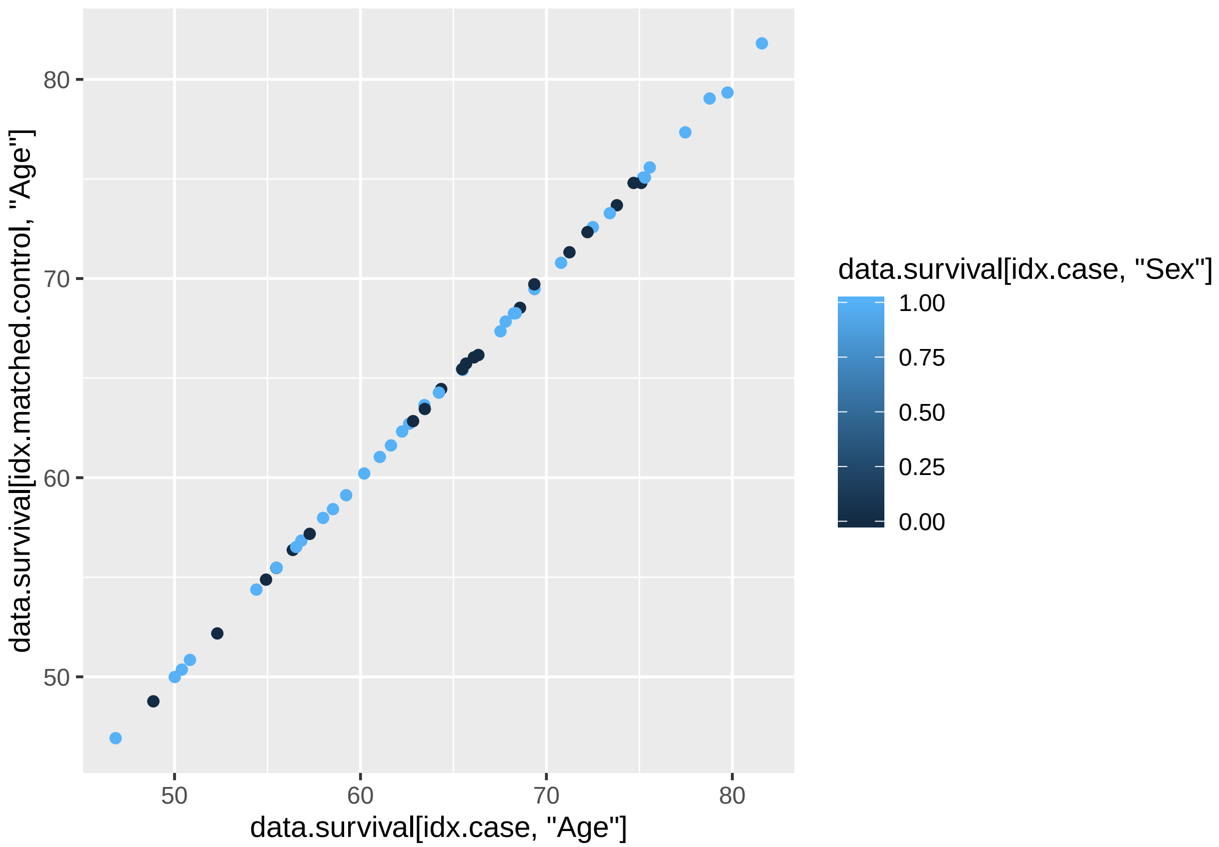

Figure 42: Quantile-quantile plot of the age of the sub-sampled cohort in persons with all-case mortality event (x-axis) and surviving persons without the event (y-axis). The two genders are shown in light and blue colors. Points falling on a diagonal line indicate a good age-matching in the two groups of the subcohort.

5.2 Step 5B: Analysis of an Age-and-Sex-Matched Subcohort

```
##
##  Paired t-test
##
## data:  data.survival[idx.case, "Age"] and data.survival[idx.matched.control, "Age"]
## t = 0.7071, df = 53, p-value = 0.4826
## alternative hypothesis: true difference in means is not equal to 0
## 95 percent confidence interval:
##  -0.02278712  0.04760194
## sample estimates:
## mean of the differences
##              0.01240741
```

```
##
## Call:
## glm(formula = censor_doed_profil.reversed.numeric ~ SM.d41.1 +
##     Age + BMI + BP_Systolic + Cholesterol + eGFR + HbA1c + Medication_Statins +
##     Sex + Smoking + TG_total_log + UAER_log, data = data.survival.stratified)
##
## Deviance Residuals:
##      Min        1Q    Median        3Q       Max
```

```
## -0.86519  -0.36291  -0.02769   0.34499   0.79471
##
## Coefficients:
##                      Estimate Std. Error t value Pr(>|t|)
## (Intercept)         9.3731947  2.5000402   3.749 0.000305 ***
## SM.d41.1           -0.3877748  0.1146773  -3.381 0.001048 **
## Age                 0.0007879  0.0057495   0.137 0.891288
## BMI                -0.0083531  0.0147462  -0.566 0.572416
## BP_Systolic        -0.0012972  0.0027038  -0.480 0.632491
## Cholesterol         0.0425939  0.0716868   0.594 0.553813
## eGFR               -0.0041364  0.0018327  -2.257 0.026302 *
## HbA1c               0.0168307  0.0432329   0.389 0.697923
## Medication_Statins  0.1714852  0.1157483   1.482 0.141773
## Sex                -0.0771974  0.1072989  -0.719 0.473623
## Smoking             0.1191930  0.1197801   0.995 0.322216
## TG_total_log        0.0135161  0.0726105   0.186 0.852727
## UAER_log            0.0181071  0.0234331   0.773 0.441610
## ---
## Signif. codes:  0 '***' 0.001 '**' 0.01 '*' 0.05 '.' 0.1 ' ' 1
##
## (Dispersion parameter for gaussian family taken to be 0.2133333)
##
##     Null deviance: 27.000  on 107  degrees of freedom
## Residual deviance: 20.267  on  95  degrees of freedom
## AIC: 153.79
##
## Number of Fisher Scoring iterations: 2
```

```
##
## Call:
## lm(formula = SM.d41.1 ~ censor_doed_profil.reversed.numeric +
##     Age + BMI + BP_Systolic + Cholesterol + eGFR + HbA1c + Medication_Statins +
##     Sex + Smoking + TG_total_log + UAER_log, data = data.survival.stratified)
##
## Residuals:
##      Min       1Q   Median       3Q      Max
## -1.08663 -0.23451  0.00349  0.24801  0.80040
##
## Coefficients:
##                                       Estimate Std. Error t value Pr(>|t|)
## (Intercept)                         21.2477338  0.6114086  34.752  < 2e-16
## censor_doed_profil.reversed.numeric -0.2770409  0.0819297  -3.381  0.00105
## Age                                 -0.0050528  0.0048325  -1.046  0.29841
## BMI                                 -0.0165859  0.0123686  -1.341  0.18313
## BP_Systolic                          0.0002579  0.0022880   0.113  0.91051
## Cholesterol                          0.3859975  0.0460084   8.390 4.51e-13
## eGFR                                -0.0027896  0.0015641  -1.784  0.07770
## HbA1c                                0.0863963  0.0354810   2.435  0.01676
## Medication_Statins                   0.1604999  0.0975797   1.645  0.10331
## Sex                                 -0.1201282  0.0901014  -1.333  0.18564
## Smoking                             -0.1916226  0.0998527  -1.919  0.05798
## TG_total_log                        -0.0340404  0.0612853  -0.555  0.57990
## UAER_log                            -0.0143670  0.0198141  -0.725  0.47018
##
```

```
## (Intercept)                         ***
## censor_doed_profil.reversed.numeric **
## Age
## BMI
## BP_Systolic
## Cholesterol                         ***
## eGFR                                .
## HbA1c                               *
## Medication_Statins
## Sex
## Smoking                             .
## TG_total_log
## UAER_log
## ---
## Signif. codes:  0 '***' 0.001 '**' 0.01 '*' 0.05 '.' 0.1 ' ' 1
##
## Residual standard error: 0.3904 on 95 degrees of freedom
## Multiple R-squared:  0.5687, Adjusted R-squared:  0.5142
## F-statistic: 10.44 on 12 and 95 DF,  p-value: 7.906e-13
```

5.2.1 Survival Model with Details

```
## Call:
## survival::coxph(formula = survival::Surv(time = t_doed_profil,
##     event = censor_doed_profil.reversed.numeric) ~ SM.d41.1 +
##     Age + BMI + BP_Systolic + Cholesterol + eGFR + HbA1c + Medication_Statins +
##     Sex + Smoking + TG_total_log + UAER_log, data = data.survival.stratified)
##
##   n= 108, number of events= 54
##
##                         coef exp(coef)  se(coef)      z Pr(>|z|)
## SM.d41.1           -1.245927  0.287674  0.373723 -3.334 0.000857 ***
## Age                 0.012702  1.012783  0.018732  0.678 0.497708
## BMI                 0.002475  1.002478  0.046936  0.053 0.957949
## BP_Systolic        -0.012498  0.987580  0.008782 -1.423 0.154694
## Cholesterol         0.192726  1.212551  0.255306  0.755 0.450319
## eGFR               -0.009692  0.990355  0.005628 -1.722 0.085052 .
## HbA1c              -0.038030  0.962684  0.147448 -0.258 0.796469
## Medication_Statins  0.844130  2.325953  0.413480  2.042 0.041199 *
## Sex                -0.655899  0.518975  0.361692 -1.813 0.069768 .
## Smoking             0.480851  1.617450  0.329439  1.460 0.144399
## TG_total_log        0.077683  1.080780  0.216428  0.359 0.719646
## UAER_log            0.055642  1.057219  0.070568  0.788 0.430408
## ---
## Signif. codes:  0 '***' 0.001 '**' 0.01 '*' 0.05 '.' 0.1 ' ' 1
##
##                    exp(coef) exp(-coef) lower .95 upper .95
## SM.d41.1              0.2877     3.4762    0.1383    0.5984
## Age                   1.0128     0.9874    0.9763    1.0507
## BMI                   1.0025     0.9975    0.9144    1.0991
## BP_Systolic           0.9876     1.0126    0.9707    1.0047
## Cholesterol           1.2126     0.8247    0.7352    1.9999
## eGFR                  0.9904     1.0097    0.9795    1.0013
## HbA1c                 0.9627     1.0388    0.7211    1.2853
## Medication_Statins    2.3260     0.4299    1.0343    5.2307
## Sex                   0.5190     1.9269    0.2554    1.0544
## Smoking               1.6174     0.6183    0.8480    3.0850
## TG_total_log          1.0808     0.9253    0.7072    1.6518
## UAER_log              1.0572     0.9459    0.9207    1.2140
##
## Concordance= 0.697  (se = 0.041 )
## Rsquare= 0.232   (max possible= 0.986 )
## Likelihood ratio test= 28.45  on 12 df,   p=0.004748
## Wald test            = 27.6  on 12 df,   p=0.006318
## Score (logrank) test = 29.31  on 12 df,   p=0.00354
```

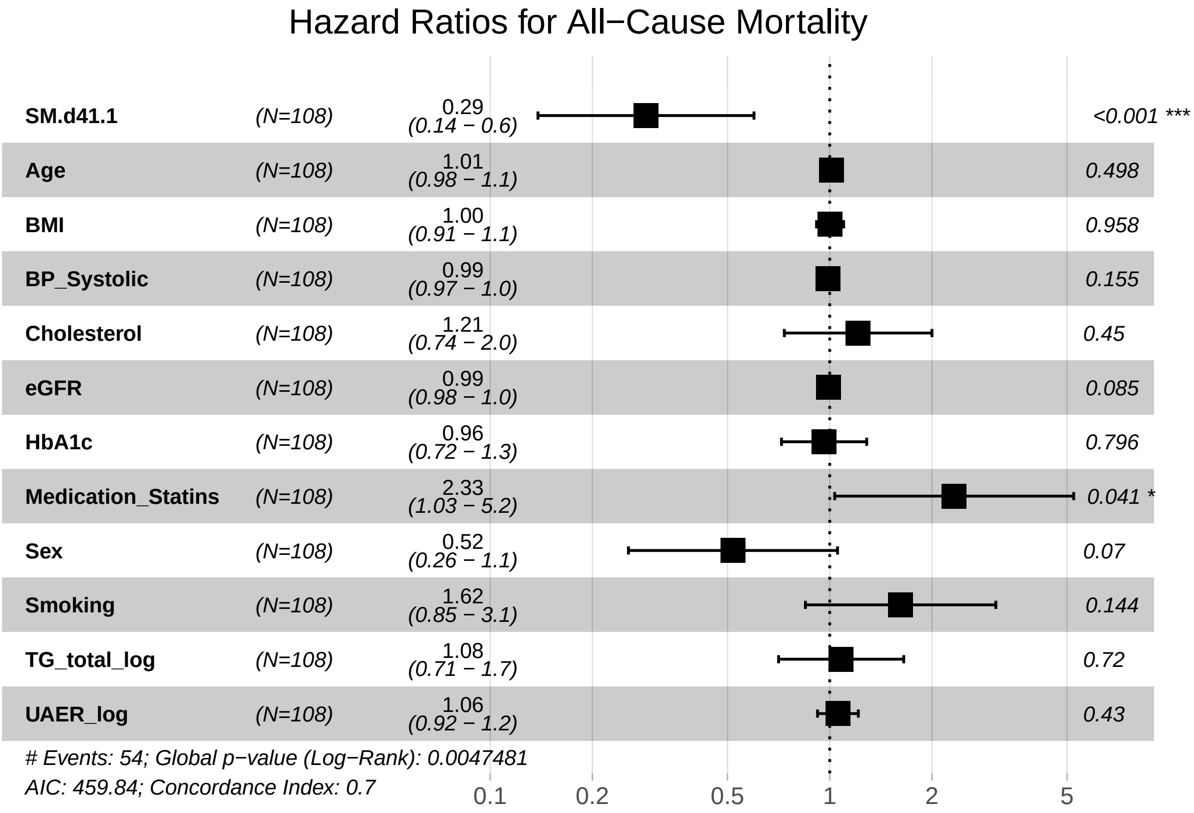

Figure 43: Forest plot of all the coefficients in the adjusted survival model for all-cause mortality. Shown in the figure are the name of the coefficient, number of observations (N), hazard ratio with 95 % confidence interval and the p-value of the association.

5.2.1.1 Forest Plot with Clinical Variables

5.2.1.2 Diagnostics of the Survival Model

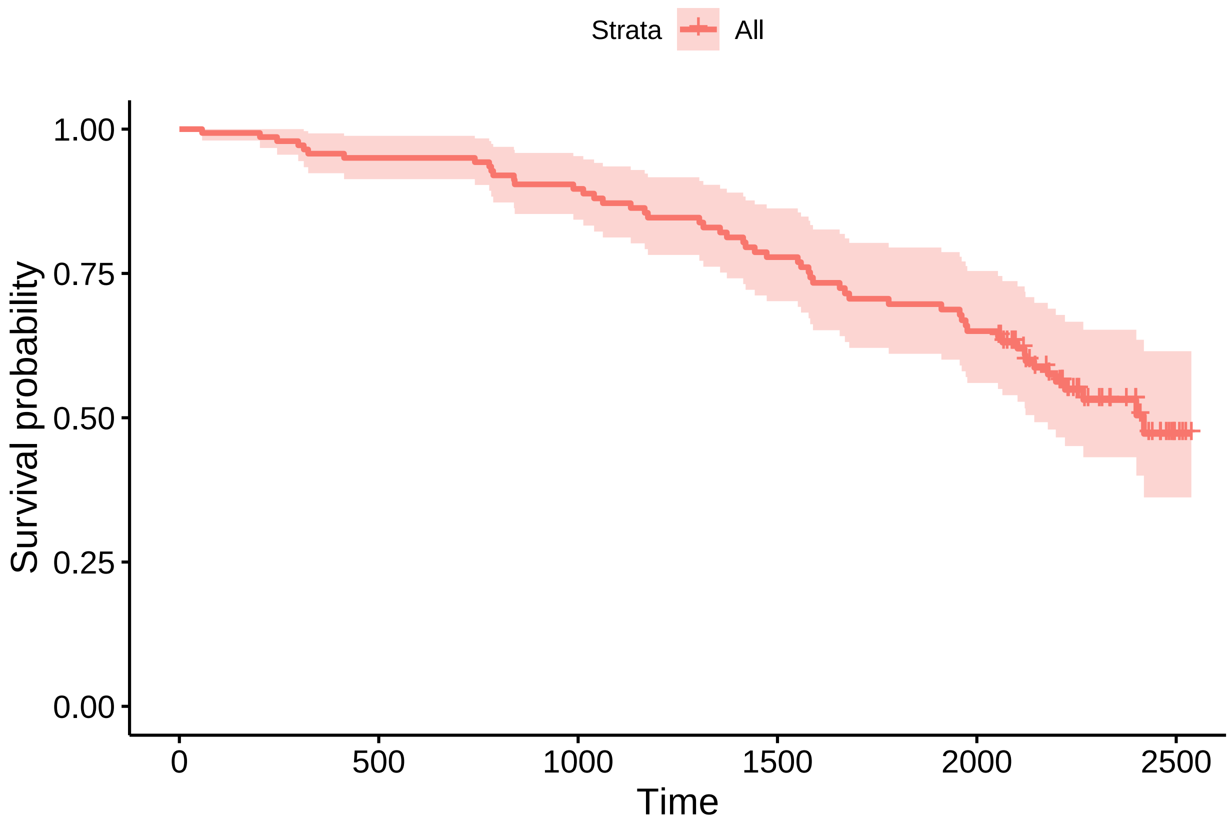
Strata
All
Survival probability
1.00
0.75
0.50
0.25
0.00
0
500
1000
1500
2000
2500
Time

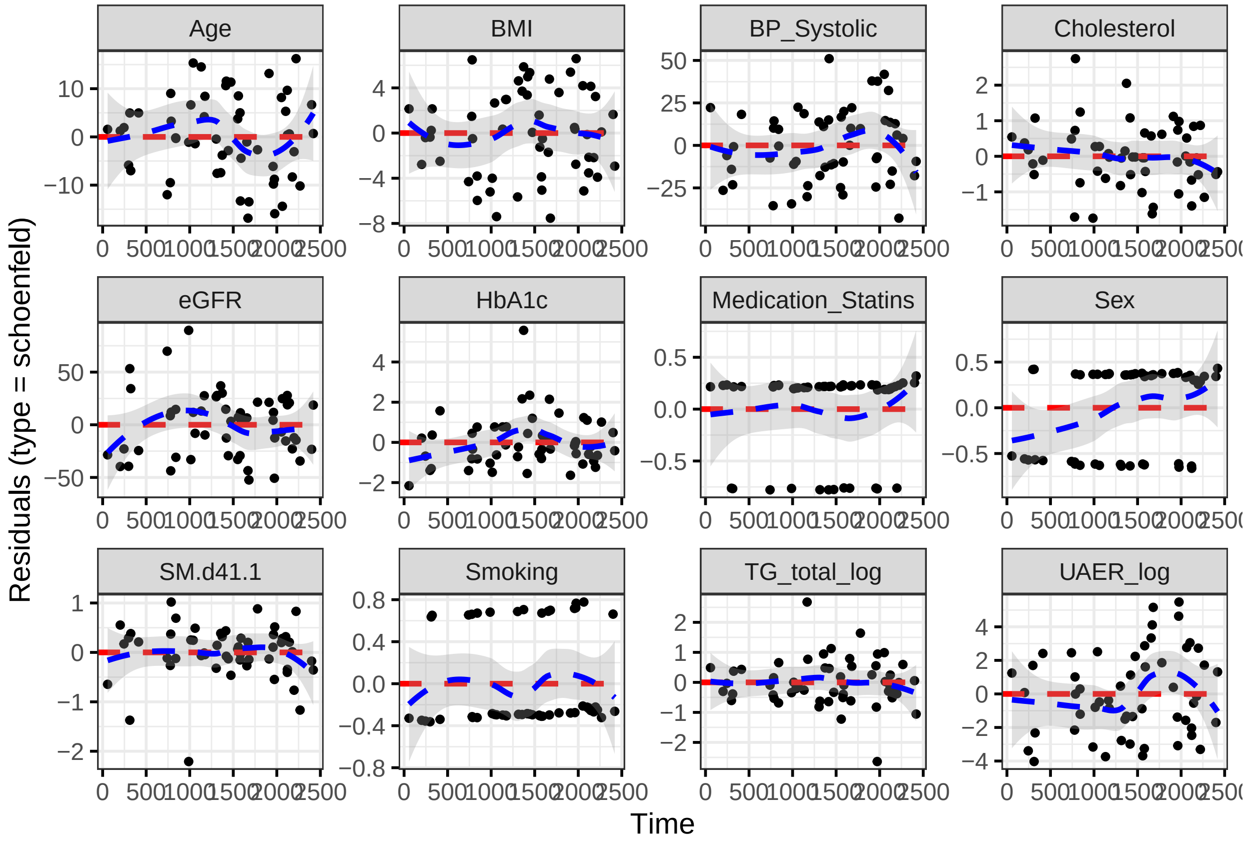
Age
BMI
BP_Systolic
Cholesterol
eGFR
HbA1c
Medication_Statins
Sex
SM.d41.1
Smoking
TG_total_log
UAER_log
Residuals (type = schoenfeld)
Time

5.2.1.3 Kaplan-Maier Curve with Median Cutpoint

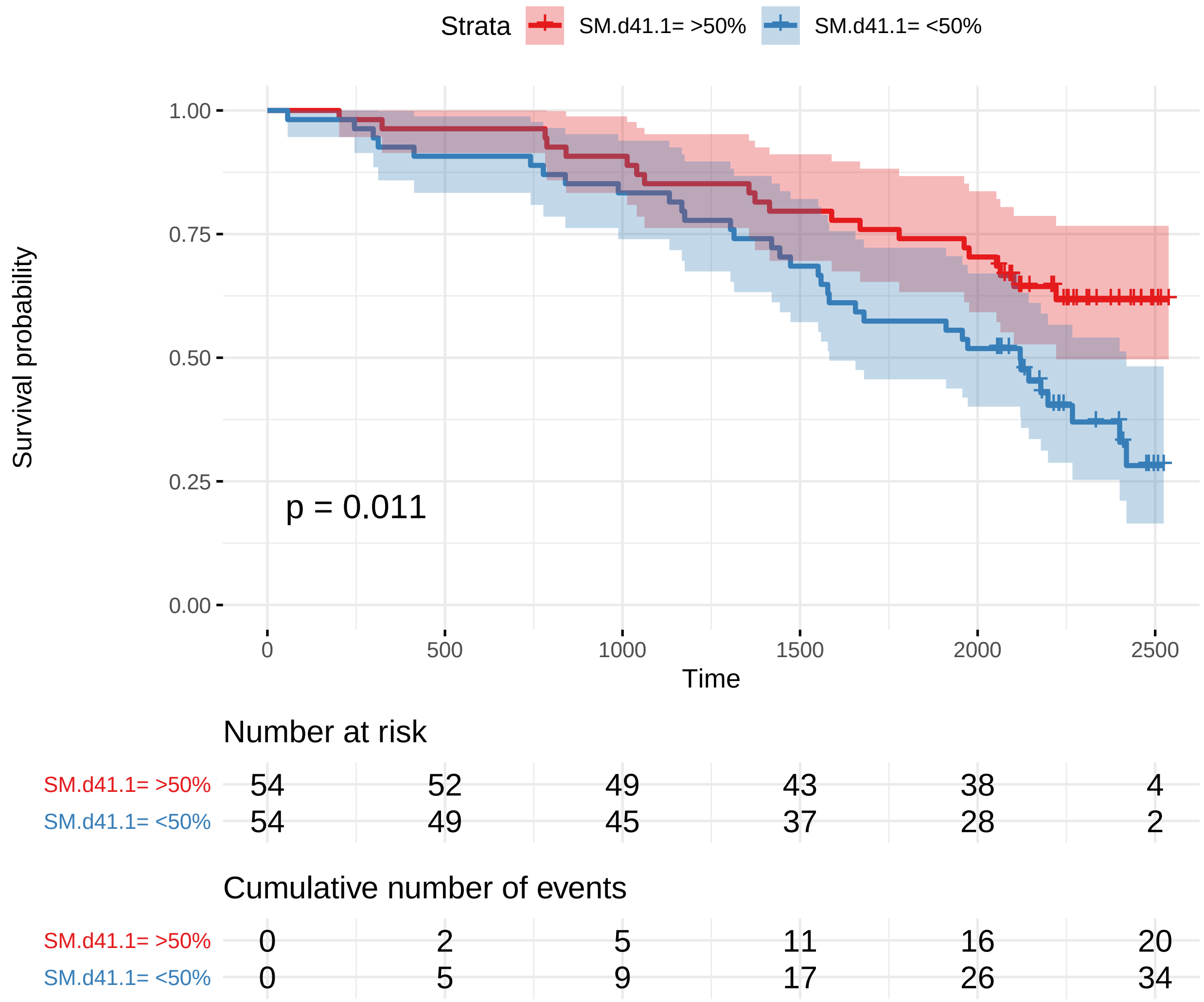

Figure 44: Kaplan-Meier figure of all-cause mortality in participants with lipid SM(41:1) above or below its median level (red and blue curves, respectively). Time of follow-up and survival probablity, respectively, are shown on the x-axis and y-axis. Cumulative number of events and the number of remaining participants are shown for the two groups in the table below the figure.

5.2.2 Other Model Fits

```
## Call:
## survival::coxph(formula = survival::Surv(time = t_doed_profil,
##     event = censor_doed_profil.reversed.numeric) ~ SM.d41.1,
##     data = data.km)
##
##   n= 108, number of events= 54
##
##                  coef exp(coef) se(coef)     z Pr(>|z|)
## SM.d41.1 <50% 0.7078    2.0296   0.2824 2.506   0.0122 *
## ---
## Signif. codes:  0 '***' 0.001 '**' 0.01 '*' 0.05 '.' 0.1 ' ' 1
##
##               exp(coef) exp(-coef) lower .95 upper .95
## SM.d41.1 <50%      2.03     0.4927     1.167      3.53
##
## Concordance= 0.577  (se = 0.036 )
## Rsquare= 0.059   (max possible= 0.986 )
## Likelihood ratio test= 6.55  on 1 df,   p=0.01051
## Wald test            = 6.28  on 1 df,   p=0.01219
## Score (logrank) test = 6.55  on 1 df,   p=0.01051
```

```
##
## Call:
## glm(formula = censor_doed_profil.reversed.numeric ~ SM.d41.1 +
##     Age + BMI + BP_Systolic + Cholesterol + eGFR + HbA1c + Medication_Statins +
##     Sex + Smoking + TG_total_log + UAER_log, data = data.survival.stratified)
##
## Deviance Residuals:
##      Min        1Q    Median        3Q       Max
## -0.86519  -0.36291  -0.02769   0.34499   0.79471
##
## Coefficients:
##                     Estimate Std. Error t value Pr(>|t|)
## (Intercept)         9.3731947  2.5000402   3.749 0.000305 ***
## SM.d41.1           -0.3877748  0.1146773  -3.381 0.001048 **
## Age                 0.0007879  0.0057495   0.137 0.891288
## BMI                -0.0083531  0.0147462  -0.566 0.572416
## BP_Systolic        -0.0012972  0.0027038  -0.480 0.632491
## Cholesterol         0.0425939  0.0716868   0.594 0.553813
## eGFR               -0.0041364  0.0018327  -2.257 0.026302 *
## HbA1c               0.0168307  0.0432329   0.389 0.697923
## Medication_Statins  0.1714852  0.1157483   1.482 0.141773
## Sex                -0.0771974  0.1072989  -0.719 0.473623
## Smoking             0.1191930  0.1197801   0.995 0.322216
## TG_total_log        0.0135161  0.0726105   0.186 0.852727
## UAER_log            0.0181071  0.0234331   0.773 0.441610
## ---
## Signif. codes:  0 '***' 0.001 '**' 0.01 '*' 0.05 '.' 0.1 ' ' 1
##
## (Dispersion parameter for gaussian family taken to be 0.2133333)
##
##     Null deviance: 27.000  on 107  degrees of freedom
```

```
## Residual deviance: 20.267  on  95  degrees of freedom
## AIC: 153.79
##
## Number of Fisher Scoring iterations: 2
```

```
##
## Call:
## lm(formula = SM.d41.1 ~ censor_doed_profil.reversed.numeric +
##     Age + BMI + BP_Systolic + Cholesterol + eGFR + HbA1c + Medication_Statins +
##     Sex + Smoking + TG_total_log + UAER_log, data = data.survival.stratified)
##
## Residuals:
##      Min       1Q   Median       3Q      Max
## -1.08663 -0.23451  0.00349  0.24801  0.80040
##
## Coefficients:
##                                      Estimate Std. Error t value Pr(>|t|)
## (Intercept)                         21.2477338  0.6114086  34.752  < 2e-16
## censor_doed_profil.reversed.numeric -0.2770409  0.0819297  -3.381  0.00105
## Age                                 -0.0050528  0.0048325  -1.046  0.29841
## BMI                                 -0.0165859  0.0123686  -1.341  0.18313
## BP_Systolic                          0.0002579  0.0022880   0.113  0.91051
## Cholesterol                          0.3859975  0.0460084   8.390 4.51e-13
## eGFR                                -0.0027896  0.0015641  -1.784  0.07770
## HbA1c                                0.0863963  0.0354810   2.435  0.01676
## Medication_Statins                   0.1604999  0.0975797   1.645  0.10331
## Sex                                 -0.1201282  0.0901014  -1.333  0.18564
## Smoking                             -0.1916226  0.0998527  -1.919  0.05798
## TG_total_log                        -0.0340404  0.0612853  -0.555  0.57990
## UAER_log                            -0.0143670  0.0198141  -0.725  0.47018
##
## (Intercept)                         ***
## censor_doed_profil.reversed.numeric **
## Age
## BMI
## BP_Systolic
## Cholesterol                         ***
## eGFR                                .
## HbA1c                               *
## Medication_Statins
## Sex
## Smoking                             .
## TG_total_log
## UAER_log
## ---
## Signif. codes:  0 '***' 0.001 '**' 0.01 '*' 0.05 '.' 0.1 ' ' 1
##
## Residual standard error: 0.3904 on 95 degrees of freedom
## Multiple R-squared:  0.5687, Adjusted R-squared:  0.5142
## F-statistic: 10.44 on 12 and 95 DF,  p-value: 7.906e-13
```

6 Correlation Matrix

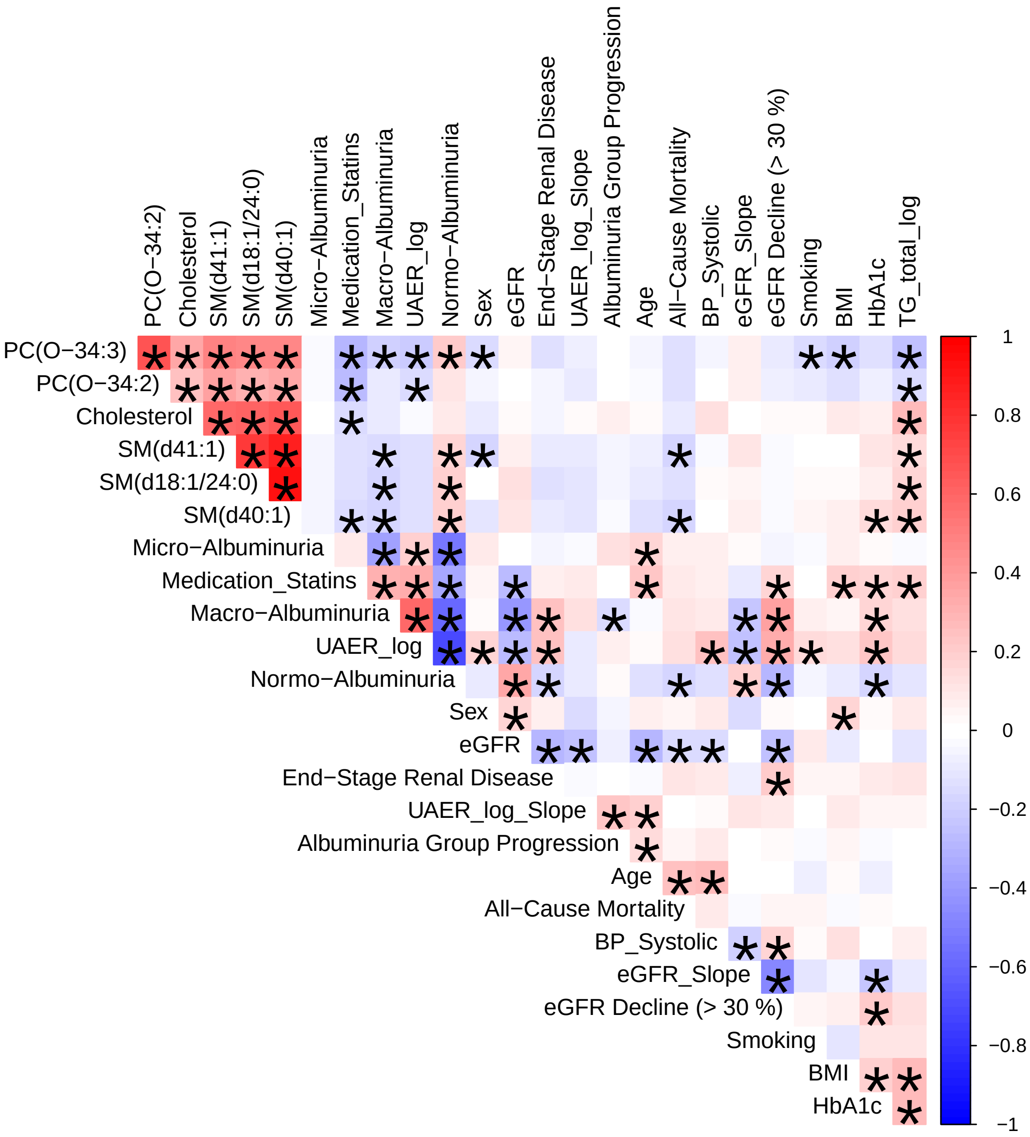

Figure 45: Correlation matrix of relevant variables. Variables are shown on the rows and columns. Spearman correlation of two variables is shown as the color of the square at the intersection of the row and column that corresponds to the two variables. Blue, red and white, respectively, indicate inverse, positive and no correlation. Correlations with statistical significance at p < 0.05 after correction for multiple testing are labeled with asterisk.

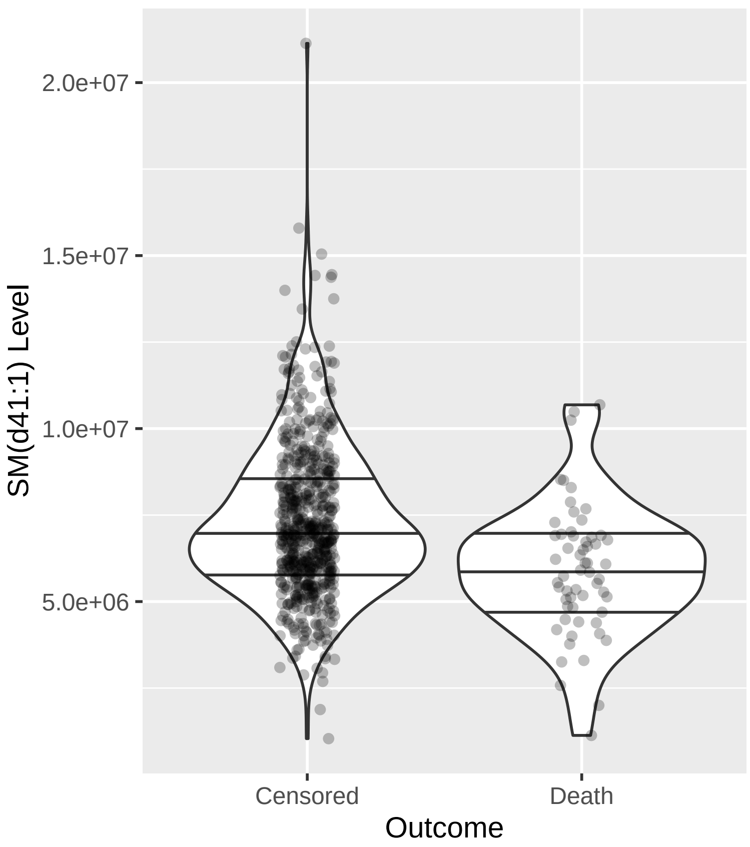

Figure 46: Violin plot of observations of the level of the lipid SM(d41:1). Subpopulations of the study are shown on the x-axis and the level of the lipid is shown on the y-axis. The density of the observations is shown in the shape of the violin and the quantiles of the distribution are shown as horizontal lines.

7 Boxplots

7.1 Entire Cohort

```
## [1] ""
## [1] ""
## [1] ""
## [1] ""
## `geom_smooth()` using method = 'loess' and formula 'y ~ x'
```

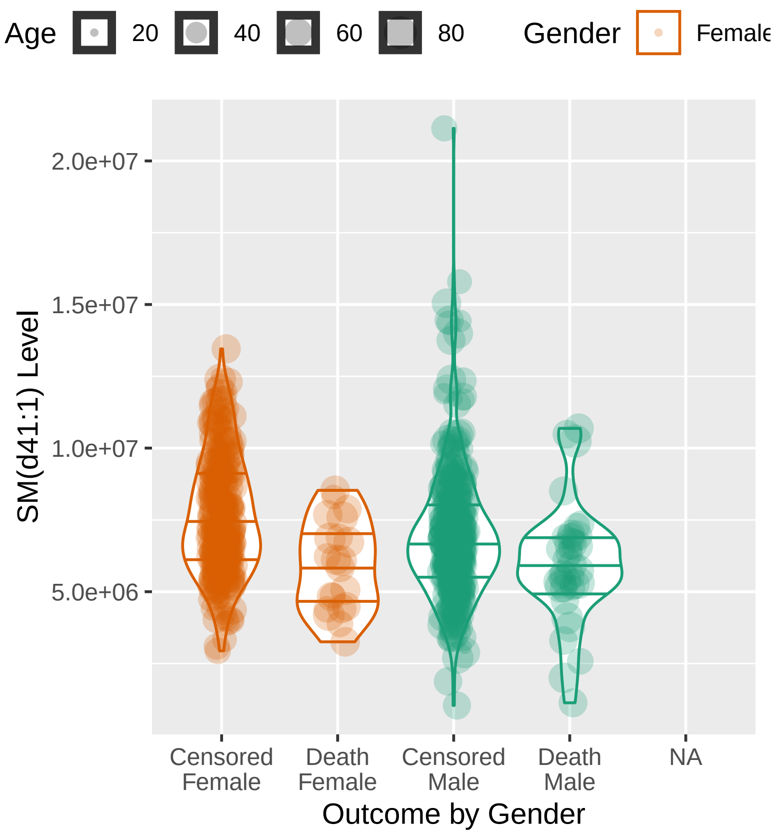

Figure 47: Violin plot of observations of the level of the lipid SM(d41:1). Subpopulations of the study are shown on the x-axis and the level of the lipid is shown on the y-axis. The density of the observations is shown in the shape of the violin and the quantiles of the distribution are shown as horizontal lines.

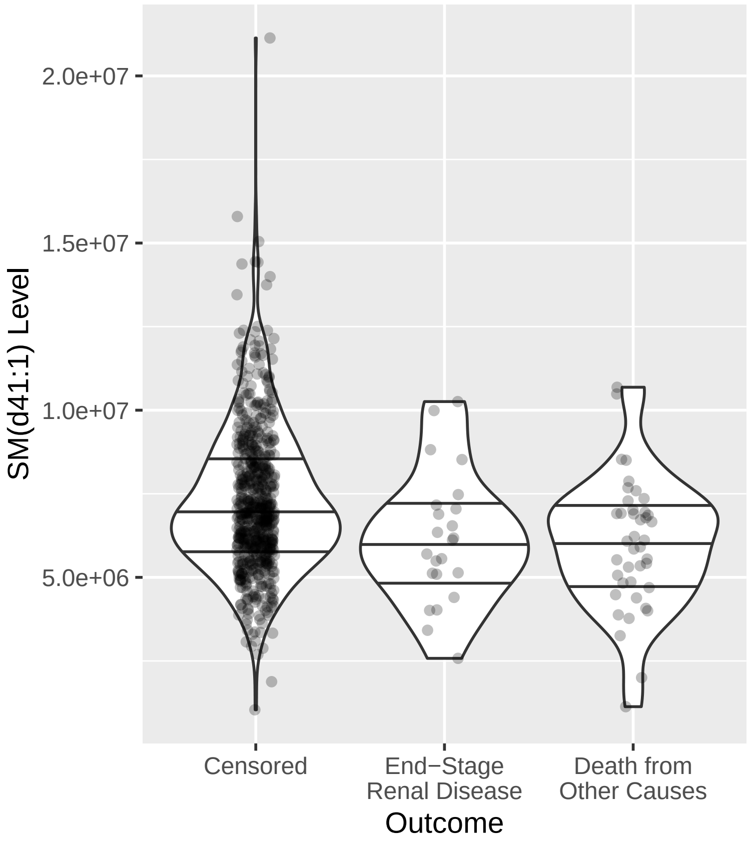

Figure 48: Violin plot of observations of the level of the lipid SM(d41:1). Subpopulations of the study are shown on the x-axis and the level of the lipid is shown on the y-axis. The density of the observations is shown in the shape of the violin and the quantiles of the distribution are shown as horizontal lines.

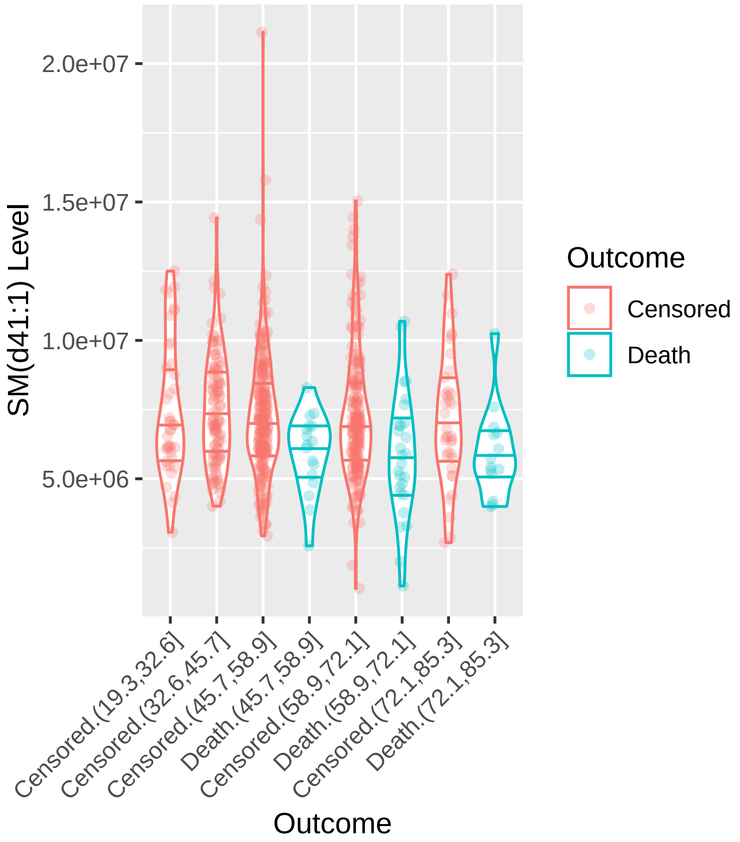

Figure 49: Violin plot of observations of the level of the lipid SM(d41:1). Subpopulations of the study are shown on the x-axis and the level of the lipid is shown on the y-axis. The density of the observations is shown in the shape of the violin and the quantiles of the distribution are shown as horizontal lines.

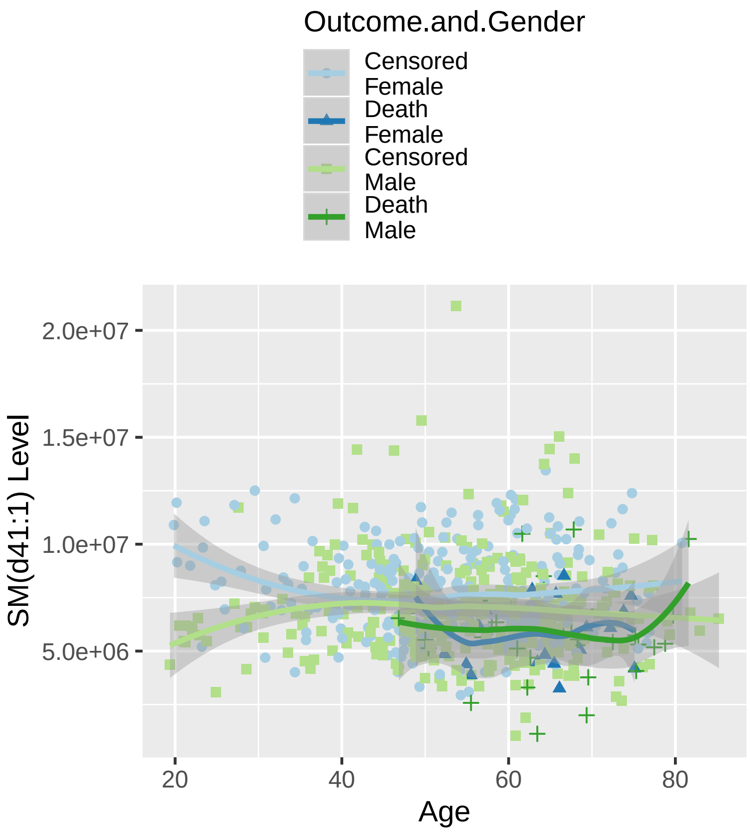

Figure 50: Observations of the level of the lipid SM(d41:1) against age. Subpopulations of the study are in different color. Possible trend in the observations is shown with a subgroup-specific nonlinear regression with the LOESS model.

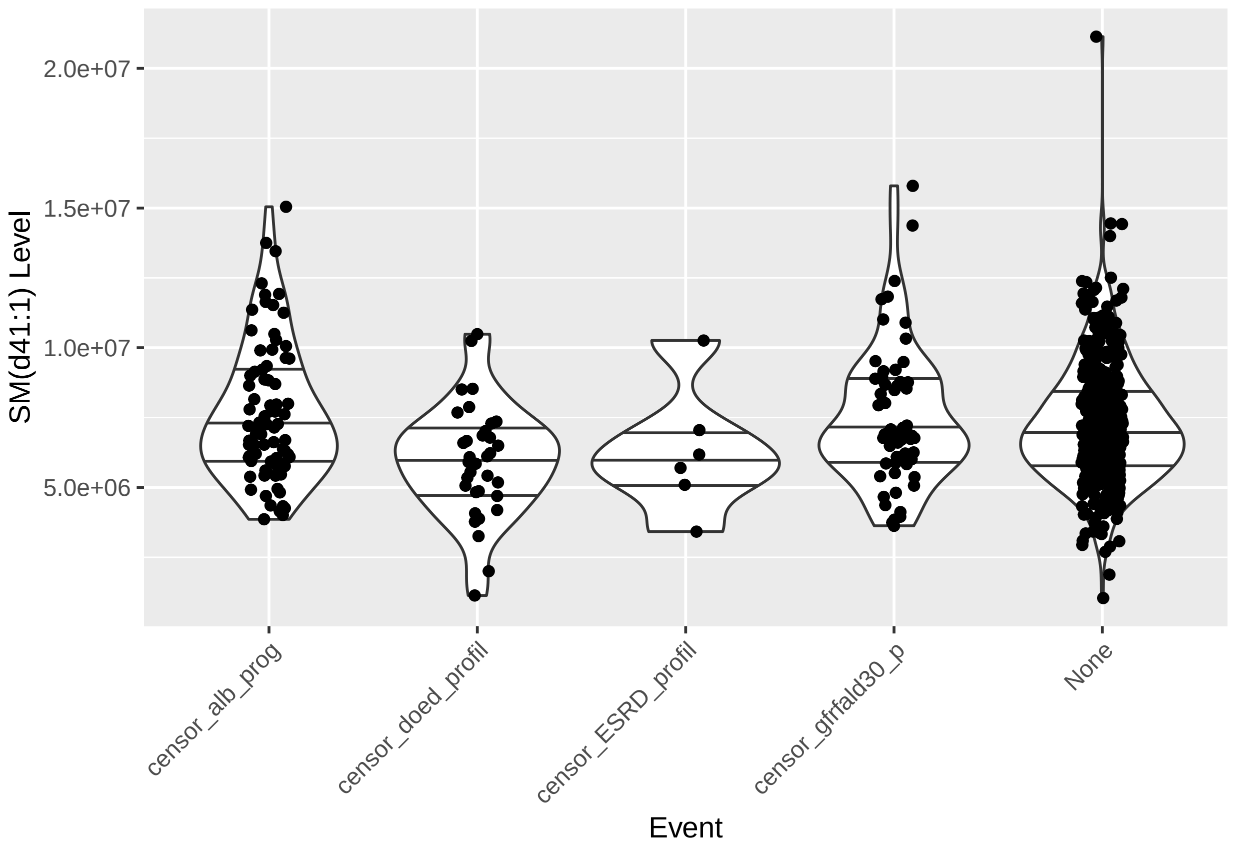

Figure 51: Violin plot of observations of the level of the lipid SM(d41:1). Subpopulations of the study are shown on the x-axis and the level of the lipid is shown on the y-axis. The density of the observations is shown in the shape of the violin and the quantiles of the distribution are shown as horizontal lines.

7.2 Age-and-Sex-Matched Subcohort

```
## [1] ""
## [1] ""
## [1] ""
## [1] ""
```

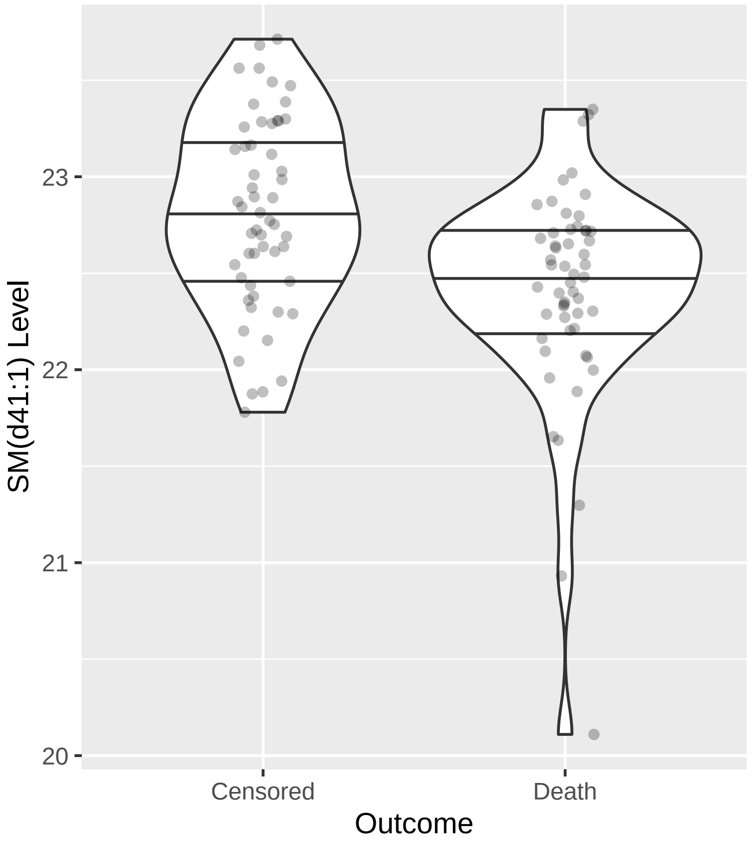

Figure 52: Violin plot of observations of the level of the lipid SM(d41:1). Subpopulations of the study are shown on the x-axis and the level of the lipid is shown on the y-axis. The density of the observations is shown in the shape of the violin and the quantiles of the distribution are shown as horizontal lines.

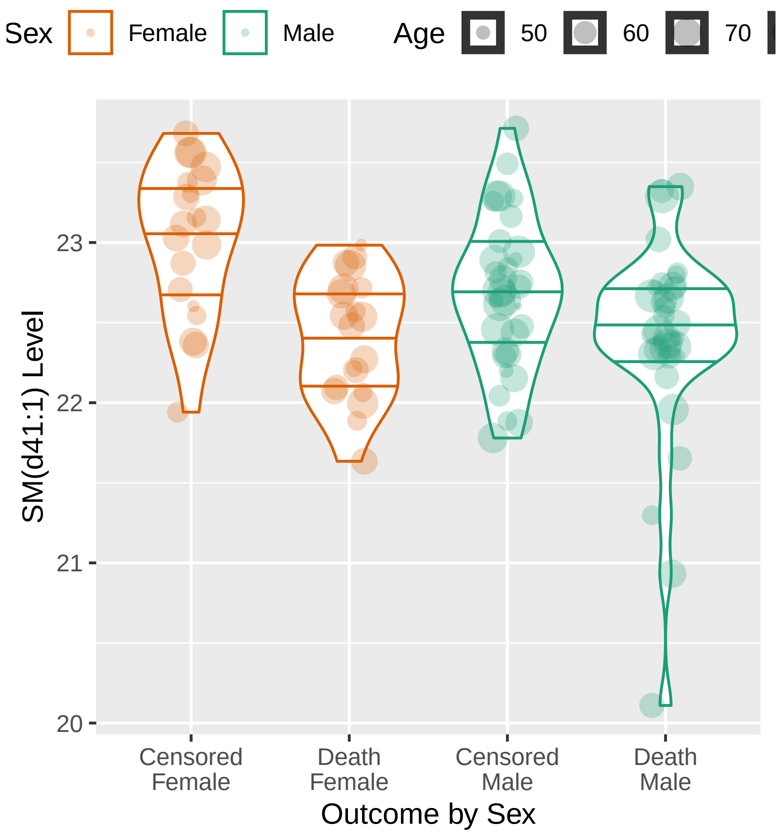

Figure 53: Violin plot of observations of the level of the lipid SM(d41:1). Subpopulations of the study are shown on the x-axis and the level of the lipid is shown on the y-axis. The density of the observations is shown in the shape of the violin and the quantiles of the distribution are shown as horizontal lines.

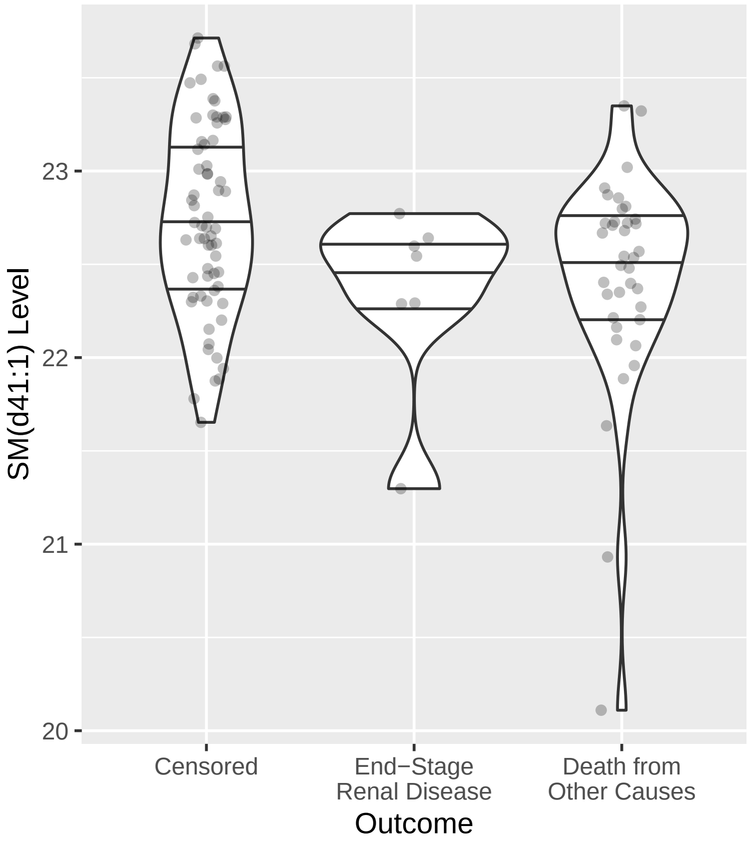

Figure 54: Violin plot of observations of the level of the lipid SM(d41:1). Subpopulations of the study are shown on the x-axis and the level of the lipid is shown on the y-axis. The density of the observations is shown in the shape of the violin and the quantiles of the distribution are shown as horizontal lines.

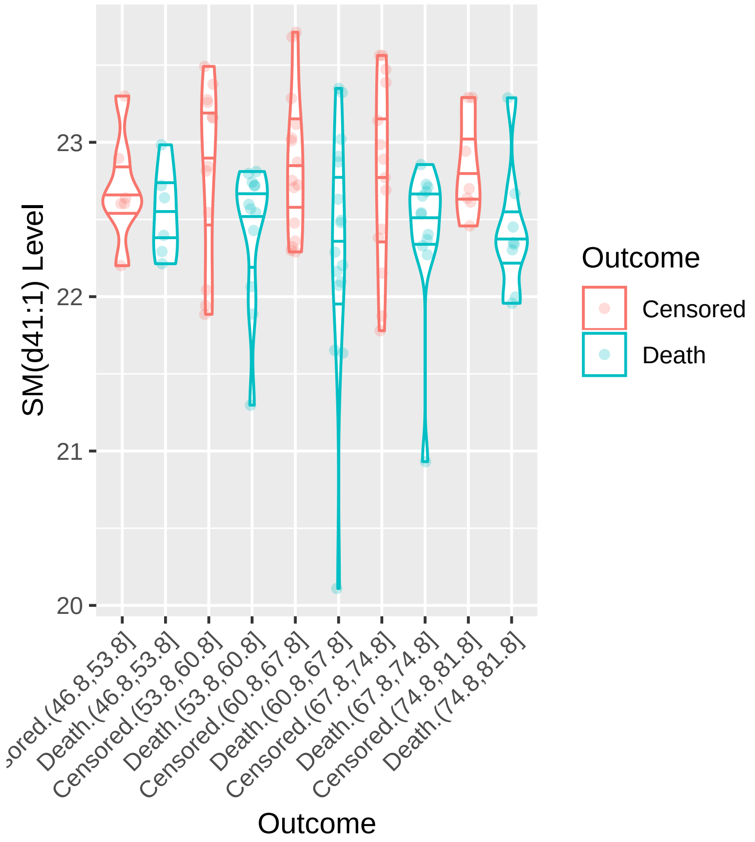

Figure 55: Violin plot of observations of the level of the lipid SM(d41:1). Subpopulations of the study are shown on the x-axis and the level of the lipid is shown on the y-axis. The density of the observations is shown in the shape of the violin and the quantiles of the distribution are shown as horizontal lines.

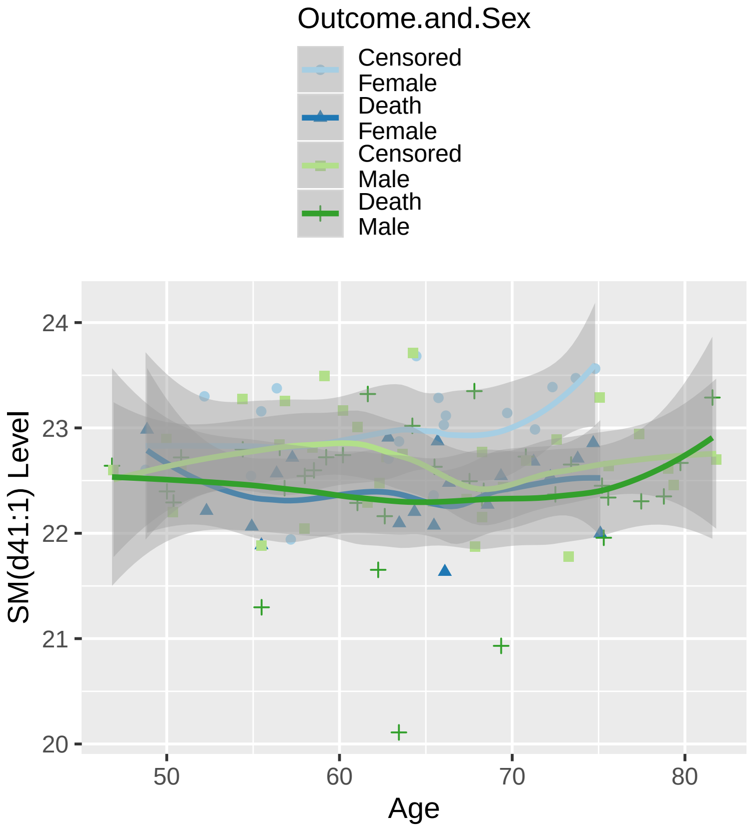

Figure 56: Observations of the level of the lipid SM(d41:1) against age. Subpopulations of the study are in different color. Possible trend in the observations is shown with a subgroup-specific nonlinear regression with the LOESS model.

```
## `geom_smooth()` using method = 'loess' and formula 'y ~ x'
```

8 Lipids' Proportion of the Total Amount of Lipid Class

```
##
##
## Table: List of lipids in the class LPC
## and the lipid's respective proportion of observed signal within the class.
## Shown are each lipid's rank (Rank),
## name (Name),
## class (Class) and
## proportion of the lipid in its class (Pcnt.of.Class; %).
##
##  Rank  Name        Class   Pcnt.of.Class
## -----  ----------  ------  --------------
##     1  LPC(16:0)   LPC     47
##     2  LPC(18:0)   LPC     20
##     3  LPC(18:2)   LPC     16
##     4  LPC(18:1)   LPC     13
##     5  LPC(20:4)   LPC     2.9
##     6  LPC(16:1)   LPC     1.2
##
##
## Table: List of lipids in the class PC
## and the lipid's respective proportion of observed signal within the class.
## Shown are each lipid's rank (Rank),
## name (Name),
## class (Class) and
## proportion of the lipid in its class (Pcnt.of.Class; %).
##
##  Rank  Name       Class   Pcnt.of.Class
## -----  ---------  ------  --------------
##     1  PC(34:2)   PC      13
##     2  PC(36:2)   PC      13
##     3  PC(36:4)   PC      12
##     4  PC(34:1)   PC      12
##     5  PC(36:3)   PC      10
##     6  PC(38:6)   PC      8.2
##     7  PC(38:4)   PC      7.3
##     8  PC(38:5)   PC      4.3
##     9  PC(36:5)   PC      4.1
##    10  PC(38:3)   PC      4
##    11  PC(40:6)   PC      3.5
##    12  PC(32:1)   PC      2.1
##    13  PC(34:3)   PC      1.2
##    14  PC(32:0)   PC      1
##    15  PC(40:5)   PC      0.85
##    16  PC(35:2)   PC      0.69
##    17  PC(35:1)   PC      0.66
##    18  PC(40:7)   PC      0.5
##    19  PC(38:2)   PC      0.48
##    20  PC(32:2)   PC      0.43
##    21  PC(33:1)   PC      0.37
##    22  PC(37:2)   PC      0.13
##
##
```

```
## Table: List of lipids in the class PC-O/P
## and the lipid's respective proportion of observed signal within the class.
## Shown are each lipid's rank (Rank),
## name (Name),
## class (Class) and
## proportion of the lipid in its class (Pcnt.of.Class; %).
##
##  Rank  Name         Class    Pcnt.of.Class
## -----  -----------  -------  --------------
##     1  PC(O-36:4)   PC-O/P   22
##     2  PC(O-38:5)   PC-O/P   19
##     3  PC(O-36:5)   PC-O/P   16
##     4  PC(O-34:3)   PC-O/P   12
##     5  PC(O-38:4)   PC-O/P   10
##     6  PC(O-34:2)   PC-O/P   9.1
##     7  PC(O-36:2)   PC-O/P   4
##     8  PC(O-38:6)   PC-O/P   3.9
##     9  PC(O-36:3)   PC-O/P   3.8
##
##
## Table: List of lipids in the class SM
## and the lipid's respective proportion of observed signal within the class.
## Shown are each lipid's rank (Rank),
## name (Name),
## class (Class) and
## proportion of the lipid in its class (Pcnt.of.Class; %).
##
##  Rank  Name             Class   Pcnt.of.Class
## -----  ---------------  ------  --------------
##     1  SM(42:2)         SM      20
##     2  SM(d34:1)        SM      16
##     3  SM(d40:1)        SM      9.6
##     4  SM(d18:2/24:1)   SM      8.6
##     5  SM(d40:2)        SM      7.6
##     6  SM(d18:1/24:0)   SM      7.3
##     7  SM(d36:1)        SM      6
##     8  SM(d38:1)        SM      5.3
##     9  SM(d41:1)        SM      4.3
##    10  SM(d16:1/18:1)   SM      3.7
##    11  SM(d41:2)        SM      3.3
##    12  SM(d32:1)        SM      2.7
##    13  SM(d36:2)        SM      1.5
##    14  SM(d38:2)        SM      1.5
##    15  SM(d39:1)        SM      1.4
##    16  SM(d33:1)        SM      1.4
##
##
## Table: List of lipids in the class TG
## and the lipid's respective proportion of observed signal within the class.
## Shown are each lipid's rank (Rank),
## name (Name),
## class (Class) and
## proportion of the lipid in its class (Pcnt.of.Class; %).
##
```

```
##  Rank  Name                  Class   Pcnt.of.Class
## -----  -------------------  ------  --------------
##     1  TG(18:1/18:1/16:0)    TG      12
##     2  TG(18:2/18:1/16:0)    TG      11
##     3  TG(16:0/18:2/18:2)    TG      7.4
##     4  TG(18:1/18:1/18:1)    TG      6.6
##     5  TG(14:0/18:1/18:1)    TG      6.4
##     6  TG(50:1)              TG      6.1
##     7  TG(18:2/18:1/18:1)    TG      5.6
##     8  TG(50:3)              TG      3.6
##     9  TG(18:1/18:2/18:2)    TG      3.5
##    10  TG(16:0/18:0/18:1)    TG      2.9
##    11  TG(54:2)              TG      2.8
##    12  TG(14:0/16:0/18:1)    TG      2.6
##    13  TG(18:1/12:0/18:1)    TG      2
##    14  TG(45:0)              TG      1.9
##    15  TG(16:0/22:5/18:1)    TG      1.8
##    16  TG(18:2/22:5/16:0)    TG      1.6
##    17  TG(16:0/18:2/18:3)    TG      1.6
##    18  TG(52:2)              TG      1.5
##    19  TG(18:2/18:2/18:2)    TG      1.4
##    20  TG(52:3)              TG      1.3
##    21  TG(51:2)              TG      1
##    22  TG(14:0/18:2/18:2)    TG      0.95
##    23  TG(16:0/18:2/22:6)    TG      0.94
##    24  TG(52:4)              TG      0.89
##    25  TG(46:1)              TG      0.83
##    26  TG(54:3)              TG      0.75
##    27  TG(48:3)              TG      0.65
##    28  TG(18:0/18:1/20:4)    TG      0.63
##    29  TG(54:4)              TG      0.62
##    30  TG(50:0)              TG      0.61
##    31  TG(50:2)              TG      0.61
##    32  TG(49:1)              TG      0.56
##    33  TG(51:3)              TG      0.56
##    34  TG(18:1/18:1/22:6)    TG      0.53
##    35  TG(53:2)              TG      0.51
##    36  TG(53:3)              TG      0.51
##    37  TG(51:1)              TG      0.43
##    38  TG(46:0)              TG      0.42
##    39  TG(46:2)              TG      0.41
##    40  TG(54:5)              TG      0.4
##    41  TG(49:2)              TG      0.4
##    42  TG(49:3)              TG      0.38
##    43  TG(56:5)              TG      0.35
##    44  TG(58:9)              TG      0.32
##    45  TG(52:5)              TG      0.31
##    46  TG(53:4)              TG      0.27
##    47  TG(56:4)              TG      0.25
##    48  TG(54:6)              TG      0.23
##    49  TG(56:3)              TG      0.23
##    50  TG(56:6)              TG      0.22
##    51  TG(47:1)              TG      0.21
##    52  TG(56:7)              TG      0.2
```

9 Quality Control Table of Lipid Measurements

Table 11: Coefficient of variation (CV; %; also called relative standard deviation) of the lipidomic features in quality control samples and study samples. Values are shown for pooled samples from the study (Pool.Study), NIST samples (NIST), long-term quality control samples from the clinic of Steno Diabetes Center Copenahgen (Pool.Clinic), and study samples (Sample). Additionally, the ratio of CVs in the study samples and in the pooled samples from the study are shown (Ratio.Sample.v.Pool). The rows of the table are sorted by this ratio.

Name	Pool.Study	NIST	Pool.Clinic	Sample	Ratio.Sample.v.Pool
PC(35:1)	4.58	21.11	13.10	31.49	6.87
SM(d36:1)	2.46	8.29	8.25	15.88	6.45
SM(d32:1)	4.60	14.49	8.78	25.76	5.60
SM(d38:1)	4.49	11.35	11.33	23.28	5.18
PC(33:1)	5.60	19.09	13.81	28.57	5.10
PC(36:5)	8.85	39.53	14.10	44.70	5.05
SM(d33:1)	4.96	16.28	8.29	24.68	4.97
PC(40:6)	5.81	18.49	11.81	28.71	4.94
PC(38:6)	5.35	18.32	8.66	26.04	4.87
TG(18:2/22:5/16:0)	9.52	52.28	13.35	45.18	4.75
SM(d41:2)	5.52	19.20	12.13	25.25	4.57
SM(d38:2)	3.98	8.20	9.05	18.00	4.52
PC(40:7)	5.98	25.03	14.38	26.59	4.44
SM(d40:2)	4.30	12.55	10.57	18.94	4.41
SM(d39:1)	6.26	18.70	12.79	26.75	4.28
SM(42:2)	5.23	16.82	9.95	21.87	4.18
TG(53:2)	8.85	25.11	14.95	36.78	4.16
TG(56:3)	8.19	24.92	16.42	33.19	4.05
SM(d18:2/24:1)	4.62	14.21	10.41	18.53	4.01
TG(54:2)	8.43	23.04	15.61	32.55	3.86
SM(d16:1/18:1)	3.59	13.45	9.34	13.76	3.83
TG(16:0/18:0/18:1)	7.96	25.73	15.81	30.28	3.80
TG(18:1/18:1/22:6)	13.71	66.72	19.28	49.94	3.64
TG(51:2)	9.02	25.03	14.04	30.22	3.35
TG(53:3)	8.08	22.50	14.99	26.78	3.31
PC(34:2)	4.12	12.86	17.26	13.66	3.31
PC(38:2)	5.92	18.78	13.49	19.50	3.29
PC(O-38:4)	5.36	17.02	18.24	16.83	3.14
TG(51:1)	10.96	22.86	15.11	32.90	3.00
TG(58:9)	14.28	39.16	22.76	42.32	2.96
TG(54:3)	5.81	12.24	8.64	17.04	2.93
PC(40:5)	6.92	17.63	14.21	20.13	2.91
TG(50:1)	7.94	16.60	13.24	22.98	2.89
TG(14:0/16:0/18:1)	8.72	18.91	13.83	25.00	2.87
SM(d40:1)	5.39	13.27	12.51	14.66	2.72
TG(46:1)	10.56	28.58	15.34	27.81	2.63
TG(18:1/18:1/18:1)	8.59	16.34	12.97	22.40	2.61
TG(49:1)	11.22	32.01	15.46	29.26	2.61
SM(d41:1)	7.17	17.41	13.91	18.49	2.58
PC(37:2)	11.27	30.48	17.21	28.68	2.54

Name	Pool.Study	NIST	Pool.Clinic	Sample	Ratio.Sample.v.Pool
TG(51:3)	9.13	24.67	15.63	23.12	2.53
PC(O-36:2)	6.98	18.49	14.84	17.32	2.48
TG(18:1/12:0/18:1)	9.46	22.90	14.15	23.42	2.48
TG(46:2)	11.11	25.71	18.43	27.42	2.47
TG(53:4)	8.52	19.69	17.40	20.76	2.44
TG(50:3)	6.00	17.88	11.92	14.38	2.40
TG(56:5)	11.32	22.83	15.72	27.03	2.39
TG(16:0/18:2/22:6)	22.15	55.17	23.12	52.39	2.37
PC(38:3)	4.94	13.44	11.56	11.65	2.36
PC(32:1)	6.60	10.31	14.00	15.42	2.34
TG(49:2)	9.96	24.15	15.98	22.42	2.25
TG(50:0)	14.25	29.08	24.34	31.64	2.22
PC(32:2)	8.10	23.93	14.89	17.94	2.22
TG(14:0/18:1/18:1)	7.11	15.29	10.51	15.67	2.20
PC(16:0e/18:1(9Z)	7.98	18.64	10.33	17.56	2.20
PC(34:1)	3.81	5.23	6.74	8.36	2.19
TG(52:2)	6.20	8.49	7.91	13.39	2.16
TG(56:7)	10.32	15.38	13.93	22.21	2.15
SM(d18:1/24:0)	7.42	17.68	14.58	15.88	2.14
TG(56:4)	14.65	18.86	17.96	30.45	2.08
TG(56:6)	10.49	13.29	12.89	21.69	2.07
TG(45:0)	4.35	5.83	7.68	8.93	2.05
PC(O-36:3)	10.22	21.62	13.02	20.89	2.04
PC(O-38:5)	6.62	16.21	11.95	13.42	2.03
TG(18:0/18:1/20:4)	17.37	20.86	24.04	35.02	2.02
TG(48:3)	14.00	31.86	23.28	28.16	2.01
TG(50:2)	6.64	11.62	8.90	13.15	1.98
TG(52:4)	5.77	9.58	9.03	11.30	1.96
TG(16:0/18:2/18:3)	10.31	25.12	17.29	19.53	1.89
TG(14:0/18:2/18:2)	9.76	23.95	16.77	18.11	1.86
PC(38:5)	4.66	9.84	8.25	8.41	1.81
PC(O-38:6)	6.84	13.50	10.21	12.33	1.80
TG(46:0)	11.75	22.81	13.95	20.93	1.78
PC(34:3)	21.13	37.19	24.31	37.18	1.76
TG(18:2/18:2/18:2)	15.81	26.85	16.17	27.46	1.74
TG(18:1/18:2/18:2)	8.62	18.14	15.07	14.70	1.71
LPC(18:1)	10.23	3.46	7.96	17.13	1.67
TG(54:6)	10.98	16.72	13.81	18.02	1.64
SM(d34:1)	4.28	4.32	6.66	7.01	1.64
TG(16:0/22:5/18:1)	15.96	16.91	17.01	26.06	1.63
PC(36:4)	7.56	6.28	14.96	12.35	1.63
TG(54:4)	6.03	10.07	9.33	9.81	1.63
TG(54:5)	7.22	11.02	9.98	11.74	1.63
TG(16:0/18:2/18:2)	7.35	13.21	14.55	11.91	1.62
TG(52:5)	12.90	19.07	16.36	20.56	1.59
PC(35:2)	16.03	20.39	24.54	25.13	1.57
LPC(16:0)	5.91	4.49	6.12	9.24	1.56
TG(49:3)	3.72	4.23	4.02	5.47	1.47
PC(38:4)	5.25	7.04	8.01	7.59	1.45
PC(O-36:5)	8.02	11.89	9.86	11.36	1.42
PC(36:2)	4.30	5.16	6.61	5.81	1.35
SM(d36:2)	5.59	6.27	8.14	7.51	1.34

Name	Pool.Study	NIST	Pool.Clinic	Sample	Ratio.Sample.v.Pool
LPC(18:0)	9.79	4.91	6.88	13.15	1.34
PC(32:0)	6.15	5.42	6.95	7.97	1.30
PC(O-36:4)	11.70	15.74	11.88	14.83	1.27
TG(47:1)	23.48	32.44	28.18	28.16	1.20
TG(52:3)	7.60	8.74	9.63	9.10	1.20
LPC(16:1)	9.77	5.90	11.40	10.54	1.08
PC(36:3)	10.12	9.17	11.57	10.82	1.07
PC(O-34:3)	15.20	15.27	10.86	15.98	1.05
LPC(20:4)	13.51	8.79	10.65	14.00	1.04
TG(18:2/18:1/18:1)	16.72	14.52	14.33	16.62	0.99
PC(O-34:2)	26.89	18.12	12.97	23.00	0.86
LPC(18:2)	10.68	6.03	9.06	9.01	0.84
TG(18:1/18:1/16:0)	31.60	13.28	22.78	25.92	0.82
TG(18:2/18:1/16:0)	33.75	19.63	24.84	26.83	0.79

10 Appendix

```
## R version 3.4.2 (2017-09-28)
## Platform: x86_64-w64-mingw32/x64 (64-bit)
## Running under: Windows 7 x64 (build 7601) Service Pack 1
##
## Matrix products: default
##
## locale:
## [1] LC_COLLATE=Danish_Denmark.1252  LC_CTYPE=Danish_Denmark.1252
## [3] LC_MONETARY=Danish_Denmark.1252 LC_NUMERIC=C
## [5] LC_TIME=Danish_Denmark.1252
##
## attached base packages:
## [1] stats     graphics  grDevices utils     datasets  methods   base
##
## other attached packages:
## [1] stringr_1.2.0
##
## loaded via a namespace (and not attached):
##  [1] Rcpp_0.12.13        lattice_0.20-35     tidyr_0.8.0
##  [4] zoo_1.8-0           gtools_3.5.0        assertthat_0.2.0
##  [7] digest_0.6.12       psych_1.7.8         R6_2.2.2
## [10] survminer_0.4.2     plyr_1.8.4          evaluate_0.14
## [13] ggplot2_3.2.0       highr_0.6           gplots_3.0.1
## [16] rlang_0.3.4         lazyeval_0.2.0      data.table_1.10.4-3
## [19] gdata_2.18.0        Matrix_1.2-11       rmarkdown_1.13
## [22] labeling_0.3        splines_3.4.2       readr_1.1.1
## [25] foreign_0.8-69      munsell_0.5.0       broom_0.4.3
## [28] compiler_3.4.2      xfun_0.7            pkgconfig_2.0.1
## [31] mnormt_1.5-5        htmltools_0.3.6     tidyselect_0.2.3
## [34] tibble_1.3.4        gridExtra_2.3       km.ci_0.5-2
## [37] withr_2.0.0         dplyr_0.7.4         ggpubr_0.1.6
## [40] bitops_1.0-6        grid_3.4.2          nlme_3.1-131
## [43] xtable_1.8-2        gtable_0.2.0        magrittr_1.5
## [46] KMsurv_0.1-5        scales_1.0.0        KernSmooth_2.23-15
## [49] stringi_1.1.5       reshape2_1.4.2      bindrcpp_0.2
## [52] limma_3.32.10       survMisc_0.5.4      cowplot_0.9.2
## [55] RColorBrewer_1.1-2  tools_3.4.2         cmprsk_2.2-7
## [58] forcats_0.2.0       glue_1.2.0          purrr_0.2.4
## [61] hms_0.4.0           dummies_1.5.6       parallel_3.4.2
## [64] survival_2.41-3     yaml_2.2.0          colorspace_1.3-2
## [67] caTools_1.17.1      corrplot_0.84       knitr_1.23
## [70] bindr_0.1           haven_1.1.0
```
